# Cyclopropylmethyl Boronic Esters as General Reagents in Transition-Metal Catalyzed Homoallylation Reactions

**DOI:** 10.1021/jacs.5c15781

**Published:** 2025-10-31

**Authors:** Blanca Lozano, Javier Teresa, Israel Fernández, Mariola Tortosa

**Affiliations:** † Organic Chemistry Department, Universidad Autónoma de Madrid (UAM), 28049 Madrid, Spain; ∥ Organic Chemistry Department, Faculty of Chemistry, Complutense University of Madrid, 28040 Madrid, Spain; § Institute for Advanced Research in Chemical Sciences (IAdChem), Universidad Autónoma de Madrid, 28049 Madrid, Spain

## Abstract

Herein we disclose the use of cyclopropylmethyl boronates as general reagents in Negishi-type homoallylation reactions. This strategy provides a novel approach to generate enantioenriched homoallyl-Zn species through boron-to-zinc transmetalation. Subsequent sp^2^–sp^3^ cross-coupling offers a platform for the preparation of arenes, ketones, and 1,5-dienes containing a chiral homoallylic scaffold. The method has been applied to the late-stage functionalization of known drugs and the preparation of precursors of biologically relevant compounds. Mechanistic experiments and DFT calculations provide insight into the transmetalation/ring-opening sequence.

## Graphical abstract

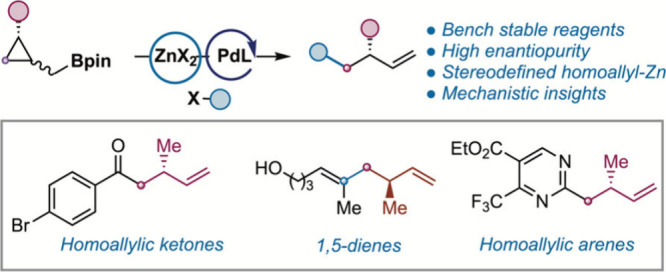

Stereodefined organoboron compounds play an important role in the asymmetric synthesis of organic molecules.^1,2^ In particular, allylic boronates have been widely used in total synthesis for the asymmetric allylation of aldehydes and ketones.^3^ In contrast, the corresponding homoallylation using the cyclopropanated analogs of allylic boronates has only been disclosed recently. In a series of elegant papers, Krauss described the use of stereodefined cyclopropylmethyl boronates to promote the homoallylation of aldehydes (Scheme 1A).^4^

**Scheme 1:**
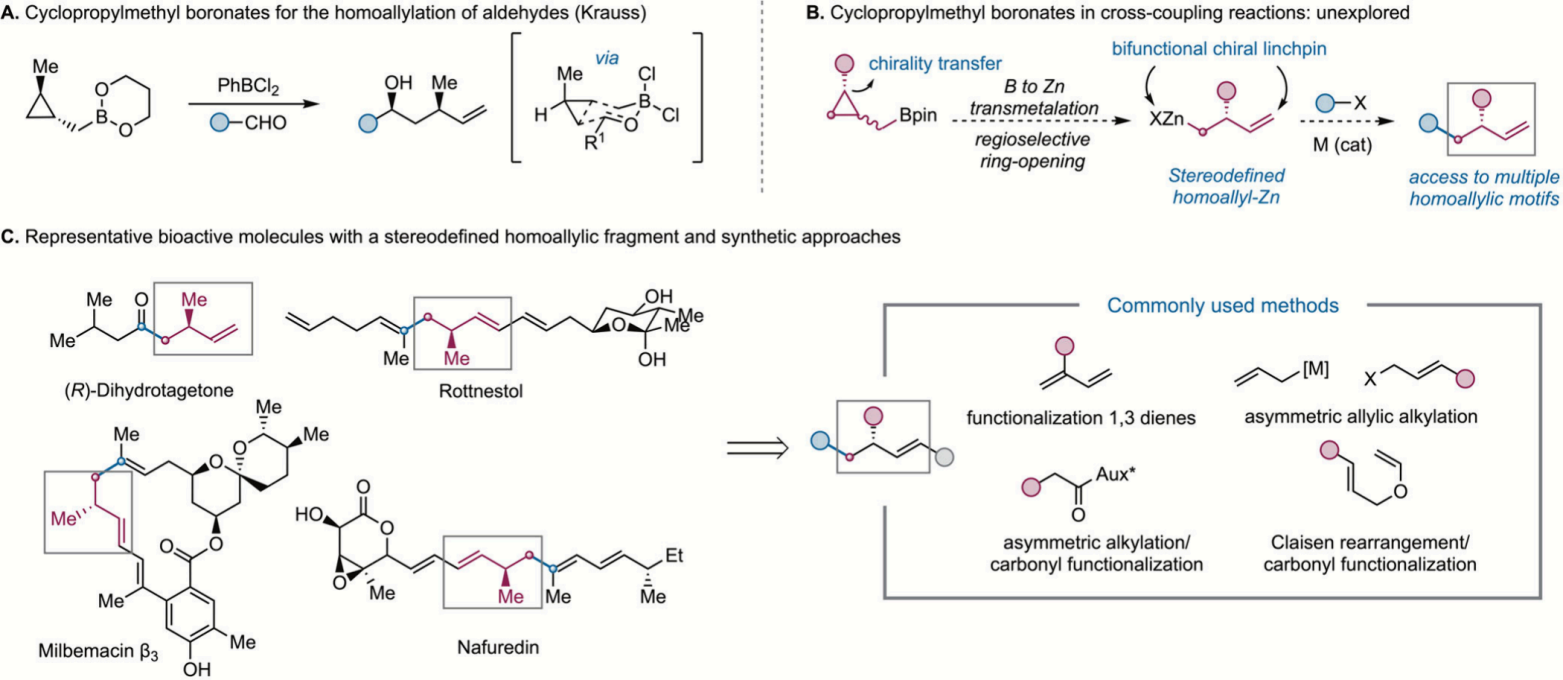
Cyclopropylmethyl Boronates as Homoallylation Reagents

Moved by our previous experience in the preparation of boron-containing cyclopropanes,^5^ we envisioned the use of cyclopropylmethyl boronates as general reagents in transition-metal-catalyzed homoallylations. In particular, we chose the Negishi cross-coupling reaction to evaluate this idea due to the enormous synthetic potential of this transformation.^6^ Based on the pioneering work by Morken on the stereospecific transmetalation of secondary alkyl boronates,^7^ we hypothesized that a boron-to-zinc transmetalation^8^ followed by regioselective ring opening^9,10^ would provide an easy access to stereodefined homoallyl-Zn species from bench-stable reagents (Scheme 1B).^11^ Although chiral β-substituted homoallyl-Zn species have been described before, the general preparation and use of these species remains challenging.^12,13^ Regarding the regioselective ring opening, we were encouraged by the previous work by Marek on the generation of homoallylic metal species from stereodefined alkylidenecyclopropanes with complete preservation of the stereochemical integrity.^14^ From the homoallyl-Zn species, the implementation of a subsequent sp^3^–sp^2^ cross-coupling would offer a straightforward platform to install a stereodefined homoallylic motif in a large variety of biologically active compounds such as those shown in Scheme 1C. Previous methods to install this fragment in complex molecules include the asymmetric functionalization of 1,3-dienes,^15^ asymmetric allylic alkylation,^16^ Claisen rearrangement,^17^ and asymmetric alkylation followed by carbonyl functionalization (Scheme 1C).^18^ Compared to these approaches, the use of cyclopropylmethyl boronates would offer a different synthetic disconnection to assemble such molecules. Additionally, different types of stereodefined homoallylic fragments could be prepared from a common reagent simply by selecting the appropriate electrophile in the cross-coupling event (Scheme 1B).

We recognized from the outset that a synthetically useful homoallylation would necessarily require easy and general access to the reagents. We envisioned that a copper-catalyzed stereospecific borylative cyclization of an activated homoallylic alcohol^19^ could provide direct access to enantioenriched *cis*/*trans*-cyclopropylmethyl boronates with a defined stereocenter (Scheme 2). In principle, these reagents could be used as *cis*/*trans* mixtures on the carbon bearing the −CH_2_Bpin unit. Enantioenriched homoallylic alcohols seemed to be ideal precursors because they are readily available in both enantiomeric forms from easily accessible aldehydes^20^ or enantioenriched epoxides.^21^ Scheme 2 shows the implementation of this strategy to prepare enantiopure boronate **1a**. Starting from commercially available (*S*)-propylene oxide, copper-catalyzed vinylmagnesium bromide addition followed by quenching with a chlorophosphate provided an activated alcohol that was used directly in the next step without purification. Then a copper-catalyzed borylative cyclization using conditions described by Ito^19^ for homoallylic bromides provided a *cis*/*trans* mixture of bench-stable cyclopropylmethyl boronate **1a** in 90% yield over three steps after distillation.^19^ Importantly, this sequence was scalable, and compound **1a** preserved the enantiopurity of the starting epoxide.^22^

**Scheme 2:**
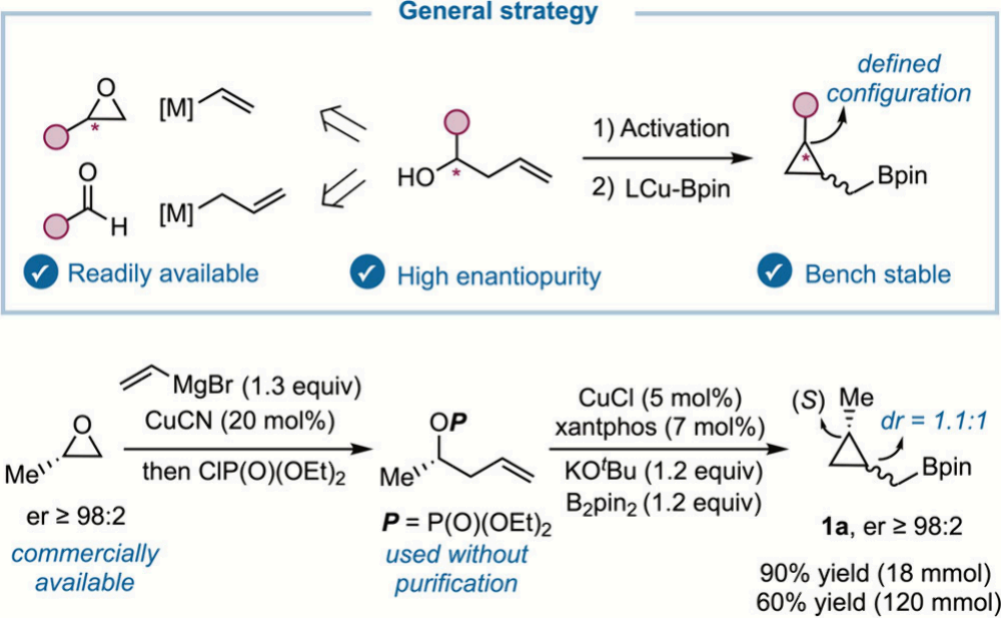
Synthesis of Enantioenriched Reagents

From **1a**, we first tried the direct use of the boron-ate complex formed by addition of *s*-BuLi in a Suzuki–Miyaura cross-coupling^23^ with an aryl bromide using Pd­(OAc)_2_ with SPhos as the ligand at 60 °C (Table 1, entry 1).^24^ Unfortunately, we observed low conversion of **1a** to an inseparable mixture of open and closed cross-coupling products **2a** and **2a′**. *t*-BuLi provided better results but did not avoid the formation of cyclopropane **2a′** (Table 1, entry 2). Gratifyingly, when *t*-BuLi and Zn­(OAc)_2_ were used at 60 °C to promote a boron-to-Zn transmetalation,^7b^ ring-opened product **2a** was obtained in excellent yield without formation of **2a′** (Table 1, entry 3). The use of 1 equiv of **1a** instead of 1.5 equiv provided a lower yield (Table 1, entry 4). Importantly, preformation of the alkyl-Zn species was essential (Table 1, entry 5), as adding the zinc salt and the palladium catalyst simultaneously afforded results similar to those obtained without Zn. Alternatively, ZnCl_2_ could be used with the same efficiency.

**Table 1:**
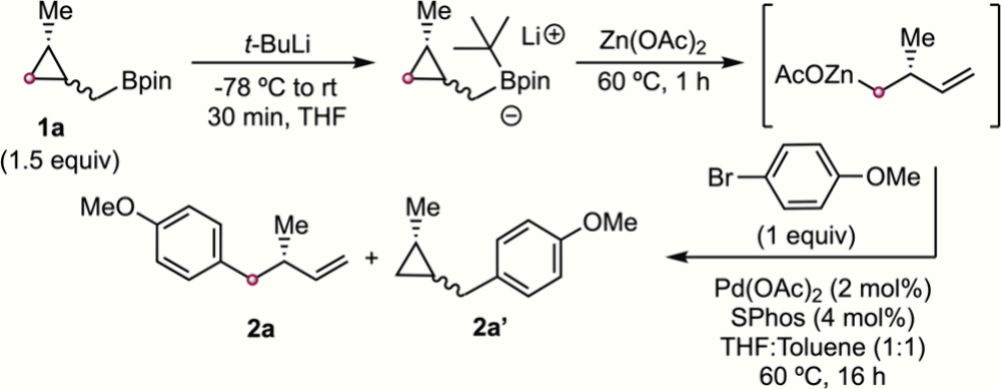
Optimization of the Homoallylation

entry	deviation from standard conditions^a^	yield of **2a** (%)^b^	**2a**:**2a′** ^c^
1	*s*-BuLi, no zinc salt	15	58:42
2	no zinc salt	60	82:18
3	–	92	>98:2
4	1 equiv of **1a**	60	>98:2
5	Zn(OAc)_2_/Pd(OAc)_2_ added together	48	82:18
6	ZnCl_2_	87	>98:2

a Standard conditions: **1a** (1.5 equiv), *t*-BuLi (1.5 equiv), Zn­(OAc)_2_ (1.5 equiv), Pd­(OAc)_2_ (2 mol %), SPhos (4 mol %), ArBr (1 equiv), THF/toluene, 0.2 M, 60 °C.

b Yields were calculated by ^1^H NMR.

c Ratios were calculated by GC-MS.

With the optimized conditions for an effective transmetalation/ring-opening sequence, we studied the generality of the homoallylation with different cyclopropylmethyl boronates (**1a**–**1h**) and different electrophiles (Scheme 3). Enantioenriched homoallylated arenes **2a**–**2k** with electron-donating and -withdrawing groups were prepared in good yields. Moreover, several heterocycles with a stereodefined homoallylic chain (**2l**–**2q**) were efficiently synthesized using either Pd­(OAc)_2_/SPhos (**2o**, **2q**) or PdG3CPhos (2 mol %) (**2l**–**n**, **2p**) as the catalyst.^25^

**Scheme 3:**
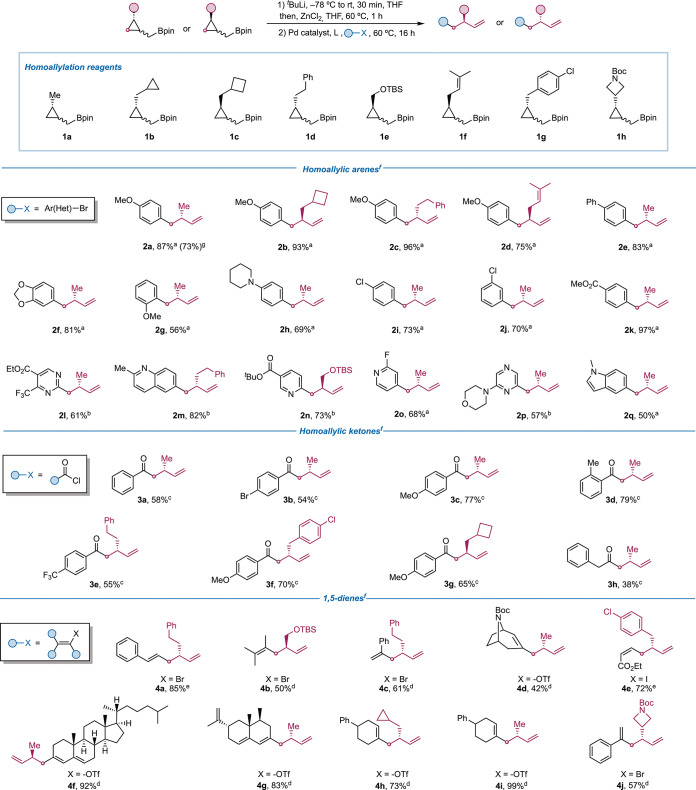
Scope of the Homoallylation

a Conditions A: **1** (1.5 equiv), *t*-BuLi (1.5 equiv), Zn­(OAc)_2_ (1.5 equiv), ArBr (0.2 mmol), Pd­(OAc)_2_ (2 mol %), SPhos (4 mol %), THF/Toluene, 0.2 M, 60 °C.
b Conditions B: **1** (1.5 equiv), *t*-BuLi (1.5 equiv), ZnCl_2_ (1.5 equiv), (Het)­ArCl (0.2 mmol), PdG3CPhos (2 mol %), CPhos (2 mol %), THF, 0.2 M, 60 °C.
c Conditions C: **1** (1.5 equiv), *t*-BuLi (1.5 equiv), ZnCl_2_ (1.5 equiv), AcCl (0.2 mmol), Pd­(PPh_3_)_4_ (5 mol %), 0.2 M, 60 °C.
d Conditions D: **1** (1.5 equiv), *t*-BuLi (1.5 equiv), ZnCl_2_ (1.5 equiv), alkenyl-X (0.2 mmol), Pd­(OAc)_2_ (2 mol %), CPhos (4 mol %), THF, 0.2 M, 60 °C.
e Conditions E: **1** (1.5 equiv), *t*-BuLi (1.5 equiv), ZnCl_2_ (1.5 equiv), alkenyl-X (0.2 mmol), PdCl_2_(Amphos)_2_ (2 mol %), NMI (2 equiv), THF, 0.2 M, 60 °C.
f Isolated yield.
g 5 mmol scale.

We then focused on the use of acyl chlorides and alkenyl (pseudo)­halides to prepare stereodefined homoallylic ketones and 1,5-dienes such as those present in the molecules shown in Scheme 1C. After some optimization, we observed that ZnCl_2_ provided better results than Zn­(OAc)_2_ for these electrophiles.^26^ Using acyl chlorides and 5 mol % Pd­(PPh_3_)_4_,^27^ enantiopure aryl ketones (**3a**–**3g**) were successfully prepared. Aliphatic homoallylic ketone **3h** was also synthesized in moderate yield. The reaction with vinyl halides and triflates in the presence of Pd­(OAc)_2_ (2 mol %) and CPhos (4 mol %) provided the corresponding 1,5-dienes (**4b**–**4d**, **4f**–**4j**).^24^ For compounds **4a** and **4e**, Lipshutz conditions for Negishi cross-coupling (PdCl_2_Amphos_2_/*N*-methylimidazole) were used to avoid isomerization of the alkene.^28^ The preparations of compounds **3f**, **4e**, and **4j** are noteworthy because they highlight the compatibility of an aryl chloride and a Boc protecting group with the *t*-BuLi used to form the ate complex starting from reagents **1g** and **1h**.^29^

We also applied the homoallylation conditions to the late-stage functionalization of known drugs and to the preparation of precursors of biologically active compounds (Scheme 4). Starting from brompheniramine, vandetanib, and drug-like Merck informer X17, homoallylated products **2r**, **2s**, and **2t** were prepared in moderate to high yields. Additionally, (*S*)-flobufen precursor^15^ **3i** was prepared using cyclopropylmethyl boronate **1a** and a commercially available carboxylic acid. Finally, we applied our method toward the synthesis of fragment **4k**, which has been used before as intermediate in the total synthesis of rottnestol.^16^ Starting from protected alkynol **6**, vinyl boronic ester **7** was prepared through copper-catalyzed borylation. To control the regioselectivity in this transformation, we reoptimized the conditions developed by Kanai^30^ for the carboboration of internal alkynes. Interestingly, we found that the use of *t*-BuOH as a proton donor was key to provide high levels of regioselectivity. Compound **7** was transformed into vinyl iodide **8**, which was used as a cross-coupling partner with **1a** to provide enantiomerically enriched **4k** as a single diastereomer.

**Scheme 4:**
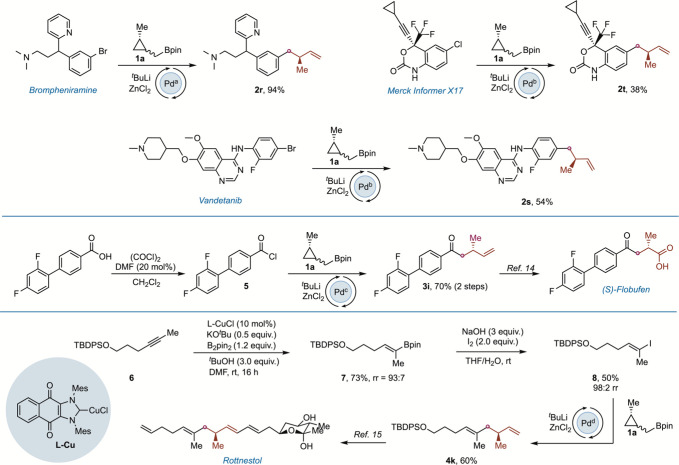
Late-Stage Functionalization and Biologically Relevant Intermediates

a Conditions A in Scheme 3.
b Conditions B in Scheme 3.
c Conditions C in Scheme 3.
d Conditions D in Scheme 3.

To get insight into the transmetalation/ring-opening process, we performed different ^11^B and ^1^H NMR experiments. After addition of *t*-BuLi to **1a**, the ^1^H NMR spectrum showed quantitative formation of boron-ate complex **I** with no sign of olefinic protons, indicating that the ring opening takes place after transmetalation (Scheme 5A, right). We envisioned two possible pathways for the transmetalation/ring-opening sequence (Scheme 5B, left). One would involve a concerted process through a six-membered transition state, reminiscent of that proposed for the boron-to-zinc transmetalation in allyl boronates.^31^ A second possibility would be stepwise transmetalation/β-carbon elimination.^32^ When ZnCl_2_ was added to boron-ate complex **I** under the reaction conditions (60 °C), complete transmetalation was observed by ^11^B NMR after 10 min, with disappearance of the sp^3^-hybridized boron signal at 8.8 ppm and appearance of the *t*-BuBpin signal at 35.0 ppm (Scheme 5A, middle). The ^1^H NMR spectrum at 60 °C showed the clean formation of, presumably, homoallyl-Zn intermediate **III**,^33^ while cyclopropylmethyl-Zn species **IV** could not be detected (Scheme 5, right).

**Scheme 5:**
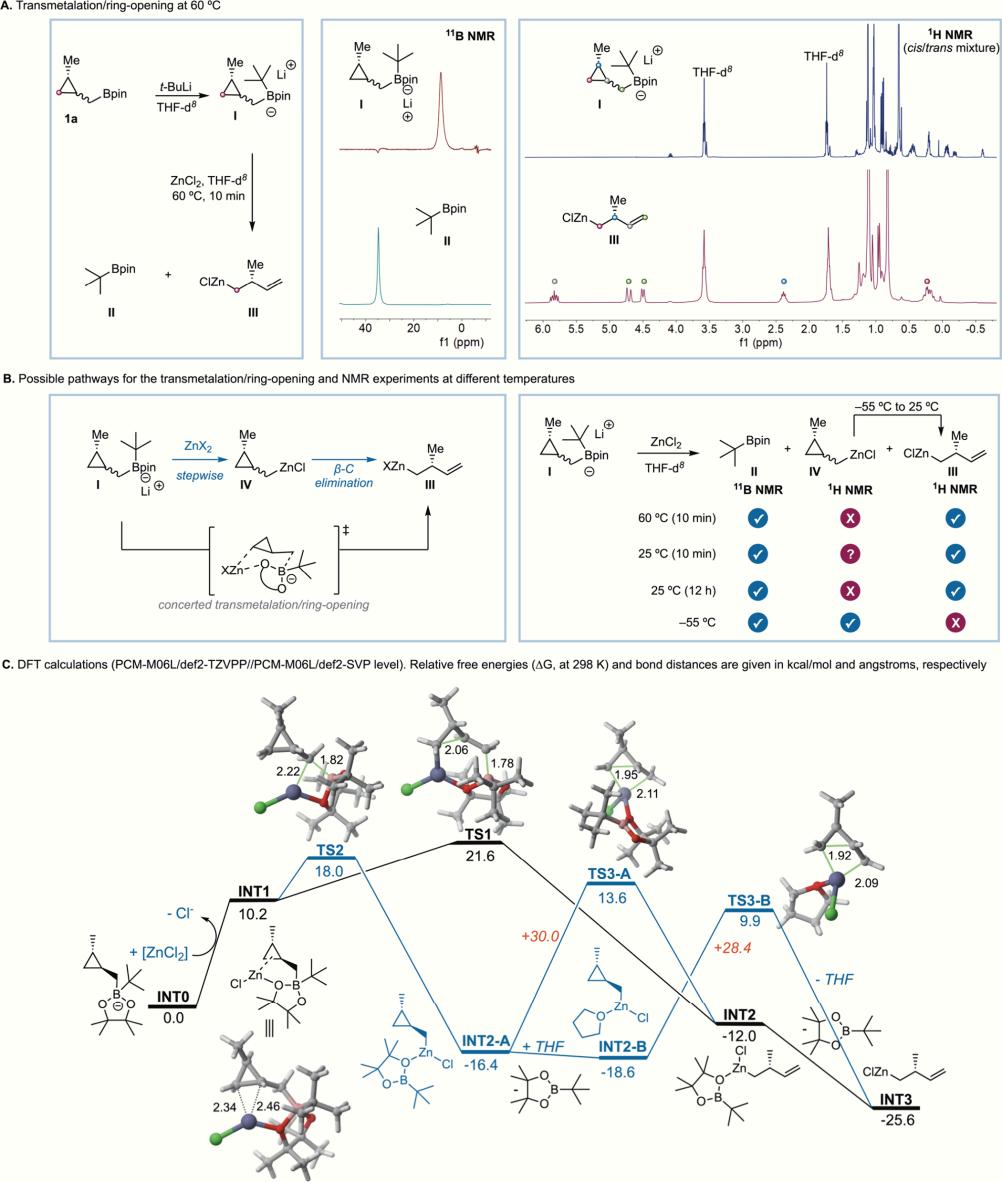
Studies of the Transmetalation/Ring-Opening Process by ^1^H and ^11^B NMR Experiments and DFT Calculations

At 25 °C, ^11^B NMR showed again complete transmetalation (Scheme 5B, right). At short times the ^1^H NMR spectrum showed a complex mixture indicating the coexistence of different species at 25 °C. From this mixture we could clearly identify the olefinic protons of complex **III**. This mixture evolved overnight to a clean spectrum of presumably complex **III**. Finally, at −55 °C the ^11^B NMR spectrum showed almost complete transmetalation with the appearance of *t*-BuBPin, while the ^1^H NMR spectrum did not show any signals of olefinic protons. This result was indicative of the formation of plausible cyclopropylmethylzinc **IV** at low temperature. Indeed, as the temperature increased in the NMR tube, the closed-ring products evolved cleanly to the formation of apparently homoallyl-Zn **III**. From these experiments, we concluded that at lower temperatures the stepwise mechanism seems to be operating.

To get further insight, density functional theory (DFT) calculations at the PCM-M06L/def2-TZVPP//PCM-M06L/def2-SVP level were carried out from the *trans* diastereomer of cyclopropylmethyl boronate **1a**. To this end, our calculations started from the anionic intermediate **INT0** derived from the reaction of *trans*-cyclopropylmethyl boronate **1a** with *t*-BuLi (Scheme 5, bottom). Initially, this species reacts with the zinc salt to produce the starting neutral intermediate **INT1** via replacement of a chloride ligand by **INT0**. This complex is formed upon coordination of an oxygen atom of the Bpin fragment to the Zn atom, which is also weakly bonded to the C–C bond of the cyclopropyl moiety in σ-type bonding. From **INT1**, two alternative pathways can be envisaged, namely, a concerted transmetalation/ring-opening reaction and the corresponding stepwise mechanism. Our calculations indicate that the concerted process takes place via **TS1**, a saddle point associated with the simultaneous rupture of the B–CH_2_ and cyclopropyl C–C bonds, with a low barrier of only 11.5 kcal/mol. This process leads to the highly exergonic (Δ*G* = −22.2 kcal/mol) formation of homoallylic intermediate **INT2**, which easily releases *t*-BuBpin to produce the key Zn­(II) intermediate **INT3**, also in a highly exergonic transformation (Δ*G* = −13.8 kcal/mol). This concerted mechanism is therefore consistent with the experimental (NMR) detection of *t*-BuBpin and the homoallyl-Zn complex. Alternatively, we also located a lower-lying transition state, **TS2** (Δ*G* ^⧧^ = 7.9 kcal/mol), which is associated exclusively with the transmetalation reaction, i.e., with Zn–CH_2_ bond formation with concomitant B–CH_2_ bond rupture. This step leads to the Zn­(II) intermediate **INT2-A** in a highly exergonic reaction (Δ*G* = −26.6 kcal/mol), therefore indicating that the first step of this stepwise pathway is both kinetically and thermodynamically favored over the (also feasible) concerted mechanism (a similar result was found when a molecule of THF is coordinated to the metal atom; see the Supporting Information). Intermediate **INT2-A** may then evolve to the same intermediate **INT2** via **TS3-A** with a relatively high barrier of 30.0 kcal/mol in a slightly endergonic process (Δ*G* = 4.4 kcal/mol). A similar barrier was computed when the boronate ligand was replaced either by a molecule of THF (Δ*G* ^⧧^ = 28.4 kcal/mol, via **TS3-B**) or LiCl present in the reaction (Δ*G* ^⧧^ = 27.5 kcal/mol, not shown in the computed profile). Either way, the kinetic and thermodynamic preference for the formation of **INT2-A** together with the relatively high barrier and endergonicity associated with the subsequent cyclopropyl ring opening is fully consistent with the exclusive detection of ring-closed species in the kinetic control experiment (−55 °C). On the other hand, under thermodynamic control (rt or 60 °C), **INT3** could be formed either through the concerted mechanism or via the stepwise pathway, as the barrier associated with the cyclopropane ring opening (via **TS3**) can be easily reached.

In summary, a novel way to prepare enantioenriched homoallyl-Zn species from stereodefined cyclopropylmethyl boronates has been developed. Their use in subsequent Negishi cross-coupling reactions provides a general and straightforward platform to introduce the homoallyl fragment with a defined stereocenter in complex molecules. We expect that this transformation may open the door to the use of these reagents in more challenging sp^3^–sp^3^ cross-coupling reactions as well as in further transition-metal-catalyzed transformations.

## Supplementary Material



## Data Availability

The data underlying this study are available in the published article and its Supporting Information.

## Notes

General procedures, product characterization, and copies of NMR spectra (PDF)

## References

[1] Boronic Acids: Preparation and Applications in Organic Synthesis, Medicine and Materials; Hall, D. G. , Ed.;Wiley-VCH: Weinheim, Germany, 2011.

[2] Aggarwal V. K., Sandford C. (2017). Stereospecific functionalizations and transformations
of secondary and tertiary boronic esters. Chem.
Commun..

[3] Yus M., González-Gómez J. C., Foubelo F. (2011). Catalytic Enantioselective
Allylation of Carbonyl Compounds
and Imines. Chem. Rev..

[4] Pei W., Krauss I. J. (2011). Homoallylboration and Homocrotylboration
of Aldehydes. J. Am. Chem. Soc..

[5] Parra A., Amenós L., Guisán-Ceinos M., López A., García Ruano J.
L., Tortosa M. (2014). Copper-Catalyzed
Diastereo- and Enantioselective Desymmetrization of Cyclopropenes:
Synthesis of Cyclopropylboronates. J. Am. Chem.
Soc..

[6] Haas D., Hammann J. M., Greiner R., Knochel P. (2016). Recent Developments
in Negishi Cross-Coupling Reactions. ACS Catal..

[7] Xu N., Liang H., Morken J. P. (2022). Copper-Catalyzed
Stereospecific Transformations of Alkylboronic Esters. J. Am. Chem. Soc..

[8] Boudier A., Flachsmann F., Knochel P. (1998). Stereoselective Preparation
and Reaction of Chiral Secondary Cycloalkyl- and Alkyl-Zinc Reagents. Synlett.

[9] Lambert C., Schleyer P. v. R. (1994). Are Polar Organometallic Compounds
“Carbanions”? The Gegenion Effect on Structure and Energies
of Alkali-Metal Compounds. Angew. Chem., Int.
Ed. Engl..

[10] Cohen Y., Cohen A., Marek I. (2021). Creating Stereocenters
within Acyclic Systems by C-C Bond Cleavage of Cyclopropanes. Chem. Rev..

[11] Knochel P., Singer R. D. (1993). Preparation and
reactions of polyfunctional organozinc reagents in organic synthesis. Chem. Rev..

[12] Charette A., Naud J. (1998). Regioselective Opening
of Substituted (Cyclopropylmethyl)­Lithiums Derived from Cyclopropylmethyl
Iodides. Tetrahedron Lett..

[13] Roy S. R., Didier D., Kleiner A., Marek I. (2016). Diastereodivergent
combined carbometalation/zinc homologation/C-C fragmentation reaction
as an efficient tool to prepare acyclic allylic quaternary carbon
stereocenters. Chem. Sci..

[14] Simaan S., Goldberg A. F. G., Rosset S., Marek I. (2010). Metal-Catalyzed Ring-Opening of Alkylidenecyclopropanes: New Access
to Building Blocks with an Acyclic Quaternary Stereogenic Center. Chem.—Eur. J..

[15] Parsutkar M. M., RajanBabu T. V. (2021). α- and β-Functionalized Ketones from 1,3-Dienes
and Aldehydes: Control of Regio- and Enantioselectivity in Hydroacylation
of 1,3-Dienes. J. Am. Chem. Soc..

[16] Meng F., McGrath K. P., Hoveyda A. H. (2014). Multifunctional
Organoboron Compounds
for Scalable Natural Product Synthesis. Nature.

[17] Li M., O’Doherty G. A. (2006). De Novo Asymmetric Synthesis of Milbemycin
β_3_ via an Iterative Asymmetric Hydration Approach. Org. Lett..

[18] Crimmins M. T., Al-awar R. S., Vallin I. M., Hollis W. G., O’Mahony R., Lever J. G., Bankaitis-Davis D. M. (1996). Asymmetric
Total Synthesis of (+)-Milbemycin D. J. Am.
Chem. Soc..

[19] Kubota K., Yamamoto E., Ito H. (2013). Copper­(I)-Catalyzed
Borylative *exo*-Cyclization of Alkenyl Halides Containing
Unactivated
Double Bond. J. Am. Chem. Soc..

[20] Kim I. S., Ngai M.-Y., Krische M. J. (2008). Enantioselective
iridium-catalyzed allylation from the alcohol or aldehyde oxidation
level via transfer hydrogenative coupling of allyl acetate: departure
from chirally modified allyl metal reagents in carbonyl addition. J. Am. Chem. Soc..

[21] Schaus S. E., Brandes B. D., Larrow J. F., Tokunaga M., Hansen K. B., Gould A. E., Furrow M. E., Jacobsen E. N. (2002). Highly
Selective Hydrolytic Kinetic Resolution of Terminal Epoxides Catalyzed
by Chiral (salen)­Co­(III) Complexes. Practical Synthesis of Enantioenriched
Terminal Epoxides and 1,2-Diols. J. Am. Chem.
Soc..

[22] The enantiopurity of **1a** was established from the enantiopurity of cross-coupling product **2k**. See the Supporting Information for details.

[23] Zou G., Falck J. R. (2001). Suzuki-Miyaura cross-coupling
of lithium *n*-alkylborates. Tetrahedron Lett..

[24] Han C., Buchwald S. L. (2009). Negishi Coupling
of Secondary Alkyl zinc Halides with Aryl Bromides and Chlorides. J. Am. Chem. Soc..

[25] Yang Y., Niedermann K., Han C., Buchwald S. L. (2014). Highly Selective
Palladium-Catalyzed Cross-Coupling of Secondary Alkylzinc Reagents
with Heteroaryl Halides. Org. Lett..

[26] Eckert P., Sharif S., Organ M. G. (2021). Salt to
Taste: The Critical Roles
Played by Inorganic Salts in Organozinc Formation and in the Negishi
Reaction. Angew. Chem., Int. Ed..

[27] Parida B. B., Das P. P., Niocel M., Cha J. K. (2013). *C*-Acylation of Cyclopropanols: Preparation
of Functionalized 1,4-Diketones. Org. Lett..

[28] Krasovskiy A., Lipshutz B. H. (2011). Highly Selective
Reactions of Unbiased Alkenyl Halides
and Alkylzinc Halides: Negishi-Plus Couplings. Org. Lett..

[29] The absolute configuration of the products was established from the corresponding cyclopropylmethyl boronates and confirmed by comparison of the optical rotation of compounds **3a**, **3b**, **3c**, and **3i** with those described in the literature. See the Supporting Information for details.

[30] Itoh T., Shimizu Y., Kanai M. (2016). Ligand-Enabled, Copper-Catalyzed
Regio- and Stereoselective Synthesis of Trialkylsubstituted Alkenylboronates
from Unactivated Internal Alkynes. J. Am. Chem.
Soc..

[31] Li W., Su Z., Hu C. (2013). Mechanism of Ketone
Allylation with Allylboronates as Catalyzed by Zinc Compounds: A DFT
Study. Chem.—Eur. J..

[32] O’Reilly M. E., Dutta S., Veige A. S. (2016). β-Alkyl Elimination: Fundamental
Principles and Some Applications. Chem. Rev..

[33] Although only a single set of ^1^H and ^13^C phenyl resonances were observed, the exact composition of the homoallyl-Zn species remains unknown.

