# Supplementary material for: Cyclopropylmethyl Boronic Esters as General Reagents in Transition-Metal Catalyzed Homoallylation Reactions
Source: J Am Chem Soc. 2025 Oct 31;147(45):41221–8. doi: 10.1021/jacs.5c15781 (PMC12616689; doi:10.1021/jacs.5c15781)

Supporting information

Cyclopropylmethyl Boronic Esters as General Reagents in Transition-Metal Catalyzed Homoallylation Reactions

Blanca Lozano, ‡[a] Javier Teresa, ‡[a] Israel Fernández,[b]
Mariola Tortosa*[a,c]

[a]Organic Chemistry Department, Universidad Autónoma de Madrid (UAM), 28049 Madrid, Spain.

[b]Organic Chemistry Department, Faculty of Chemistry, Complutense University of Madrid, 28040 Madrid, Spain.

[c]Institute for Advanced Research in Chemical Sciences (IAdChem), Universidad Autónoma de Madrid, Madrid 28049, Spain.

TABLE OF CONTENTS

1. General experimental details S4

2. Synthesis of cyclopropylmethyl boronic pinacol esters S6

2.1. List of synthesized cyclopropylmethyl boronic pinacol esters S6

2.2. Synthetic routes for the synthesis of cyclopropylmethyl boronic pinacol esters S6

2.2.1. Synthetic route A S6

2.2.2. Synthetic route B S7

2.3. General procedure for the synthesis of epoxides S7

2.4. Synthesis of cyclopropyl methyl boronic esters S9

2.5. tert-butyl 3-((1*S*)-2-((4,4,5,5-tetramethyl-1,3,2-dioxaborolan-2-yl)methyl)cyclopropyl)azetidine-1-carboxylate (**1h**) S14

2.6. Large scale synthesis of 4,4,5,5-tetramethyl-2-(((2*S*)-2-methylcyclopropyl) methyl)-1,3,2-dioxaborolane (**1a**) S17

3. Negishi cross-coupling reaction with aryl halides S18

3.1. Optimization of Negishi reaction conditions with aryl halides S18

3.2. General procedure A for the Negishi cross-coupling reaction with aryl halides S18

3.3. General procedure B for the Negishi cross-coupling reaction with aryl halides S19

3.4. Large scale procedure for the Negishi cross-coupling reaction S19

3.5. Characterization data of Negishi aryl-homoallylation products S20

4. Negishi cross-coupling reaction with acyl halides S31

4.1. Optimization of Negishi reaction conditions with acyl halides S31

4.2. General procedure for the Negishi cross-coupling reaction with acyl halides S31

4.3. Characterization data of Negishi homoallylation products with acyl chlorides S31

5. Negishi cross-coupling reaction with alkenyl halides S37

5.1. Optimization of Negishi reaction conditions with alkenyl halides S37

5.2. General procedure for the Negishi cross-coupling reaction with alkenyl halides S37

5.3. Characterization data of Negishi homoallylation products with vinyl (pseudo)halides S38

5.4. Synthesis of Rottnestol fragment S43

6. Mechanistic experiments by Nuclear Magnetic Resonance studies S46
6.1. Boron-ate complex Formation and Characterization S46
6.2. Boron to Zinc transmetallation at different temperatures S50
6.2.1. Transmetalation at 60 °C S50
6.2.2. Transmetalation at Room Temperature S51
6.2.3. Transmetalation at Low Temperature S53
7. Absolute configuration of the cyclopropylmethyl boronates S56
8. Proof of chirality transfer from the enantiopure allylic phosphate to the cross-coupling products.......S56
9. Computational details...S60
10. References S70
11. Characterization data S72
11.1. NMR Data: Starting Materials...S72
11.2. NMR Data: Negishi cross-coupling aryl halide’s products..S85
11.3. NMR Data: Negishi cross-coupling acyl halide’s products S107
11.4. NMR Data: Negishi cross-coupling vinyl (pseudo)halide’s products S113

1. General experimental details

Dried solvents were purchased from different commercial suppliers: tetrahydrofuran and toluene from Carlo Erba, anhydrous MeOH was purchased from Acros Organics. Diethyl ether was dried using activated 4 Å molecular sieves and stored under argon.

NMR spectra were acquired on a *Bruker Advance 300* and *500 MHz* spectrometer, 300 or 500 MHz (^{1}H NMR), at 75 or 125 MHz (^{13}C NMR), at 128.4 (^{11}B NMR) and 376 MHz (^{19}F NMR). Chemical shifts (δ) are reported in ppm relative to residual solvent signals ($CDCl_3$, δH = 7.26 ppm, δC = 77.16 ppm). ^{13}C NMR and ^{19}F spectra were acquired on a broad band decoupled mode. The following abbreviations are used to describe peak patterns when appropriate: s (singlet), d (doublet), t (triplet), q (quartet), quint (quintet), sex (sextet), sep (septet) m (multiplet), br (broad).

Analytical thin layer chromatography (TLC) was performed using pre-coated aluminum-backed plates (Merck Kieselgel 60 F254) and visualized by ultraviolet irradiation and phosphomolybdic acid dip, potassium permanganate dip or cerium ammonium molybdate dip. Flash column chromatography (FC) was performed using silica gel Merck-60 from Aldrich.

Optical rotations were measured at 20 °C on a *Perkin-Elmer 241 polarimeter* using a sodium lamp and in $CHCl_3$. The enantiomeric ratio (er) of the products was determined by HPLC using chiral columns.

High Resolution Mass Spectrometry (HRMS) were registered in an *Agilent 6520 Accurate Mass Q-TOF* or a *GCT Agilent Technologies 6890 N* spectrometers using Electronic Impact (EI^+) techniques at 70 eV and electrospray (ESI^+) or Bruker maXis IITM ($APCI^+$). Melting points were determined in a Stuart™ melting point SMP3 apparatus or a Reichert Koffler block in open capillary tubes.

Commercially available substrates were purchased from Aldrich, BLD, Alfa Aesar and Fluorochem. The epoxides used as precursors of the starting materials were purchased or synthesized:

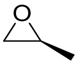	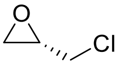	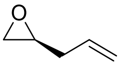
**S1a**<br>(2*S*)-2-Methyloxirane<br>*Manchester Organics*	**S1b**<br>(*S*)-(+)-Epichlorohydrin (97+% ee)<br>*Activate Scientific*	**S1c**<br>(*S*)-2-Allyloxirane
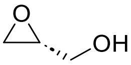	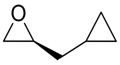	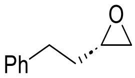
**S1d**<br>(*R*)-(+)-Glycidol (97% ee)<br>*Activate Scientific*	**S1e**<br>(*S*)-2-(Cyclopropylmethyl)oxirane	**S1f**<br>(*S*)-2-Phenethyloxirane
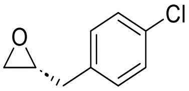	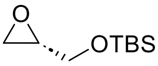	
**S1g**<br>(*R*)-2-(4-Chlorobenzyl)oxirane	**S1h**<br>*tert*-Butyldimethylsilyl (*S*)-(+)-glycidyl ether (95+% ee)<br>*BLD*	

2. Synthesis of cyclopropylmethyl boronic pinacol esters

2.1. List of synthesized cyclopropylmethyl boronic pinacol esters

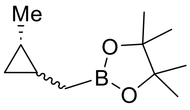

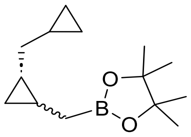

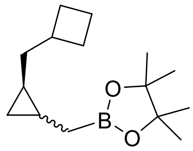

**1a**	**1b**	**1c**

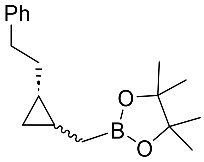

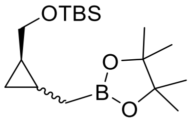

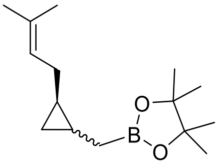

**1d**	**1e**	**1f**

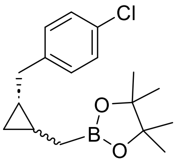

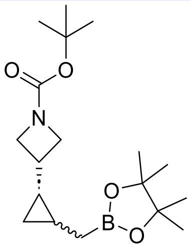

**1g**	**1h**

2.2. Synthetic routes for the synthesis of cyclopropylmethyl boronic pinacol esters

2.2.1. Synthetic route A

The below synthetic pathway was employed for the synthesis of compounds: **1a**, **1b**, **1d**, **1e** and **1g**.

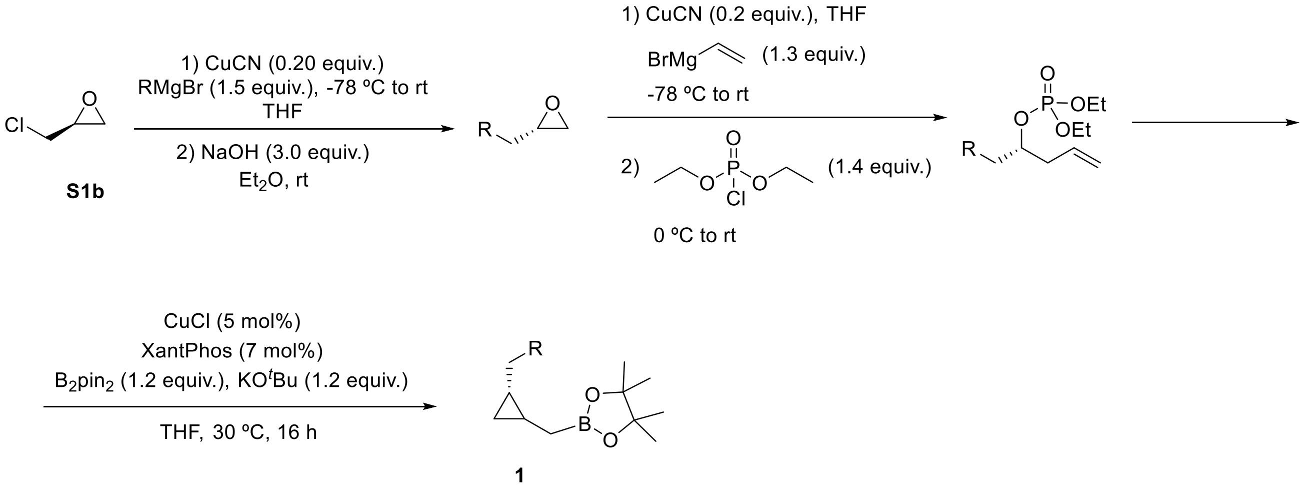

2.2.2. Synthetic route B

The below synthetic pathway was employed for the synthesis of compounds: **1c** and **1f**.

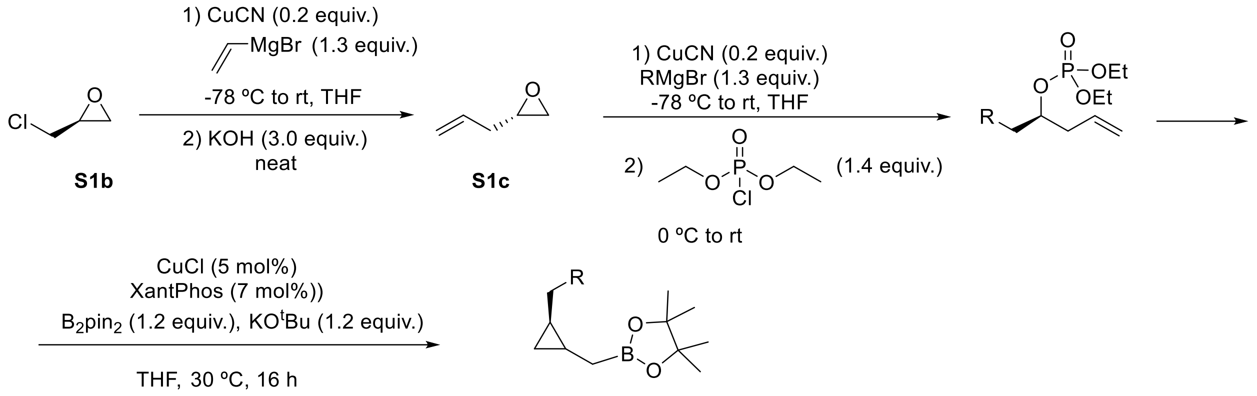

1

2.3. General procedure for the synthesis of epoxides

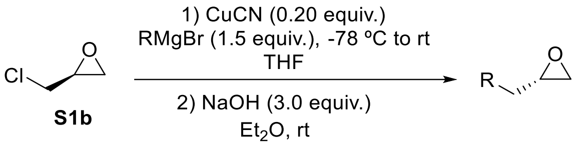

To a stirred solution of (*S*)-2-(chloromethyl)oxirane **S1b** (1 equiv.) and CuCN (0.20 equiv.) in dry THF (1 M), was added over 45 min a solution of alkylmagnesium bromide in THF (1.5 equiv.) dropwise at -78 °C under an argon atmosphere. The mixture was allowed to warm up to 0 °C and quenched with sat. NH_4Cl, extracted with Et_2O(x3) and concentrated in vacuo. Then NaOH (3.0 equiv) and Et_2O (0.20 M) were added and the reaction was stirred at rt for 16 h. Then water was added and the layers were separated, the aqueous layer extracted with Et_2O (x3), the combined ethereal extracts were washed with brine and dried ($MgSO_4$). Evaporation of the solvent and chromatographic purification of the crude product (silica, EtOAc/n-hexane 1:5) gave the product as an oil.

(*S*)-2-Allyloxirane (**S1c**)

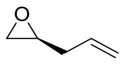

To a stirred solution of (*S*)-2-(chloromethyl)oxirane, **S1b** (7.9 mL, 100 mmol, 1 equiv.) and CuCN (1.8 g, 20.0 mmol, 0.20 equiv.) in dry THF (1 M), was added over 45 min a 1.0 M solution of vinylmagnesium bromide in THF (100 mL, 100 mmol, 1.0 equiv.) dropwise at -78 °C under argon atmosphere. The mixture was allowed to warm up to 0 °C and quenched with sat. NH_4Cl, extracted with Et_2O(x3) and concentrated in vacuo. KOH (6.2 g, 110 mmol, 1.1 equiv.) was added into the crude product and the reaction mixture was heated in an oil bath at 80 °C and distilled the titled compound **S1c** by downward distillation over two hours as a colourless oil (6.37 g, 76 mmol, 66%). The boiling point matched with that reported in literature (80 – 82 °C).

Rf = 0.59 (20 % EtOAc/cyclohexane).

^{1}**H NMR** (300 MHz, $CDCl_3$) δ 5.83 (ddt, *J* = 17.0, 10.2, 6.7 Hz, 1H), 5.23 – 5.05 (m, 2H), 3.00 (tdd, *J* = 5.4, 3.9, 2.7 Hz, 1H), 2.76 (dd, *J* = 5.0, 3.9 Hz, 1H), 2.51 (dd, *J* = 5.0, 2.7 Hz, 1H), 2.32 (m, 2H).

Spectroscopic data matched with those described in the literature.[1]

(*S*)-2-(Cyclopropylmethyl)oxirane (**S1e**)

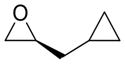

Prepared following the general procedure A using (*S*)-2-(chloromethyl)oxirane, **S1b** (1.7 mL, 22 mmol, 1.0 equiv.) and cyclopropylmagnesium bromide prepared in situ (22 mmol, 1 equiv.), the title compound **S1e** (0.65 g, 6.6 mmol) was obtained in 31% yield as a pale-yellow oil, after purification by column chromatography (silica, Et_2O/pentane 1:3).

Rf = 0.7 (20% EtOAc in cyclohexane).

^{1}**H NMR** (300 MHz, $CDCl_3$) δ 2.97 (tdd, *J* = 5.6, 4.0, 2.7 Hz, 1H), 2.72 (dd, *J* = 5.2, 4.0 Hz, 1H), 2.48 (dd, *J* = 5.1, 2.7 Hz, 1H), 1.60 – 1.29 (m, 2H), 0.86 – 0.66 (m, 1H), 0.55 – 0.35 (m, 2H), 0.15 – 0.03 (m, 2H).

^{13}**C NMR** (75 MHz, $CDCl_3$) δ 52.05, 46.73, 37.39, 7.22, 4.28, 3.88.

Spectra

(*S*)-2-Phenethyloxirane (**S1f**)

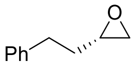

Prepared following the general procedure A using (*S*)-2-(chloromethyl)oxirane, **S1b** (2.5 mL, 32.4 mmol, 1.0 equiv.) and benzylmagnesium bromide (1.7 M, 29 mL, 48.6 mmol, 1.5 equiv.), the title compound **S1f** (3.20 g, 21.6 mmol) was obtained in 67% yield as a colorless oil, after purification by column chromatography (silica, Et_2O/pentane 1:5).

Spectroscopic data matched those of the literature.[2]

^{1}**H NMR (400 MHz, $CDCl_3$)** δ 7.30 (dd, J = 8.2, 6.9 Hz, 2H), 7.22 (d, J = 7.4 Hz, 3H), 2.96 (qt, J = 4.5, 2.8 Hz, 1H), 2.90-2.70 (m, 3H), 2.48 (dd, J = 5.0, 2.7 Hz, 1H), 1.96-1.77 (m, 2H).

(*S*)-2-(4-Chlorobenzyl)oxirane (**S1g**)

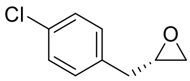

Prepared following the general procedure A using (*S*)-2-(chloromethyl)oxirane, **S1b** (1.7 mL, 21.6 mmol, 1.0 equiv.) and p-chlorophenylmagnesium bromide (0.90 M, 36 mL, 32.4 mmol, 1.5 equiv.), the title compound **S1g** (3.20 g, 19.0 mmol) was obtained in 88% yield as a colorless oil, after purification by column chromatography (silica, Et_2O/pentane 1:5).

Spectroscopic data matched those of the literature. [3]

^{1}**H NMR (400 MHz, $CDCl_3$)** δ 7.30-7.26 (m, 2H), 7.20-7.17 (m, 2H), 3.14-3.10 (m, 1H), 2.84 (d, J = 8.8, 2H), 2.80-2.78 (m, 1H), 2.51 (dd, J = 4.9, 2.6 Hz, 1H).

(*S*)-*tert*-Butyldimethyl(oxiran-2-ylmethoxy)silane (**S1h**)

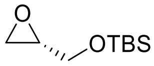

A mixture of (*R*)-glycidol, **S1d** (1.5 g, 20 mmol, 97% ee, 1 equiv.), *tert*-butyldimethylsilyl chloride (4.5 g, 30 mmol, 1.5 equiv.) and imidazole (2 mL, 30 mmol, 1.5 equiv.) in THF (40 mL) were stirred for 18 h at room temperature forming a white precipitate. The precipitate was filtered and washed with diethyl ether (40 mL). The combined filtrate was concentrated and purified by flash column chromatography (diethyl ether/petroleum ether, 1:10) to give the desired compound **S1h** (2.7 g, 14 mmol, 70%) as a clear oil. The synthesized epoxide will be used in the next step following procedure A.

^{1}H NMR (300 MHz, $CDCl_3$) δ 3.85 (dd, *J* = 12, 3 Hz, 1H), 3.66 (dd, *J* = 12, 5 Hz, 1H), 3.08 (ddd, *J* = 5, 4, 3 Hz, 1H), 2.76 (dd, *J* = 5, 4 Hz, 1H), 2.63 (dd, *J* = 5, 3 Hz, 1H), 0.90 (s, 9H), 0.08 (s, 3H), 0.07 (s, 3H).

Spectroscopic data are in agreement with those in the literature. [4]

2.4. Synthesis of cyclopropyl methyl boronic esters

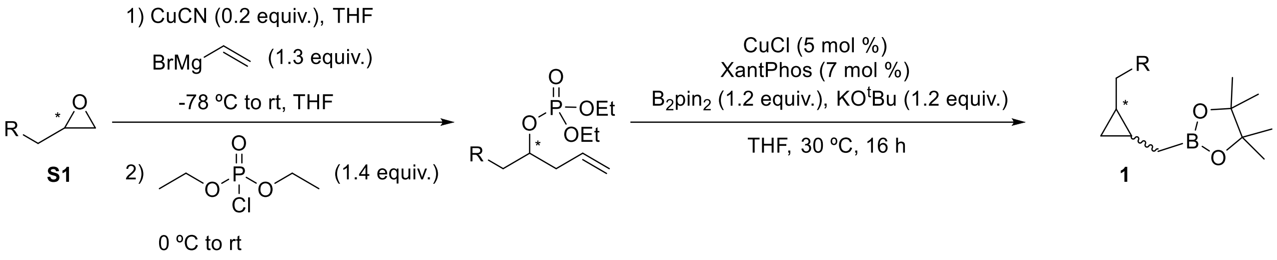

To a stirred solution of the previously synthesized epoxide (1 equiv.) and CuCN (0.20 equiv.) in dry THF (1 M), was added over 45 min a solution of vinylmagnesium bromide or alkylmagnesium halide in THF (1.3 equiv.) dropwise at -78 °C under argon atmosphere. The mixture was allowed to warm up to rt and was stirred upon completion. When the reaction was finished, diethyl phosphorochloridate (1.4 equiv.) was added at 0 °C and stirred 1 h at rt. Finally, the reaction was quenched with a saturated NH_4Cl solution. The layers were separated, the aqueous layer extracted with Et_2O (x3), the combined ethereal extracts were washed with brine and dried ($MgSO_4$). Then, the solvent was removed *in vacuo* to afford the corresponding phosphate that was directly used in the next step without further purification.

In an oven-dried round-bottom flask in the glovebox copper chloride (0.05 equiv.), XantPhos (0.07 equiv.), KO^{t}Bu (1.2 equiv.), B_2pin_2 (1.2 equiv.) were weighed, taken out of the glovebox and dissolved in THF (0.3 M). After 30 min at 30 °C the phosphate was added and it was allowed to react at 30 °C for 16 h. When the starting material was consumed, the reaction mixture was dissolved in Et_2O and filtered with a pad of silica-gel. Evaporation of the solvent and chromatographic purification of the crude product (silica, Et_2O/pentane) gave the cyclopropylmethyl boronic ester (cis/trans mixture) as an oil.

4,4,5,5-Tetramethyl-2-(((2*S*)-2-methylcyclopropyl)methyl)-1,3,2-dioxaborolane (**1a**)

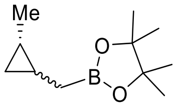

Prepared following the general procedure A using (*S*)-(-)-PropyleneOxide, **S1a** (1.0 g, 18 mmol, 1.0 equiv.) and vinyl magnesium bromide (1 M, 1.3 equiv.), the title compound **1a** (3.2 g, 16 mmol) was obtained as a 1.1:1.0 syn:*anti* mixture of isomers (measured by GC-MS) in 90% yield (3 steps) as a colorless oil, after purification by distillation.

Rf = 0.68 (20% EtOAc in cyclohexane).

^{1}H NMR-*mixture of isomers* (500 MHz, $CDCl_3$) δ 1.25 (s, 6H), 1.24 (s, 6H), 1.24 (s, 6H), 1.23 (s, 6H), 0.98 (d, *J* = 3 Hz, 3H), 0.97 (d, *J* = 3 Hz, 3H), 0.82 – 0.66 (m, 6H), 0.60 (m, 1H), 0.50 – 0.30 (m, 2H), 0.19 – 0.10 (m, 2H), -0.40 (m, 1H, **H**-CH-B cis).

^{13}C NMR-*mixture of isomers* (126 MHz, $CDCl_3$) δ 83.1 (2C), 83.0 (2C), 24.9 (2C), 24.9 (2C), 24.7 (4C), 19.1, 14.9, 14.4, 13.9, 13.4, 13.3, 10.7, 9.4. [note: the carbon attached to boron was not observed due to quadrupole broadening caused by the ^{11}B nucleus].

^{11}B NMR (160 MHz, $CDCl_3$) δ 33.6.

Spectra

HRMS (APCI): calculated for $C_{11}H_{21}BO_2$ $[M]^+$: 196.1635; found: 196.1740.

2-(((2*S*)-2-(Cyclopropylmethyl)cyclopropyl)methyl)-4,4,5,5-tetramethyl-1,3,2-dioxaborolane (**1b**)

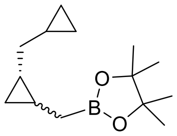

Prepared following the general procedure A using (*S*)-2-(cyclopropylmethyl)oxirane, **S1e** (0.65 g, 6.6 mmol, 1.0 equiv.) and vinyl magnesium bromide (1 M, 1.3 equiv.), the title compound **1b** (0.56 g, 2.4 mmol) was obtained as a mixture of isomers (1.5:1.0 measured by GC-MS) in 38% yield (3 steps) as a pale-yellow oil, after purification by flash column chromatography (Silica; 5–10% EtOAc in pentane).

Rf = 0.68 (20% EtOAc in cyclohexane).

^{1}H NMR-*mixture of isomers* (500 MHz, $CDCl_3$) δ 1.20 (s, 6H), 1.19 (s, 6H), 1.20 (s, 6H), 1.19 (s, 6H), 1.19 (s, 6H), 1.07 (m, 3H), 0.99 (m, 1H), 0.84 – 0.75 (m, 2H), 0.72 (m, 6H), 0.45 (m, 3H), 0.32 (m, 5H), 0.21 – 0.06 (m, 3H), -0.04 (m, 2H), -0.36 (dd, *J* = 5, 1.4 Hz, 1H, **H**-CH-B cis).

^{13}C NMR-*mixture of isomers* (126 MHz, $CDCl_3$) δ 83.0 (2C), 82.9 (2C), 39.3, 33.7, 24.9 (2C), 24.8 (4C), 24.8 (2C), 19.6, 15.4, 13.3, 12.8, 12.0, 11.0, 10.6, 10.4, 4.3, 4.2, 4.2, 4.0. [note: the carbon attached to boron was not observed due to quadrupole broadening caused by the ^{11}B nucleus].

^{11}B NMR (160 MHz, $CDCl_3$) δ 33.7. *Spectra*

HRMS (APCI): calculated for $C_{14}H_{25}BO_2$ $[M]^+$: 236.1948; found: 236.2018.

2-(((2*S*)-2-(Cyclobutylmethyl)cyclopropyl)methyl)-4,4,5,5-tetramethyl-1,3,2-dioxaborolane (**1c**)

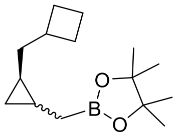

Prepared following the general procedure B using (*S*)-2-allyloxirane **S1c** (2.0 g, 7.8 mmol, 1.0 equiv.) and cyclobutylmagnesium bromide prepared from the corresponding bromide (1.5 equiv.), the title compound **1c** (1.01 g, 4.0 mmol) was obtained as a mixture of isomers (1.2:1.0 measured by GC-MS) in 51% yield (3 steps) as a transparent oil, after purification by flash column chromatography (Silica; 5–10% EtOAc in pentane).

Rf = 0.68 (20% EtOAc in cyclohexane).

^{1}H NMR-*mixture of isomers* (500 MHz, $CDCl_3$) δ 2.31 (sex, *J* = 8 Hz, 2H), 2.04 – 1.91 (m, 5H), 1.83 – 1.65 (m, 4H), 1.63 – 1.50 (m, 4H), 1.47 – 1.35 (m, 2H), 1.19 (s, 6H), 1.19 (s, 6H), 1.19 (s, 6H), 1.18 (s, 6H), 0.82 (m, 1H), 0.78 – 0.70 (m, 2H), 0.70 – 0.66 (m, 3H), 0.52 (m, 2H), 0.44 – 0.35 (m, 1H), 0.30 – 0.20 (m, 1H), 0.15 – 0.03 (m, 2H), -0.38 – -0.44 (m, 1H, **H**-CH-B cis).

^{13}C NMR-*mixture of isomers* (126 MHz, $CDCl_3$) δ 82.9, 82.9, 41.5, 36.6, 36.4, 35.6, 28.5, 28.5, 28.4, 28.4, 24.9 (4C), 24.8 (4C), 18.8, 18.7, 18.0, 13.6, 13.2, 12.8, 11.9, 10.1. [note: the carbon attached to boron was not observed due to quadrupole broadening caused by the ^{11}B nucleus].

^{11}B NMR (160 MHz, $CDCl_3$) δ 33.8.

Spectra

HRMS (APCI): calculated for $C_{15}H_{27}BO_2$ $[M]^+$: 250.2104; found: 250.2154.

4,4,5,5-Tetramethyl-2-(((2*S*)-2-phenethylcyclopropyl)methyl)-1,3,2-dioxaborolane (**1d**)

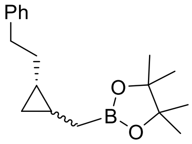

Prepared following the general procedure A using the previous synthesized (*S*)-2-phenethyloxirane **S1f** (3.2 g, 21.6 mmol, 1.0 equiv.) and vinyl magnesium bromide (1 M, 1.3 equiv.) the title compound **1d** (4.4 g, 15 mmol was obtained as a mixture of isomers (1.1:1.0 measured by GC-MS) in 71% yield (3 steps) as a colorless oil, after purification by flash column chromatography (Silica; 5–30% EtOAc in pentane).

Rf = 0.60 (20% EtOAc in cyclohexane).

^{1}H NMR-*mixture of isomers* (500 MHz, $CDCl_3$) δ7.28 (m, 1H), 7.27 (m, 2H), 7.25 (d, *J* = 2 Hz, 2H), 7.21 (m, 1H), 7.20 (m, 2H), 7.18 (m, 1H), 7.17 (m, 1H), 2.71 (m, 4H), 1.64 (ddt, *J* = 14, 9, 7 Hz, 1H), 1.55 (dtd, *J* = 14, 7, 4 Hz, 2H), 1.50 – 1.43 (m, 1H), 1.27 (s, 12H), 1.26 (s, 12H), 0.92 – 0.82 (m, 2H), 0.79 (d, *J* = 6.9 Hz, 2H), 0.79 – 0.68 (m, 2H), 0.64 (td, *J* = 8, 4 Hz, 1H), 0.59 – 0.51 (m, 1H), 0.51 – 0.41 (m, 1H), 0.28 – 0.17 (m, 2H), -0.29 (dd, *J* = 5, 5 Hz, 1H, **H**-CH-B cis).

^{13}C NMR-*mixture of isomers* (126 MHz, $CDCl_3$) δ 143.0, 143.0, 128.6 (2C), 128.6 (2C), 128.3 (4C), 125.7, 125.6, 83.1 (2C), 83.1 (2C), 36.7, 36.5, 36.1, 31.0, 25.0 (2C), 25.0 (4C), 25.0 (2C), 19.9, 15.6, 14.0, 13.3, 12.2, 10.9.

[note: the carbon attached to boron was not observed due to quadrupole broadening caused by the ^{11}B nucleus].

^{11}B NMR (160 MHz, $CDCl_3$) δ 33.8.

Spectra

HRMS (APCI): calculated for $C_{18}H_{27}BO_2$ $[M]^+$: 286.2104; found: 286.2150.

tert-Butyldimethyl(((1*R*)-2-((4,4,5,5-tetramethyl-1,3,2-dioxaborolan-2-yl)methyl)cyclopropyl)methoxy)silane (**1e**)

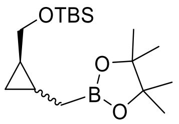

Prepared following the general procedure A using (*R*)-*tert*-butyldimethyl(oxiran-2-ylmethoxy)silane **S1h** (4.0 g, 21.2 mmol, 1.0 equiv.) and vinyl magnesium bromide (1 M, 1.3 equiv.) the title compound **1e** (3.9 g, 11.9 mmol) was obtained as a mixture of isomers (1.0:1.0 measured by GC-MS) in 58% yield (3 steps) as a colorless oil, after purification by flash column chromatography (Silica; 10–50% EtOAc in pentane).

Rf = 0.4 (20% EtOAc in cyclohexane).

^1H NMR-*mixture of isomers* (500 MHz, $CDCl_3$) δ 3.62 – 3.47 (m, 3H), 3.33 (dd, *J* = 11, 7 Hz, 1H), 1.20 (s, 12H), 1.19 (s, 12H), 0.95 – 0.87 (m, 3H), 0.84 (s, 9H), 0.83 (s, 9H), 0.81 – 0.77 (m, 1H), 0.76 – 0.66 (m, 2H), 0.66 – 0.55 (m, 3H), 0.31 (dt, *J* = 9, 5 Hz, 1H), 0.19 (m, 1H), 0.02 – -0.05 (m, 12H), -0.16 (dd, *J* = 5, 5 Hz, 1H, **H**-CH-B cis).

^{13}C NMR-*mixture of isomers* (126 MHz, $CDCl_3$) δ 83.0 (2C), 83.0 (2C), 67.1, 63.6, 26.1 (4C), 26.1 (4C), 24.9 (6C), 22.3, 18.5, 18.5, 18.0, 11.8, 11.4, 10.9, 10.8, -5.0, -5.0 (2C), -5.07. [note: the carbon attached to boron was not observed due to quadrupole broadening caused by the ^{11}B nucleus].

^{11}B NMR (160 MHz, $CDCl_3$) δ 33.7.

Spectra

HRMS (APCI): calculated for $C_{17}H_{35}BO_3Si$ $[M]^+$: 326.2448; found: 326.2555.

4,4,5,5-Tetramethyl-2-(((2*S*)-2-(3-methylbut-2-en-1-yl)cyclopropyl)methyl)-1,3,2-dioxaborolane (**1f**)

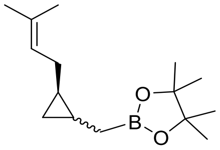

Prepared following the general procedure B using (*S*)-2-allyloxirane **S1c** (2.0 g, 7.9 mmol, 1.0 equiv.) and 2-Methyl-1-propenyl magnesium chloride (1 M, 1.3 equiv.) the title compound **1f** (181 mg, 1.6 mmol) was obtained as a mixture of isomers (1.3:1.0 measured by GC-MS) in 20% yield (3 steps) as a transparent oil, after purification by flash column chromatography (Silica; 5–20% EtOAc in pentane).

Rf = 0.74 (20% EtOAc in cyclohexane).

^{1}H NMR-*mixture of isomers* (500 MHz, $CDCl_3$) δ 5.22 (m, 2H), 2.00 – 1.81 (m, 5H), 1.69 (d, *J* = 1 Hz, 3H), 1.68 (d, *J* = 1 Hz, 3H), 1.59 (d, *J* = 1 Hz, 3H), 1.57 (d, *J* = 1 Hz, 3H), 1.26 (s, 12H), 1.25 (s, 12H), 0.91 – 0.65 (m, 8H), 0.62 (m, 1H), 0.56 – 0.47 (m, 1H), 0.42 (m, 1H), 0.23 (dt, *J* = 8, 5 Hz, 1H), 0.15 (dt, *J* = 8, 5 Hz, 1H), -0.29 (dd, *J* = 5, 5 Hz, 1H, **H**-CH-B cis).

^{13}C NMR-*mixture of isomers* (126 MHz, $CDCl_3$) δ 131.1, 131.0, 124.8, 124.2, 83.1 (2C), 83.1 (2C), 32.6, 27.3, 25.9, 25.9, 25.0 (2C), 25.0 (2C), 25.0 (4C), 20.1, 17.9, 17.9, 16.1, 13.4, 12.9, 12.1, 10.7. [note: the carbon attached to boron was not observed due to quadrupole broadening caused by the ^{11}B nucleus].

^{11}B NMR (160 MHz, $CDCl_3$) δ 33.8.

Spectra

HRMS (APCI): calculated for $C_{15}H_{27}BO_2$ $[M]^+$: 250.2104; found: 250.2175.

2-(((2*R*)-2-(4-Chlorobenzyl)cyclopropyl)methyl)-4,4,5,5-tetramethyl-1,3,2-dioxaborolane (**1g**)

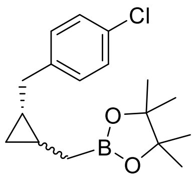

Prepared following the general procedure A using (*S*)-2-(4-chlorobenzyl)oxirane **S1g** (2.27 g, 13.5 mmol, 1.0 equiv.) and vinyl magnesium bromide (1 M, 1.3 equiv.), the title compound **1g** (2.1 g, 6.8 mmol) was obtained as a mixture of isomers (2.0:1.0 measured by GC-MS) in 50% yield (3 steps) as a pale-yellow oil, after purification by flash column chromatography (Silica; 5–20% EtOAc in pentane).

Rf = 0.57 (20% EtOAc in cyclohexane).

^{1}H NMR-*mixture of isomers* (500 MHz, $CDCl_3$) δ 7.31 – 7.27 (m, 2H), 7.27 – 7.24 (m, 3H), 7.23 – 7.20 (m, 3H), 2.60 – 2.50 (m, 4H), 1.26 (s, 24H), 0.87 (d, *J* = 6 Hz, 1H), 0.84 (d, *J* = 6 Hz, 2H), 0.82 – 0.78 (m, 1H), 0.73 (m, 3H), 0.41 (m, 2H), 0.33 (m, 2H).

^{13}C NMR-*mixture of isomers* (126 MHz, $CDCl_3$) δ 141.3, 140.9, 131.5, 131.4, 129.8 (2C), 129.8 (2C), 128.4 (2C), 128.4 (2C), 83.2 (2C), 83.1 (2C), 39.56, 33.9, 25.0 (2C), 24.9 (4C), 24.9 (2C), 20.7, 16.6, 14.1, 13.3, 12.5, 11.2. [note: the carbon attached to boron was not observed due to quadrupole broadening caused by the 11B nucleus].

^{11}B NMR (160 MHz, $CDCl_3$) δ 33.5.

Spectra

HRMS (APCI): calculated for $C_{17}H_{24}BClO_2$ $[M]^+$: 306.1558; found: 306.1989.

2.5. tert-butyl 3-((1*S*)-2-((4,4,5,5-tetramethyl-1,3,2-dioxaborolan-2-yl)methyl)cyclopropyl)azetidine-1-carboxylate (**1h**)

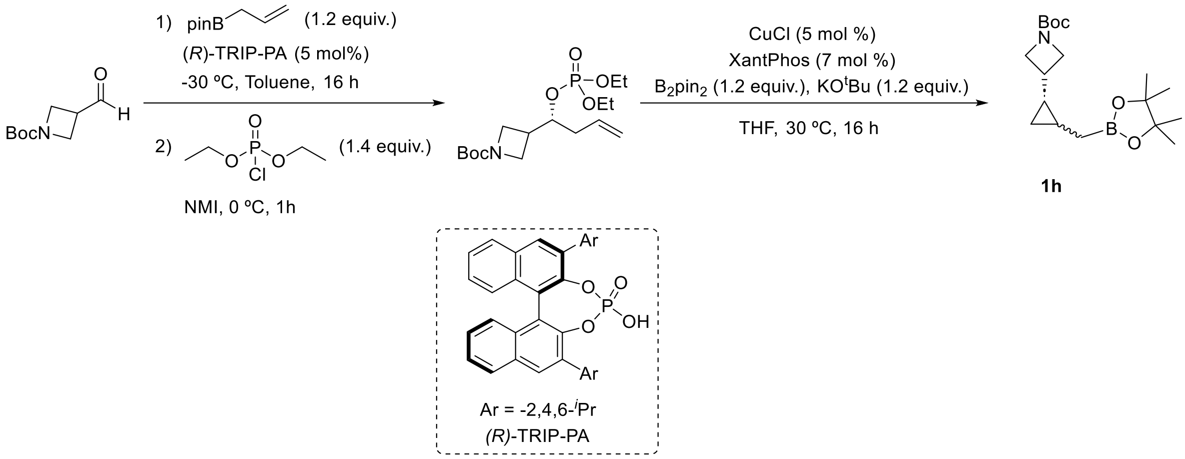

1h

The enantioselective allylation was performed using Antilla's procedure [5]: (*R*)-TRIP-PA (94.1 mg, 0.125 mmol, 5 mol%) was weighted in a flame-dried reaction vial and vacuum-argon cycles were done (x3). Under argon atmosphere, 1.5 mL of anhydrous toluene and *tert*-butyl-3-formylazetidine-1-carboxylate (0.39 mL, 2.5 mmol, 1.0 equiv.) were added. The reaction mixture was then cooled to -30 °C followed by dropwise the addition of allylboronic acid pinacol ester (0.56 mL, 3.0 mmol, 1.2 equiv.). The mixture was stirred overnight at this temperature. Then, the solvent was evaporated, and the reaction crude was used in the next step without purification. The enantiomeric ratio was determined to be 92:8 (see below for details).

Under argon atmosphere *tert*-butyl (*R*)-3-(1-hydroxybut-3-en-1-yl)azetidine-1-carboxylate (568 mg, 2.5 mol, 1.0 equiv.) was dissolved in dichloromethane (2.5mL) and cooled to 0 °C. *N*-Methylimidazole (0.79 mL, 10.0 mmol, 4.0 equiv.) was added by syringe and the solution was stirred for 20 min. Diethyl chlorophosphate (0.72 mL, 5.0 mmol, 2.0 equiv.) was then added by syringe and the solution was stirred for 30 minutes at 0 °C. After warming to room temperature, saturated NH_4Cl (20 mL) was added, and the aqueous phase was extracted with CH_2Cl_2. The organic extracts were washed with brine, dried with Na_2SO_4, filtered, and concentrated in vacuo to give the product which was used in the next step without further purification.

In an oven-dried round-bottom flask in the glovebox copper chloride (12 mg, 0.13 mmol, 0.05 equiv.), XantPhos (0.10 g, 0.18 mmol, 0.07 equiv.), KO^{t}Bu (0.34 g, 3.0 mmol, 1.2 equiv.), B_2pin_2 (0.76 g, 3.0 mmol, 1.2 equiv.) were weighed, taken out of the glovebox and dissolved in THF (0.3 M). After 30 min at 30 °C the *tert*-butyl (*R*)-3-(1-((diethoxyphosphoryl)oxy)but-3-en-1-yl)azetidine-1-carboxylate was added and it was allowed to react at 30 °C for 16 h. When the starting material was consumed, the reaction mixture was dissolved in Et_2O and filtered with a pad of silica-gel.

The title compound (*S*)-**1h** (0.208 g, 0.6 mmol) was obtained in 25% yield (3 steps) as a colorless oil, after purification by flash column chromatography (Silica; 5–20% EtOAc in pentane).

Rf = 0.44 (4:1 cyclohexane/EtOAc).

^{1}H NMR-*mixture of isomers* (500 MHz, $CDCl_3$) δ 4.03 (td, *J* = 8, 4 Hz, 2H), 3.93 (td, *J* = 9, 3 Hz, 3H), 3.75 – 3.68 (m, 1H), 3.65 (m, 2H), 2.23 – 2.08 (m, 2H), 1.43 (s, 9H), 1.43 (s, 9H), 1.25 (s, 24H), 1.00 (m, 1H), 0.95 – 0.84 (m, 3H), 0.78 (dd, *J* = 16, 6 Hz, 1H), 0.69 (dp, *J* = 12, 4 Hz, 3H), 0.33 – 0.22 (m, 3H), -0.21 (d, *J* = 5 Hz, 1H, **H**-CH-B cis). **^{13}C NMR-*mixture of isomers*** (126 MHz, $CDCl_3$) δ 156.6 (2C), 83.3 (2C), 83.2 (2C), 79.2 (2C), 79.1 (2C), 32.6 (2C), 28.6 (6C), 25.0 (2C), 25.0 (2C), 25.0 (2C), 25.0 (2C), 24.7 (2C), 23.0 (2C), 20.4 (2C), 12.2 (2C), 11.8 (2C). [note: the carbon attached to boron was not observed due to quadrupole broadening caused by the 11B nucleus]. **^{11}B NMR** (160 MHz, $CDCl_3$) δ 33.6. *Spectra*

HRMS (APCI): calculated for $C_{18}H_{32}BNO_4$ $[M]^+$: 337.2424; found: 337.2508.

To determine the enantioselectivity of the allylation reaction *tert*-butyl (*R*)-3-(1-((diphenoxyphosphoryl)oxy)but-3-en-1-yl)azetidine-1-carboxylate (**S8**) was synthesized and the two enantiomers were separated using Chiral HPLC.

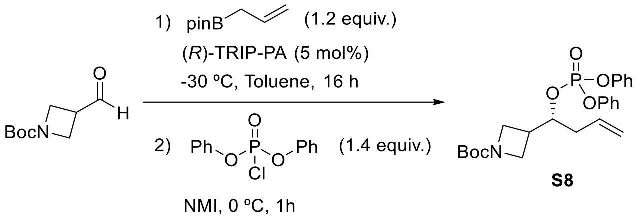

The enantioselective allylation was performed using Antilla's procedure [5]: (*R*)-TRIP-PA (18.8 mg, 0.025 mmol, 5 mol%) was weighted in a flame-dried reaction vial and vacuum-argon cycles were done (x3). Under argon atmosphere, 5.0 mL of anhydrous toluene and *tert*-butyl-3-formylazetidine-1-carboxylate (92.6 mg, 0.5 mmol, 1.0 equiv.) were added. The reaction mixture was then cooled to -30 °C followed by dropwise the addition of allylboronic acid pinacol ester (0.11 mL, 0.6 mmol, 1.2 equiv.). The mixture was stirred overnight at this temperature,then the solvent was evaporated, and the reaction crude was used in the next step without purification.

Under argon atmosphere *tert*-butyl (*R*)-3-(1-hydroxybut-3-en-1-yl)azetidine-1-carboxylate (114 mg, 0.5 mol, 1.0 equiv.) was dissolved in dichloromethane (0.5mL) and cooled to 0 °C. N-Methylimidazole (0.16 mL, 2.0 mmol, 4.0 equiv.) was added by syringe and the solution was stirred for 20 min. Diphenyl chloridophosphate (0.20 mL, 1.0 mmol, 2.0 equiv.) was then added by syringe and the solution was stirred for 30 minutes at 0 °C. After warming to room temperature, saturated NH_4Cl (20 mL) was added, and the aqueous phase was extracted with CH_2Cl_2. The organic extracts were washed with brine, dried with Na_2SO_4, filtered, and concentrated in vacuo to give the product (188 mg, 0.4 mmol) in 82% yield which was obtained after purification by column chromatography (5-40% EtOAc in cyclohexane).

[α] ^{25} $_D$ = + 35.9 (c = 1.0, $CHCl_3$).

^{1}H NMR (300 MHz, $CDCl_3$) δ 7.32 (t, J = 7.7 Hz, 4H), 7.25 – 7.12 (m, 6H), 5.78 – 5.58 (m, 1H), 5.07 (s, 1H), 5.05 – 4.98 (m, 1H), 4.78 (ddd, J = 13.2, 7.5, 5.8 Hz, 1H), 3.95 – 3.74 (m, 3H), 3.68 (dd, J = 8.9, 6.1 Hz, 1H), 2.89 – 2.73 (m, 1H), 2.40 (dt, J = 9.3, 4.5 Hz, 2H), 1.40 (s, 9H).

^{13}C NMR (76 MHz, $CDCl_3$) δ 150.6 (dd, J = 7.2, 5.2 Hz), 129.8 (d, J = 2.8 Hz), 125.5 (dd, J = 6.3, 1.7 Hz), 120.1 (dd, J = 5.1, 3.2 Hz), 81.0 (d, J = 6.3 Hz), 37.9 (d, J = 2.8 Hz), 32.6 (d, J = 6.6 Hz).

^{31}P NMR (122 MHz, $CDCl_3$) δ -12.23. *Spectra*

The enantiomeric ratio (92:8) was determined by chiral HPLC using Chiralpak-IBN column [hexane/i-PrOH (90:10)], 1 mL/min, τ_{major} = 4.6 min, τ_{minor} = 5.1 min.

HPLC chromatogram: Racemic phosphate

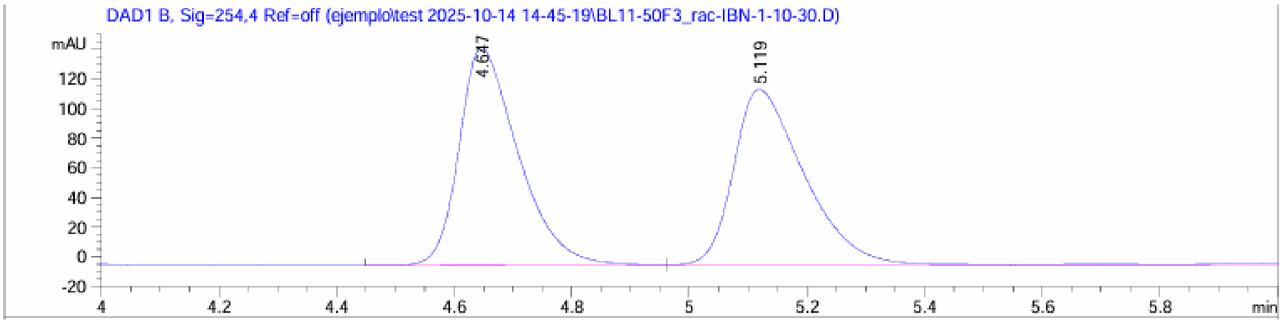

Peak #	RetTime [min]	Type	Width [min]	Area [mAU*s]	Height [mAU]	Area %
1	4.647	BB	0.1043	1022.18134	146.59753	50.4408
2	5.119	BV R	0.1213	1004.31409	118.36977	49.5592

HPLC chromatogram: Enantioselective phosphate

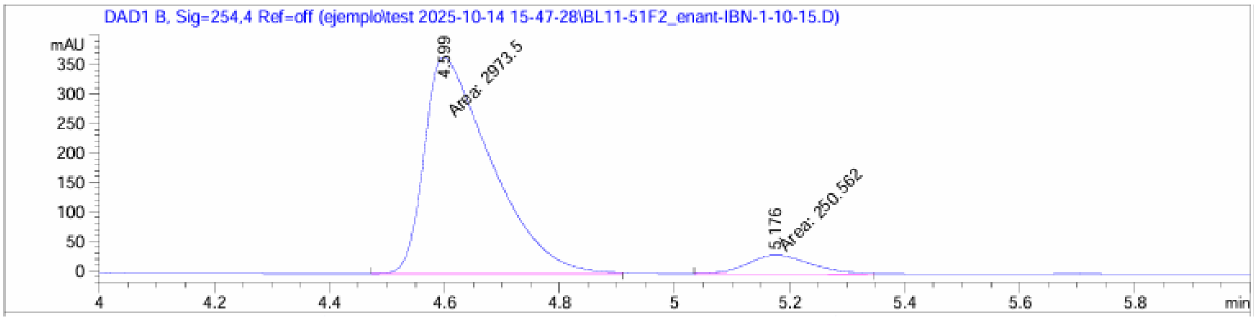

Peak #	RetTime [min]	Type	Width [min]	Area [mAU*s]	Height [mAU]	Area %
1	4.599	MF	0.1346	2973.50024	368.24005	92.2284
2	5.176	FM	0.1294	250.56194	32.26291	7.7716

2.6. Large scale synthesis of 4,4,5,5-tetramethyl-2-(((2*S*)-2-methylcyclopropyl) methyl)-1,3,2-dioxaborolane (**1a**)

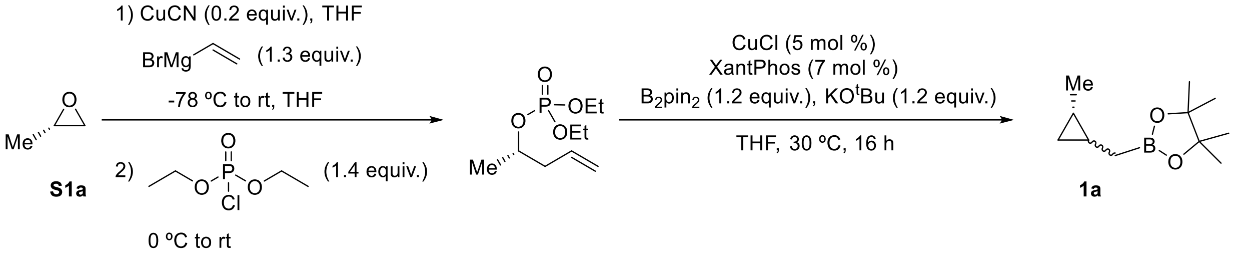

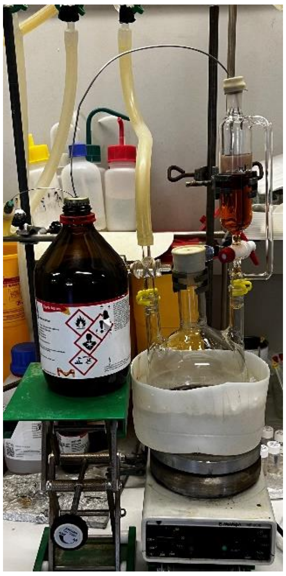

To a 1L three-necked round-bottom flask equipped with a dropping funnel was added CuCN (2.16 g, 24.1 mmol, 0.2 equiv.) and vacuum-argon cycles were done. Under argon atmosphere, 200 mL of dry THF was added and a 1 M solution of vinylmagnesium bromide in THF (181 mL, 181 mmol, 1.5 equiv.) was added dropwise at - 10 °C through the dropping funnel.* Then, (*S*)-(-)-PropyleneOxide **S1a** (8.4 mL, 120 mmol, 1.0 equiv.) in THF (50 mL) was added dropwise over 1 h and the mixture was allowed to warm up to rt and was stirred for 1 hour. Upon completion, diethyl phosphorochloridate (22.5 mL, 157 mmol, 1.3 equiv.) was added dropwise at 0 °C and stirred 1 h at rt. Finally, the reaction was quenched with a saturated NH_4Cl solution. The layers were separated, the aqueous layer extracted with Et_2O (x3), the combined ethereal extracts were washed with brine and dried ($MgSO_4$). Then, the solvent was removed *in vacuo* to afford the corresponding phosphate (25.6 g, 115 mmol, 95%) that was directly used in the next step without further purification.

In an oven-dried round-bottom flask copper chloride (0.57 g, 5.8 mmol,0.05 equiv.), XantPhos (4.67 g, 8.1 mmol, 0.07 equiv.), KO^{t}Bu (15.5 g, 138 mmol, 1.2 equiv.), B_2pin_2 (35.1 g, 138 mmol, 1.2 equiv.) were weighed, taken out of the glovebox and dissolved in THF (0.3 M). After 1 h (to ensure complexation) at 30 °C the phosphate (25.6 g, 115 mmol, 1 equiv.) was added and it was allowed to react at 30 °C for 16 h. When the reaction had finished, the reaction was dissolved in Et_2O and filtered through a silica pad. Evaporation of the solvent and distillation (50 °C, 1 mbar) gave the product **1a** as an oil in a cis/trans mixture (13.6 g, 69 mmol, 60%, 3 steps).

> **Inverse addition was used when we scale up, as the main by-product that may arise from this reaction is the formation of the halohydrine. At large scale the formation of this by-product is more difficult to control. With a slow inverse addition we guaranteed that the vinyl Grignard is in high excess and suppressed the formation of halohydrine.* [6]

3. Negishi cross-coupling reaction with aryl halides

3.1. Optimization of Negishi reaction conditions with aryl halides

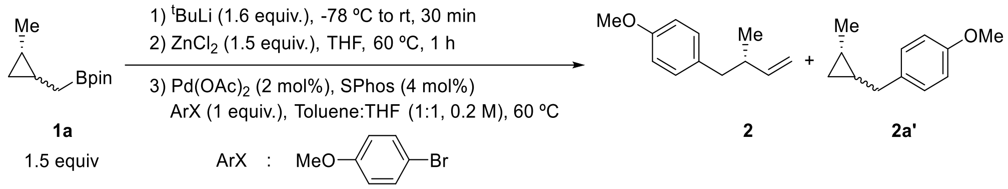

Entry	Deviation from standard conditions	Yield 2a (%) [a]	Ratio 2a:2a' [a]
**1**	-	92%	100:0
**2**	No Zinc source	60%	82:18
**3**	Addition of 2 and 3 at once	48%	82:18
**4**	secBuLi, No Zinc source	15%	58:42
**5**	1 equiv. 1a	60%	100:0
**6**	$Zn(OAc)_2$	87%	100:0

[a] Yield calculated by ^{1}H NMR using an internal standard. [b] Ratio calculated by GCMS

3.2. General procedure A for the Negishi cross-coupling reaction with aryl halides

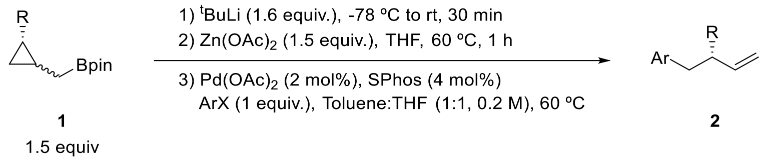

In an oven-dried Schlenk flask, cyclopropyl methyl boronic ester **1** (0.3 mmol, 1.5 equiv.) was weighed and vacuum-argon cycles were done (x3). Under argon atmosphere, 0.2 mL of anhydrous THF was added, the solution was cooled to -78 °C (dry ice/acetone bath) and *tert*-butyllithium (1.6 equiv.) was added dropwise with a syringe. Then, the reaction mixture was stirred while warming to room temperature for 30 min. After this time, zinc acetate (1.6 equiv.) was added as a solid to the mixture under argon and the reaction was stirred at 60 °C (oil bath) for 1 hour. Meanwhile, palladium acetate (0.02 equiv.), SPhos (0.04 equiv.) and THF (0.3 mL) were added to an oven-dried vial under argon atmosphere. The mixture was allowed to stir at room temperature for 1 hour. Then, the aryl bromide (0.2 mmol, 1 equiv.) was added to the Schlenk flask containing the organozinc reagent, followed by preformed palladium complex and 0.5 mL of toluene. The reaction was allowed to stir at 60 °C for 16 hours. Upon completion, the reaction mixture was passed through a short pad of silica gel, washed with EtOAc and concentrated under reduced pressure. The resulting crude oil was purified by silica gel column chromatography (silica deactivated with 5% Et_3N in pentane, pentane/EtOAc, stained with $KMnO_4$) to furnish the desired product **2**. Deactivation of the silica gel was necessary to avoid isomerization of the double bond in the products.

3.3. General procedure B for the Negishi cross-coupling reaction with aryl halides

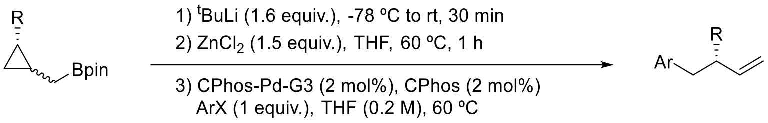

1
1.5 equiv
2

In an oven-dried vial, cyclopropyl methyl boronic ester **1** (0.3 mmol, 1.5 equiv.) was weighed and vacuum-argon cycles were done (x3). Under argon atmosphere, 0.2 mL of anhydrous THF was added, the solution was cooled to -78 °C (dry ice/acetone bath) and *tert*-butyllithium (1.6 equiv.) was added dropwise with a syringe. Then, the reaction mixture was stirred while warming to room temperature for 30 min. After this time, the reaction vial was transferred into the glovebox and zinc chloride (0.3 mmol, 1.5 equiv.) was added as a solid. The vial was brought out of the glovebox and the reaction was stirred at 60 °C (oil bath) for 1 hour. In a separate vial, CPhos-Pd-G3 and CPhos (2 mol%) were dissolved in 0.2 mL THF. Then, the aryl halide was added directly to the reaction vial, the generated alkyl zinc solution was added and the reaction was allowed to stir at 60 °C for 16 hours. Upon completion, the reaction mixture was passed through a short pad of silica gel, washed with EtOAc and concentrated under reduced pressure. The resulting crude oil was purified by silica gel column chromatography (pentane/EtOAc) to furnish the desired product **2**. Deactivation of the silica gel was necessary to avoid isomerization of the double bond in the products.

3.4. Large scale procedure for the Negishi cross-coupling reaction

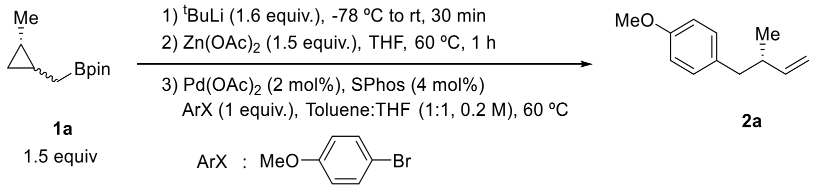

1a
1.5 equiv
2a

In an oven-dried round-bottom flask, 4,4,5,5-tetramethyl-2-(((2*R*)-2-methylcyclopropyl)methyl)-1,3,2-dioxaborolane **1a** (1.5 g, 7.5 mmol, 1.5 equiv.) was weighed and vacuum-argon cycles were done (x3). Under argon atmosphere, 5.2 mL of anhydrous THF was added, the solution was cooled to -78 °C (dry ice/acetone bath) and *tert*-butyllithium (4.7 mL, 8.0 mmol, 1.7 M, 1.6 equiv.) was added dropwise with a syringe. Then, the reaction mixture was stirred while warming to room temperature for 30 min. After this time, zinc acetate (1.5 g, 8.0 mmol, 1.6 equiv.) was added as a solid to the mixture under argon and the reaction was stirred at 60 °C (oil bath) for 1 hour. Meanwhile, palladium acetate (22 mg, 0.1 mmol, 0.02 equiv.), SPhos (82 mg, 0.2 mmol, 0.04 equiv.) and THF (7.8 mL) were added to an oven-dried vial under argon atmosphere. The mixture was allowed to stir at room temperature for 1 hour. Then, 4-bromoanisole (0.63 mL, 5.0 mmol, 1.0 equiv.) was added to the round-bottom flask containing the organozinc reagent, followed by preformed palladium complex and 13 mL of toluene. The reaction was allowed to stir at 60 °C for 16 hours. Upon completion, the reaction mixture was passed through a short pad of silica gel, washed with EtOAc and concentrated under

reduced pressure. The resulting crude oil was purified by silica gel column chromatography chromatography (5% triethylamine in pentane, stained with $KMnO_4$) to furnish the desired product **2a** (0.65 g, 3.7 mmol, 73%). Deactivation of the silica gel was necessary to avoid isomerization of the double bond in the products

3.5. Characterization data of Negishi aryl-homoallylation products

(*S*)-1-Methoxy-4-(2-methylbut-3-en-1-yl)benzene (2a)

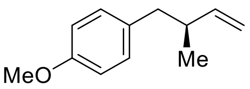

From cyclopropyl methyl boronic ester **1a** (58.8 mg, 0.30 mmol) and 1-bromo-4-methoxybenzene (25 µL, 0.20 mmol), following the general procedure A described above, compound **2a** was obtained. Purification by flash column chromatography (pentane) afforded **2a** (30.7 mg, 0.174 mmol, 87%) as a colorless oil.

[α] ^{25} $_{D}$ = - 32.5 (*c* = 1.0, $CHCl_3$).

Rf = 0.55 (10 % EtOAc/cyclohexane).

^{1}H NMR (500 MHz, $CDCl_3$): δ 7.06 (d, *J* = 8.7 Hz, 1H), 6.82 (d, *J* = 8.7 Hz, 2H), 5.79 (ddd, *J* = 17.2, 10.4, 6.8, 1H), 4.98-4.88 (m, 3H), 3.79 (s, 3H), 2.63 (dd, *J* = 13.3, 6.6 Hz, 1H), 2.51 – 2.35 (m, 2H), 0.98 (d, *J* = 6.6 Hz, 3H).

^{13}C NMR (126 MHz, $CDCl_3$): δ 157.9, 144.2, 133.0, 130.2 (2C), 113.7 (2C), 112.8, 55.4, 42.5, 39.6, 19.4.

Spectra

HRMS (APCI): calculated for $C_{12}H_{16}O$ $[M]^+$: 176.1201; found: 176.1189.

(*R*)-1-(2-(Cyclobutylmethyl)but-3-en-1-yl)-4-methoxybenzene (2b)

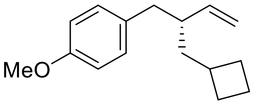

From cyclopropyl methyl boronic ester **1c** (75.1 mg, 0.30 mmol) and 1-bromo-4-methoxybenzene (25 µL, 0.20 mmol), following the general procedure A described above, compound **2b** was obtained. Purification by flash column chromatography (pentane) afforded **2b** (42.8 mg, 0.186 mmol, 93%) as a colorless oil.

[α] ^{25} $_{D}$ = - 45.5 (*c* = 1.0, $CHCl_3$).

Rf = 0.62 (10 % EtOAc/cyclohexane).

^{1}H NMR (300 MHz, $CDCl_3$) δ 7.05 (d, *J* = 8.2 Hz, 2H), 6.82 (d, *J* = 8.2 Hz, 2H), 5.58 (ddd, *J* = 17.1, 10.3, 8.6 Hz, 1H), 4.90 (ddd, *J* = 10.3, 2.0, 0.7 Hz, 1H), 4.84 (ddd, *J* = 17.1, 2.0, 0.9 Hz, 1H), 3.79 (s, 3H), 2.63 – 2.46 (m, 2H), 2.45 – 2.28 (m, 1H), 2.27 – 2.13 (m, 1H), 2.08 – 1.93 (m, 2H), 1.88 – 1.71 (m, 2H), 1.64 – 1.48 (m, 2H), 1.44 – 1.34 (m, 1H), 1.32 – 1.22 (m, 1H).

^{13}C NMR (76 MHz, $CDCl_3$) δ 157.8, 142.8, 132.9, 130.2 (2C), 114.2, 113.6 (2C), 55.3, 44.2, 41.9, 41.2, 34.3, 29.1, 28.7, 18.7.

[Spectra](#)

HRMS (APCI): calculated for $C_{16}H_{22}O$ [M]$^+$: 230.1671; found: 230.1665.

(S)-1-Methoxy-4-(2-phenethylbut-3-en-1-yl)benzene (**2c**)

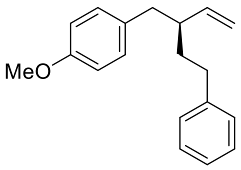

From cyclopropyl methyl boronic ester **1d** (85.9 mg, 0.30 mmol) and 1-bromo-4-methoxybenzene (25 µL, 0.20 mmol), following the general procedure A described above, compound **2c** was obtained. Purification by flash column chromatography (pentane) afforded **2c** (51.2 mg, 0.192 mmol, 96%) as a colorless oil.

[α] ^{25} $_D$ = - 17.1 (c = 1.0, $CHCl_3$).

Rf = 0.52 (10 % EtOAc/cyclohexane).

^{1}H NMR (500 MHz, $CDCl_3$) δ 7.23 (t, J = 7.5 Hz, 2H), 7.20 – 7.13 (m, 2H), 7.11 (d, J = 7.7 Hz, 2H), 7.01 (d, J = 8.5 Hz, 2H), 6.78 (d, J = 8.6 Hz, 1H), 5.63 (ddd, J = 17.1, 10.3, 8.5 Hz, 1H), 4.99 (dd, J = 10.1, 2.0 Hz, 1H), 4.91 (dd, J = 17.0, 2.0 Hz, 1H), 3.75 (s, 3H), 2.83 – 2.53 (m, 3H), 2.49 (m, 1H), 2.28 (m, 1H), 1.73 (m, 1H), 1.54 (dtd, J = 14.1, 9.9, 5.1 Hz, 1H).

^{13}C NMR (126 MHz, $CDCl_3$) δ 157.9, 142.7, 142.3, 132.5, 130.3 (2C), 128.5 (2C), 128.4 (2C), 125.8, 115.2, 113.6 (2C), 55.3, 45.5, 41.0, 35.8, 33.6.

[Spectra](#)

HRMS (APCI): calculated for $C_{19}H_{22}O$ [M]$^+$: 266.1670; found: 266.1665.

(R)-1-Methoxy-4-(5-methyl-2-vinylhex-4-en-1-yl)benzene (**2d**)

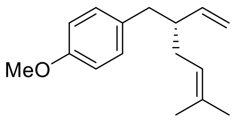

From cyclopropyl methyl boronic ester **1f** (75.1 mg, 0.30 mmol) and 5-bromo-2H-1,3-benzodioxole (24 µL, 0.20 mmol), following the general procedure A described above, compound **2d** was obtained. Purification by flash column chromatography (pentane) afforded **2d** (34.4 mg, 0.15 mmol, 75%) as a colorless oil.

[α] ^{25} $_D$ = - 34.0 (c = 1.0, $CHCl_3$).

Rf = 0.59 (10 % EtOAc/cyclohexane).

^{1}H NMR (300 MHz, $CDCl_3$) δ 7.07 (d, J = 8.6 Hz, 2H), 6.82 (d, J = 8.6 Hz, 2H), 5.68 (ddd, J = 17.1, 10.4, 7.9 Hz, 1H), 5.22 – 5.09 (m, 1H), 5.02 – 4.83 (m, 2H), 3.79 (s, 3H), 2.72 – 2.48 (m, 2H), 2.32 (m, 1H), 2.21 – 1.91 (m, 2H), 1.71 (s, 3H), 1.58 (s, 3H).

^{13}C NMR (76 MHz, $CDCl_3$) δ 157.8, 142.5, 132.9, 132.6, 130.3 (2C), 122.6, 114.3, 113.6 (2C), 55.3, 46.0, 40.1, 32.7, 25.9, 18.1, 1.8.

Spectra

HRMS (APCI): calculated for $C_{16}H_{22}O$ $[M]^+$: 230.1671; found: 230.1666.

(*S*)-4-(2-Methylbut-3-en-1-yl)-1,1'-biphenyl (2e)

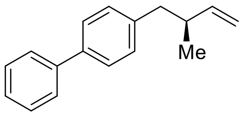

From cyclopropyl methyl boronic ester **1a** (58.8 mg, 0.30 mmol) and 1-bromo-4-methoxybenzene (46.6 mg, 0.20 mmol), following the general procedure A described above, compound **2e** was obtained. Purification by flash column chromatography (pentane) afforded **2e** (37.1 mg, 0.17 mmol, 83%) as a colorless oil.

[α] ^{25} $_D$ = + 10.0 (*c* = 1.0, $CHCl_3$).

Rf = 0.81 (10 % EtOAc/cyclohexane).

^{1}H NMR (300 MHz, $CDCl_3$) δ 7.55 – 7.48 (m, 2H), 7.43 (d, *J* = 8.4 Hz, 1H), 7.38 – 7.29 (m, 2H), 7.29 – 7.19 (m, 1H), 7.14 (d, *J* = 8.4 Hz, 1H), 5.75 (ddd, *J* = 17.2, 10.4, 6.8 Hz, 1H), 4.99 – 4.81 (m, 2H), 2.65 (dd, *J* = 13.0, 6.5 Hz, 1H), 2.55 – 2.31 (m, 2H), 0.95 (d, *J* = 6.6 Hz, 3H).

^{13}C NMR (126 MHz, $CDCl_3$) δ 144.0, 141.2, 140.0, 138.8, 129.8 (2C), 128.8 (2C), 127.3, 127.1 (2C), 127.0 (2C), 113.0, 43.0, 39.4, 19.5.

Spectra

HRMS (APCI): calculated for $C_{17}H_{18}$ $[M]^+$: 222.1408; found: 222.1404.

(*S*)-5-(2-Methylbut-3-en-1-yl)benzo[d][1,3]dioxole (2f)

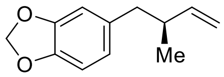

From cyclopropyl methyl boronic ester **1a** (58.8 mg, 0.30 mmol) and 5-bromo-2H-1,3-benzodioxole (24 µL, 0.20 mmol), following the general procedure A described above, compound **2f** was obtained. Purification by flash column chromatography (pentane) afforded **2f** (31.0 mg, 0.16 mmol, 81%) as a colorless oil.

[α] ^{25} $_D$ = - 18.9 (*c* = 1.0, $CHCl_3$).

Rf = 0.52 (10 % EtOAc/cyclohexane).

^{1}H NMR (500 MHz, $CDCl_3$) δ 6.72 (d, *J* = 7.8 Hz, 1H), 6.65 (d, *J* = 1.8 Hz, 1H), 6.59 (dd, *J* = 7.9, 1.8 Hz, 1H), 5.92 (s, 2H), 5.77 (ddd, *J* = 17.2, 10.3, 6.8 Hz, 1H), 4.98 – 4.89 (m, 2H), 2.60 (dd, *J* = 13.2, 6.6 Hz, 1H), 2.48 – 2.33 (m, 2H), 0.98 (d, *J* = 6.6 Hz, 3H).

^{13}C NMR (126 MHz, $CDCl_3$) δ 147.5, 145.7, 144.0, 134.7, 122.1, 112.9, 109.7, 108.0, 100.8, 43.1, 39.6, 19.4.

Spectra

HRMS (APCI): calculated for $C_{12}H_{14}O_2$ $[M]^+$: 190.0994; found: 190.0989.

Spectroscopic data are in agreement with those described in the literature.[7]

(*S*)-1-Methoxy-2-(2-methylbut-3-en-1-yl)benzene (**2g**)

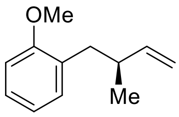

From cyclopropyl methyl boronic ester **1a** (58.8 mg, 0.30 mmol) and 2-Bromophenyl methyl ether (37.4 mg, 0.20 mmol), following the general procedure A described above, compound **2g** was obtained. Purification by flash column chromatography (pentane) afforded **2g** (19.6 mg, 0.11 mmol, 56%) as a colorless oil.

[α] $^{25}_{D}$ = - 33.5 (*c* = 1.0, $CHCl_3$).

Rf = 0.55 (10 % EtOAc/cyclohexane).

^{1}H NMR (500 MHz, $CDCl_3$) δ 7.21 (td, *J* = 7.7, 1.8 Hz, 1H), 7.12 (dd, *J* = 7.4, 1.9 Hz, 1H), 6.90 (td, *J* = 7.4, 1.2 Hz, 1H), 6.88 (d, *J* = 8.2 Hz, 1H), 5.86 (ddd, *J* = 17.1, 10.3, 6.7 Hz, 1H), 5.00 – 4.89 (m, 2H), 3.84 (s, 3H), 2.77 – 2.67 (m, 1H), 2.66 – 2.49 (m, 2H), 1.02 (d, *J* = 6.6 Hz, 3H).

^{13}C NMR (126 MHz, $CDCl_3$) δ 157.8, 144.6, 131.1, 129.4, 127.2, 120.2, 112.3, 110.4, 55.3, 37.8, 37.5, 19.6.

Spectra

HRMS (APCI): calculated for $C_{12}H_{16}O$ $[M]^+$: 176.1201; found: 176.1193.

(*S*)-1-(4-(2-Methylbut-3-en-1-yl)phenyl)piperidine (**2h**)

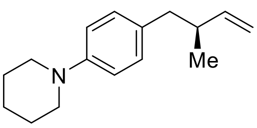

From cyclopropyl methyl boronic ester **1a** (58.8 mg, 0.30 mmol) and 4-(N-Piperidyl)bromobenzene (35.6 µL, 0.20 mmol), following the general procedure A described above, compound **2h** was obtained. Purification by flash column chromatography (pentane) afforded **2h** (31.5 mg, 0.137 mmol, 69%) as a colorless oil.

[α] $^{25}_{D}$ = - 9.1 (*c* = 1.0, $CHCl_3$).

Rf = 0.52 (10 % EtOAc/cyclohexane).

^{1}H NMR (500 MHz, $CDCl_3$) δ 7.03 (d, *J* = 8.7 Hz, 2H), 6.87 (d, *J* = 8.1 Hz, 2H), 5.80 (ddt, *J* = 16.5, 10.2, 6.3 Hz, 1H), 4.98 – 4.88 (m, 2H), 3.19 – 3.09 (m, 4H), 2.60 (m, 1H), 2.51 – 2.34 (m, 2H), 1.76 – 1.65 (m, 4H), 1.57 (m, 2H), 0.97 (d, *J* = 6.3 Hz, 3H).

^{13}C NMR (126 MHz, $CDCl_3$) δ 144.4, 131.9, 129.9 (2C), 118.2, 116.6, 112.6 (2C), 51.2, 50.6, 42.5, 39.5, 26.1, 25.8, 24.4, 19.4.

Spectra

HRMS (APCI): calculated for $C_{16}H_{23}N$ $[M]^+$: 229.1830; found: 229.1827.

(*S*)-1-Chloro-4-(2-methylbut-3-en-1-yl)benzene (**2i**)

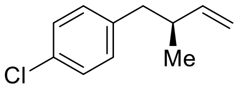

From cyclopropyl methyl boronic ester **1a** (58.8 mg, 0.30 mmol) and 4-Chlorobromobenzene (23.6 µL, 0.20 mmol), following the general procedure A described above, compound **2i** was obtained. Purification by flash column chromatography (pentane) afforded **2i** (26.4 mg, 0.146 mmol, 73%) as a colorless oil.

[α] $^{25}_{D}$ = - 16.7 (*c* = 1.0, $CHCl_3$).

Rf = 0.79 (10 % EtOAc/cyclohexane).

^{1}H NMR (300 MHz, $CDCl_3$) δ 7.23 (d, *J* = 8.4 Hz, 2H), 7.07 (d, *J* = 8.6 Hz, 2H), 5.75 (ddd, *J* = 17.5, 9.9, 6.9 Hz, 1H), 4.99 – 4.86 (m, 2H), 2.64 (dd, *J* = 13.2, 6.9 Hz, 1H), 2.56 – 2.31 (m, 2H), 0.98 (d, *J* = 6.6 Hz, 3H).

^{13}C NMR (126 MHz, $CDCl_3$) δ 143.6, 139.2, 131.7, 130.7 (2C), 128.3 (2C), 113.2, 42.6, 39.4, 19.5.

Spectra

HRMS (ESI^+): calculated for $C_{11}H_{13}Cl$ $[M]^+$: 180.0710; found: 180.0704.

(*S*)-1-Chloro-3-(2-methylbut-3-en-1-yl)benzene (**2j**)

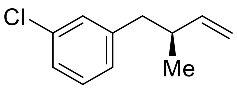

From cyclopropyl methyl boronic ester **1a** (58.8 mg, 0.30 mmol) and 1-Bromo-3-chloro-benzene (23.5 µL, 0.20 mmol), following the general procedure A described above, compound **2j** was obtained. Purification by flash column chromatography (pentane) afforded **2j** (26.3 mg, 0.146 mmol, 73%) as a colorless oil with impurities in the aromatic region due to the close polarity of the SM and product.

[α] $^{25}_{D}$ = - 15.0 (*c* = 0.6, $CHCl_3$).

Rf = 0.79 (10 % EtOAc/cyclohexane).

^{1}H NMR (300 MHz, $CDCl_3$) δ 7.34 – 7.27 (m, 1H), 7.26 (m, 2H), 7.23 (m, 1H), 7.11 (dt, *J* = 6.7, 2.0 Hz, 1H), 5.94 – 5.77 (m, 1H), 5.07 – 5.03 (m, 1H), 5.00 (d, *J* = 1.1 Hz, 1H), 2.74 (dd, *J* = 13.1, 6.8 Hz, 1H), 2.72 – 2.42 (m, 2H), 1.08 (d, *J* = 6.6 Hz, 3H).

^{13}C NMR (75 MHz, $CDCl_3$) δ 143.5, 142.9, 134.0, 129.5, 129.4, 127.6, 126.2, 113.3, 43.0, 39.3, 19.5.

Spectra

HRMS (APCI): calculated for $C_{11}H_{13}Cl$ $[M]^+$: 180.0710; found: 180.0706.

Methyl (*S*)-4-(2-methylbut-3-en-1-yl)benzoate (**2k**)

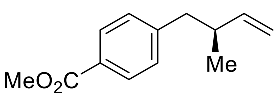

From cyclopropyl methyl boronic ester 1a (58.8 mg, 0.30 mmol) and Methyl 4-Bromobenzoate (25.5 µL, 0.20 mmol), following the general procedure A described

above, compound **2k** was obtained. Purification by flash column chromatography (pentane) afforded **2k** (39.3 mg, 0.192 mmol, 97%) as a colorless oil.

[α] ^{25} $_{D}$ = - 1.8 (*c* = 0.7, $CHCl_3$).

Rf = 0.57 (10 % EtOAc/cyclohexane).

^{1}H NMR (300 MHz, $CDCl_3$) δ 7.95 (d, *J* = 8.5 Hz, 2H), 7.21 (d, *J* = 9.1 Hz, 2H), 5.85 – 5.67 (m, 1H), 4.96 – 4.93 (m, 1H), 4.89 (m, 1H), 3.89 (s, 3H), 2.72 (dd, *J* = 13.2, 7.1 Hz, 1H), 2.59 (m, 1H), 2.52 – 2.38 (m, 1H), 1.00 (d, *J* = 6.7 Hz, 3H).

^{13}C NMR (126 MHz, $CDCl_3$) δ 167.2, 146.3, 143.4, 129.5 (2C), 129.3 (2C), 127.9, 113.3, 52.0, 43.2, 39.2, 19.5.

Spectra

HRMS (APCI): calculated for $C_{13}H_{16}O_2$ $[M]^+$: 204.1150; found: 204.1156.

Ethyl (*S*)-2-(2-methylbut-3-en-1-yl)-4-(trifluoromethyl)pyrimidine-5-carboxylate (**2l**)

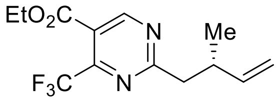

From cyclopropyl methyl boronic ester **1a** (58.8 mg, 0.30 mmol) and ethyl 2-chloro-4-(trifluoromethyl)pyrimidine-5-carboxylate (50.9 mg, 0.20 mmol), following the general procedure B described above, compound **2l** was obtained. Purification by flash column chromatography (20% Et_2O in pentane) afforded **2l** (35.4 mg, 0.123 mmol, 61%) as a colorless oil.

[α] ^{25} $_{D}$ = - 7.3 (*c* = 1.0, $CHCl_3$).

Rf = 0.24 (10 % EtOAc/cyclohexane).

^{1}H NMR (300 MHz, $CDCl_3$) δ 9.12 (s, 1H), 5.79 (ddd, *J* = 17.4, 10.3, 7.2 Hz, 1H), 5.01 – 4.83 (m, 2H), 4.44 (q, *J* = 7.1 Hz, 2H), 3.20 – 3.01 (m, 2H), 2.95 (m, 1H), 1.40 (t, *J* = 7.1 Hz, 3H), 1.08 (d, *J* = 6.7 Hz, 2H).

^{13}C NMR (75 MHz, $CDCl_3$) δ 172.8, 163.8, 159.7, 153.1 (q, *J* = 37 Hz), 142.7, 121.6, 120.3 (q, *J* = 277 Hz), 113.8, 63.0, 46.1, 37.4, 20.0, 14.0.

^{19}F NMR (282 MHz, $CDCl_3$) δ -66.3.

Spectra

HRMS (APCI): calculated for $C_{13}H_{15}F_3N_2O_2$ $[M]^+$: 288.1085; found: 288.1076.

(*S*)-2-Methyl-6-(2-phenethylbut-3-en-1-yl)quinoline (**2m**)

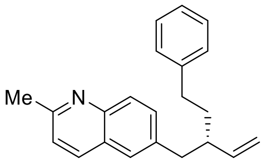

From cyclopropyl methyl boronic ester **1d** (85.9 mg, 0.30 mmol) and 6-bromo-2-methylquinoline (44.4 mg, 0.20 mmol), following the general procedure B described above, compound **2m** was obtained. Purification by flash column chromatography (20% Et_2O in pentane) afforded **2m** (52 mg, 0.170 mmol, 82%) as a colorless oil.

[α] ^{25} $_D$ = + 16.0 (*c* = 1.0, $CHCl_3$).

Rf = 0.41 (30 % EtOAc/cyclohexane).

^{1}H NMR (300 MHz, $CDCl_3$) δ 7.92 (dd, *J* = 10.9, 8.8 Hz, 2H), 7.46 (m, 2H), 7.29 – 7.19 (m, 3H), 7.16 (m, 1H), 7.14 – 7.06 (m, 2H), 5.68 (ddd, *J* = 17.1, 10.3, 8.6 Hz, 1H), 5.00 (dd, *J* = 10.5, 1.9 Hz, 1H), 4.91 (ddd, *J* = 17.1, 1.9, 0.9 Hz, 1H), 2.82 (t, *J* = 6.9 Hz, 2H), 2.72 (s, 3H), 2.72- 2.64 (m, 1H), 2.58 – 2.47 (m, 1H), 2.47 – 2.36 (m, 1H), 1.86 – 1.70 (m, 1H), 1.68 – 1.50 (m, 1H).

^{13}C NMR (76 MHz, $CDCl_3$) δ 158.3, 146.8, 142.5, 141.9, 138.0, 135.9, 131.7, 128.5 (2C), 128.4 (2C), 128.3, 127.1, 126.5, 125.8, 122.0, 115.7, 45.2, 41.8, 36.0, 33.6, 25.4.

Spectra

HRMS (APCI): calculated for $C_{22}H_{23}N$ $[M]^+$: 301.1830; found: 301.1814.

tert-Butyl (*R*)-6-(2-(((*tert*-butyldimethylsilyl)oxy)methyl)but-3-en-1-yl)nicotinate (**2n**)

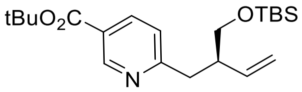

From cyclopropyl methyl boronic ester **1e** (97.9 mg, 0.30 mmol, 1.5 equiv) and 6-bromo-nicotinic acid tert-butyl ester (51.6 mg, 0.20 mmol), following the general procedure B described above, compound **2n** was obtained. Purification by flash column chromatography (40% Et_2O in pentane) afforded **2n** (55.2 mg, 0.146 mmol, 73%) as a colorless oil.

[α] ^{25} $_D$ = - 7.3 (*c* = 1.0, $CHCl_3$).

Rf = 0.28 (10 % EtOAc/cyclohexane).

^{1}H NMR (500 MHz, $CDCl_3$) δ 9.09 – 9.04 (m, 1H), 8.10 (dd, *J* = 8.1, 2.3 Hz, 1H), 7.17 (d, *J* = 8.1 Hz, 1H), 5.73 (ddd, *J* = 17.5, 10.8, 8.1 Hz, 1H), 5.02 – 4.88 (m, 2H), 3.62 – 3.53 (m, 2H), 3.10 (dd, *J* = 12.9, 5.3 Hz, 1H), 2.84 – 2.71 (m, 2H), 1.59 (s, 9H), 0.89 (s, 9H), 0.03 (s, 6H).

^{13}C NMR (126 MHz, $CDCl_3$) δ 165.0, 164.8, 150.6, 139.0, 137.0, 125.3, 123.3, 116.2, 81.8, 66.0, 46.6, 40.1, 28.3 (3C), 26.0 (3C), 18.5, -5.2, -5.3.

Spectra

HRMS (APCI): calculated for $C_{21}H_{35}NO_3Si$ $[M]^+$: 377.2386; found: 377.2380.

(*S*)-2-Fluoro-4-(2-methylbut-3-en-1-yl)pyridine (**2o**)

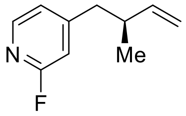

From cyclopropyl methyl boronic ester **1a** (58.8 mg, 0.30 mmol) and 4-bromo-2-fluoropyridine (20.5 µL, 0.20 mmol), following the general procedure A described above, compound **2o** was obtained. Purification by flash column chromatography (pentane) afforded **2o** (22.3 mg, 0.135 mmol, 68%) as a colorless oil.

[α] $^{25}{}_{D}$ = - 18.1 (*c* = 1.0, $CHCl_3$).

Rf = 0.39 (10 % EtOAc/cyclohexane).

^{1}H NMR (500 MHz, $CDCl_3$) δ 8.09 (d, *J* = 5.0 Hz, 1H), 6.96 (dd, *J* = 3.3, 1.9 Hz, 1H), 6.71 (s, 1H), 5.72 (ddd, *J* = 17.4, 10.5, 7.2 Hz, 1H), 4.98 – 4.89 (m, 2H), 2.68 (dd, *J* = 13.4, 7.5 Hz, 1H), 2.59 (dd, *J* = 13.4, 7.0 Hz, 1H), 2.49 (sex, *J* = 6.9 Hz, 1H), 1.03 (d, *J* = 6.7 Hz, 3H).

^{13}C NMR (126 MHz, $CDCl_3$) δ 164.2 (d, *J* = 237.6 Hz), 155.8 (d, *J* = 7.8 Hz), 147.3 (d, *J* = 15.2 Hz), 142.5, 122.5 (d, *J* = 4.1 Hz), 114.1, 110.0 (d, *J* = 36.8 Hz), 42.4, 38.7, 19.7.

^{19}F NMR (471 MHz, $CDCl_3$) δ -69.3.

Spectra

HRMS (APCI): calculated for $C_{10}H_{12}FN$ $[M]^+$: 165.0954; found: 165.0952.

(*S*)-4-(6-(2-Methylbut-3-en-1-yl)pyrazin-2-yl)morpholine (**2p**)

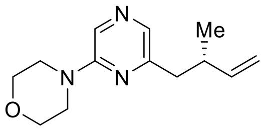

From cyclopropyl methyl boronic ester **1a** (58.8 mg, 0.30 mmol, 1.5 equiv) and 2-Chloro-6-morpholinopyrazine (39.9 mg, 0.20 mmol), following the general procedure B described above, compound **2p** was obtained. Purification by flash column chromatography (40% Et_2O in pentane) afforded **2p** (26.5 mg, 0.114 mmol, 57%) as a colorless oil.

[α] $^{25}{}_{D}$ = - 20.7 (*c* = 1.0, $CHCl_3$).

Rf = 0.16 (10 % EtOAc/cyclohexane).

^{1}H NMR (500 MHz, $CDCl_3$) δ 7.92 (s, 1H), 7.73 (s, 1H), 5.79 (ddd, *J* = 17.1, 10.4, 6.5 Hz, 1H), 4.98 – 4.85 (m, 2H), 3.85 – 3.77 (m, 4H), 3.60 – 3.47 (m, 4H), 2.76 – 2.62 (m, 2H), 2.61 – 2.51 (m, 1H), 1.02 (d, *J* = 6.4 Hz, 3H).

^{13}C NMR (126 MHz, $CDCl_3$) δ 154.6, 153.2, 143.6, 133.7, 127.8, 113.1, 66.7 (2C), 45.0 (2C), 42.2, 37.3, 19.7.

Spectra

HRMS (APCI): calculated for $C_{13}H_{19}N_3O$ $[M]^+$: 233.1528; found: 233.1525.

(*S*)-1-Methyl-5-(2-methylbut-3-en-1-yl)-1H-indole (**2q**)

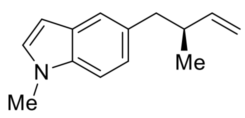

From cyclopropyl methyl boronic ester **1a** (58.8 mg, 0.30 mmol) and 5-Bromo-1-methylindole (28.6 µL, 0.20 mmol), following the general procedure A described above, compound **2q** was obtained. Purification by flash column chromatography (pentane) afforded **2q** (20.1 mg, 0.101 mmol, 50%) as a colorless oil.

[α] ^{25} $_{\mathbf{D}}$ = - 27.1 (*c* = 1.0, $CHCl_3$).

Rf = 0.48 (10 % EtOAc/cyclohexane).

^{1}H NMR (300 MHz, $CDCl_3$) δ 7.39 (dd, *J* = 1.6, 0.8 Hz, 1H), 7.24 (d, *J* = 8.4 Hz, 1H), 7.09 – 6.98 (m, 2H), 6.42 (dd, *J* = 3.1, 0.9 Hz, 1H), 5.86 (ddd, *J* = 17.1, 10.3, 6.7 Hz, 1H), 5.03 – 4.86 (m, 2H), 3.77 (s, 3H), 2.81 (dd, *J* = 13.1, 6.5 Hz, 1H), 2.61 (dd, *J* = 13.1, 7.8 Hz, 1H), 2.49 (m, 1H), 1.00 (d, *J* = 6.6 Hz, 3H).

^{13}C NMR (75 MHz, $CDCl_3$) δ 144.6, 135.6, 131.6, 128.9, 128.7, 123.4, 121.1, 112.5, 108.8, 100.6, 43.5, 39.9, 33.0, 19.4.

Spectra

HRMS (APCI): calculated for $C_{14}H_{17}N$ $[M]^{+}$: 199.1360; found: 199.1356.

N,N-Dimethyl-3-(4-((*S*)-2-methylbut-3-en-1-yl)phenyl)-3-(pyridin-2-yl)propan-1-amine (**2r**)

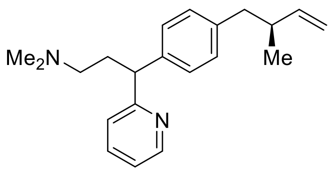

From cyclopropyl methyl boronic ester **1a** (58.8 mg, 0.30 mmol) and brompheniramine (63.9 mg, 0.20 mmol), following the general procedure A described above, compound **2r** was obtained. Purification by flash column chromatography (pentane) afforded **2r** (58 mg, 0.188 mmol, 94%) as a yellowish oil.

[α] ^{25} $_{\mathbf{D}}$ = + 2.7 (*c* = 1.0, $CHCl_3$).

Rf = 0.29 (20 % MeOH/DCM).

^{1}H NMR (300 MHz, $CDCl_3$) δ 8.51 (dd, *J* = 4.9, 0.9 Hz, 1H), 7.51 (td, *J* = 7.6, 1.9 Hz, 1H), 7.13 (m, 4H), 7.08 – 7.04 (m, 1H), 7.02 (m, 2H), 5.71 (ddd, *J* = 17.1, 10.3, 6.6 Hz, 1H), 4.93 – 4.79 (m, 2H), 4.15 (t, *J* = 7.5 Hz, 1H), 3.05 – 2.89 (m, 2H), 2.75 (s, 6H), 2.64 – 2.25 (m, 4H), 0.90 (d, *J* = 6.5 Hz, 3H).

^{13}C NMR (76 MHz, $CDCl_3$) δ 161.2, 149.1, 143.8, 139.6, 139.2, 136.9, 129.7 (2C), 127.5 (2C), 123.7, 121.9, 112.7, 56.6, 49.9, 42.9, 42.7, 39.0, 28.8, 19.3.

Spectra

HRMS (ACPI): calculated for $C_{22}H_{31}N_2$ $[M+H]^{+}$: 323.2487; found: 323.2492.

(*S*)-N-(2-Fluoro-4-(2-methylbut-3-en-1-yl)phenyl)-6-methoxy-7-((1-methylpiperidin-4-yl)methoxy)quinazolin-4-amine (**2s**)

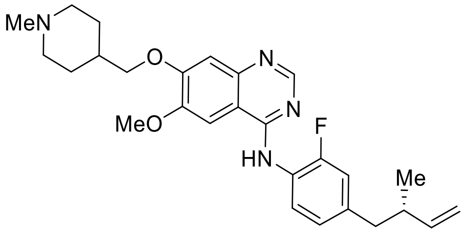

From cyclopropyl methyl boronic ester **1a** (118 mg, 0.60 mmol, 3.0 equiv.) and N-(4-bromo-2-fluorophenyl)-6-methoxy-7-((1-methylpiperidin-4-yl)methoxy)quinazolin-4-amine (95.1 mg, 0.20 mmol), following the general procedure B described above, compound **2s** was obtained. Purification by flash column chromatography (10% MeOH in CH_2Cl_2) and addition of chloroform in which the product is insoluble and filtration afforded **2s** (54 mg, 0.12 mmol, 59%) as a white solid.

mp.= 170 °C (decomp.)

[α] ^{25} $_{D}$ = - 1.8 (*c* = 1.0, $CHCl_3$).

Rf = 0.15 (20 % MeOH/DCM).

^{1}**H NMR** (300 MHz, DMSO) δ 9.73 (s, 1H), 8.35 (s, 2H), 7.90 (s, 1H), 7.64 (dd, *J* = 10.0, 2.2 Hz, 2H), 7.58 – 7.40 (m, 3H), 7.23 (s, 1H), 5.75 (s, 1H), 4.07 (d, *J* = 6.4 Hz, 3H), 3.96 (s, 4H), 3.34 (d, *J* = 10.1 Hz, 4H), 3.13 (s, 2H), 2.92 (t, *J* = 11.0 Hz, 2H), 2.67 (s, 4H), 2.08 (s, 0H), 1.98 (d, *J* = 13.4 Hz, 2H), 1.66 (m, 3H), 1.22 (s, 1H).

^{13}**C NMR** (76 MHz, DMSO) δ 158.4, 156.9, 155.0, 153.5, 152.9, 149.0, 146.8, 129.5 (d, *J* = 2.8 Hz), 127.4 (d, *J* = 3.6 Hz), 126.5, 126.4, 119.4, 119.1, 117.5 (d, *J* = 9.1 Hz), 108.3 (d, *J* = 66.6 Hz), 102.4, 71.6, 56.3, 54.9, 52.6, 42.7, 32.3, 31.2, 29.0, 25.7.

^{19}**F NMR** (282 MHz, MeOD) δ -118.55.

Spectra

HRMS (ACPI): calculated for $C_{27}H_{33}FN_4O_2$ [M^+] : 464.2587; found: 464.2576.

(*S*)-4-(Cyclopropylethynyl)-6-((*S*)-2-methylbut-3-en-1-yl)-4-(trifluoromethyl)-1,4-dihydro-2H-benzo[d][1,3]oxazin-2-one (**2t**)

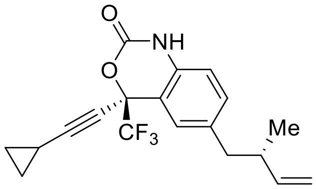

From cyclopropyl methyl boronic ester **1a** (118 mg, 0.60 mmol, 3 equiv) and Efavirenz (63.1 mg, 0.20 mmol), following the general procedure B described above, compound **2t** was obtained. Purification by flash column chromatography (20% Et_2O in pentane) afforded **2t** (26.6 mg, 0.076 mmol, 38%) as a colorless oil.

[α] ^{25} $_{D}$ = - 1.8 (*c* = 1.0, $CHCl_3$).

Rf = 0.48 (10 % EtOAc/cyclohexane).

^{1}H NMR (300 MHz, $CDCl_3$) δ 7.99 – 7.92 (m, 1H), 7.79-7.75 (m, 1H), 7.57 – 7.51 (m, 1H), 7.47 (s, 1H), 5.89 – 5.71 (m, 1H), 4.99 – 4.92 (m, 1H), 4.91 (d, *J* = 1.1 Hz, 1H), 2.86 (dd, *J* = 13.5, 7.3 Hz, 1H), 2.74 (dd, *J* = 13.4, 7.3 Hz, 1H), 2.56 (sex, *J* = 6.6 Hz, 1H), 2.33 – 2.08 (m, 1H), 1.26 – 1.15 (m, 2H), 1.13-1.10 (m, 2H), 1.04 (d, *J* = 6.7 Hz, 3H).

^{13}C NMR (75 MHz, $CDCl_3$) δ 163.5, 162.1, 147.9, 143.5, 139.6, 133.6 (q, *J* = 31.0 Hz), 132.2, 129.3, 123.4, 121.4, 117.1 (q, *J* = 5.2 Hz), 113.4, 68.1, 43.5, 39.4, 19.6, 18.2, 10.8.

^{19}F NMR (282 MHz, $CDCl_3$) δ -61.6.

Spectra

HRMS (ESI^+): calculated for $C_{19}H_{18}F_3NO_2$ $[M]^+$: 349.1290; found: 349.1285.

4. Negishi cross-coupling reaction with acyl halides

4.1. Optimization of Negishi reaction conditions with acyl halides

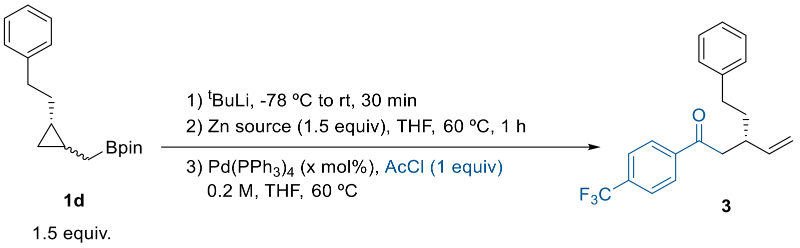

Entry [a]	Zn Source	Pd (x mol%)	Yield **3** (%) [b]
1	$Zn(OAc)_2$	5	67
2	ZnI_2 or $ZnCl_2$	5	74

[a] Reaction conditions: ArBr (0.2 mmol, 1 equiv.), **1d** (1.5 equiv.), ^{t}BuLi (1.6 equiv.), Zn (1.6 equiv.), $Pd(PPh_3)_4$ (x mol%), THF (0.2 M), 60 °C and 16 h. [b] Yield and ratio calculated by ^{1}H NMR using an internal standard.

4.2. General procedure for the Negishi cross-coupling reaction with acyl halides

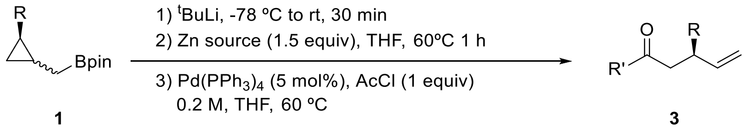

In an oven-dried vial, cyclopropyl methyl boronic ester **1** (0.3 mmol, 1.5 equiv.) was weighed and vacuum-argon cycles were done. Under argon atmosphere, 0.5 mL of anhydrous THF was added, the solution was cooled to -78 °C (dry ice/acetone bath) and *tert*-butyllithium (1.6 equiv.) was added dropwise by a syringe. Then, the reaction mixture was stirred while warming to room temperature for 30 min. After this time, the reaction vial was transferred into the glovebox and zinc chloride (0.3 mmol, 1.5 equiv.) was added as a solid. The vial was brought out of the glovebox and the reaction was stirred at 60 °C (oil bath) for 1 hour. In a separate vial, acyl chloride (0.2 mmol, 1 equiv.), and tetrakis(triphenylphosphine)palladium(0) (5 mol%) were dissolved in 0.5 mL THF. Then the generated alkyl zinc solution was added and the reaction was allowed to stir at 60 °C for 16 hours. Upon completion, the reaction mixture was passed through a short pad of silica gel, washed with EtOAc and concentrated under reduced pressure. The resulting crude oil was purified by silica gel column chromatography (pentane/EtOAc) to furnish the desired product **3**.

4.3. Characterization data of Negishi homoallylation products with acyl chlorides

(*S*)-3-Methyl-1-phenylpent-4-en-1-one (**3a**)

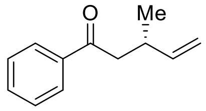

From cyclopropyl methyl boronic ester **1a** (58.8 mg, 0.30 mmol) and benzoyl chloride (28.1 mg, 0.20 mmol), following the general procedure described above, compound **3a** was obtained. Purification by flash column chromatography (n-hexane/EtOAc, 9:1) afforded **3a** (20.3 mg, 0.117 mmol, 58%) as a colorless oil.

[α] ^{25} $_{D}$ = - 19.7 (*c* = 1.0, $CHCl_3$).

Rf = 0.52 (10 % EtOAc/cyclohexane).

^{1}H NMR (300 MHz, $CDCl_3$) δ 7.97 (m, 2H), 7.59-7.51 (m, 3H), 5.91-5.78 (m, 1H), 4.99 (m, 2H), 3.08 – 2.89 (m, 3H), 1.10 (d, *J* = 6.5 Hz, 3H).

Spectroscopic data are in agreement with those described in the literature. [8]

(*S*)-1-(4-Bromophenyl)-3-methylpent-4-en-1-one (3b)

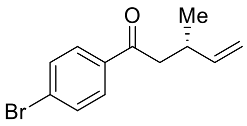

From cyclopropyl methyl boronic ester **1a** (58.8 mg, 0.30 mmol) and 4-bromobenzoyl chloride (44.0 mg, 0.20 mmol), following the general procedure described above, compound **3b** was obtained. Purification by flash column chromatography (n-hexane/EtOAc, 9:1) afforded **3b** (27.7 mg, 0.109 mmol, 54%) as a colorless oil.

[α] ^{25} $_{D}$ = - 18.2(*c* = 1.0, $CHCl_3$).

Rf = 0.55 (10 % EtOAc/cyclohexane).

^{1}H NMR (300 MHz, $CDCl_3$) δ 7.73 (d, 2H), 7.52 (d, 2H), 5.84-5.69 (m, 1H), 5.01-4.87 (m, 2H), 2.97 – 2.74 (m, 3H), 1.02 (d, *J* = 6.5 Hz, 3H).

HRMS (APCI): calculated for $C_{12}H_{13}BrO$ $[M]^+$: 252.0150; found: 252.0139.

Spectroscopic data are in agreement with those described in the literature. [9]

(*S*)-1-(4-Methoxyphenyl)-3-methylpent-4-en-1-one (3c)

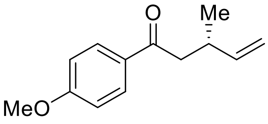

From cyclopropyl methyl boronic ester **1a** (58.8 mg, 0.30 mmol) and 4-methoxybenzoyl chloride (34.1 mg, 0.20 mmol), following the general procedure described above, compound **3c** was obtained. Purification by flash column chromatography (n-hexane/EtOAc, 9:1) afforded **3c** (31.3 mg, 0.153 mmol, 77%) as a colorless oil.

[α] ^{25} $_{D}$ = - 17.2 (*c* = 1.0, $CHCl_3$).

Rf = 0.21 (10 % EtOAc/cyclohexane).

^{1}H NMR (300 MHz, $CDCl_3$) δ 7.95-7.90 (m, 2H), 6.94-6.89 (m, 2H), 5.90-5.79 (m, 1H), 5.04-4.92 (m, 2H), 3.85 (s, 3H), 3.00-2.79 (m, 3H), 1.08 (d, J = 6.5 Hz, 3H).

HRMS (APCI): calculated for $C_{13}H_{16}O_2$ $[M]^+$: 204.1150; found: 204.1144.

Spectroscopic data are in agreement with those described in the literature. [10]

(*S*)-3-Methyl-1-(o-tolyl)pent-4-en-1-one (**3d**)

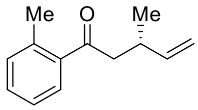

From cyclopropyl methyl boronic ester **1a** (58.8 mg, 0.30 mmol) and 2-methylbenzoyl chloride (30.9 mg, 0.20 mmol), following the general procedure described above, compound **3d** was obtained. Purification by flash column chromatography (n-hexane/EtOAc, 9:1) afforded **3d** (29.9 mg, 0.159 mmol, 79%) as a colorless oil.

[α] $^{25}_{D}$ = - 16.4 (c = 1.0, $CHCl_3$).

Rf = 0.48 (10 % EtOAc/cyclohexane).

^{1}H NMR (300 MHz, $CDCl_3$): δ 7.80-7.70 (m, 2H), 7.40-7.31 (m, 2H), 5.89-5.78 (m, 1H), 5.05-4.94 (m, 2H), 3.01-2.81 (m, 1H), 2.49 (s, 3H), 1.10 (d, J = 6.2 Hz, 3H).

HRMS (APCI): calculated for $C_{13}H_{16}O$ $[M]^+$: 188.1201; found: 188.1193.

Spectroscopic data are in agreement with those described in the literature.[11]

(*S*)-3-Phenethyl-1-(4-(trifluoromethyl)phenyl)pent-4-en-1-one (**3e**)

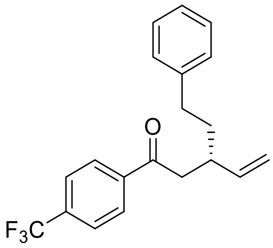

From cyclopropyl methyl boronic ester **1d** (58.8 mg, 0.30 mmol) and 4-(trifluoromethyl)benzoyl chloride (41.7 mg, 0.20 mmol), following the general procedure described above, compound **3e** was obtained. Purification by flash column chromatography (n-hexane/EtOAc, 9:1) afforded **3e** (36.8 mg, 0.111 mmol, 55%) as a colorless oil.

[α] $^{25}_{D}$ = - 14.3 (c = 1.0, $CHCl_3$).

Rf = 0.39 (10 % EtOAc/cyclohexane).

^{1}H NMR (300 MHz, $CDCl_3$) δ 8.02-7.98 (m, 2H), 7.73-7.70 (m, 2H), 7.30-7.16 (m, 5H), 5.80-5.68 (m, 1H), 5.10-5.03 (m, 2H), 3.03 (dd, J = 6.8, 1.7 Hz, 2H), 2.87-2.55 (m, 3H), 1.90-1.64 (m, 2H).

^{13}C NMR (76 MHz, $CDCl_3$) δ 198.3, 142.2, 140.9, 140.1 128.6 (2C), 126.1, 125.9 (q, J = 3.8 Hz), 116.0, 44.4, 39.6, 36.5, 33.5.

^{19}F NMR (470.6 MHz, $CDCl_3$) δ -63.1.

[Spectra](#)

HRMS (APCI): calculated for $C_{20}H_{19}F_3O$ $[M]^+$: 332.1388; found: 332.1382.

(*S*)-3-(4-Chlorobenzyl)-1-(4-methoxyphenyl)pent-4-en-1-one (**3f**)

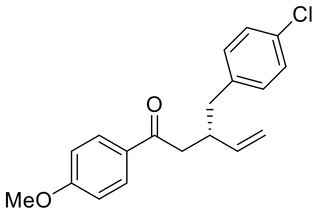

From cyclopropyl methyl boronic ester **1g** (92.0 mg, 0.30 mmol) and 4-methoxybenzoyl chloride (34.1 mg, 0.20 mmol), following the general procedure described above, compound **3f** was obtained. Purification by flash column chromatography (n-hexane/EtOAc, 9:1) afforded **3f** (44.3 mg, 0.141 mmol, 70%) as a colorless oil.

[α] ^{25} $_{D}$ = - 19.1 (*c* = 1.0, $CHCl_3$).

Rf = 0.18 (10 % EtOAc/cyclohexane).

^{1}H NMR (300 MHz, $CDCl_3$) δ 7.89-7.86 (m, 2H), 7.25-7.22 (m, 2H), 7.13-7.10 (m, 2H), 6.94-6.89 (m, 2H), 5.81-5.69 (m, 1H), 4.98-4.89 (m, 2H), 3.86 (s, 3H), 3.09-3.00 (m, 1H), 2.95-2.92 (m, 2H), 2.72 (qd, J = 13.5, 7.2 Hz, 2H), 1.90-1.64 (m, 2H).

^{13}C NMR (126 MHz, $CDCl_3$) δ 197.7, 163.6, 140.6, 138.4, 132.0, 130.8 (2C), 130.5 (2C), 130.4, 128.4 (2C), 115.3, 113.9 (2C), 55.6, 42.3, 41.1, 40.4.

[Spectra](#)

HRMS (APCI): calculated for $C_{19}H_{19}ClO_2$ $[M]^+$: 314.1073; found: 314.1068.

(*R*)-3-(Cyclobutylmethyl)-1-(4-methoxyphenyl)pent-4-en-1-one (**3g**)

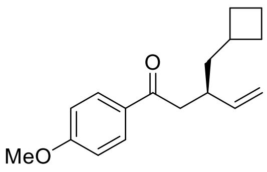

From cyclopropyl methyl boronic ester **1c** (75.1 mg, 0.30 mmol) and 4-methoxybenzoyl chloride (34.1 mg, 0.20 mmol), following the general procedure described above, compound **3g** was obtained. Purification by flash column chromatography (n-hexane/EtOAc, 9:1) afforded **3g** (33.8 mg, 0.131 mmol, 65%) as a colorless oil.

[α] ^{25} $_{D}$ = - 37.4 (*c* = 1.0, $CHCl_3$).

Rf = 0.27 (10 % EtOAc/cyclohexane).

^{1}H NMR (300 MHz, $CDCl_3$) δ 7.95-7.88 (m, 2H), 6.94-6.89 (m, 2H), 5.72-5.60 (m, 1H), 4.98-4.90 (m, 2H), 3.86 (s, 3H), 2.89-2.87 (m, 1H), 2.74-2.62 (m, 1H), 2.40-2.22 (m, 1H), 2.07-1.95 (m, 2H), 1.87-1.72 (m, 2H), 1.67-1.57 (m, 2H), 1.54-1.44 (m, 2H).

^{13}**C NMR** (126 MHz, $CDCl_3$) δ 198.2, 163.6, 142.0, 130.7, 130.6 (2C), 114.5, 113.9 (2C), 55.6, 43.8, 42.4, 38.6, 34.2, 29.1, 28.7, 18.8.

[Spectra]

HRMS (APCI): calculated for $C_{17}H_{22}O_2$ $[M]^+$: 258.1619; found: 258.1613.

(*S*)-4-Methyl-1-phenylhex-5-en-2-one (**3h**)

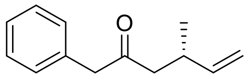

From cyclopropyl methyl boronic ester **1a** (58.8 mg, 0.30 mmol) and 2-phenylacetyl chloride (30.9 mg, 0.20 mmol), following the general procedure described above, compound **3h** was obtained. Purification by flash column chromatography (n-hexane/EtOAc, 9:1) afforded **3h** (14.0 mg, 0.074 mmol, 38%) as a colorless oil.

[α] $^{25}_{D}$ = - 20.1 (*c* = 1.0, $CHCl_3$).

Rf = 0.66 (10 % EtOAc/cyclohexane).

^{1}**H NMR** (300 MHz, $CDCl_3$) δ 7.36-7.17 (m, 5H), 5.78-5.66 (m, 1H), 5.00-4.90 (m, 2H), 3.67 (s, 3H), 3.77-2.67 (m, 1H),2.43 (m, 2H), 0.97 (d, *J* = 6.5 Hz, 3H).

^{13}**C NMR** (76 MHz, $CDCl_3$) δ 207.3, 142.9, 134.2, 129.6 (2C), 128.8 (2C), 127.2, 113.3, 50.9, 48.7, 33.4, 19.8.

[Spectra]

HRMS (APCI): calculated for $C_{13}H_{16}O$ $[M]^+$: 188.1201; found: 188.1182.

(*S*)-1-(2',4'-Difluoro-[1,1'-biphenyl]-4-yl)-3-methylpent-4-en-1-one (**3i**)

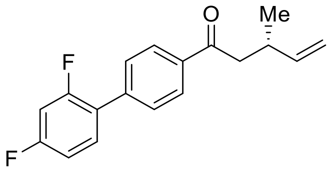

From cyclopropyl methyl boronic ester **1a** (58.8 mg, 0.30 mmol) and 2',4'-difluoro-[1,1'-biphenyl]-4-carbonyl chloride **5** (50.5 mg, 0.20 mmol) prepared by mixing 2',4'-difluoro-[1,1'-biphenyl]-4-carboxylic acid (117 mg, 0.50 mmol), oxalyl chloride (0.056 mL, 0.65 mmol) and DMF (30 mol%, 0.012 mL, 0.150 mmol) in DCM (0.20 M) in quantitative yield after evaporation. Following the general procedure described above, compound **3i** was obtained. Purification by flash column chromatography (n-hexane/EtOAc, 9:1) afforded **3i** (40.0 mg, 0.140 mmol, 70%) as a colorless oil that solidified upon standing.

[α] $^{25}_{D}$ = - 12.2 (*c* = 1.0, $CHCl_3$).

Rf = 0.48 (10 % EtOAc/cyclohexane).

^{1}**H NMR** (300 MHz, $CDCl_3$) δ 8.03-8.00 (m, 2H), 7.62-7.57 (m, 2H), 7.47-7.39 (m, 1H), 7.01-6.89 (m, 1H), 5.92-5.81 (m, 1H), 5.08-4.95 (m, 2H), 3.11-3.00 (m, 1H), 2.98-2.88 (m, 2H), 2.74-2.62 (m, 1H), 1.12 (d, *J* = 6.4 Hz, 3H).

^{13}C NMR (126 MHz, $CDCl_3$) δ ^{13}C NMR (126 MHz, $CDCl_3$) δ 198.9, 161.4 (m, 2C), 143.1, 139.6, 136.5, 131.5 (q, *J* = 4.7 Hz), 129.2 (d, *J* = 3.10 Hz), 128.5, 124.4 (m), 113.2, 112.0 (dd, *J* = 21.2, 3.8 Hz), 104.7 (m), 104.5, 45.3, 33.8, 19.9.

^{19}F NMR (282 MHz, $CDCl_3$) δ -109.85 (d, *J* = 7.7 Hz), -112.88 (d, *J* = 8.2 Hz).

Spectra

HRMS (APCI): calculated for $C_{18}H_{16}F_2O$ $[M]^+$: 286.1169; found: 286.1160.

Spectroscopic data are in agreement with those described in the literature. [9]

5. Negishi cross-coupling reaction with alkenyl halides

5.1. Optimization of Negishi reaction conditions with alkenyl halides

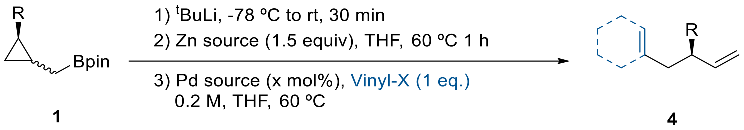

1 4

Entry [a]	R	Vinyl-X	Zn Source	Pd (x mol%)	Yield **4** (%) [b]
**1**	$-CH_2CH_2Ph$	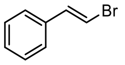	ZnI_2	$Pd(PPh_3)_4$ (5)	60
**2**	$-CH_2CH_2Ph$	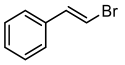	$ZnCl_2$	$Pd(OAc)_2$/CPhos (2)	81%[c]
**3**	$-CH_2CH_2Ph$	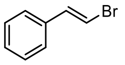	$ZnCl_2$	$PdCl_2$(Amphos) (2)/ NMI (2 equiv)	87%[d]
**4**	$-CH_2CH_2Ph$	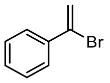	$ZnCl_2$	$Pd(OAc)_2$/CPhos (2)	85%
**5**	$-CH_3$	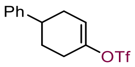	$ZnCl_2$	$Pd(OAc)_2$/CPhos (2)	99%

[a] Reaction conditions: ArBr (0.2 mmol, 1 equiv.), **1** (1.5 equiv.), ^{t}BuLi (1.6 equiv.), Zn (1.6 equiv.), $Pd(PPh_3)_4$ (x mol%), THF (0.2 M), 60 °C and 16 h. [b] Yield and ratio calculated by ^1H NMR using an internal standard. [c] Isomerization of the olefin. [d] Less than 10 % isomerization observed.

5.2. General procedure for the Negishi cross-coupling reaction with alkenyl halides

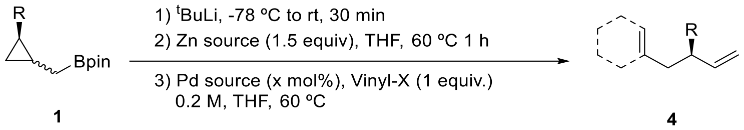

1 4

In an oven-dried vial, cyclopropyl methyl boronic ester **1** (0.3 mmol, 1.5 equiv.) was weighed and vacuum-argon cycles were done. Under argon atmosphere, 0.5 mL of anhydrous THF was added, the solution was cooled to -78 °C (dry ice/acetone bath) and *tert*-butyllithium (1.6 equiv.) was added dropwise by a syringe. Then, the reaction mixture was stirred while warming to room temperature for 30 min. After this time, the reaction vial was transferred into the glovebox and zinc chloride (0.3 mmol, 1.5 equiv.) was added as a solid. The vial was brought out of the glovebox and the reaction was stirred at 60 °C (oil bath) for 1 hour.

In a separate vial, $Pd(OAc)_2$ and CPhos (2 mol%) or $PdCl_2$Amphos (2 mol%) were dissolved in 0.2 mL THF. Then, vinyl halide was added directly if liquid of as a solution (in 0.3 mL) if solid to the reaction vial (at this time NMI when required was added). Then the generated alkyl zinc solution was added and the reaction was allowed to

stir at 60 °C for 16 hours. Upon completion, the reaction mixture was passed through a short pad of silica gel, washed with EtOAc and concentrated under reduced pressure. The resulting crude oil was purified by silica gel column chromatography (pentane/EtOAc) to furnish the desired product **4**.

5.3. Characterization data of Negishi homoallylation products with vinyl (pseudo)halides

(*S,E*)-(4-Vinylhex-1-ene-1,6-diyl)dibenzene (**4a**)

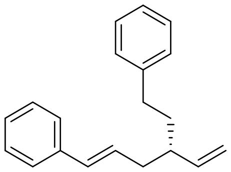

From cyclopropyl methyl boronic ester **1d** (85.9 mg, 0.30 mmol) and (E)-(2-bromovinyl)benzene (90:10, E/Z) (36.6 mg, 0.20 mmol), following the general procedure described above ($PdCl_2(Amphos)_2$, NMI), compound **4a** was obtained. Purification by flash column chromatography and pTLC (n-hexane/EtOAc, 9:1) afforded **4a** (44.7 mg, 0.170 mmol, 85%,82:18, E:Z) as a colorless oil.

[α] ^{25} $_D$ = + 13.1 (*c* = 1.0, $CHCl_3$).

Rf = 0.76 (10 % EtOAc/cyclohexane).

^{1}H NMR (300 MHz, $CDCl_3$) δ 7.46 – 7.16 (m, 10H), 6.60 – 6.36 (m, 1H), 6.24 (dt, *J* = 15.8, 7.0 Hz, 1H), 5.86 – 5.62 (m, 1H), 5.24 – 5.03 (m, 2H), 2.76 (m, 1H), 2.70 – 2.49 (m, 1H), 2.49 – 2.33 (m, 2H), 2.32 – 2.15 (m, 1H), 1.87 (m, 1H), 1.76 – 1.60 (m, 1H).

^{13}C NMR (126 MHz, $CDCl_3$) δ 142.7, 142.3, 137.9, 131.3, 128.9, 128.8, 128.6 (2C), 128.6 (2C), 128.4 (2C), 127.0, 126.1 (2C), 125.8, 115.3, 43.8, 38.8, 36.2, 33.6.

Spectra

HRMS (APCI): calculated for $C_{20}H_{22}$ $[M]^+$: 262.1722; found: 262.1719.

(*R*)-*tert*-Butyl((4,5-dimethyl-2-vinylhex-4-en-1-yl)oxy)dimethylsilane (**4b**)

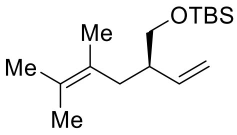

From cyclopropyl methyl boronic ester **1e** (98.0 mg, 0.30 mmol) and 2-bromo-3-methylbut-2-ene (29.8 mg, 0.20 mmol), following the general procedure described above ($Pd(OAc)_2$/CPhos), compound **4b** was obtained. Purification by flash column chromatography (n-hexane/EtOAc, 9:1) afforded **4b** (27.0 mg, 0.101 mmol, 50%) as a colorless oil.

[α] ^{25} $_D$ = + 21.2 (*c* = 1.0, $CHCl_3$).

Rf = 0.88 (10 % EtOAc/cyclohexane).

^{1}H NMR (300 MHz, $CDCl_3$) δ 5.69 (dddd, *J* = 17.1, 10.3, 8.0, 3.7 Hz, 1H), 5.05-4.91 (m, 2H), 3.51 (d, *J* = 5.7 Hz, 2H), 2.38-2.12 (m, 2H), 2.01 (m, 1H), 1.66-1.60 (m, 9H), 1.25 (d, *J* = 2.8 Hz, 3H), 0.89 (s, 6H), 0.03 (s, 6H).

^{13}**C NMR** (126 MHz, $CDCl_3$) δ 140.7, 126.0, 125.5, 114.8, 66.3, 45.7, 36.1, 31.1, 26.1 (3C), 20.8, 19.0, 18.5, -5.2, -5.2.

Spectra

HRMS (APCI): calculated for $C_{16}H_{32}OSi$ $[M]^+$: 268.2222; found: 268.2155.

(*S*)-(3-Vinylhex-5-ene-1,5-diyl)dibenzene (**4c**)

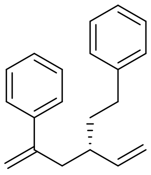

From cyclopropyl methyl boronic ester **1d** (85.9 mg, 0.30 mmol) and (1-bromovinyl)benzene (36.6 mg, 0.20 mmol), following the general procedure described above ($Pd(OAc)_2$, CPhos), compound **4c** was obtained. Purification by flash column chromatography and pTLC (n-hexane/EtOAc, 9:1) afforded **4c** (31.9 mg, 0.122 mmol, 61%) as a colorless oil.

[α] ^{25} $_D$ = + 40.9 (*c* = 1.0, $CHCl_3$).

Rf = 0.76 (10 % EtOAc/cyclohexane).

^{1}**H NMR** (300 MHz, $CDCl_3$) δ 7.34-7.18 (m, 7H), 7.15-7.06 (m, 3H), 5.66-5.54 (m, 1H), 5.23 (d, J = 1.7 Hz, 1H), 5.00-4.82 (m, 3H), 2.66-2.37 (m, 4H), 2.20-2.08 (m, 1H), 1.80-1.68 (m, 1H), 1.58-1.45 (m, 1H).

^{13}**C NMR** (76 MHz, $CDCl_3$) δ 146.9, 142.8, 142.2, 141.4, 128.5 (4C), 128.4 (2C), 127.4, 126.5 (2C), 125.7, 115.0, 114.3, 41.8, 41.5, 36.0, 33.5.

Spectra

HRMS (APCI): calculated for $C_{20}H_{22}$ $[M]^+$: 262.1722; found: 262.1716.

tert-Butyl 3-((*S*)-2-methylbut-3-en-1-yl)-8-azabicyclo[3.2.1]oct-2-ene-8-carboxylate (**4d**)

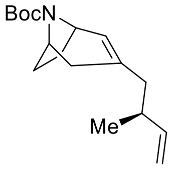

From cyclopropyl methyl boronic ester **1a** (58.8 mg, 0.30 mmol) and tert-butyl 3-(((trifluoromethyl)sulfonyl)oxy)-8-azabicyclo[3.2.1]oct-2-ene-8-carboxylate (71.5 mg, 0.20 mmol), following the general procedure described above ($Pd(OAc)_2$/CPhos), compound **4d** was obtained as a mixture of diastereoisomers. Purification by flash column chromatography (n-hexane/EtOAc, 9:1) afforded **4d** (23.2 mg, 0.08 mmol, 42%) as a colorless oil.

[α] ^{25} $_D$ = - 0.9 (*c* = 1.0, $CHCl_3$).

Rf = 0.30 (10 % EtOAc/cyclohexane).

^{1}**H NMR** (300 MHz, $CDCl_3$) δ 5.74-5.60 (m, 2H), 4.96-4.85 (m, 2H), 4.27 (brs, 2H), 2.67 (brs, 1H), 2.11-1.52 (m, 9H), 1.43 (s, 9H), 1.01-0.80 (m, 3H).

^{13}**C NMR** (76 MHz, $CDCl_3$) δ 154.4, 154.3, 144.5, 144.2, 112.7, 112.6, 79.3, 44.0, 43.8, 35.7, 35.6, 28.7 (2C), 19.7.

Spectra

HRMS (APCI): calculated for $C_{17}H_{28}NO_2$ [M+H$^+$]: 278.2120; found: 278.2109.

Ethyl (*R*,*Z*)-5-(4-chlorobenzyl)hepta-2,6-dienoate (**4e**)

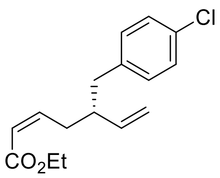

From cyclopropyl methyl boronic ester **1g** (92.0 mg, 0.30 mmol) and ethyl (Z)-3-iodoacrylate (45.2 mg, 0.20 mmol), following the general procedure described above ($PdCl_2(Amphos)_2$/NMI), compound **4e** was obtained. Purification by flash column chromatography and pTLC (n-hexane/EtOAc, 9:1) afforded **4e** (40.1 mg, 0.144 mmol, 72%) as a colorless oil.

[α] ^{25} $_D$ = - 24.3 (*c* = 1.0, $CHCl_3$).

Rf = 0.48 (10 % EtOAc/cyclohexane).

^{1}H NMR (300 MHz, $CDCl_3$) δ 7.25-7.20 (m, 2H), 7.09-7.05 (m,2H), 6.22-6.13 (m, 1H), 5.80 (dt, *J* = 11.5, 1.8 Hz, 1H), 5.62 (ddd, *J* = 17.1, 10.3, 8.2 Hz, 1H), 5.99-5.86 (m, 2H), 4.15 (q, *J* = 7.1 Hz, 2H), 2.86-2.58 (m, 4H), 2.49-2.36 (m, 1H), 1.27 (t, *J* = 7.1 Hz, 3H).

^{13}C NMR (126 MHz, $CDCl_3$) δ 166.6, 148.2, 140.9, 138.7, 130.8 (2C), 128.5 (2C), 120.9, 115.9, 77.3, 60.1, 45.7, 40.9, 33.5, 14.5.

Spectra

HRMS (APCI): calculated for $C_{16}H_{19}ClO_2$ [M]$^+$: 278.1074; found: 278.1068.

(*8S,9S,10R,13R,14S,17R*)-10,13-Dimethyl-3-((*S*)-2-methylbut-3-en-1-yl)-17-((*R*)-6-methylheptan-2-yl)-2,7,8,9,10,11,12,13,14,15,16,17-dodecahydro-1H-cyclopenta[a]phenanthrene (**4f**)

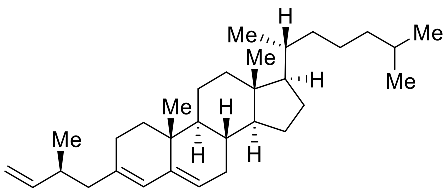

From cyclopropyl methyl boronic ester **1a** (58.8 mg, 0.30 mmol) and (8S,9S,10R,13R,14S,17R)-10,13-dimethyl-17-((R)-6-methylheptan-2-yl)-2,7,8,9,10,11,12,13,14,15,16,17-dodecahydro-1H-cyclopenta[a]phenanthren-3-yl trifluoromethanesulfonate (103.0 mg, 0.20 mmol), following the general procedure described above ($Pd(OAc)_2$/CPhos), compound **4f** was obtained. Purification by flash column chromatography (n-hexane/EtOAc, 9:1) afforded **4f** (80.5 mg, 0.184 mmol, 92%) as a colorless oil that solidified upon standing.

[α] ^{25} $_D$ = - 17.0 (*c* = 1.0, $CHCl_3$).

Rf = 0.89 (10 % EtOAc/cyclohexane).

^{1}H NMR (300 MHz, $CDCl_3$) δ 5.75-5.68 (m, 2H), 5.32 (m, 1H), 4.98-4.88 (m, 2H), 2.37 (sep, J = 6.8 Hz, 1H), 2.18-2.09 (m, 2H), 2.04-1.93 (m, 4H), 1.88-1.79 (m, 2H), 1.73-1.49 (m, 6H), 1.46-1.24 (m, 7H), 1.22-1.03 (m, 10H), 1.01-0.97 (m, 4H), 0.94-0.91 (m, 6H), 0.88-0.86 (m, 7H).

^{13}C NMR (126 MHz, CDCl3) δ 144.6, 142.1, 135.4, 125.9, 121.7, 112.5, 57.3, 56.4, 48.6, 45.5, 42.7, 40.1, 39.8, 36.5, 36.3, 36.1, 35.1, 34.5, 32.1, 32.1, 28.5, 28.3, 26.6, 24.1, 23.1, 22.8, 21.4, 20.4, 19.1, 19.0, 12.3.

Spectra

HRMS (APCI): calculated for $C_{32}H_{52}$ $[M]^+$: 436.4069; found: 436.4064.

(*2S,8S,8aR*)-8,8a-Dimethyl-6-((*S*)-2-methylbut-3-en-1-yl)-2-(prop-1-en-2-yl)-1,2,3,7,8,8a-hexahydronaphthalene (**4g**)

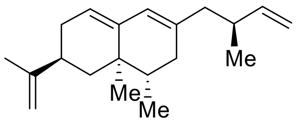

From cyclopropyl methyl boronic ester **1a** (58.8 mg, 0.30 mmol) and (4S,4aR,6S)-4,4a-dimethyl-6-(prop-1-en-2-yl)-3,4,4a,5,6,7-hexahydronaphthalen-2-yl trifluoromethanesulfonate (70.1 mg, 0.20 mmol), following the general procedure described above ($Pd(OAc)_2$/CPhos), compound **4g** was obtained. Purification by flash column chromatography and pTLC (n-hexane/EtOAc, 9:1) afforded **4g** (45.4 mg, 0.168 mmol, 83%) as a colorless oil.

[α] ^{25} $_{D}$ = + 34.4 (c = 1.0, $CHCl_3$).

Rf = 0.61 (10 % EtOAc/cyclohexane).

^{1}H NMR (300 MHz, $CDCl_3$) δ 5.77-5.65 (m, 2H), 5.36 (dd, J = 5.2, 2.7 Hz, 1H), 5.03-4.85 (m, 2H), 4.79-4.71 (m, 2H), 2.51-2.15 (m, 3H), 2.09-1.84 (m, 5H), 2.04-1.93 (m, 4H), 1.76 (s, 3H), 1.63-1.47 (m, 1H), 0.99 (d, J = 6.7 Hz, 3H), 0.89 (d, J = 6.8 Hz, 3H), 0.87 (s, 3H).

^{13}C NMR (76 MHz, CDCl3) δ 150.7, 144.5, 142.7, 136.3, 125.6, 121.2, 112.6, 108.8, 45.3, 40.4, 39.4, 37.6, 36.2 (2C), 35.9, 31.4, 30.0, 21.0, 20.4, 17.6, 15.0.

Spectra

HRMS (APCI): calculated for $C_{20}H_{30}$ $[M]^+$: 270.2347; found: 270.2302.

4-((*S*)-2-(Cyclopropylmethyl)but-3-en-1-yl)-1,2,3,6-tetrahydro-1,1'-biphenyl (**4h**)

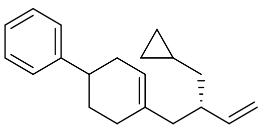

From cyclopropyl methyl boronic ester **1b** (48.0 mg, 0.20 mmol) and 1,2,3,6-tetrahydro-[1,1'-biphenyl]-4-yl trifluoromethanesulfonate (46.0 mg, 0.15 mmol), following the general procedure described above ($Pd(OAc)_2$/CPhos), compound **4h** was obtained as a mixture of diastereoisomers. Purification by flash column chromatography and pTLC (n-hexane/EtOAc, 9:1) afforded **4h** (28.9 mg, 0.11 mmol, 73%) as a colorless oil.

[α] ^{25} $_D$ = + 8.4 (*c* = 1.0, $CHCl_3$).

Rf = 0.88 (10 % EtOAc/cyclohexane).

^{1}H NMR (300 MHz, $CDCl_3$) δ 7.50-7.05 (m, 1H), 5.69 (dddd, *J* = 17.2, 10.2, 8.4, 4.8 Hz, 1H), 5.48 (d, *J* = 4.0, 1H), 5.09-4.93 (m, 2H), 2.74 (m, 1H), 2.31 (m, 2H), 2.22-1.86 (m, 7H), 1.83-1.64 (m, 2H), 1.40-1.24 (m, 2H), 1.21-1.02 (m, 1H), 0.72 (m, 1H), 0.46-0.41 (m, 2H), 0.10 – -0.05 (m, 2H).

^{13}C NMR (126 MHz, CDCl3) δ 147.5, 143.7, 143.5, 136.4, 136.3, 128.6, 128.5, 127.2, 127.2, 126.1, 122.6, 122.4 (2C), 113.8, 43.8(2C), 42.7, 42.6, 40.4, 40.3 (2C), 40.0, 33.7, 30.3 (2C), 29.2, 29.1, 9.2, 5.20, 5.10, 4.83, 4.79.

Spectra

HRMS (APCI): calculated for $C_{20}H_{26}$ $[M]^+$: 266.2034; found: 266.2028.

4-((*S*)-2-Methylbut-3-en-1-yl)-1,2,3,6-tetrahydro-1,1'-biphenyl (**4i**)

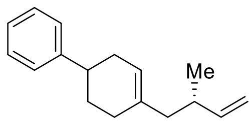

From cyclopropyl methyl boronic ester **1a** (58.8 mg, 0.30 mmol) and 1,2,3,6-tetrahydro-[1,1'-biphenyl]-4-yl trifluoromethanesulfonate (61.3 mg, 0.20 mmol), following the general procedure described above ($Pd(OAc)_2$/CPhos), compound **4i** was obtained as a mixture of diastereoisomers. Purification by flash column chromatography and pTLC (n-hexane/EtOAc, 9:1) afforded **4i** (45.0 mg, 0.199 mmol, 99%) as a colorless oil.

[α] ^{25} $_D$ = - 8.4 (*c* = 1.0, $CHCl_3$).

Rf = 0.88 (10 % EtOAc/cyclohexane).

^{1}H NMR (300 MHz, $CDCl_3$) δ 7.31-7.14 (m, 5H), 5.81-5.68 (m, 1H), 5.49-5.45 (brm, 1H), 5.01-4.89 (m, 2H), 2.79-2.68 (m, 1H), 2.38-2.24 (m, 2H), 2.19-1.89 (m, 6H), 1.81-1.67 (m, 1H), 0.97 (d, J = 7 Hz, 3H).

^{13}C NMR (76 MHz, $CDCl_3$) δ 147.4, 144.9, 144.7, 136.2, 136.2, 130.7, 128.4, 127.1, 127.0, 126.0, 122.5, 122.5, 112.3, 112.2, 77.2, 45.5, 45.3, 40.3, 35.7, 35.6, 33.7, 33.7, 30.3, 30.2, 29.0, 19.9, 19.6.

Spectra

HRMS (APCI): calculated for $C_{17}H_{22}$ $[M]^+$: 226.1722; found: 226.1717.

tert-butyl (*S*)-3-(5-phenylhexa-1,5-dien-3-yl)azetidine-1-carboxylate (**4j**)

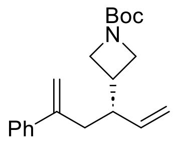

From cyclopropyl methyl boronic ester **1h** (101 mg, 0.30 mmol) and (1-bromovinyl)benzene (36.6 mg, 0.20 mmol), following the general procedure described above ($Pd(OAc)_2$, CPhos), compound **4j** was obtained. Purification by flash column chromatography and pTLC (n-hexane/EtOAc, 9:1) afforded **4j** (43.9 mg, 0.140 mmol, 69%) as a colorless oil.

[α] ^{25} $_D$ = - 24.9 (*c* = 1.0, $CHCl_3$).

Rf = 0.24 (10 % EtOAc/cyclohexane).

^{1}H NMR (300 MHz, $CDCl_3$) δ 7.29-7.21 (m, 5H), 5.44 (ddd, *J* = 17.1, 10.3, 8.2 Hz, 1H), 5.20 (d, *J* = 1.6 Hz, 1H), 4.95 (dd, *J* = 10.3, 1.7 Hz, 2H), 4.81 (dd, *J* = 17.1, 1.0 Hz, 1H), 3.82 (dt, *J* = 29.8, 8.5 Hz, 2H), 3.57-3.38 (m, 2H), 2.56 – 2.16 (m, 4H), 1.36 (s, 9H).

^{13}C NMR (126 MHz, $CDCl_3$) δ 156.5, 146.3, 141.1, 138.4, 128.6, 127.7, 126.5, 114.9, 79.4, 46.2, 39.1, 32.7, 28.6.

Spectra

HRMS (APCI): calculated for $C_{20}H_{27}NO_2$ $[M]^+$: 313.2042; found: 313.2036.

5.4. Synthesis of Rottnestol fragment

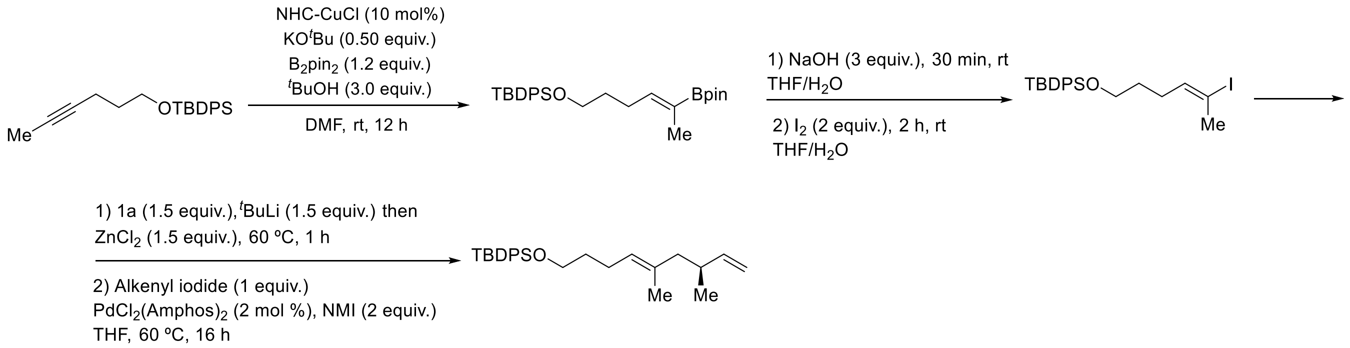

tert-butyl(pent-4-yn-1-yloxy)diphenylsilane (**S2**)

Compound **S2** was prepared according to literature procedure. [12]

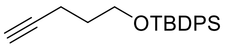

Pent-4-yn-1-ol (2.00 g, 23.8 mmol, 1 equiv.) and 1H-imidazole (4.86 g, 71.3 mmol, 3 equiv.) were dissolved in DMF (0.2 M) and cooled to 0 ºC. Then, tert-butylchlorodiphenylsilane (7.84 g, 28.5 mmol, 1.2 equiv.) was added dropwise and the reaction mixture was stirred for 16 h at room temperature. After this time, water (100 mL) was added and the mixture was extracted 3 times with Et_2O (3x50 mL), dried over $MgSO_4$ and evaporated under vacuum to obtain the product (7.3 g, 23 mmol, 95%) pure enough to follow up in the next reaction.

tert-butyl(hex-4-yn-1-yloxy)diphenylsilane (**6**)

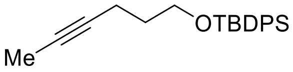

Compound **6** was prepared according to literature procedure. [13]

tert-butyl(pent-4-yn-1-yloxy)diphenylsilane (3.00 g, 9.30 mmol, 1 equiv.) was dissolved in THF (0.20 M). The solution was cooled to 0 ºC and n-BuLi (4.46 mL, 11.2 mmol, 1.2 equiv.) was added dropwise. After 30 minutes at this temperature, methyl iodide (14 mmol, 1.5 equiv.) was added and the reaction was stirred for 1 h. Then, sat. NH_4Cl (100 mL) was added and extracted with Et_2O (3x50 mL). The organic phases were dried over $MgSO_4$

and evaporated under vacuum. The crude product was purified by column chromatography (n-Hexane:EtOAc, 20:1) to give the compound S5 (2.0 g, 6.0 mmol, 65%) as a colorless oil.

Hydroiodination of internal alkyne

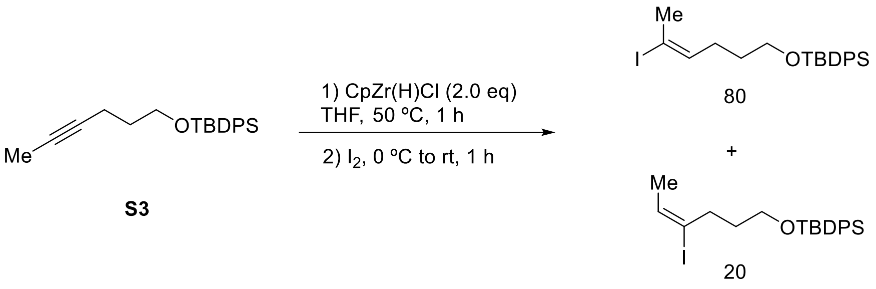

Typical hydrozirconation/iodination on this substrate gave the product in 4:1 regiomeric ratio. After extensive experimentation we reoptimized the copper-catalyzed carboboration conditions reported by Kanai [14] to achieve a regioselective protoborylation. We found that the proton source played a key role in the regioselectivity with tBuOH being the optimal proton source.

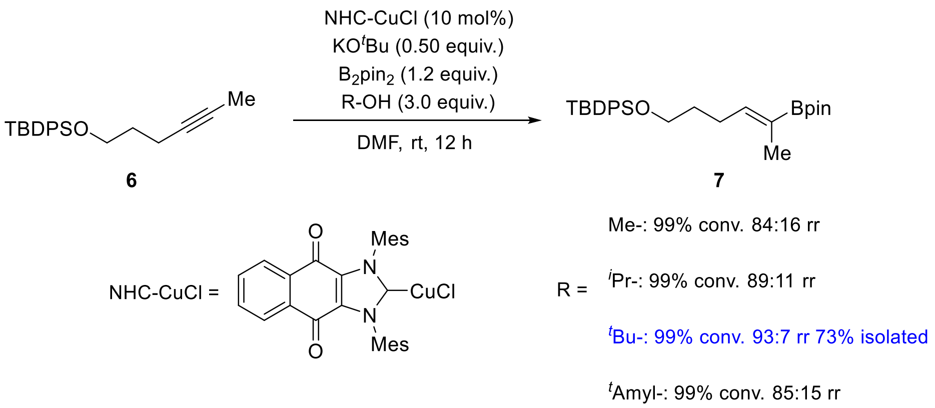

Procedure for the borylation:

In a glove box, NHC-CuCl (160 mg, 0.297 mmol, 0.10 equiv.), ^{t}BuOK (167 mg, 1.49 mmol, 0.50 equiv.), and B_2pin_2 (905 mg, 3.60 mmol, 1.20 equiv.) were added to a sealed vial and DMF (3 mL) were added. The vial was brought out of the glove box and a solution of alkyne **6** (1.00 g, 2.97 mmol, 1 equiv.) and tBuOH (0.85 mL, 8.91 mmol, 3.0 equiv.) in DMF (3mL) was added dropwise. The mixture was stirred overnight at rt. Then, water was added to the mixture and extracted with Et_2O (3x20mL). The combined organic phases were washed 2 times with brine solution and dried over $MgSO_4$. Solvent was evaporated and crude product (93:7 rr) was purified by column chromatography (*n*-Hex:EtOAc, 95:5) to furnish pure boronic ester **7**.

^{1}H NMR (500 MHz, $CDCl_3$) δ 7.54 (m, 5H), 7.30 – 7.21 (m, 5H), 6.21 (m, 1H), 3.55 (t, *J* = 6.3 Hz, 2H), 2.10 (m, 2H), 1.55 – 1.51 (m, 2H), 1.14 (s, 12H), 0.92 (s, 9H).

^{13}**C NMR** (126 MHz, $CDCl_3$) δ 146.1 (2C), 135.7 (4C), 134.2, 129.6 (2C), 127.7 (4C), 83.2, 63.7, 31.9, 27.0, 25.2, 24.9, 19.3, 14.0.

^{11}**B NMR** (160 MHz, $CDCl_3$) δ 30.1.

Spectra

Iodination:

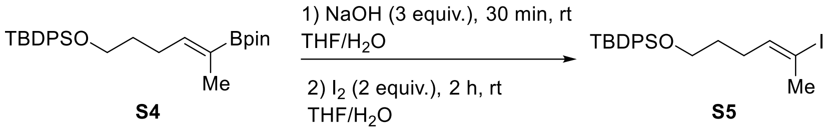

Alkenyl boronic ester **7** (818 mg, 1.76 mmol, 1 equiv.) was dissolved in THF (9 mL) and 1.76 mL of 3M NaOH (3 equiv.) were added. The mixture was stirred 30 min at rt and then a solution of I_2 (894 mg, 3.52 mmol, 2 equiv.) in THF (9 mL) was added dropwise. The mixture was stirred for 2 h at rt and then quenched with saturated sodium sulfite solution and extracted with Et_2O (3x20 mL). Solvent evaporated and crude product was purified by column chromatography (n-Hex:EtOAc, 95:5) to furnish pure product **8** as a colorless oil (410 mg, 0.88 mmol, 50%, 98:2 rr).

^{1}**H NMR** (300 MHz, $CDCl_3$) δ 7.68-7.65 (m, 4H), 7.44-7.36 (m, 6H), 6.14 (m, 1H), 3.66 (t, *J* = 6.1 Hz, 2H), 2.37-2.36 (m, 3H), 2.19-2.11 (m, 2H), 1.61 (m, 2H), 1.06 (s, 9H).

^{13}**C NMR** (75 MHz, $CDCl_3$) δ 141.1, 135.9, 135.8, 134.1, 129.9, 127.9, 94.2, 63.1, 31.9, 27.7, 27.2, 27.1, 19.5.

Spectra

Negishi coupling:

(*S,E*)-*tert*-butyl((5,7-dimethylnona-4,8-dien-1-yl)oxy)diphenylsilane (**4k**)

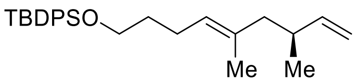

From cyclopropyl methyl boronic ester **1a** (101.3 mg, 0.52 mmol) and (*E*)-*tert*-butyl((5-iodohex-4-en-1-yl)oxy)diphenylsilane **8** (160.0 mg, 0.35 mmol), following the general procedure described above ($PdCl_2Amphos_2$/NMI), compound **4k** was obtained. Purification by flash column chromatography and pTLC (n-hexane/EtOAc, 9:1) afforded **4k** (85.0 mg, 0.210 mmol, 60%) as a colorless oil. The product contained a small impurity of EtOTBDPS, therefore it was treated with TBAF to isolate pure alcohol for characterization.

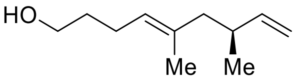

[α] $^{25}_{D}$ = + 3.9 (*c* = 0.4, $CHCl_3$).

^{1}**H NMR** (300 MHz, $CDCl_3$) δ 5.71 (m, 1H), 5.16-5.11 (m, 1H), 4.98-4.87 (m, 2H), 3.64 (m, 2H), 2.36-2.26 (m, 1H), 2.12-1.99 (m, 3H), 1.94-1.87 (m, 1H), 1.67 – 1.57 (m, 5H), 0.95 (d, *J* = 6.7 Hz, 3H).
^{13}**C NMR** (75 MHz, CDCl3) δ 144.8, 134.5, 125.9, 112.4, 63.0, 47.5, 35.8, 33.0, 24.0, 19.8, 16.2.

Spectra

HRMS (APCI): calculated for $C_{11}H_{20}O$ $[M]^+$: 168.1514; found: 168.1506.

6. Mechanistic experiments by Nuclear Magnetic Resonance studies

To get insight into the ate-complex formation and the transmetallation step we performed ^1H and ^{11}B NMR experiments of each of these steps.

6.1. Boron-ate complex Formation and Characterization

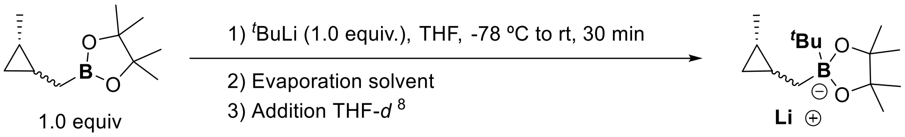

In an oven-dried Schlenk, 2-(cyclopropylmethyl)-4,4,5,5-tetramethyl-1,3,2-dioxaborolane **1a** (36.4 mg, 0.2 mmol, 1.0 equiv.) was weighed and vacuum-argon cycles were done. Under argon atmosphere, 0.2 mL of anhydrous THF were added, the solution was cooled to -78 °C (dry ice/acetone bath) and *tert*-butyllithium (1.7 M in pentane, 1.0 equiv.) was added dropwise by a syringe. Then, the reaction mixture was stirred while warming to room temperature for 30 min. After this reaction time, the solvent was evaporated and when the reaction is totally dried, the Schlenk was introduced in the glovebox and the reaction was transferred with THF-d^8 to a *J. Young* tube.

^{1}H NMR (500 MHz, THF-d^{8}) of the boron-ate complex

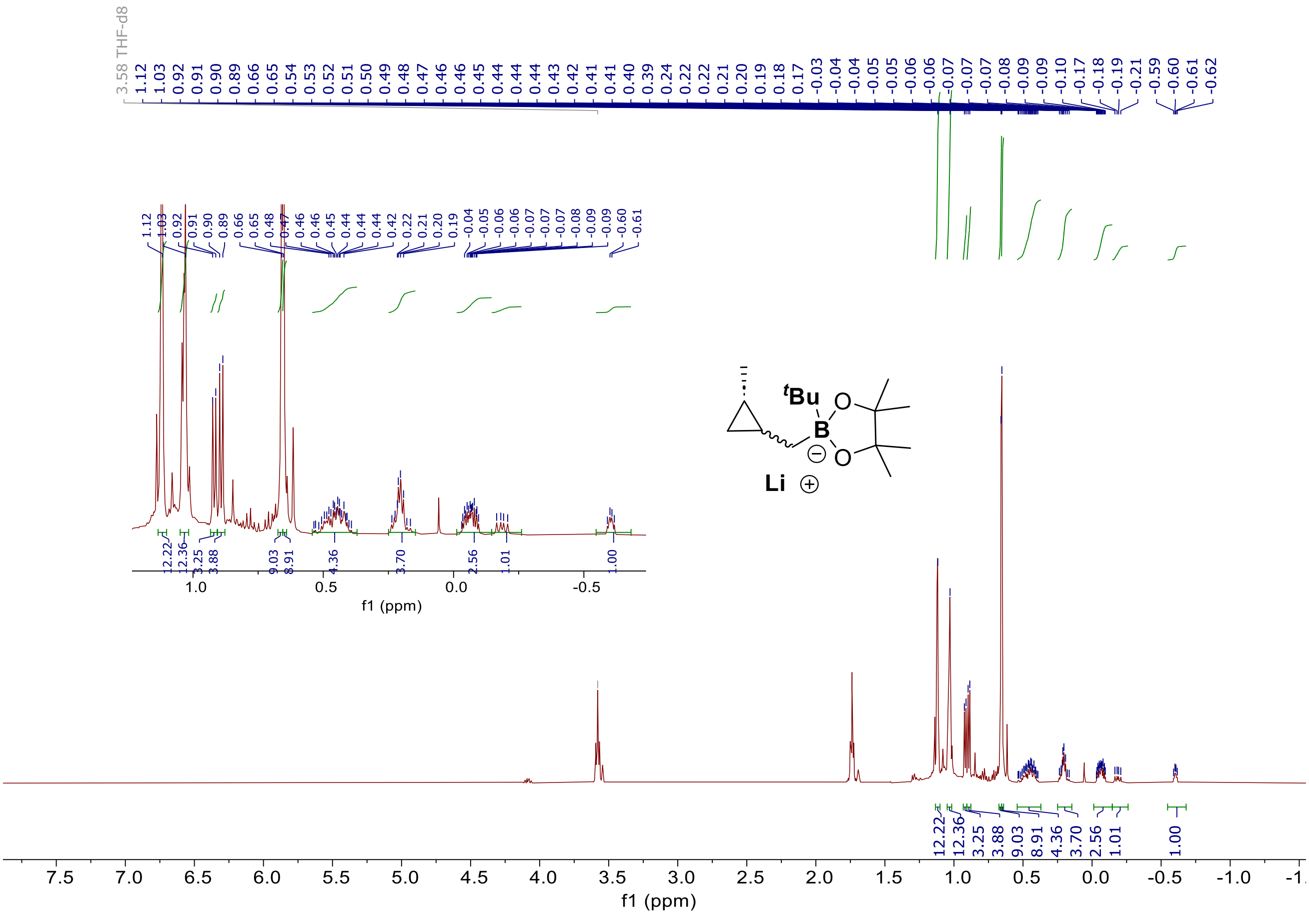

^{1}H NMR (500 MHz, THF-d^{8}) δ 1.12 (s, 9H), 1.03 (s, 9H), 0.92 (d, J = 5.9 Hz, 3H), 0.89 (d, J = 6.0 Hz, 3H), 0.66 (s, 12H), 0.65 (s, 12H), 0.54 – 0.37 (m, 5H), 0.21 (m, 3H), -0.07 (m, 2H), -0.19 (dd, J = 13.3, 7.9 Hz, 1H), -0.58 – -0.64 (m, 1H).

^{13}C NMR (126 MHz, THF-d^{8}) of the boron-ate complex

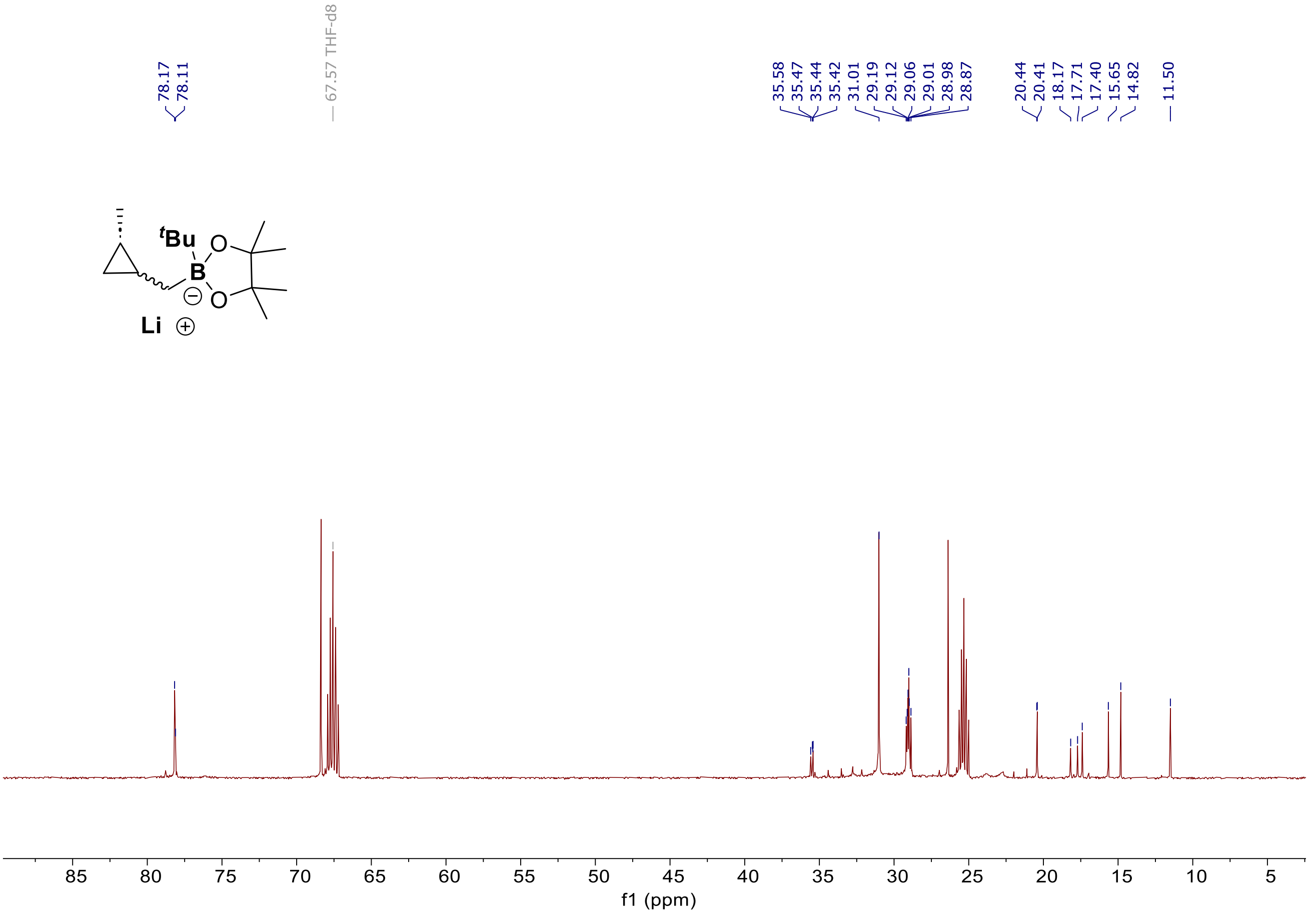

^{13}C NMR (126 MHz, THF-d^{8}) δ 78.2 (2C), 78.1 (2C), 35.6, 35.5, 35.4, 35.4, 31.0 (6C), 29.2, 29.1, 29.1, 29.0, 29.0, 28.9, 20.4, 20.4, 18.2, 17.7, 17.4, 15.6, 14.8, 11.5.

^{11}B NMR (160 MHz, THF-d^8) of the boron-ate complex

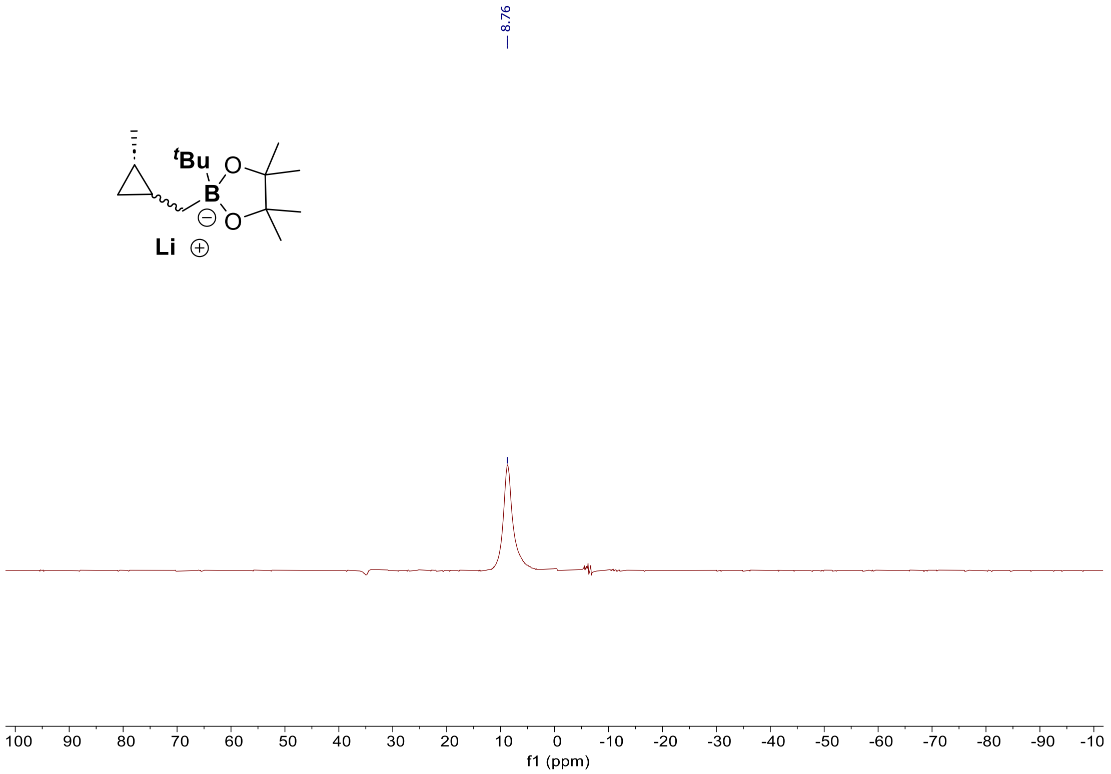

^{11}B NMR (160 MHz, THF-d^8) δ 8.76.

COSY (500 MHz, THF-d^8) of the boron-ate complex

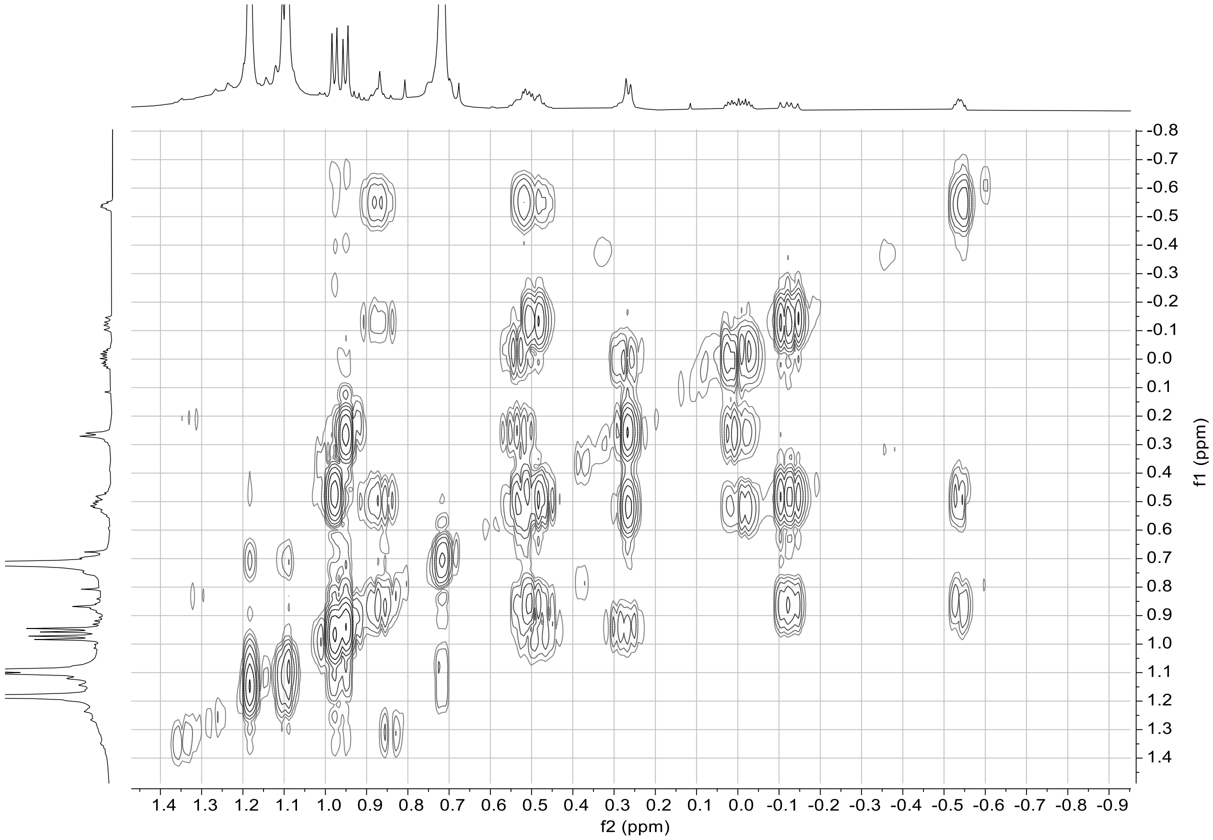

6.2. Boron to Zinc transmetallation at different temperatures

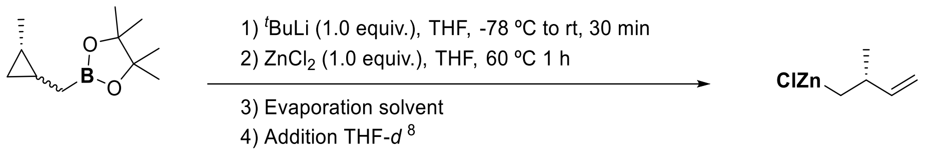

In an oven-dried Schlenk, 2-(cyclopropylmethyl)-4,4,5,5-tetramethyl-1,3,2-dioxaborolane (36.4 mg, 0.2 mmol, 1.0 equiv.) was weighed and vacuum-argon cycles were done. Under argon atmosphere, 0.2 mL of anhydrous THF were added, the solution was cooled to -78 °C (dry ice/acetone bath) and *tert*-butyllithium (1.7 M in pentane, 1.0 equiv.) was added dropwise by a syringe. Then, the reaction mixture was stirred while warming to room temperature for 30 min. After this time, the solvent was evaporated in vacuo and the Schlenk was introduced in the glovebox and the reaction was transferred with THF-d^8 to a *J. Young* tube. Then, 0.5 equiv of benzene as internal standard and zinc chloride (1.0 equiv.) was added as a solid to the mixture in the glovebox and the reaction was analyzed by nuclear magnetic resonance.

6.2.1. Transmetalation at 60 °C

After addition of the $ZnCl_2$, the reaction mixture was heated at 60 °C. The ^{1}H NMR spectrum showed complete conversion of the boron-ate complex to the homoallylzinc intermediate (86-98% yield, 0.5 equiv of benzene as internal standard). The *J. Young* tube was maintained during one hour at 60 °C and it remained stable.

^{1}H NMR (300 MHz, THF-d^8)

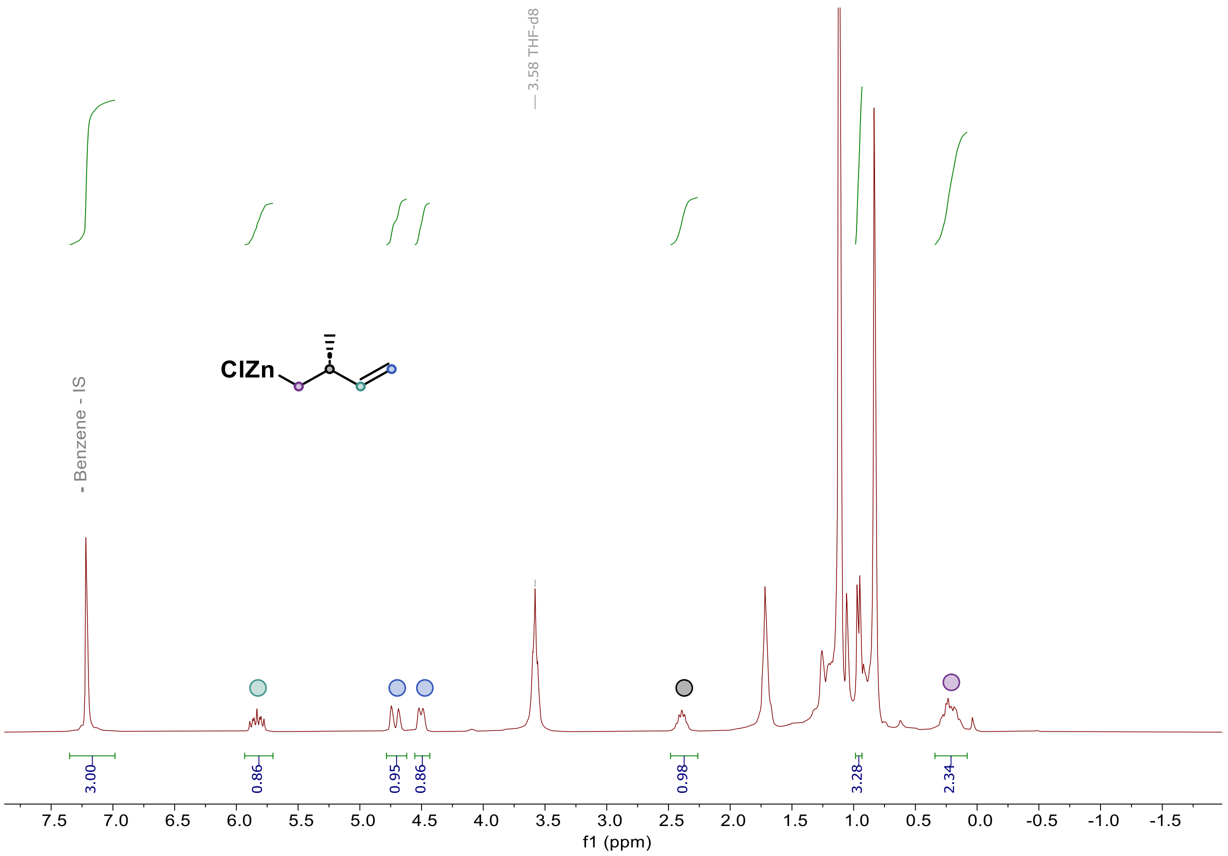

6.2.2. Transmetalation at Room Temperature

After addition of the $ZnCl_2$, the reaction mixture was maintained at 25 °C. ^{11}B NMR was recorded after 5 min showing complete boron to zinc transmetalation. ^{1}H NMR were recorded every 5 min for an hour. The ^{1}H NMR spectra after 5 min showed a mixture of compounds difficult to analyze. We could already observed signals corresponding to the homoallylzinc species indicating that the transmetalation-ring opening is taking place at room temperature. Complete conversion to the homoallylzinc intermediate was observed overnight.

^{11}B NMR (160 MHz, THF-d^{8}) of the ate complex and the ^{t}BuBpin which is the by-product of the reaction and indicates the end of the transmetalation process.

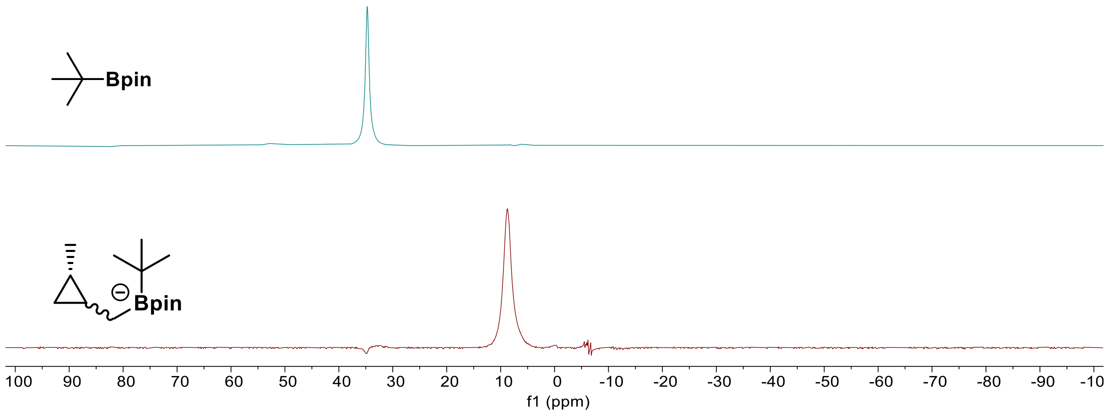

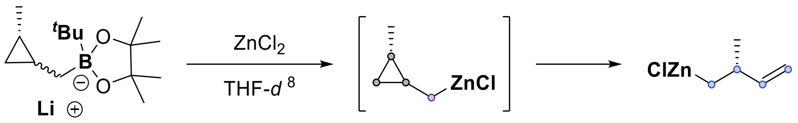

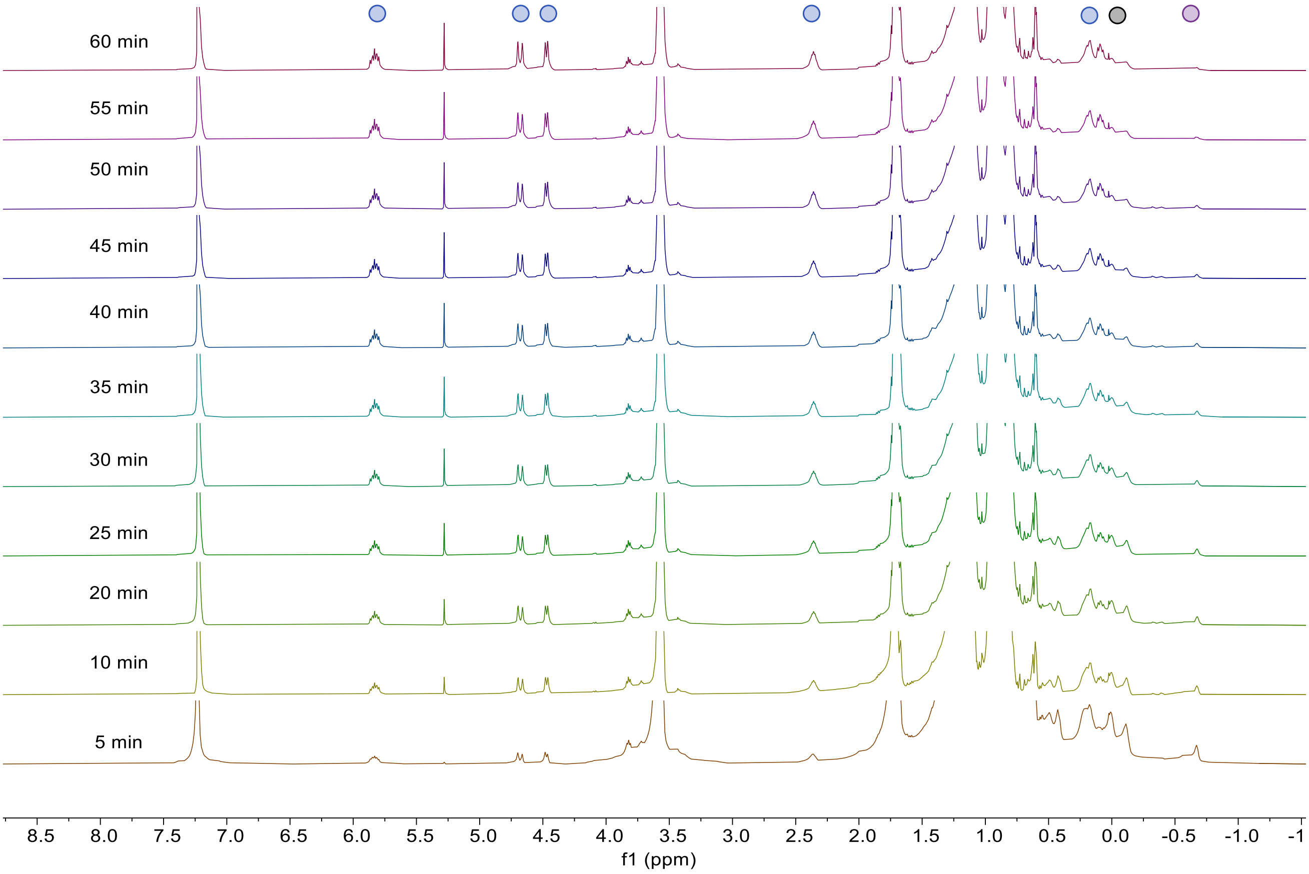

Plot of homoallylzinc formation versus time, based on yields determined from ^{1}H NMR integrals using benzene as internal standard.

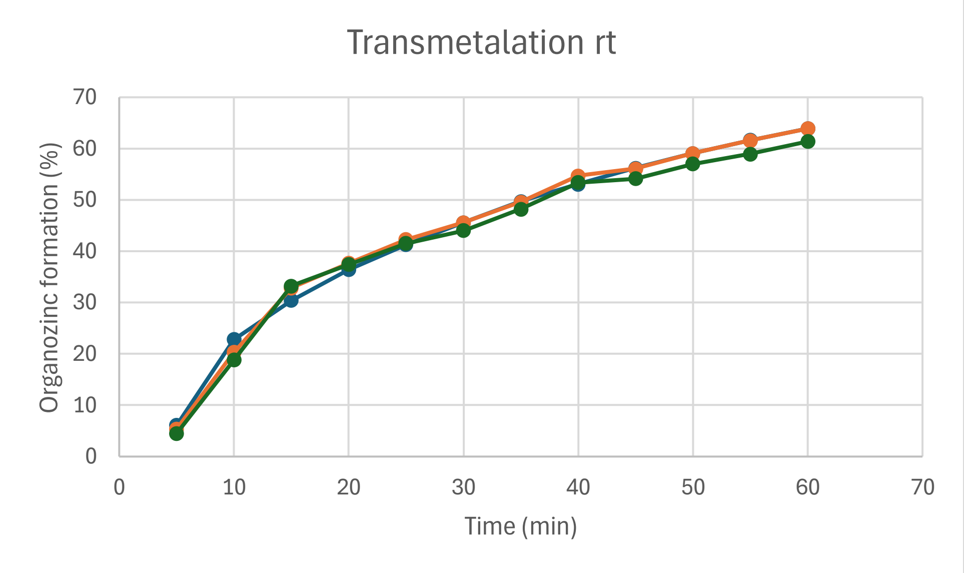

^{1}H NMR (300 MHz, THF-d^{8}) data collected after full conversion to the organozinc compound overnight.

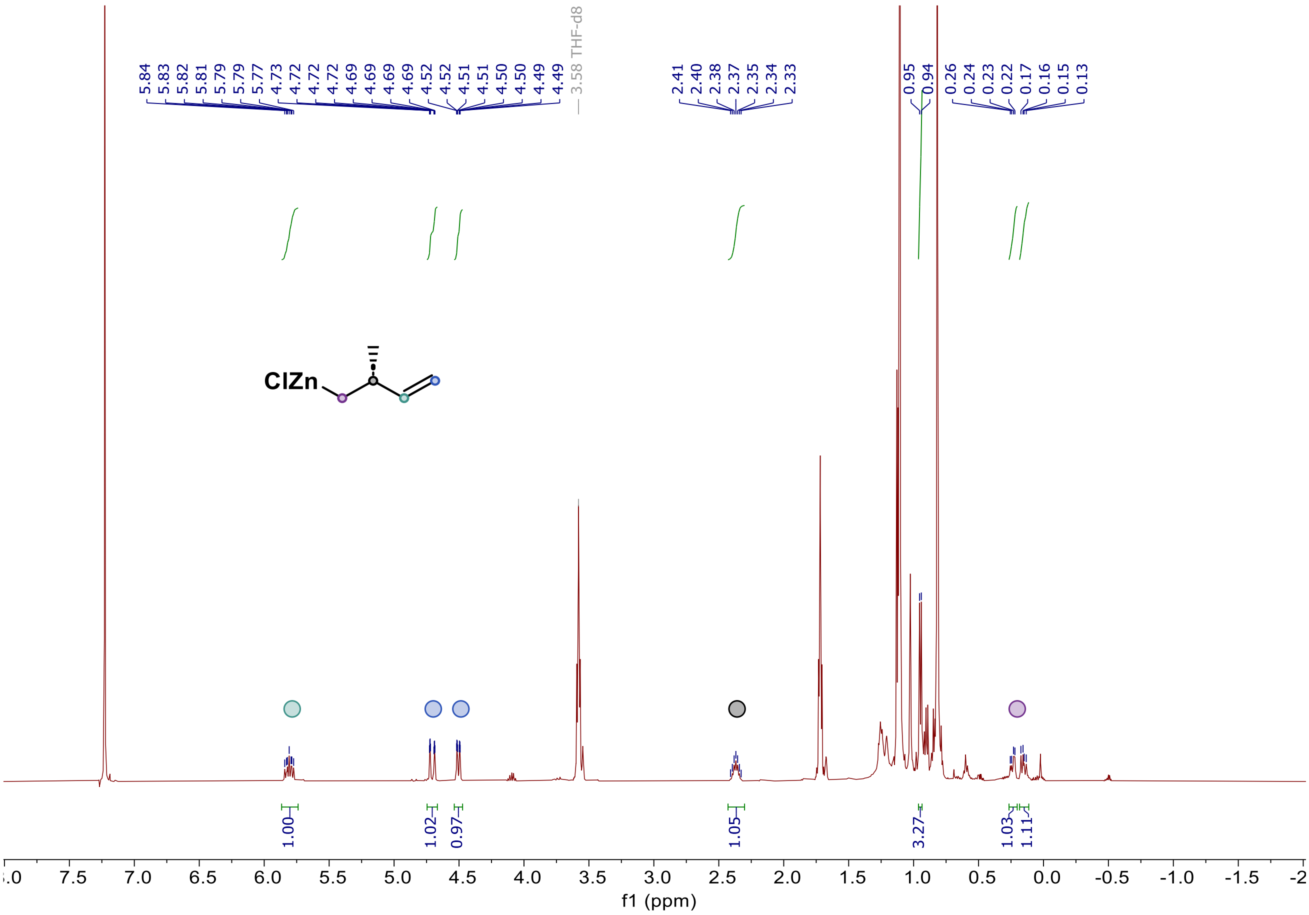

6.2.3. Transmetalation at Low Temperature

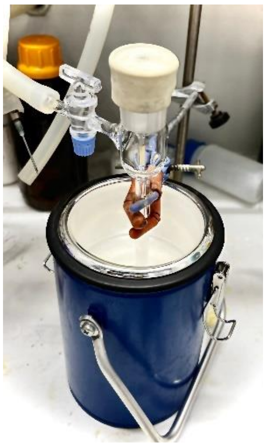

Starting from cyclopropylboronate **1a**, the boron-ate complex was formed and transferred to a *J. Young* tube in THF-d^{8}. The J. Young tube was connected to a Schlenk line and cooled to -78 °C. Then a solution of $ZnCl_2$ in THF-d^{8} was added. Then ^{11}B NMR and ^{1}H NMR spectra were recorded at -55 °C. The ^{11}B NMR showed almost complete boron to zinc transmetallation. The ^{1}H NMR at -55 °C only showed traces of olefinic protons which was indicative of having cyclopropylmethylzinc species in solution. ^{1}H NMR spectra were then recorded increasing gradually the temperature in 10 °C. We observed the peaks corresponding to the homoallyl zinc compound appearing gradually as the tenperature was increased. At 25 °C, we observed a 47% yield of the homoallylzinc intermediate with signals corresponding to ring-close products still present. This mixture of compounds evolved to a clean NMR spectrum of the homoallylzinc compound overnight.

^{11}B NMR (160 MHz, THF-d^{8}) at -55 °C

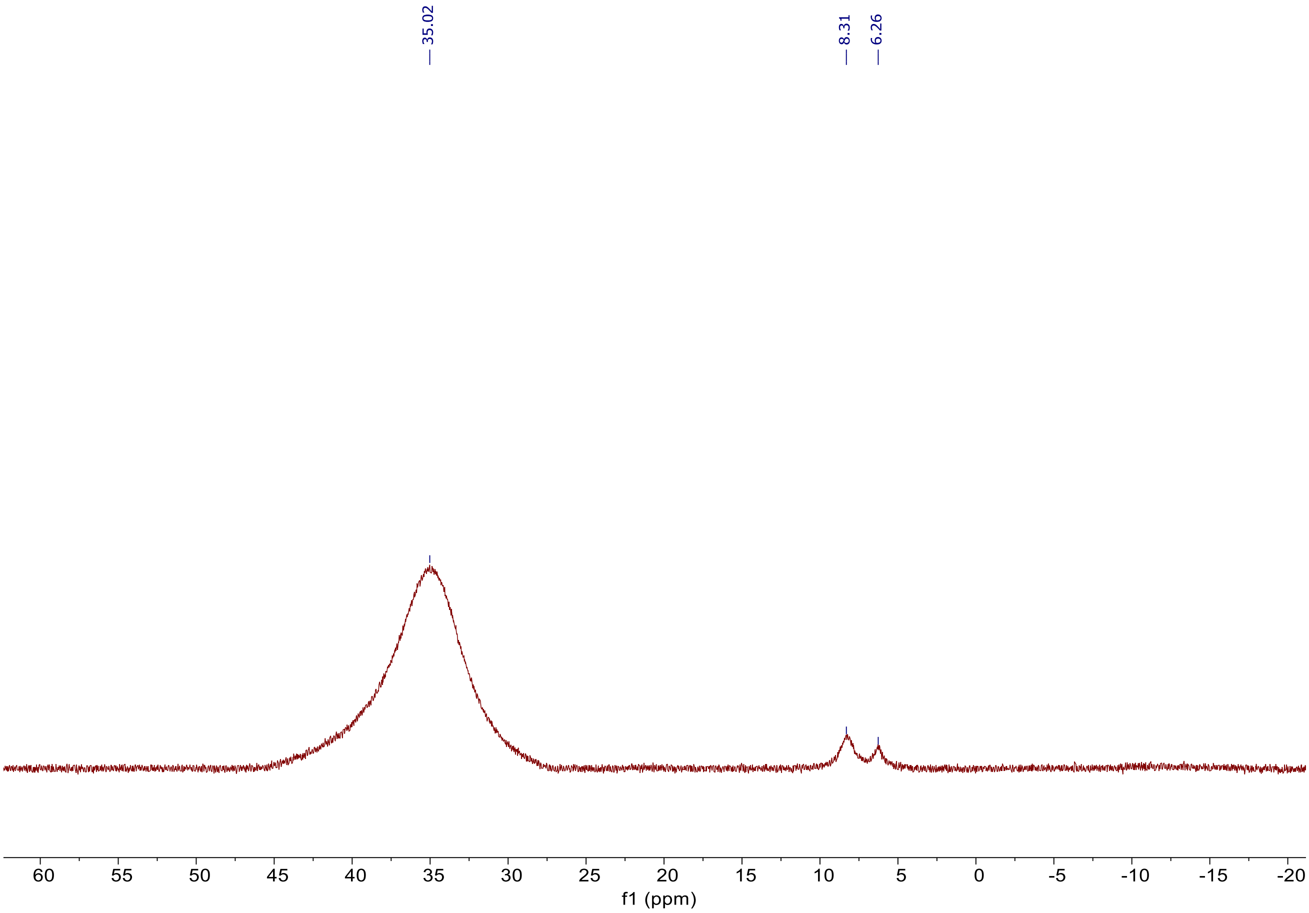

^{11}B NMR (160 MHz, THF-d^{8}) δ 35.02, 8.31, 6.26.

For the kinetic study, ^{1}H NMR spectra were acquired at 10 °C intervals during a temperature increase from –55 to 25 °C, over approximately 90 minutes.

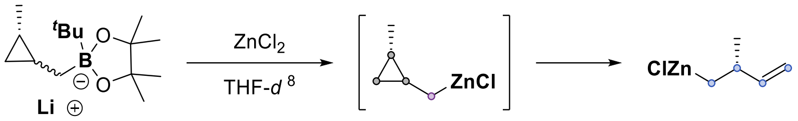

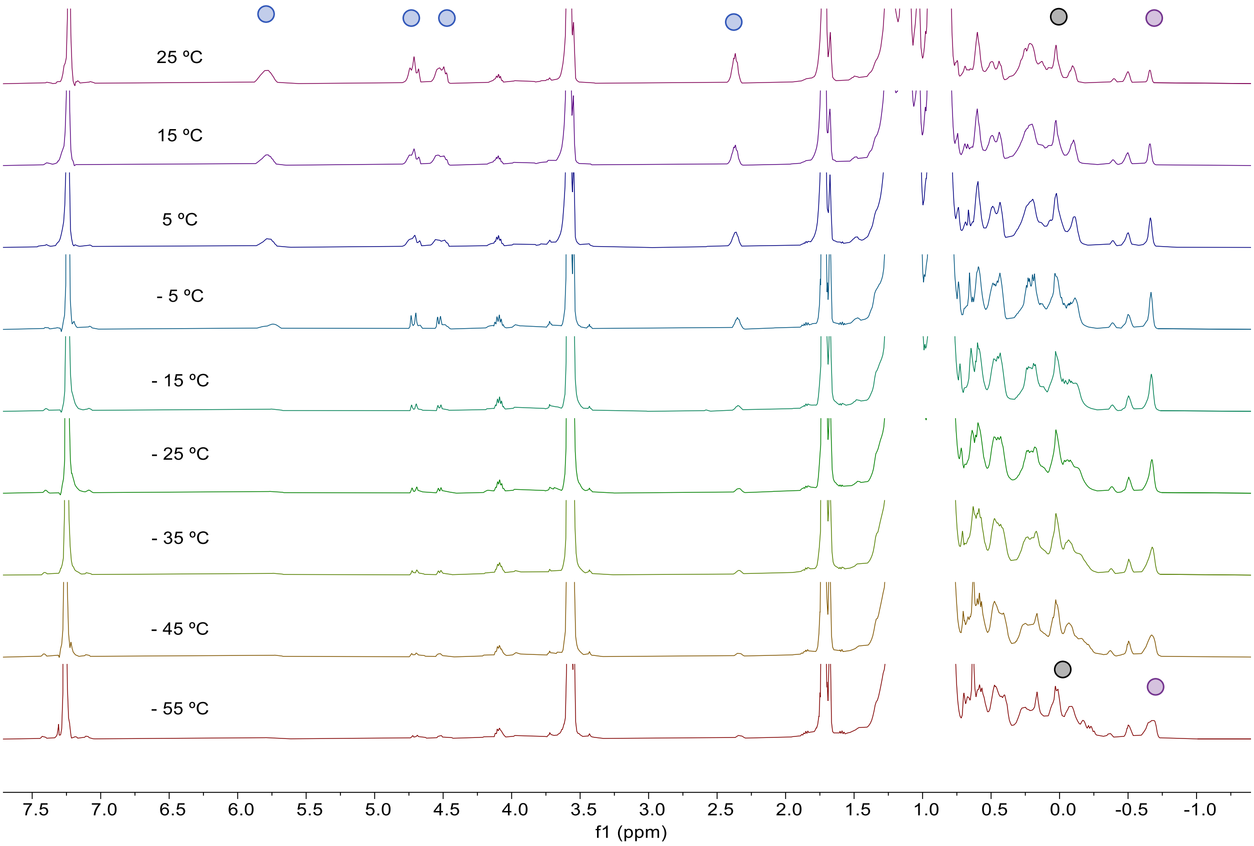

Plot of homoallylzinc formation versus temperature, based on yields determined from ^{1}H NMR integrals using benzene as internal standard.

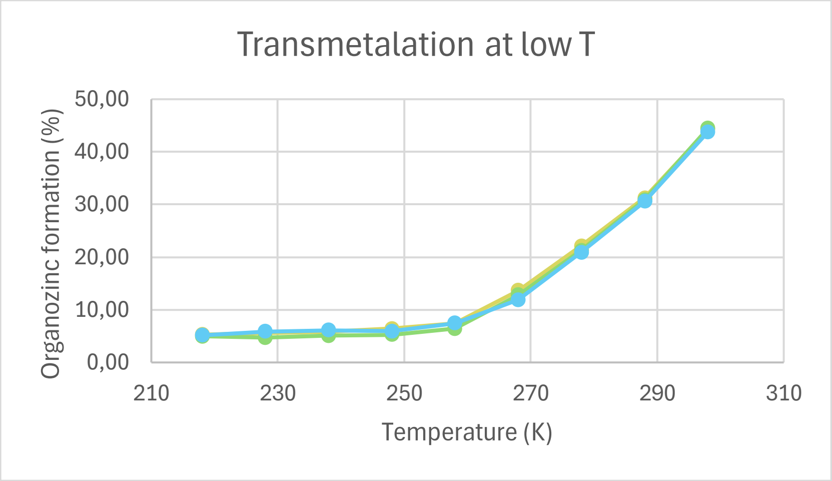

7. Absolute configuration of the cyclopropylmethyl boronates

Since the stereocenter remains untouched during the cross-coupling event, the absolute configuration of cyclopropylmethyl boronate **1a** was established from homoallylation products **3a**, **3b**, **3c** and **3i**. The absolute configuration of these products was determined by comparison of their optical rotation with those described in the literature.[8-10] These four correlations agreed on the (*S*) absolute configuration for cyclopropylboronate **1a**.

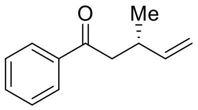

3a

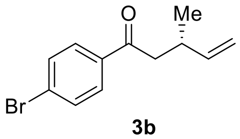

3b

	3a	3b
Experimental	$[\alpha]^{25}_{D}$ = - 19.7 (c = 1.0, $CHCl_3$).	$[\alpha]^{25}_{D}$ = - 18.2 (c = 1.0, $CHCl_3$).
Literature	$[\alpha]^{25}_{D}$ = - 2.7 (c = 1.0, $CHCl_3$).	$[\alpha]^{25}_{D}$ = - 5.55 (c = 1.0, $CHCl_3$).

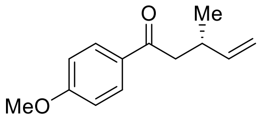

3c

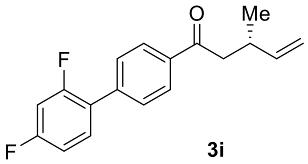

3i

	3c	3i
Experimental	$[\alpha]^{25}_{D}$ = - 17.2 (c = 1.0, $CHCl_3$).	$[\alpha]^{25}_{D}$ = - 12.2 (c = 1.0, $CHCl_3$).
Literature	$[\alpha]^{25}_{D}$ = - 3 (c = 1.0, $CHCl_3$).	$[\alpha]^{25}_{D}$ = - 4.95 (c = 1.0, $CHCl_3$).

This (*S*) configuration in **1a** implies a retention of the stereochemistry in the borylative cyclization.[15]

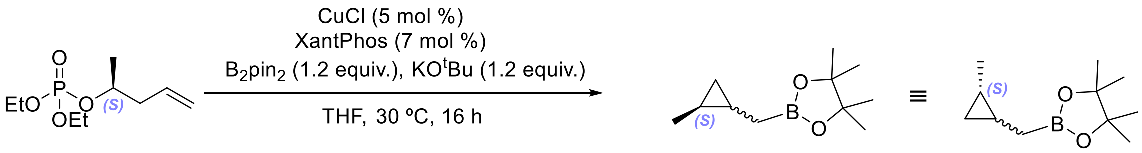

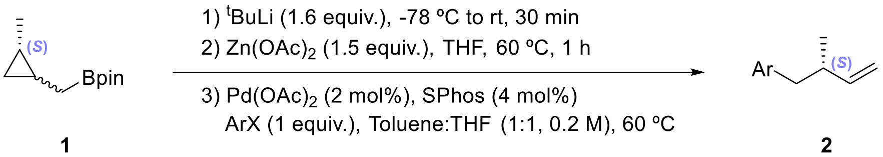

1
1.5 equiv

2

8. Proof of chirality transfer from the enantiopure allylic phosphate to the cross-coupling products

To prove the stereospecificity of the borylative cyclization and the enantiopurity of the cross-coupling products we isolated the enantioenriched phosphate **S6** and determined the enantiopurity by chiral HPLC (er = 99:1). Comparison of the enantiomeric ratio of the phosphate and that of alcohol **S7** (see below) proved that the borylative cyclization is stereospecific and, therefore, the chirality is transferred from the homoallylic alcohols to the cross-coupling products.

(*S*)-pent-4-en-2-yl diphenyl phosphate (S6)

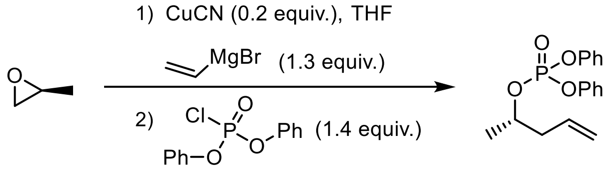

To a stirred solution of (*S*)-2-methyloxirane **S1a** (0.3 mL, 4.3 mmol, 1 equiv.) and CuCN (77 mg, 0.86 mmol, 0.20 equiv.) in dry THF (4.3 mL), was added over 45 min a 1 M solution of vinylmagnesium bromide in THF (5.6 mL, 5.6 mmol, 1.3 equiv.) dropwise at -78 °C. The mixture was allowed to warm up to rt and was stirred till it had finished. When the reaction was finished, diphenyl chloridophosphate (1.3 mL, 6.0 mmol, 1.4 equiv.) was added at 0 °C and stirred 1 h at rt. Finally, the reaction was quenched with a saturated NH_4Cl solution (5 mL). The layers were separated, the aqueous layer extracted with Et_2O (3 x 10 mL), the combined ethereal extracts were washed with brine (5 mL) and dried ($MgSO_4$) obtaining a pure enough crude product **S6** (1.4 g, 4.3 mmol, 100%).

[α] ^{25} $_{D}$ = -0.9 (*c* = 1.0, $CHCl_3$).

^{1}H NMR (300 MHz, $CDCl_3$) δ 7.39 – 7.29 (m, 4H), 7.25 – 7.19 (m, 5H), 7.19 – 7.13 (m, 1H), 5.85 – 5.60 (m, 1H), 5.13 – 5.07 (m, 1H), 5.05 (t, *J* = 1.3 Hz, 1H), 4.78 (dh, *J* = 7.3, 6.2 Hz, 1H), 2.53 – 2.28 (m, 2H), 1.35 (d, *J* = 6.3 Hz, 3H). **^{13}C NMR** (76 MHz, $CDCl_3$) δ 150.7 (d, *J* = 6.9 Hz), 132.7, 129.8 (4C), 125.3 (4C), 120.2 (d, *J* = 5.0 Hz), 118.6, 77.4 (d, *J* = 6.6 Hz), 41.6 (d, *J* = 5.8 Hz), 21.0 (d, *J* = 3.9 Hz). **^{31}P NMR** (121 MHz, $CDCl_3$) δ -12.17.

Spectra

HRMS (APCI): calculated for $C_{17}H_{19}O_4P$ $[M+H^+]^+$: 319.1099; found: 319.1094.

The enantiomeric ratio (99:1) was determined by chiral HPLC using Chiralpak-IBN column [hexane/i-PrOH (95:5)], 1 mL/min, τ_{major} = 4.0 min, τ_{minor} = 4.2 min.

HPLC chromatogram: Racemic phosphate

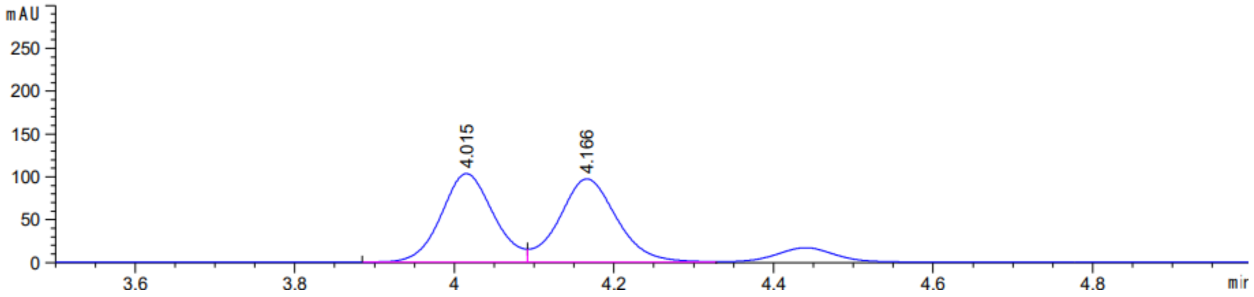

Peak #	RetTime [min]	Type	Width [min]	Area [mAU*s]	Height [mAU]	Area %
1	4.015	BV	0.0691	470.37668	103.26525	49.1897
2	4.166	VV	0.0758	485.87369	97.22414	50.8103

HPLC chromatogram: Enantioenriched phosphate

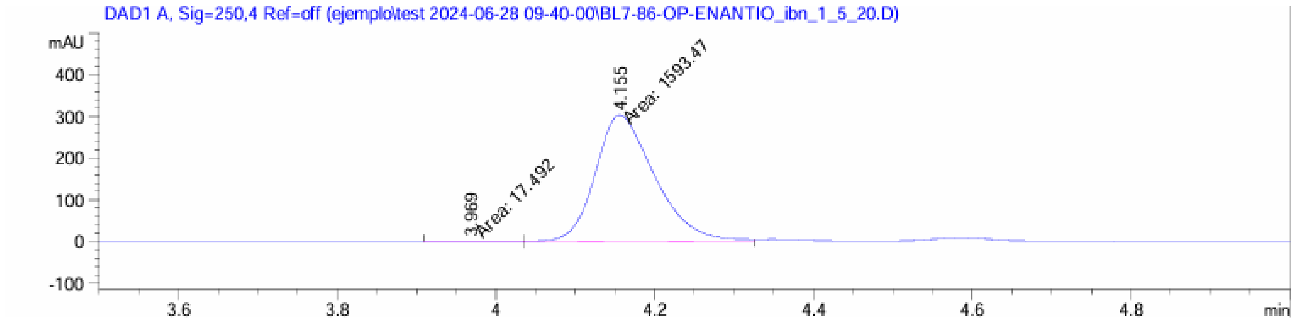

Peak #	RetTime [min]	Type	Width [min]	Area [mAU*s]	Height [mAU]	Area %
1	3.969	MF	0.0869	17.49204	2.38119	1.0858
2	4.155	FM	0.0873	1593.46704	304.11316	98.9142

methyl (*S*)-4-(4-hydroxy-2-methylbutyl)benzoate (**S7**)

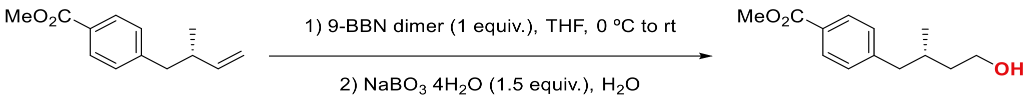

To an oven-dried 1-dram vial with a stir bar was added 9-BBN dimer (47.4 mg, 0.19 mmol, 1.0 equiv.) and THF (5 mL/mmol) under argon. The solution was cooled to 0 °C (ice bath) and a solution of methyl (*R*)-4-(2-methylbut-3-en-1-yl)benzoate **2k** (39.7 mg, 0.19 mmol, 1.0 equiv.) in THF (5 mL/mmol) was added slowly. The solution was warmed to rt and left stirring for 16 h. After full conversion, water (10 mL/mmol) and $NaBO_3$·4H2O (44.9 mg, 0.29 mmol, 1.5 equiv.) were added and the mixture was stirred for 3 h. The reaction was extracted with Et_2O and the combined organic layers were washed with brine, dried over $MgSO_4$ and the solvent was evaporated. The reaction crude was purified by flash column chromatography using a gradient of EtOAc in Cy as eluent obtaining the product **S7** as a colorless oil (33.3 mg, 0.15 mmol, 77%).

[α] ^{25} $_{\mathbf{D}}$ = -47.1 (c = 1.0, $CHCl_3$).

^{1}H NMR (300 MHz, $CDCl_3$) δ 8.01 – 7.89 (m, 2H), 7.26 – 7.16 (m, 2H), 3.89 (s, 3H), 3.79 – 3.58 (m, 2H), 2.70 (dd, *J* = 13.4, 6.3 Hz, 1H), 2.47 (dd, *J* = 13.4, 8.0 Hz, 1H), 2.05 – 1.83 (m, 1H), 1.63 (dddd, *J* = 12.4, 9.8, 7.2, 5.2 Hz, 1H), 1.56 – 1.32 (m, 1H), 0.88 (d, *J* = 6.7 Hz, 3H).

^{13}C NMR (75 MHz, $CDCl_3$) δ 167.3, 146.8, 129.7 (2C), 129.3 (2C), 128.0, 61.0, 52.1, 43.9, 39.6, 31.7, 19.5.

Spectra

HRMS (APCI): calculated for $C_{13}H_{19}O_3$ $[M+H^+]^+$: 223.1334; found: 223.1325.

The enantiomeric ratio (99:1) was determined by chiral HPLC using Chiralpak-IBN column [hexane/i-PrOH (95:5)], 1 mL/min, τ_{major} = 8.2 min, τ_{minor} = 9.1 min.

HPLC chromatogram: Racemic product

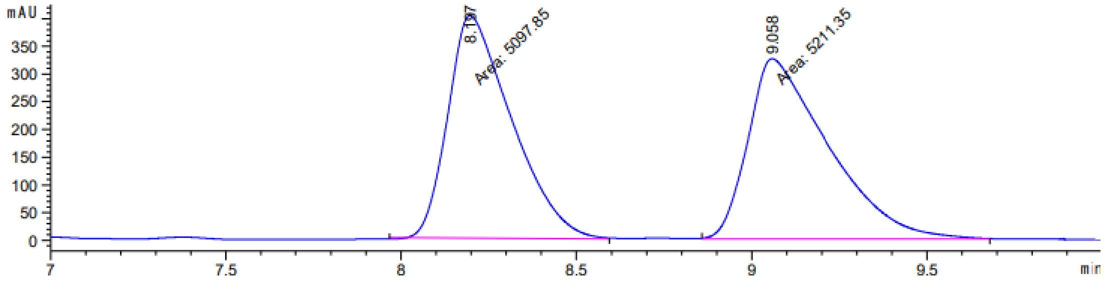

Peak #	RetTime [min]	Type	Width [min]	Area [mAU*s]	Height [mAU]	Area %
1	8.197	MM	0.2112	5097.85400	402.38058	49.4496
2	9.058	MM	0.2667	5211.34766	325.70239	50.5504

HPLC chromatogram: Enantioenriched product

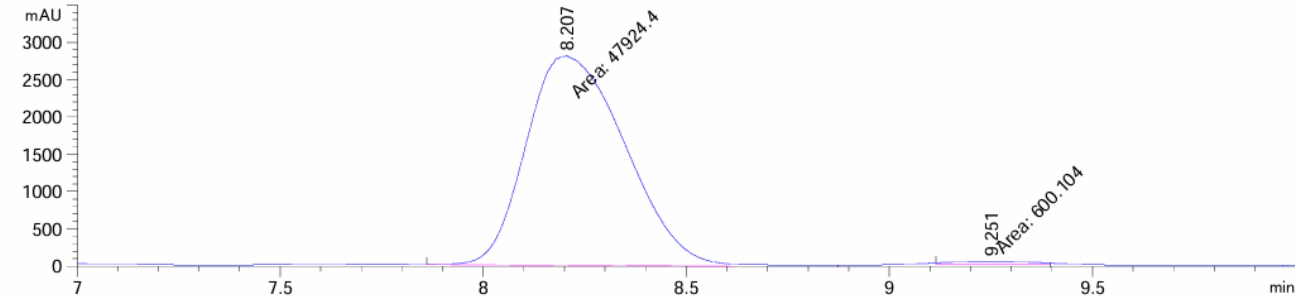

Peak #	RetTime [min]	Type	Width [min]	Area [mAU*s]	Height [mAU]	Area %
1	8.207	FM	0.2838	4.79244e4	2814.13745	98.7633
2	9.251	MM	0.2277	600.10437	43.93301	1.2367

9. Computational details

All the calculations reported in this paper were obtained with the Gaussian 16 suite of programs.[16] All species were optimized using the meta-hybrid functional M06L[17] in conjunction with standard double-ζ quality def2-SVP[18] basis sets for all atoms. Solvents effects (solvent = tetrahydrofuran) were taken into account during the geometry optimizations using the polarizable continuum model (PCM).[19] All stationary points were characterized by frequency calculations.[20] Reactants and products have positive definite Hessian matrices, whereas transition structures show only one negative eigenvalue in their diagonalized force constant matrices, and their associated eigenvectors were confirmed to correspond to the motion along the reaction coordinate under consideration using the intrinsic reaction coordinate (IRC) method.[21] Single-point energy refinements were carried out at the same DFT level, using the much larger triple-ζ quality def2-TZVPP basis sets. This level is denoted PCM-M06L/def2-TZVPP//PCM-M06L/def2-SVP. The computed thermochemistry data were corrected following Grimme's quasi-harmonic (QHA) model for entropy[22] with a frequency cutoff value of 100.0 cm^{-1} using the GoodVibes[23] program at 298.15 K and a standard concentration of 1 M.

Figure S.1 DFT calculations (PCM-M06L/def2-TZVPP//PCM-M06L/def2-SVP level) with and without coordination of a THF molecule. Relative free energies (ΔG, at 298 K) and bond distances are given in kcal/mol and angstroms, respectively.

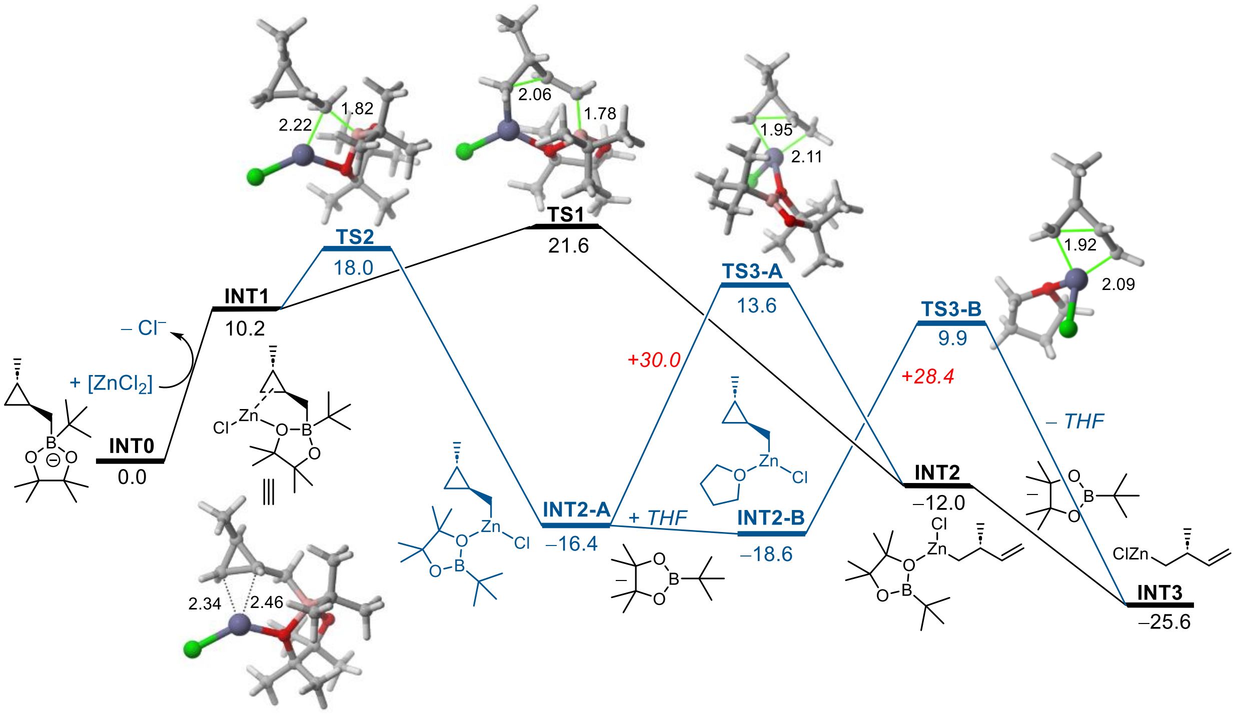

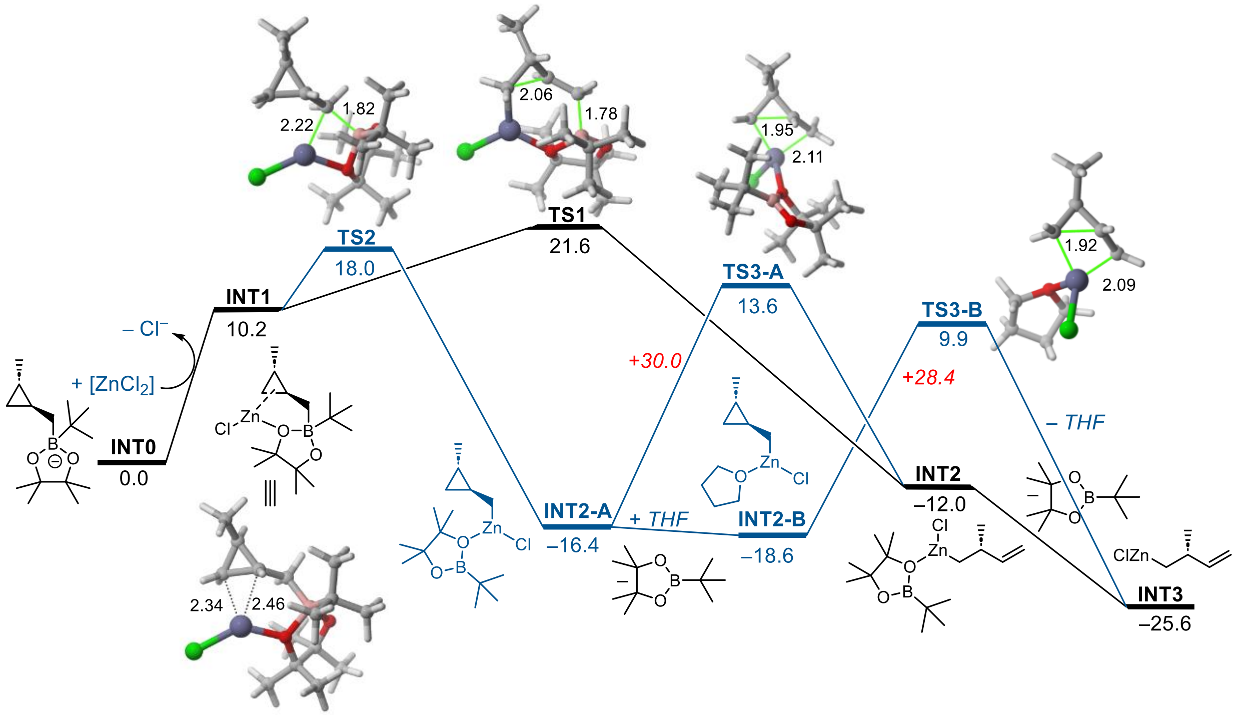

Cartesian coordinates (in Å) and electronic energies (ZPVE included, in a.u.) of all the stationary points discussed in the text. All calculations have been performed at the PCM(tetrahydrofuran)-M06L/def2-SVP level.

INT0, E = -764.051562

C	2.361223000	-0.252051000	0.042183000
C	2.585304000	-1.715171000	0.271138000
C	3.646761000	-0.894220000	-0.406841000
C	1.312270000	0.244720000	-0.923175000
B	-0.158653000	0.633504000	-0.257356000
C	-0.242090000	2.267570000	0.048212000
C	-0.312737000	3.032528000	-1.271446000
C	-1.481368000	2.626731000	0.858384000
C	0.979725000	2.748696000	0.826977000
O	-0.404412000	-0.200730000	0.970717000
C	-1.527888000	-1.026138000	0.811421000
C	-1.804602000	-0.996388000	-0.741002000
O	-1.315948000	0.257517000	-1.137531000
C	-1.212867000	-2.418084000	1.353918000
C	-2.709180000	-0.473590000	1.614586000
C	-3.278416000	-1.094091000	-1.113033000
C	-1.057975000	-2.120226000	-1.468912000
H	2.472268000	0.351358000	0.953393000
H	2.782615000	-2.072258000	1.287542000
H	2.010043000	-2.421078000	-0.336449000
H	3.729285000	-1.044231000	-1.491237000
H	1.729527000	1.121334000	-1.459707000
H	1.194340000	-0.517545000	-1.716249000
H	-0.358538000	4.129806000	-1.121348000
H	0.561610000	2.839133000	-1.915012000
H	-1.204859000	2.744879000	-1.850123000
H	-1.552742000	3.715947000	1.049880000
H	-1.484088000	2.120846000	1.836920000
H	-2.404493000	2.331256000	0.333277000
H	1.917803000	2.587622000	0.270459000
H	0.930252000	3.832040000	1.056469000
H	1.080134000	2.222349000	1.791330000
H	-0.301877000	-2.834739000	0.904136000
H	-1.047432000	-2.367596000	2.441279000
H	-2.039019000	-3.126691000	1.179197000
H	-3.571442000	-1.159817000	1.621495000
H	-2.396386000	-0.320257000	2.659056000
H	-3.046396000	0.494918000	1.225927000
H	-3.391712000	-1.085294000	-2.208015000
H	-3.736912000	-2.024505000	-0.740858000
H	-3.853495000	-0.246646000	-0.717487000
H	-1.502833000	-3.111058000	-1.284162000
H	-1.090998000	-1.932672000	-2.552822000
H	-0.003403000	-2.161752000	-1.170146000
C	4.947675000	-0.611966000	0.286224000
H	4.793586000	-0.461481000	1.365834000
H	5.673286000	-1.434454000	0.176374000
H	5.433010000	0.298856000	-0.099475000

INT1, E = -3003.180982

C	1.835047000	1.095633000	0.545166000
C	3.111423000	1.341040000	1.264927000
C	2.818372000	-0.080753000	0.910967000
C	0.496832000	1.251845000	1.242998000
B	-0.816029000	1.079737000	0.286012000
C	-0.977642000	2.308248000	-0.827891000
C	-0.474131000	1.877765000	-2.207880000
C	-2.437076000	2.750013000	-0.963133000
C	-0.195505000	3.564038000	-0.419980000
O	-2.063599000	0.814965000	0.999639000
C	-2.348278000	-0.561019000	1.114095000
C	-1.791602000	-1.170527000	-0.214172000
O	-0.611059000	-0.350384000	-0.423625000
C	-1.654184000	-1.164692000	2.337838000
C	-3.851034000	-0.732053000	1.259054000
C	-2.711409000	-0.963577000	-1.404053000
C	-1.410212000	-2.634631000	-0.128904000
H	1.875186000	1.398747000	-0.512389000
H	2.966270000	1.570069000	2.326376000
H	2.536256000	-0.756544000	1.726140000
H	0.468504000	0.566914000	2.106835000
H	0.475707000	2.256966000	1.696282000
H	-0.551635000	2.698321000	-2.944259000
H	0.586760000	1.572337000	-2.193316000
H	-1.041033000	1.023643000	-2.606798000
H	-2.541899000	3.559666000	-1.708190000
H	-2.835450000	3.128872000	-0.009938000
H	-3.107672000	1.937022000	-1.275881000
H	0.892496000	3.400511000	-0.361807000
H	-0.349609000	4.381661000	-1.146817000
H	-0.515462000	3.949247000	0.561667000
H	-0.561076000	-1.211021000	2.223372000
H	-1.868441000	-0.539906000	3.215889000
H	-2.009033000	-2.183278000	2.553626000
H	-4.145993000	-1.791306000	1.216256000
H	-4.182564000	-0.333140000	2.227854000
H	-4.391679000	-0.186746000	0.474919000
H	-2.179132000	-1.214717000	-2.332347000
H	-3.598681000	-1.608703000	-1.344467000
H	-3.047425000	0.077220000	-1.474943000
H	-2.302773000	-3.248243000	0.060944000
H	-0.974610000	-2.984202000	-1.076361000
H	-0.694679000	-2.858589000	0.674236000
Zn	1.149630000	-1.061526000	-0.409086000
Cl	2.172728000	-2.922029000	-0.940104000
H	3.469877000	-0.528831000	0.154624000
C	4.256127000	2.051902000	0.606549000
H	5.214735000	1.804674000	1.083617000
H	4.335120000	1.782727000	-0.456102000
H	4.129717000	3.142188000	0.663663000

TS1, E = -3003.162512 (i = -382 cm-1)

C	1.644194000	0.929736000	1.411676000
C	2.871187000	0.437518000	-0.163379000
C	2.932000000	1.461254000	0.969319000
C	0.356071000	1.537959000	1.324883000
B	-0.862363000	1.052019000	0.120352000
O	-0.580586000	-0.374180000	-0.288379000

C	-1.448081000	-1.278537000	0.459025000
C	-2.662474000	-0.349484000	0.817191000
O	-2.141074000	0.968755000	0.771722000
C	-1.800807000	-2.454462000	-0.427965000
C	-0.686268000	-1.776094000	1.679485000
C	-3.224000000	-0.606680000	2.208438000
C	-3.799416000	-0.462331000	-0.194247000
H	1.733330000	-0.012341000	1.975167000
H	3.646567000	-0.331920000	-0.035464000
H	2.912594000	0.916687000	-1.151453000
H	2.828678000	2.489339000	0.590598000
H	-0.216879000	1.254161000	2.216331000
H	0.433307000	2.629971000	1.237642000
H	-2.212574000	-2.131134000	-1.391304000
H	-0.909687000	-3.063688000	-0.628004000
H	-2.545319000	-3.095138000	0.066114000
H	-1.284205000	-2.487092000	2.265287000
H	0.220051000	-2.320052000	1.364055000
H	-0.392275000	-0.960717000	2.354438000
H	-4.079629000	0.059256000	2.385088000
H	-3.580869000	-1.641329000	2.319793000
H	-2.486238000	-0.407426000	2.995836000
H	-4.325724000	-1.424319000	-0.116896000
H	-4.528287000	0.336912000	-0.001151000
H	-3.445359000	-0.350421000	-1.225726000
Zn	1.331275000	-0.863259000	-0.566797000
Cl	1.733223000	-2.966928000	-1.175908000
C	4.108083000	1.332355000	1.906672000
H	4.185476000	0.310493000	2.305441000
H	5.046929000	1.555883000	1.382032000
H	4.024409000	2.021013000	2.758463000
C	-0.828212000	2.116797000	-1.121579000
C	-1.779230000	1.627150000	-2.215257000
H	-2.818856000	1.605535000	-1.858669000
H	-1.753543000	2.292811000	-3.095650000
H	-1.524421000	0.613089000	-2.562444000
C	-1.334309000	3.475056000	-0.633991000
H	-0.663806000	3.937545000	0.107627000
H	-1.417448000	4.192151000	-1.469058000
H	-2.328044000	3.390865000	-0.170306000
C	0.551134000	2.312016000	-1.741930000
H	0.526540000	3.073688000	-2.540799000
H	1.297464000	2.657085000	-1.008080000
H	0.940646000	1.394420000	-2.213086000

INT2, E = -3003.207338

C	1.945313000	1.236764000	1.959416000
C	2.857521000	-0.385239000	0.289491000
C	2.809111000	1.072541000	0.746990000
C	0.943507000	2.108482000	2.103153000
B	-0.911598000	0.926730000	-0.617971000
O	-0.781929000	-0.433799000	-0.359160000
C	-1.737969000	-0.818548000	0.701165000
C	-2.563967000	0.513214000	0.922340000
O	-1.807307000	1.522196000	0.208735000
C	-2.556424000	-1.980704000	0.183363000
C	-0.910408000	-1.248493000	1.894657000
C	-2.674663000	0.931418000	2.373072000
C	-3.938689000	0.486868000	0.275348000
H	2.195963000	0.573189000	2.801850000

H	3.328093000	-0.991433000	1.086789000
H	3.545888000	-0.470817000	-0.571218000
H	2.372867000	1.696574000	-0.056154000
H	0.369916000	2.180518000	3.031475000
H	0.655825000	2.795752000	1.300000000
H	-3.073235000	-1.739634000	-0.752513000
H	-1.900715000	-2.839168000	-0.006730000
H	-3.309497000	-2.275796000	0.927141000
H	-1.544282000	-1.535016000	2.743725000
H	-0.323733000	-2.142052000	1.627003000
H	-0.226868000	-0.454725000	2.224979000
H	-3.216458000	1.883432000	2.444052000
H	-3.232953000	0.184796000	2.954385000
H	-1.691180000	1.067155000	2.837829000
H	-4.596976000	-0.256514000	0.742948000
H	-4.409471000	1.472418000	0.386496000
H	-3.877865000	0.267076000	-0.798697000
Zn	1.214657000	-1.363101000	-0.237825000
Cl	0.591590000	-3.433167000	-0.820780000
C	4.212176000	1.609935000	1.030232000
H	4.687887000	1.051847000	1.852352000
H	4.859601000	1.503400000	0.147332000
H	4.200152000	2.672918000	1.313775000
C	-0.303289000	1.623611000	-1.891976000
C	-1.502014000	1.639696000	-2.862899000
H	-2.339839000	2.233600000	-2.468694000
H	-1.207492000	2.080812000	-3.829078000
H	-1.879209000	0.625624000	-3.070987000
C	0.129581000	3.064075000	-1.624510000
H	1.028358000	3.117335000	-0.991672000
H	0.370953000	3.582313000	-2.566783000
H	-0.661072000	3.641881000	-1.123636000
C	0.831629000	0.831216000	-2.538099000
H	1.127455000	1.283823000	-3.498561000
H	1.738055000	0.804753000	-1.914085000
H	0.536488000	-0.207035000	-2.762525000

INT3, E = -2434.848428

C	-1.644688000	1.153640000	0.416407000
C	-0.975318000	-1.215331000	0.103447000
C	-1.971196000	-0.121131000	-0.303956000
C	-1.159104000	2.266592000	-0.141247000
H	-1.793706000	1.125265000	1.506806000
H	-1.133726000	-2.124640000	-0.500313000
H	-1.165830000	-1.512258000	1.151655000
H	-1.854908000	0.071839000	-1.386750000
H	-0.996163000	2.337311000	-1.222641000
H	-0.905140000	3.149665000	0.450229000
Zn	0.866345000	-0.556262000	-0.000400000
Cl	2.836373000	0.374451000	-0.013873000
C	-3.414101000	-0.542126000	-0.046538000
H	-3.651321000	-1.481495000	-0.566723000
H	-3.586199000	-0.711659000	1.028246000
H	-4.135250000	0.218306000	-0.381341000

TS2, E = -3003.170009 (i = -101 cm-1)

C	-2.438727000	-0.597439000	0.424422000
C	-3.567173000	-0.641166000	1.411223000
C	-3.350003000	0.597190000	0.596825000

C	-0.987754000	-0.552386000	0.882683000
B	0.560814000	-0.925810000	0.000775000
C	0.297943000	-2.117171000	-1.076194000
C	-0.475041000	-1.604325000	-2.294149000
C	1.641001000	-2.642478000	-1.588273000
C	-0.446856000	-3.290381000	-0.436582000
O	1.533538000	-1.210452000	1.016851000
C	2.366896000	-0.097396000	1.302990000
C	2.395230000	0.716302000	-0.041753000
O	1.128796000	0.348832000	-0.656958000
C	1.788934000	0.704936000	2.467533000
C	3.733673000	-0.620423000	1.713705000
C	3.501021000	0.271756000	-0.982991000
C	2.466510000	2.219887000	0.133612000
H	-2.635308000	-1.181895000	-0.480158000
H	-3.254437000	-0.560711000	2.459303000
H	-2.951812000	1.480233000	1.106545000
H	-0.909217000	0.208381000	1.688427000
H	-0.783798000	-1.472706000	1.451767000
H	-0.617807000	-2.395310000	-3.050713000
H	-1.484735000	-1.229881000	-2.058110000
H	0.069005000	-0.786363000	-2.794312000
H	1.502068000	-3.516001000	-2.248684000
H	2.304386000	-2.949898000	-0.765098000
H	2.171337000	-1.882639000	-2.180580000
H	-1.469946000	-3.035528000	-0.120053000
H	-0.536969000	-4.137196000	-1.138970000
H	0.084614000	-3.666262000	0.452570000
H	0.855890000	1.225073000	2.217344000
H	1.573713000	0.021539000	3.300308000
H	2.494962000	1.465739000	2.829795000
H	4.455301000	0.198975000	1.844658000
H	3.655669000	-1.151383000	2.672402000
H	4.137895000	-1.324706000	0.976769000
H	3.330393000	0.699562000	-1.980420000
H	4.482578000	0.618445000	-0.631528000
H	3.540827000	-0.818042000	-1.081494000
H	3.401179000	2.501850000	0.639162000
H	2.455110000	2.723855000	-0.843277000
H	1.638718000	2.625264000	0.730636000
Zn	-0.639123000	1.166891000	-0.470165000
Cl	-1.538028000	3.078362000	-0.997729000
H	-4.060719000	0.835670000	-0.199182000
C	-4.758290000	-1.520351000	1.168405000
H	-5.649767000	-1.162851000	1.704538000
H	-5.012330000	-1.554463000	0.098926000
H	-4.573551000	-2.554963000	1.494002000

INT2-A, E = -3003.214756

C	3.430640000	0.273269000	0.359841000
C	4.551274000	0.518291000	1.335183000
C	4.529934000	-0.735091000	0.506429000
C	2.032437000	-0.045536000	0.814323000
B	-1.574272000	1.103667000	-0.208819000
C	-1.027984000	2.182246000	-1.213376000
C	0.177536000	1.657484000	-1.993000000
C	-2.175237000	2.474287000	-2.193275000
C	-0.657618000	3.469397000	-0.472175000
O	-2.595048000	1.310888000	0.668452000
C	-2.726484000	0.121316000	1.499867000

C	-2.118061000	-0.986891000	0.586871000
O	-1.127592000	-0.209061000	-0.172252000
C	-1.901725000	0.364868000	2.751297000
C	-4.185167000	-0.081883000	1.837236000
C	-3.094358000	-1.538084000	-0.433938000
C	-1.398040000	-2.099759000	1.309934000
H	3.494218000	0.884139000	-0.551134000
H	4.275564000	0.382802000	2.388333000
H	4.284235000	-1.680680000	0.999622000
H	2.073674000	-0.622896000	1.756152000
H	1.484846000	0.884202000	1.062622000
H	0.473642000	2.358769000	-2.790088000
H	1.061750000	1.533380000	-1.346320000
H	-0.033580000	0.689578000	-2.479340000
H	-1.870888000	3.236499000	-2.929055000
H	-3.069175000	2.852180000	-1.675769000
H	-2.472860000	1.577841000	-2.759505000
H	0.156884000	3.308815000	0.251174000
H	-0.317702000	4.244971000	-1.177723000
H	-1.513763000	3.881013000	0.082012000
H	-0.835366000	0.495679000	2.515099000
H	-2.251038000	1.279146000	3.248260000
H	-1.995153000	-0.464273000	3.465605000
H	-4.329060000	-1.023859000	2.384954000
H	-4.542201000	0.734125000	2.478891000
H	-4.814774000	-0.105248000	0.940111000
H	-2.538135000	-2.119633000	-1.181038000
H	-3.837086000	-2.196406000	0.035838000
H	-3.630135000	-0.733055000	-0.956885000
H	-2.092557000	-2.663619000	1.948173000
H	-0.975398000	-2.804160000	0.578199000
H	-0.583769000	-1.726128000	1.946126000
Zn	0.922047000	-1.045001000	-0.477425000
Cl	0.316519000	-2.519706000	-2.015108000
H	5.271684000	-0.832393000	-0.291917000
C	5.585808000	1.575480000	1.084263000
H	6.538006000	1.352431000	1.589974000
H	5.804134000	1.666970000	0.009787000
H	5.256534000	2.565519000	1.435040000

TS3-A, E = -3003.168324 (i = -428 cm-1)

C	-2.398810000	-0.127010000	-1.441022000
C	-2.942864000	0.392732000	0.362085000
C	-3.716792000	0.289303000	-0.949477000
C	-1.889386000	-1.444788000	-1.664608000
B	1.319577000	1.143820000	0.107372000
C	0.656911000	2.387557000	0.805039000
C	-0.351080000	3.035205000	-0.152194000
C	-0.042005000	1.932636000	2.090668000
C	1.739217000	3.413245000	1.150725000
O	2.547139000	1.164047000	-0.475411000
C	2.812328000	-0.153243000	-1.038239000
C	1.918144000	-1.057522000	-0.148133000
O	0.763402000	-0.142533000	0.073082000
C	2.363888000	-0.124264000	-2.488180000
C	4.290638000	-0.442502000	-0.925379000
C	2.513909000	-1.317585000	1.222907000
C	1.451233000	-2.337623000	-0.788649000
H	-1.758929000	0.713906000	-1.746153000
H	-3.341297000	-0.287680000	1.129652000

H	-2.799211000	1.417333000	0.725209000
H	-4.439427000	-0.539846000	-0.944016000
H	-2.620440000	-2.258877000	-1.727580000
H	-1.055747000	-1.508632000	-2.368058000
H	-0.835780000	3.904705000	0.322101000
H	0.133246000	3.393678000	-1.073518000
H	-1.150998000	2.340921000	-0.444729000
H	-0.552902000	2.775715000	2.583784000
H	0.675016000	1.515931000	2.815361000
H	-0.795459000	1.151265000	1.910039000
H	2.269404000	3.767608000	0.255026000
H	1.298158000	4.293323000	1.646085000
H	2.495438000	2.997632000	1.833574000
H	1.280643000	0.044548000	-2.576276000
H	2.876150000	0.691757000	-3.014099000
H	2.607916000	-1.062049000	-3.005046000
H	4.509795000	-1.469265000	-1.250406000
H	4.860525000	0.239877000	-1.569112000
H	4.653011000	-0.319895000	0.101943000
H	1.733763000	-1.717836000	1.883240000
H	3.331262000	-2.048383000	1.165369000
H	2.910053000	-0.396687000	1.675011000
H	2.317523000	-2.984176000	-0.987945000
H	0.782407000	-2.882399000	-0.106707000
H	0.924821000	-2.171700000	-1.734383000
Zn	-1.214431000	-0.960827000	0.273419000
Cl	-0.949713000	-2.142465000	2.156795000
C	-4.309088000	1.561681000	-1.497828000
H	-4.676595000	1.436967000	-2.526399000
H	-5.151601000	1.900799000	-0.879019000
H	-3.562385000	2.370031000	-1.505565000

INT2-B: E = -2666.997180

C	2.327823000	-0.091979000	0.194762000
C	2.752217000	0.499850000	-1.117075000
C	3.789623000	-0.007769000	-0.156272000
C	1.595292000	-1.405618000	0.241967000
C	-0.697831000	1.723133000	-0.617592000
C	-1.838312000	2.718340000	-0.578179000
C	-2.933067000	1.925148000	0.117476000
C	-2.146261000	1.177291000	1.165495000
O	-0.891091000	0.862977000	0.527180000
H	-3.731174000	2.547761000	0.541595000
H	-3.392208000	1.205762000	-0.578545000
H	-2.607192000	0.233454000	1.486418000
H	-1.943354000	1.797964000	2.054955000
H	2.006158000	0.647296000	0.941342000
H	2.552603000	-0.081935000	-2.022641000
H	2.682673000	1.582325000	-1.263038000
H	4.247725000	-0.966048000	-0.430189000
H	1.957925000	-2.061927000	-0.568288000
H	1.830757000	-1.942400000	1.178952000
Zn	-0.366757000	-1.220330000	0.110060000
Cl	-2.442640000	-1.870503000	-0.365986000
H	0.301471000	2.175872000	-0.551320000
H	-1.562108000	3.599565000	0.021316000
H	-2.115362000	3.075728000	-1.578282000
H	-0.731389000	1.103598000	-1.531818000
C	4.703357000	0.951792000	0.546941000
H	5.097919000	0.532173000	1.485051000

H	5.569607000	1.232057000	-0.072402000
H	4.175225000	1.882259000	0.804292000

TS3-B: E = -2666.952986 (i = -452 cm-1)

C	2.399780000	0.163999000	0.684610000
C	1.853021000	-0.333597000	-1.091416000
C	2.844373000	-0.974541000	-0.124526000
C	1.471967000	0.200983000	1.781668000
C	-1.984701000	-0.990297000	1.218453000
C	-3.339280000	-0.659875000	0.644699000
C	-3.287452000	-1.384307000	-0.691595000
C	-1.860025000	-1.141951000	-1.137857000
O	-1.091411000	-0.978158000	0.082362000
H	-3.474933000	-2.460377000	-0.552964000
H	-4.018027000	-1.015621000	-1.422813000
H	-1.763169000	-0.210745000	-1.720295000
H	-1.424417000	-1.969589000	-1.715333000
H	2.940457000	1.093727000	0.457790000
H	1.095540000	-1.048397000	-1.444357000
H	2.315034000	0.251569000	-1.894707000
H	2.458834000	-1.926906000	0.269611000
H	1.245228000	-0.754513000	2.270089000
H	1.571500000	1.059977000	2.450284000
Zn	0.295993000	0.639796000	0.115697000
Cl	-1.075899000	2.283764000	-0.517750000
H	-1.959429000	-1.993571000	1.676822000
H	-4.167125000	-0.979173000	1.290458000
H	-3.421900000	0.426962000	0.490719000
H	-1.606413000	-0.266019000	1.953581000
C	4.290780000	-1.068272000	-0.533490000
H	4.936219000	-1.364336000	0.306027000
H	4.426712000	-1.806733000	-1.336182000
H	4.656877000	-0.101720000	-0.910072000

10. References

(1) Gupta, P.; Kumar, P. *Tetrahedron Asymmetry* **2007**, *18*, 1688–1692.

(2) Yao, Y. X.; Zhang, H. W.; Lu, C. B.; Shang, H. Y.; Tian, Y. Y. *European J. Org. Chem.* **2023**, *26*, e202300111.

(3) Kon, Y.; Nakashima, T.; Makino, Y.; Nagashima, H.; Onozawa, S. ya; Kobayashi, S.; Sato, K. *Adv. Synth. Catal.* **2023**, 365, 3227–3233.

(4) Donohoe, T. J.; Ironmonger, A.; Kershaw, N. M. *Angew. Chem. Int. Ed.* **2008**, *47*, 7314–7316.

(5) Jain, P.; Antilla, J.C. *J. Am. Chem. Soc.* **2010**, *132*, 11884–11886.

(6) Alam, M.; Wise, C.; Baxter, C. A.; Cleator, E.; Walkinshaw, A. *Org. Process Res. Dev*. **2012**, *16*, 435–441.

(7) Triandafillidi, I.; Kokotou, M. G.; Kokotos, C. G. *Org. Lett.* **2018**, *20*, 36–39.

(8) Liang, X.; Wei, K.; Yang, Y. R. *Chem. Comm.* **2015**, *51*, 17471–17474.

(9) Parsutkar, M. M.; Rajanbabu, T. V. *J. Am. Chem. Soc.* **2021**, *143*, 12825–12835.

(10) Bing Li; Changkun Li. *Synlett* **2022**, *33*, 1867.

(11) Chen, T.; Yang, H.; Yang, Y.; Dong, G.; Xing, D. *ACS Catal.* **2020**, *10*, 4238–4243.

(12) Suzuki, T.; Sato, O.; Hirama, M.; Yamamoto, Y.; Murata, M.; Yasumoto, T.; Harada, N. *Tetrahedron Lett.* **1991**, *32*, 4505–4508.

(13) Koseki, Y.; Kusano, S.; Ichi, D.; Yoshida, K.; Nagasaka, T. *Tetrahedron* **2000**, *56*, 8855-8865.

(14) Itoh, T.; Shimizu, Y.; Kanai, M. *J. Am. Chem. Soc.* **2016**, *138*, 7528–7531.

(15) Kubota, K.; Yamamoto, E.; Ito, H. *J. Am. Chem. Soc.* **2013**, *135*, 2635–2640.

(16) Gaussian 16, Revision B.01, Frisch, M. J.; Trucks, G. W.; Schlegel, H. B.; Scuseria, G. E.; Robb, M. A.; Cheeseman, J. R.; Scalmani, G.; Barone, V.; Petersson, G. A.; Nakatsuji, H.; Li, X.; Caricato, M.; Marenich, A. V.; Bloino, J.; Janesko, B. G.; Gomperts, R.; Mennucci, B.; Hratchian, H. P.; Ortiz, J. V.; Izmaylov, A. F.; Sonnenberg, J. L.; Williams-Young, D.; Ding, F.; Lipparini, F.; Egidi, F.; Goings, J.; Peng, B.; Petrone, A.; Henderson, T.; Ranasinghe, D.; Zakrzewski, V. G.; Gao, J.; Rega, N.; Zheng, G.; Liang, W.; Hada, M.; Ehara, M.; Toyota, K.; Fukuda, R.; Hasegawa, J.; Ishida, M.; Nakajima, T.; Honda, Y.; Kitao, O.; Nakai, H.; Vreven, T.; Throssell, K.; Montgomery, J. A., Jr.; Peralta, J. E.; Ogliaro, F.; Bearpark, M. J.; Heyd, J. J.; Brothers, E. N.; Kudin, K. N.; Staroverov, V. N.; Keith, T. A.; Kobayashi, R.; Normand, J.; Raghavachari, K.; Rendell, A. P.; Burant, J. C.; Iyengar, S. S.; Tomasi, J.; Cossi, M.; Millam, J. M.; Klene,

M.; Adamo, C.; Cammi, R.; Ochterski, J. W.; Martin, R. L.; Morokuma, K.; Farkas, O.; Foresman, J. B.; Fox, D. J. Gaussian, Inc., Wallingford CT, **2016**.

(17) Zhao, Y.; Truhlar, D. G. *Theor. Chem. Acc.* **2008**, *120*, 215.

(18) Weigend, F.; Ahlrichs, R. *Phys. Chem. Chem. Phys*. **2005**, *7*, 3297.

(19) (a) Miertuš, S.; Scrocco, E.; Tomasi, J. *Chem. Phys*. **1981**, *55*, 117; (b) Pascual-Ahuir, J. L.; Silla, E.; Tuñón, I. *J. Comp. Chem*. **1994**, *15*, 1127; (c) Barone, V.; Cossi, M. *J. Phys. Chem. A*, **1998**, *102*, 1995.

(20) McIver, J. W.; Komornicki, A. K. *J. Am. Chem. Soc.* **1972**, *94*, 2625.

(21) González, C.; Schlegel, H. B. *J. Phys. Chem*. **1990**, *94*, 5523.

(22) Grimme, S. *Chem. Eur. J.* **2012**, *18*, 9955.

(23) Luchini, G.; Alegre-Requena, J. V.; Funes-Ardoiz, I.; Paton, R. S. *F1000 Research* **2020**, *9*, 291.

11. Characterization data

11.1. NMR Data: Starting Materials

^{1}H NMR (300 MHz, $CDCl_3$) of compound **S1e** (* denotes pentane due to the high volatility of the compound)

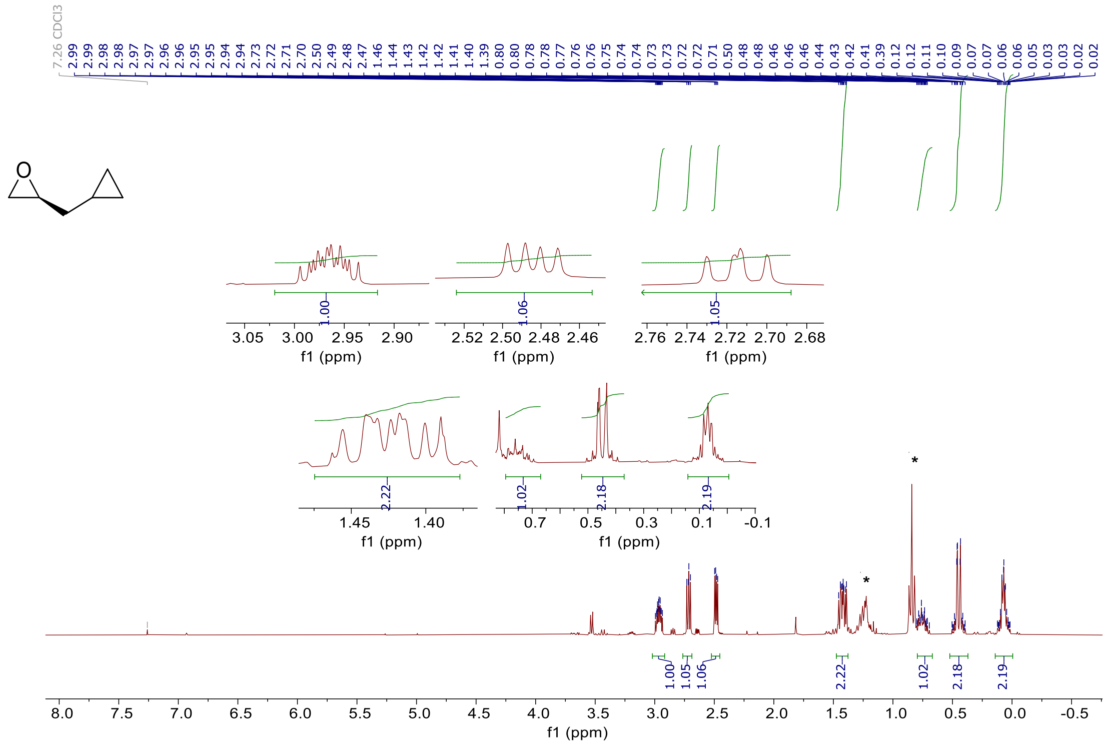

^{13}C NMR (75 MHz, $CDCl_3$) of compound **S1e** (* denotes pentane due to the high volatility of the compound)

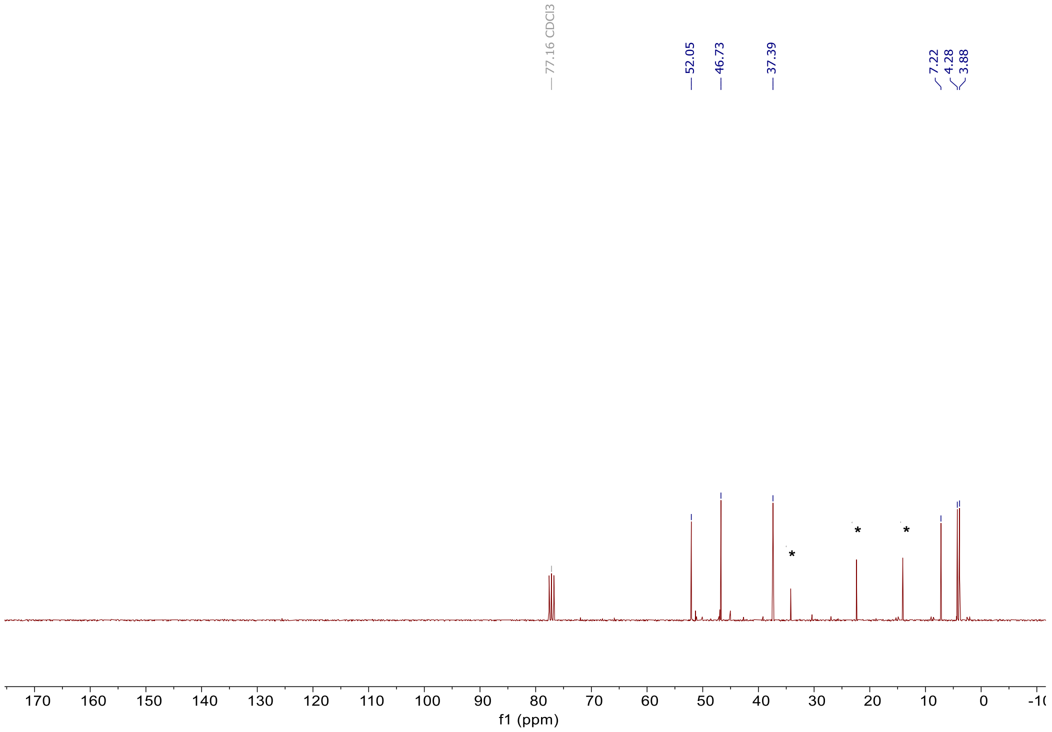

^{1}H NMR (500 MHz, $CDCl_3$) of compound **1a**

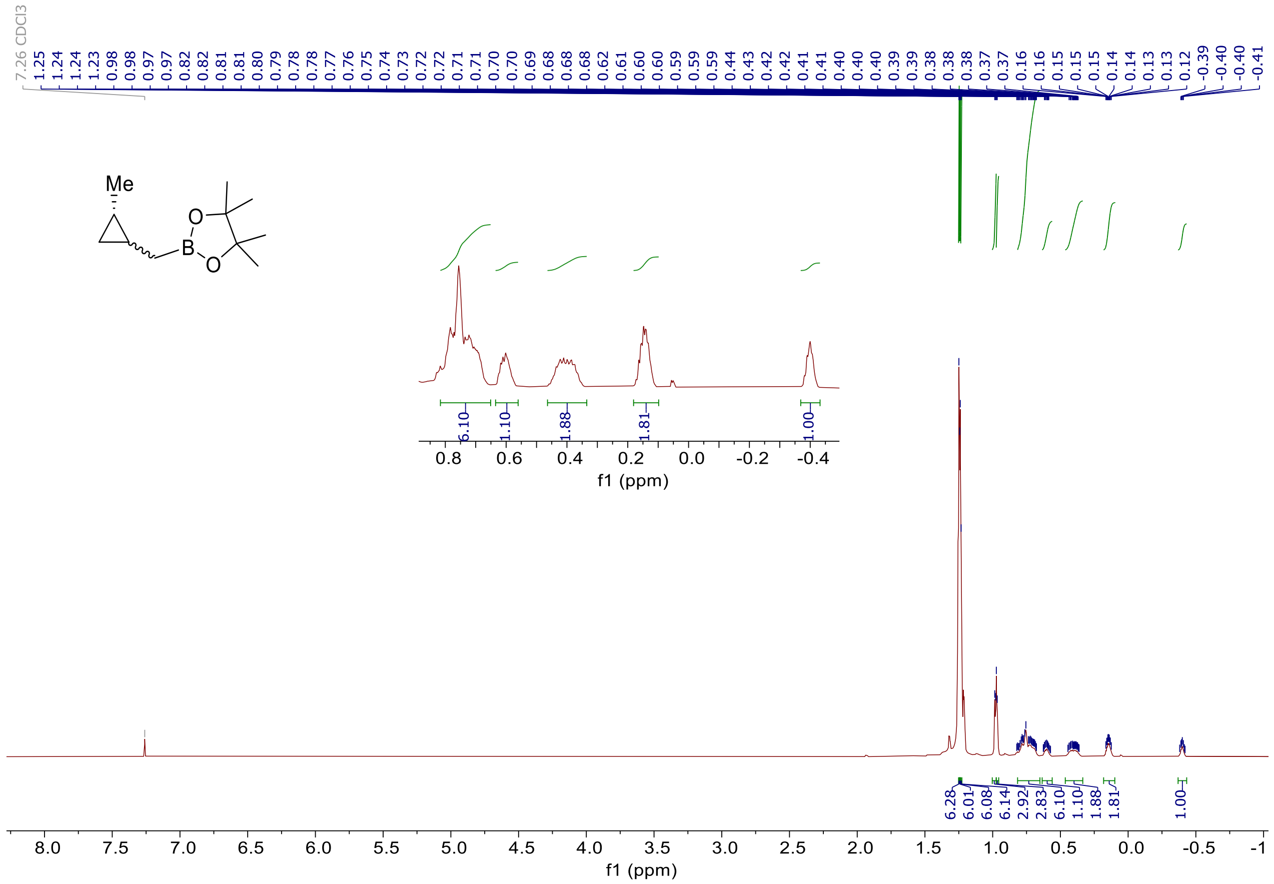

^{13}C NMR (126 MHz, $CDCl_3$) of compound **1a**

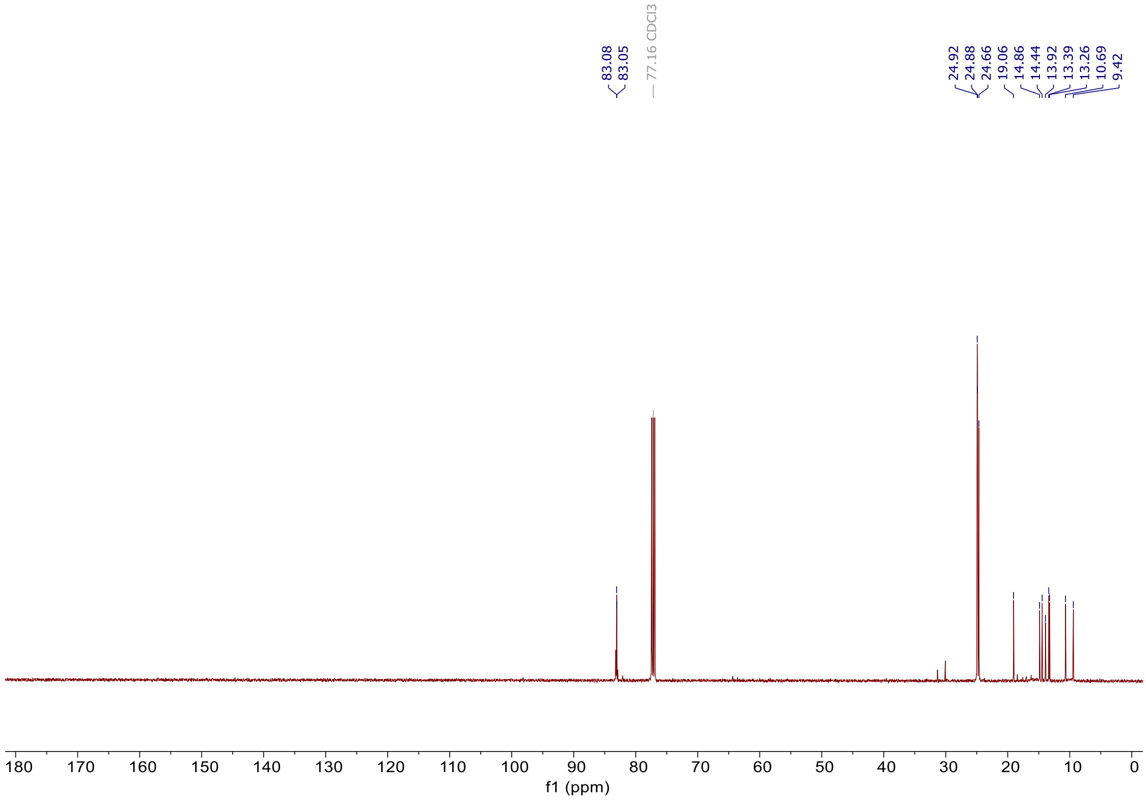

^{11}**B NMR** (160 MHz, $CDCl_3$) of compound **1a**

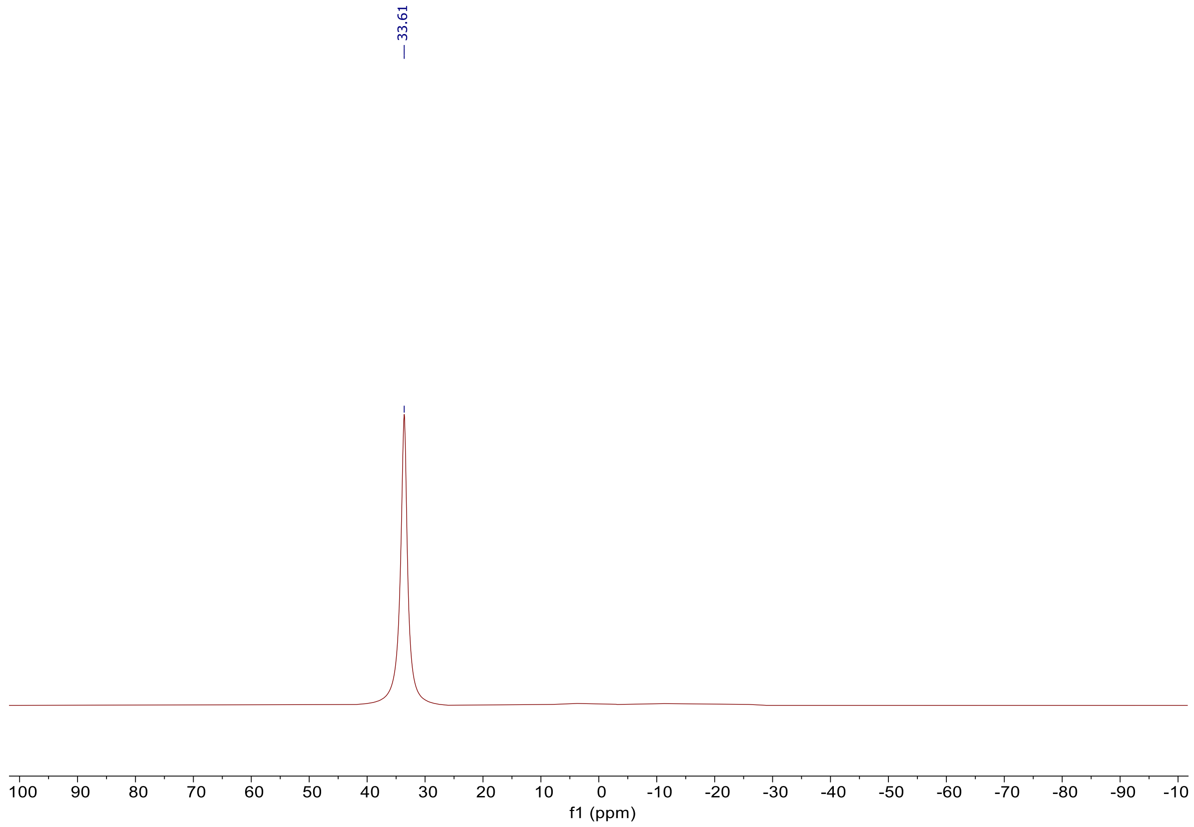

^{1}**H NMR** (500 MHz, $CDCl_3$) of compound **1b**

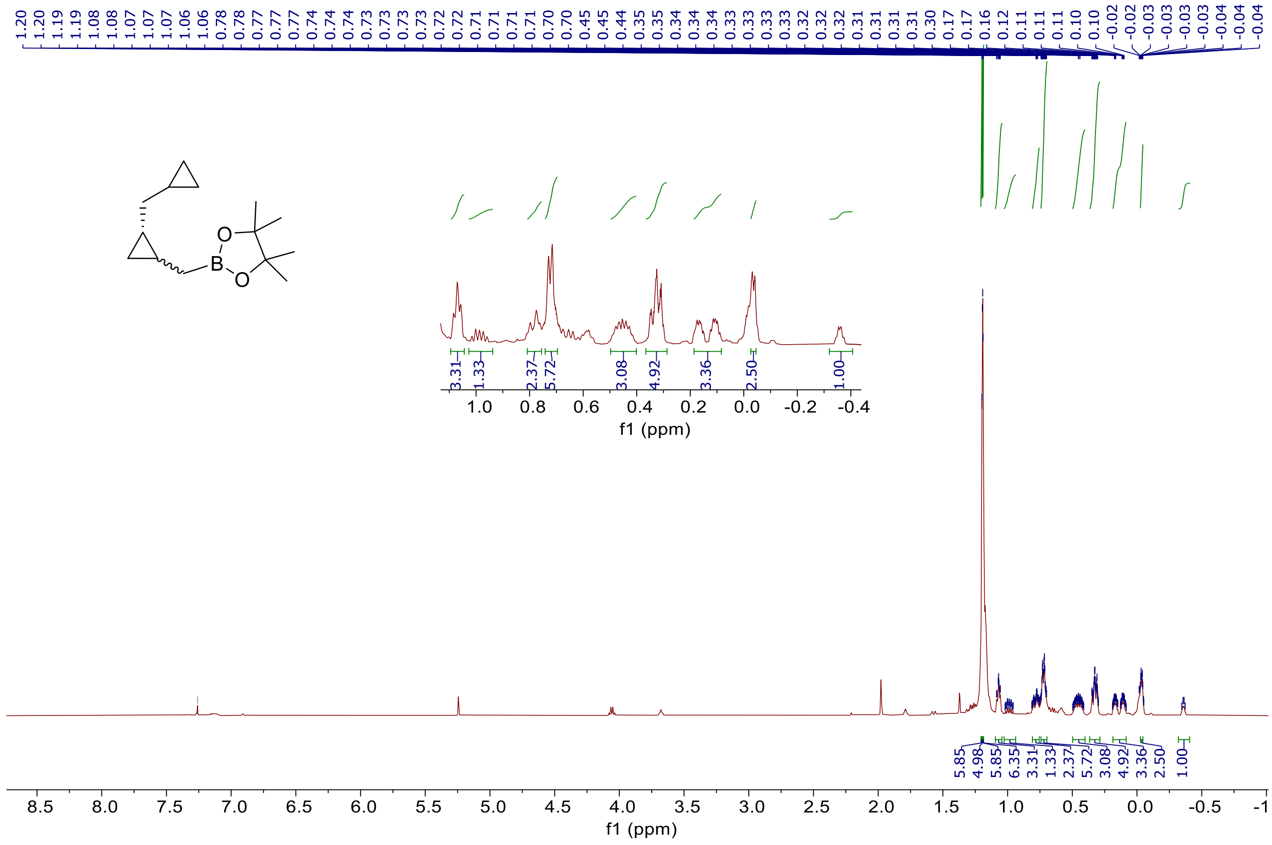

^{13}**C NMR** (126 MHz, $CDCl_3$) of compound **1b**

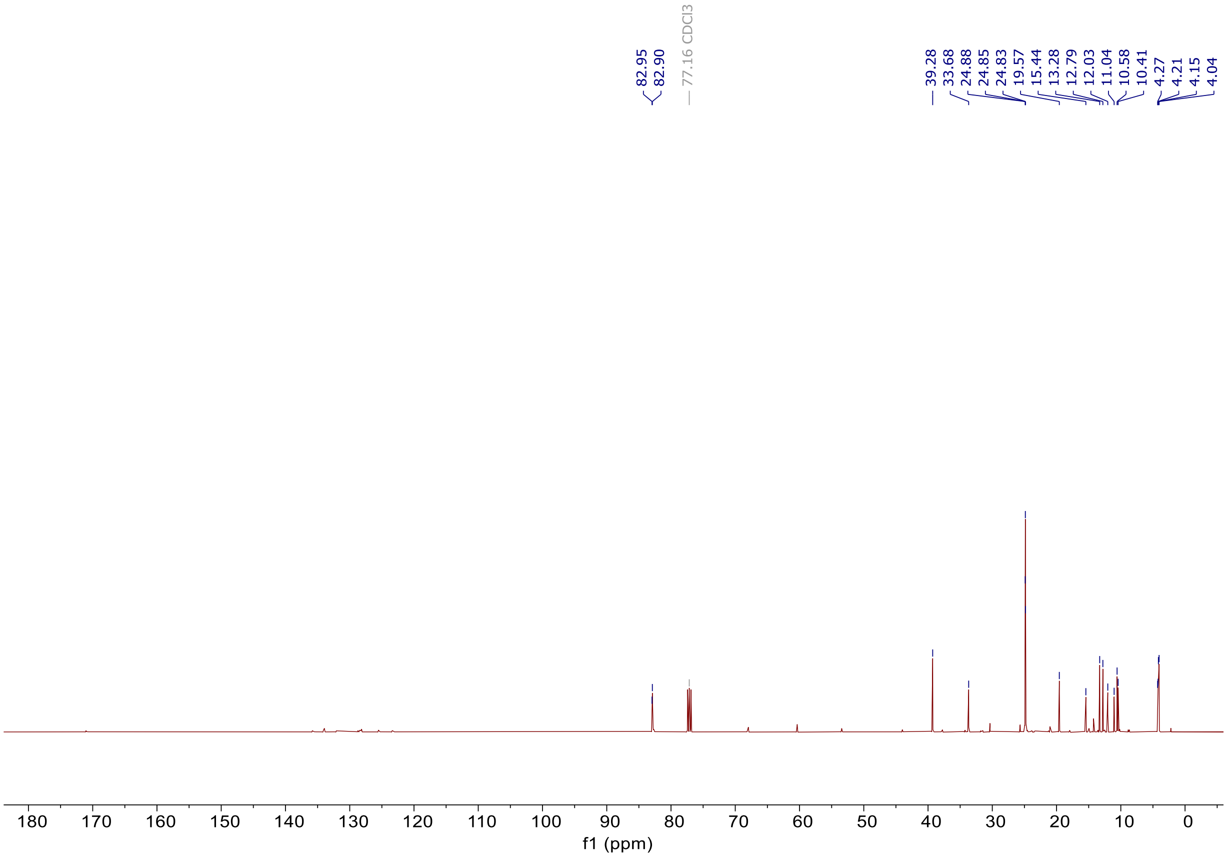

^{11}**B NMR** (160 MHz, $CDCl_3$) of compound **1b**

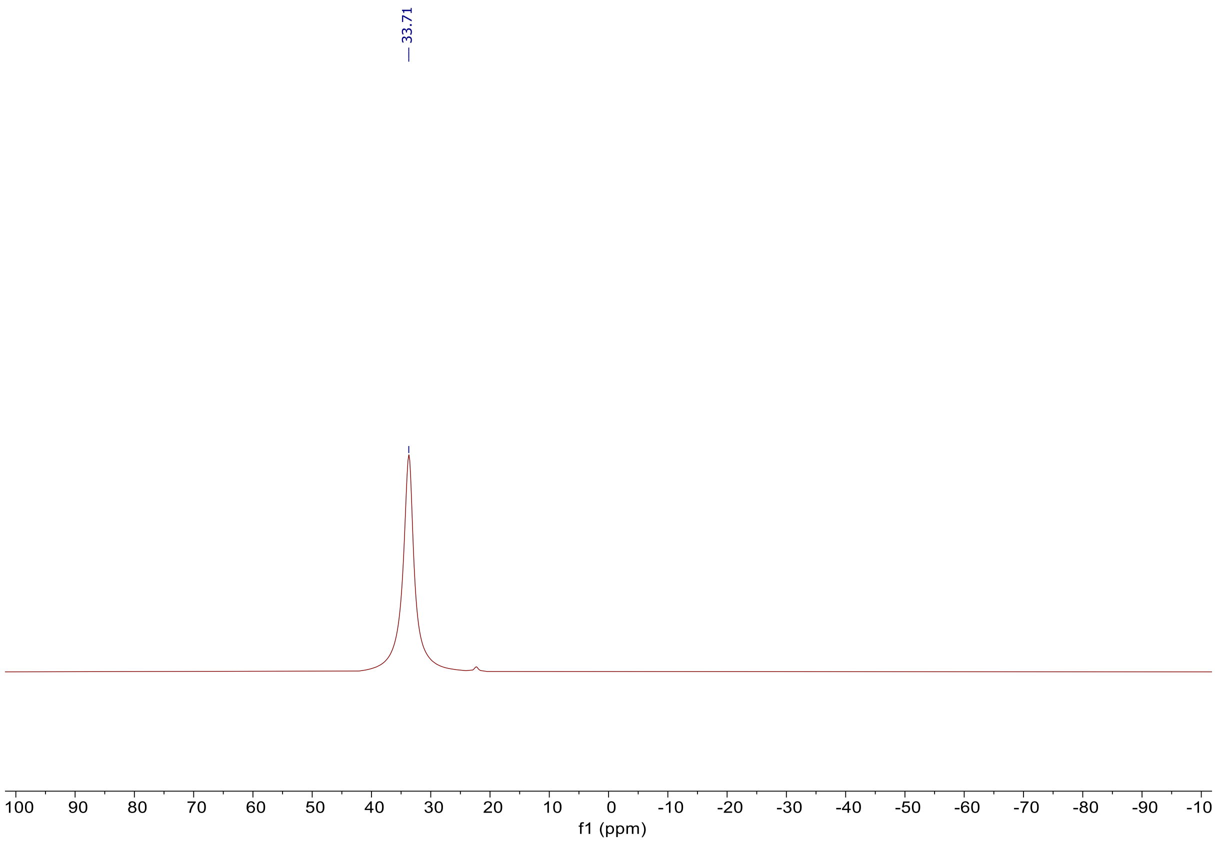

^{1}H NMR (500 MHz, $CDCl_3$) of compound **1c**

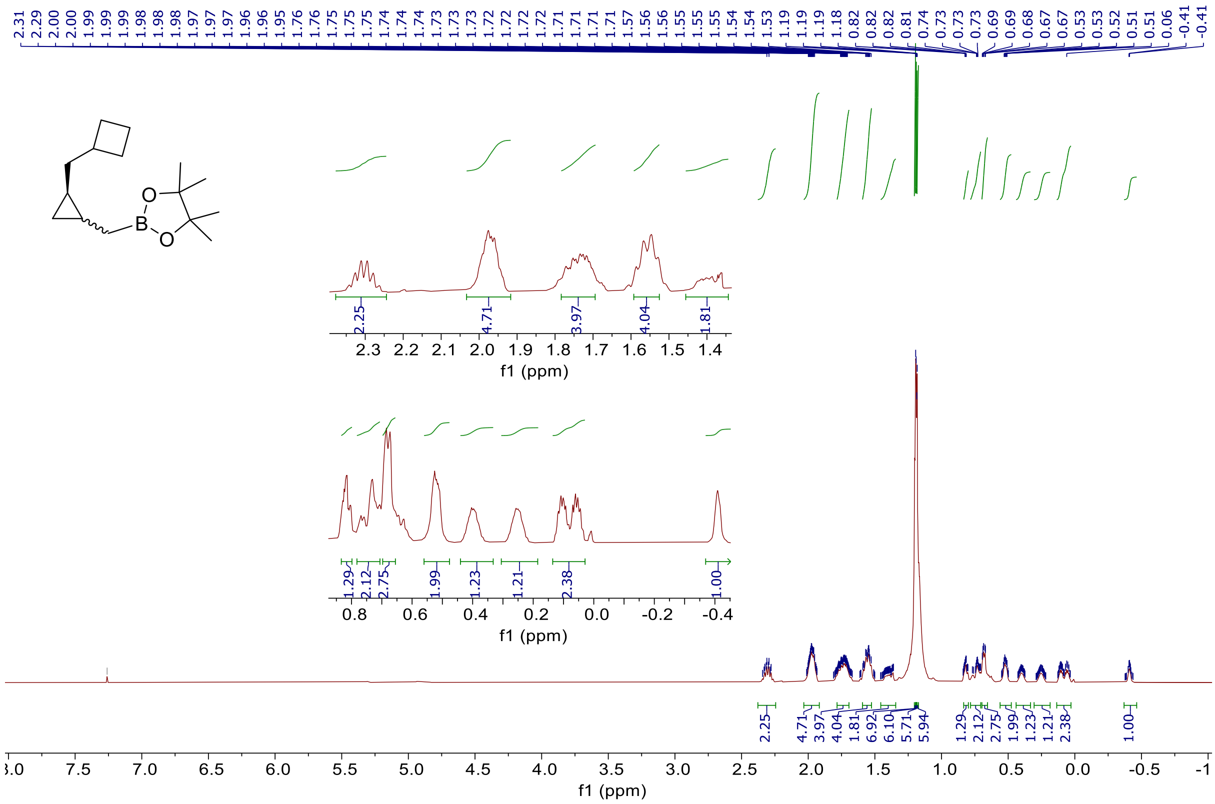

^{13}C NMR (126 MHz, $CDCl_3$) of compound **1c**

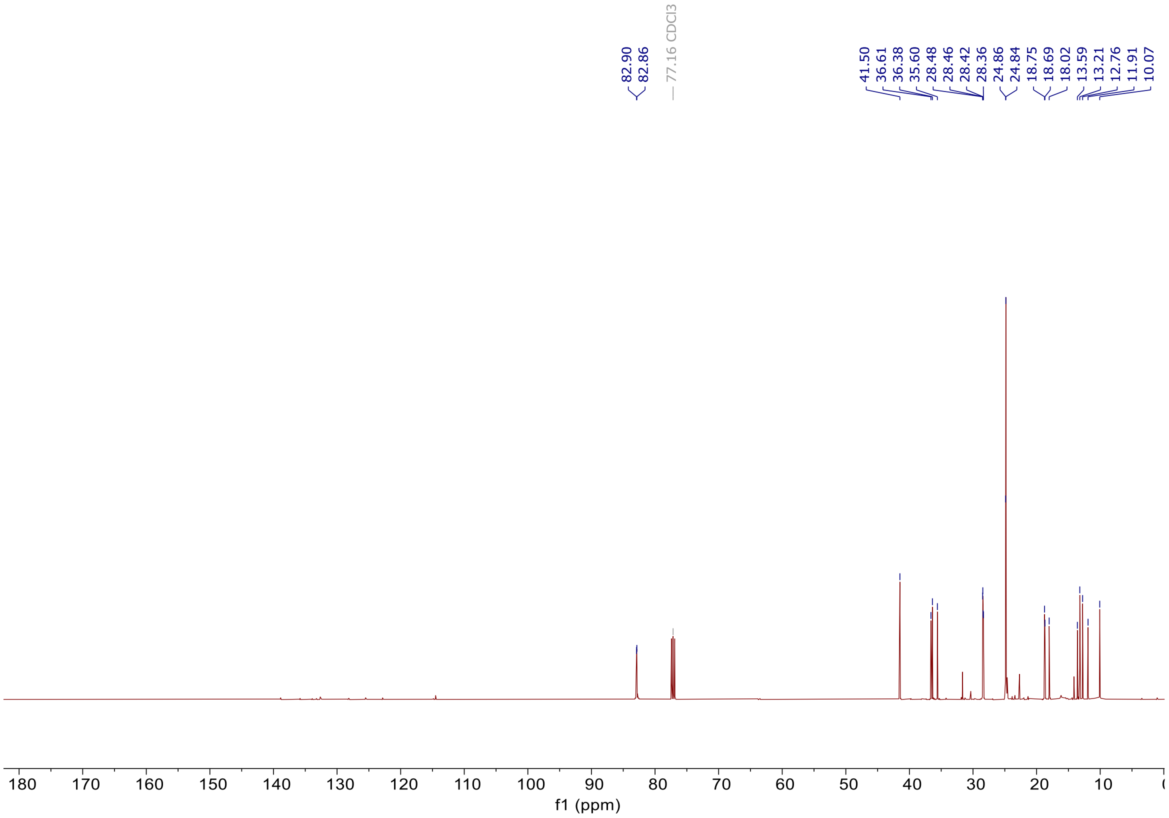

^{11}B NMR (160 MHz, $CDCl_3$) of compound **1c**

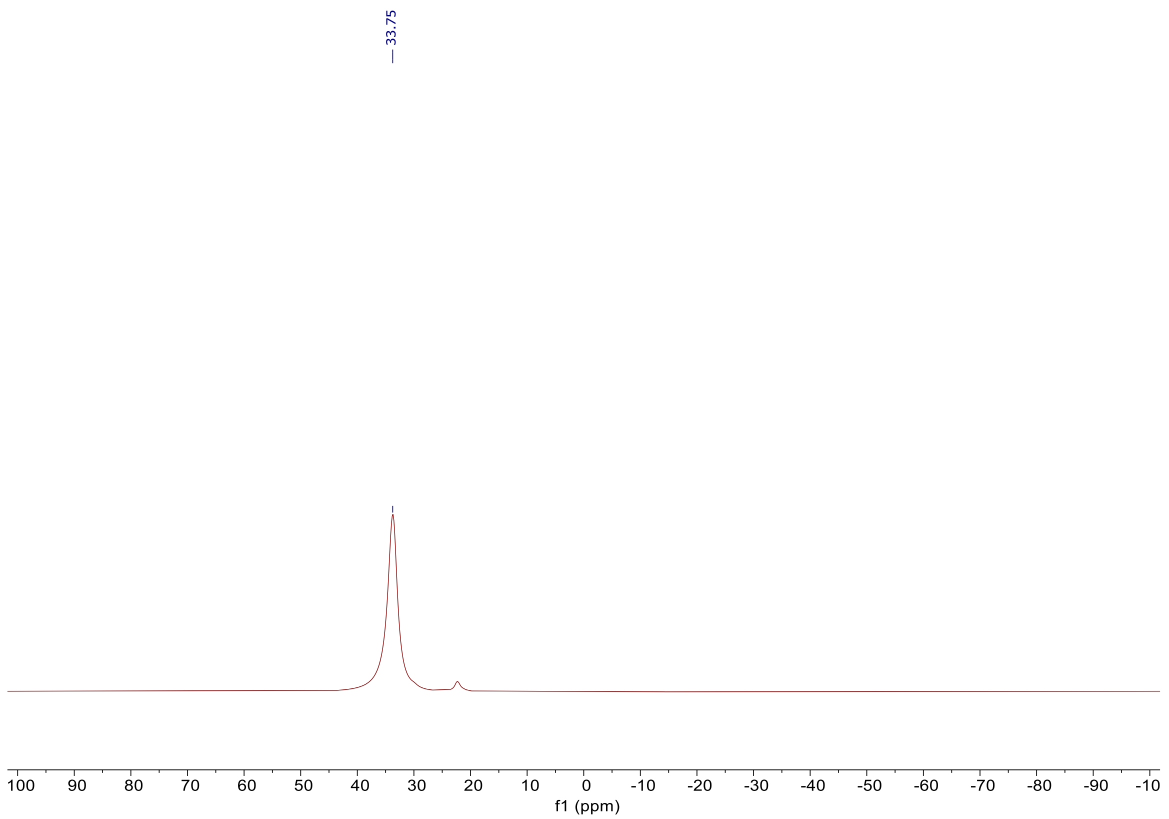

^{1}H NMR (500 MHz, $CDCl_3$) of compound **1d**

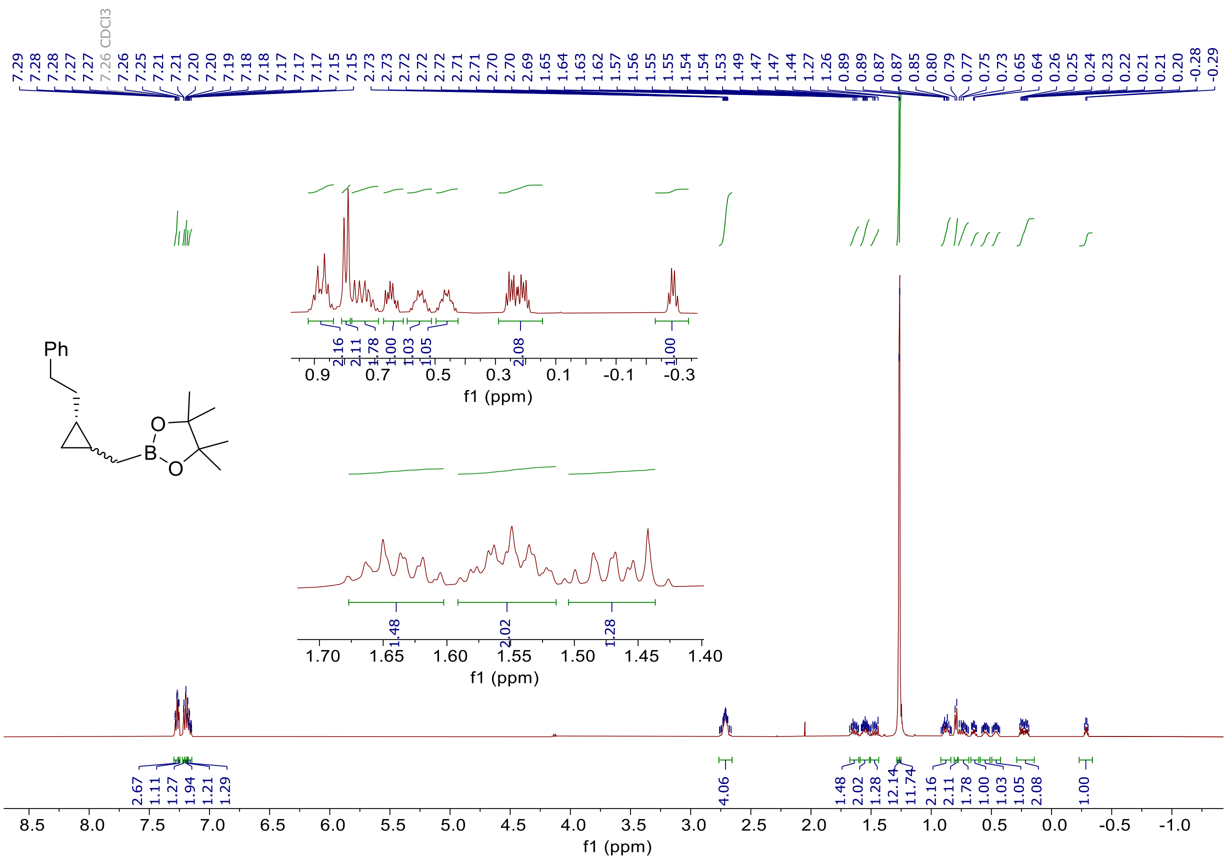

^{13}C NMR (126 MHz, $CDCl_3$) of compound **1d**

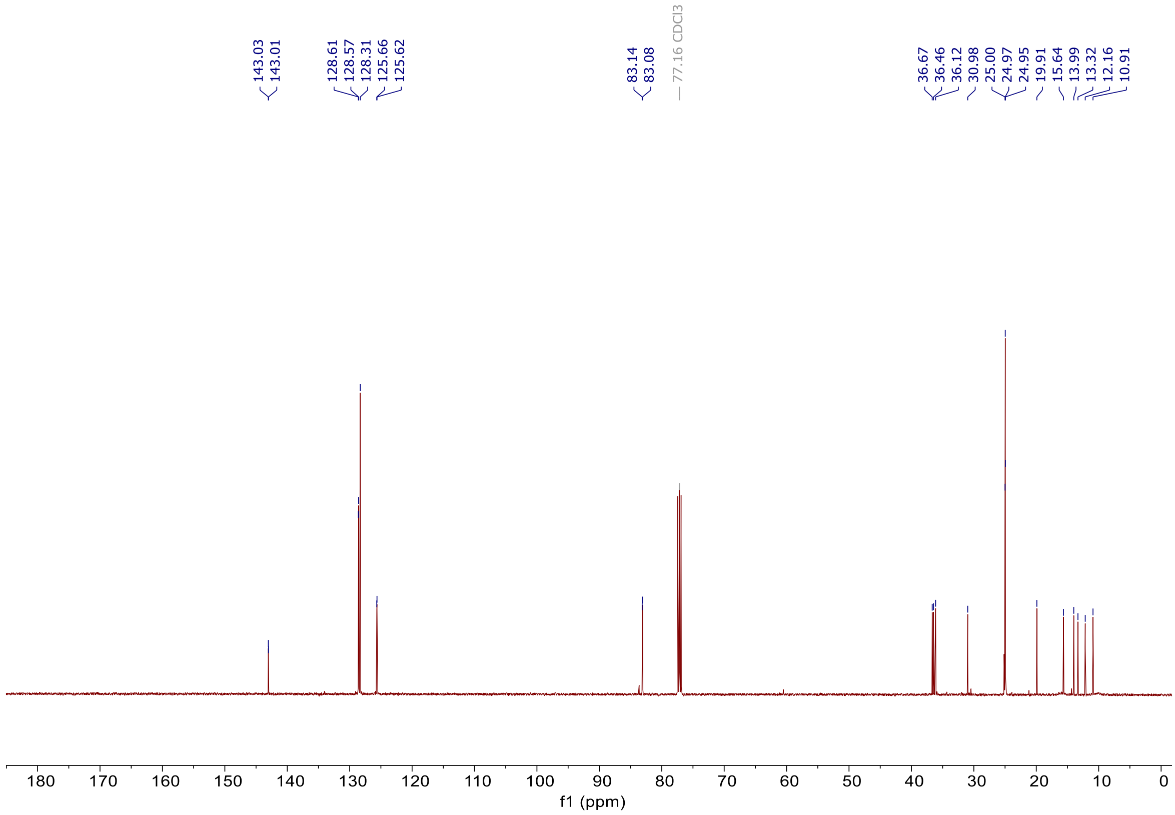

^{11}B NMR (160 MHz, $CDCl_3$) of compound **1d**

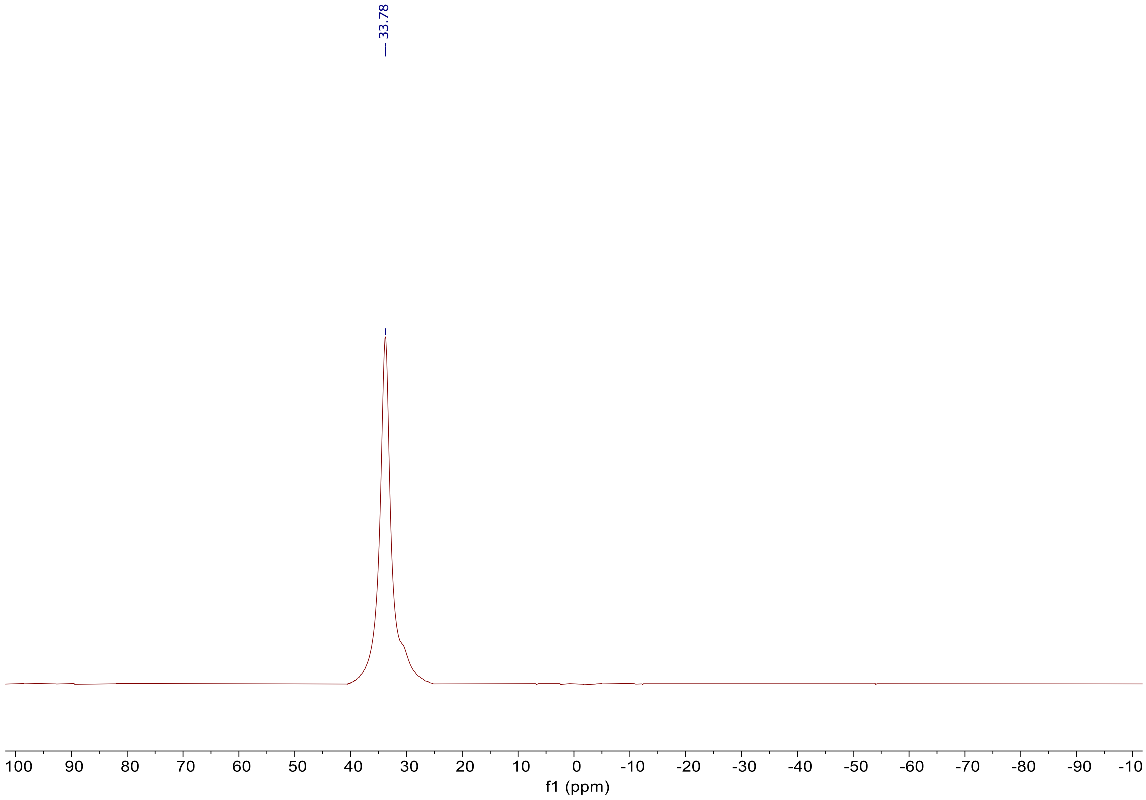

^{1}H NMR (500 MHz, $CDCl_3$) of compound **1e**

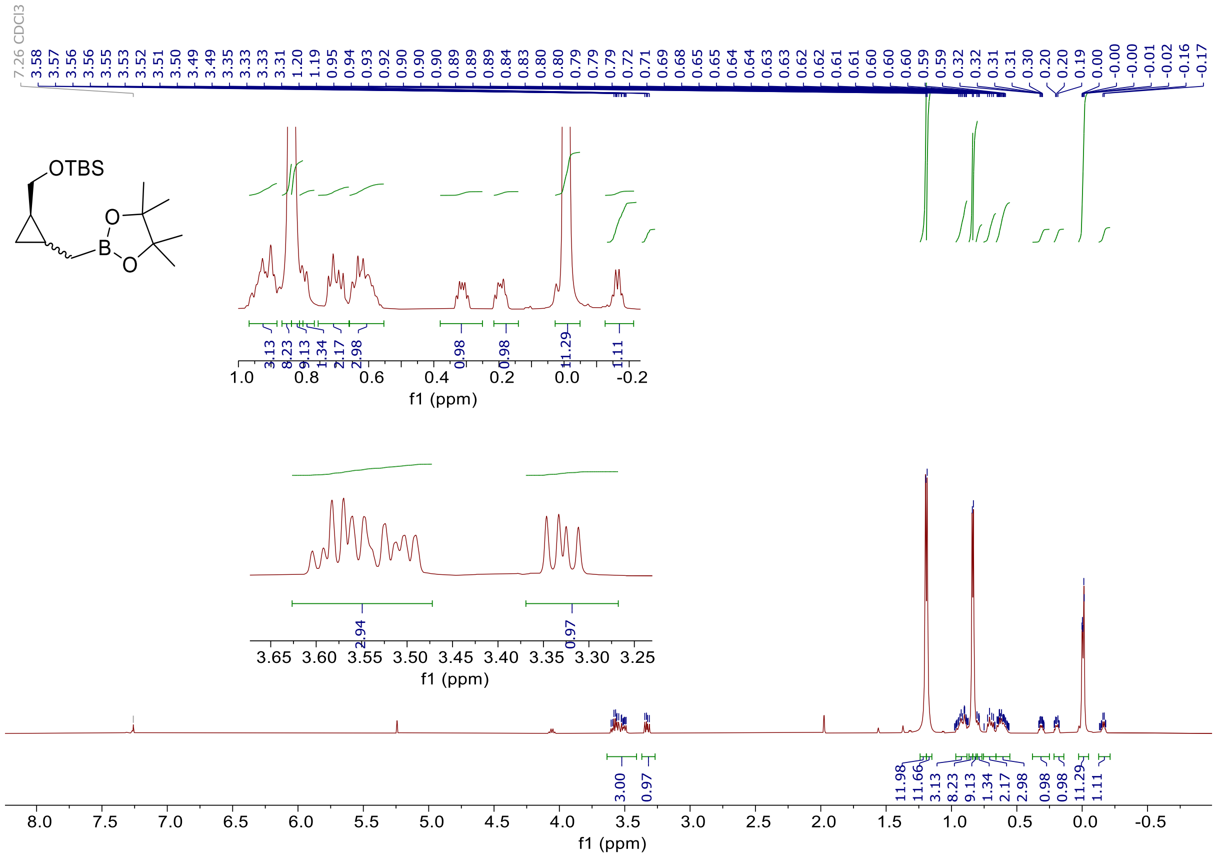

^{13}C NMR (126 MHz, $CDCl_3$) of compound **1e**

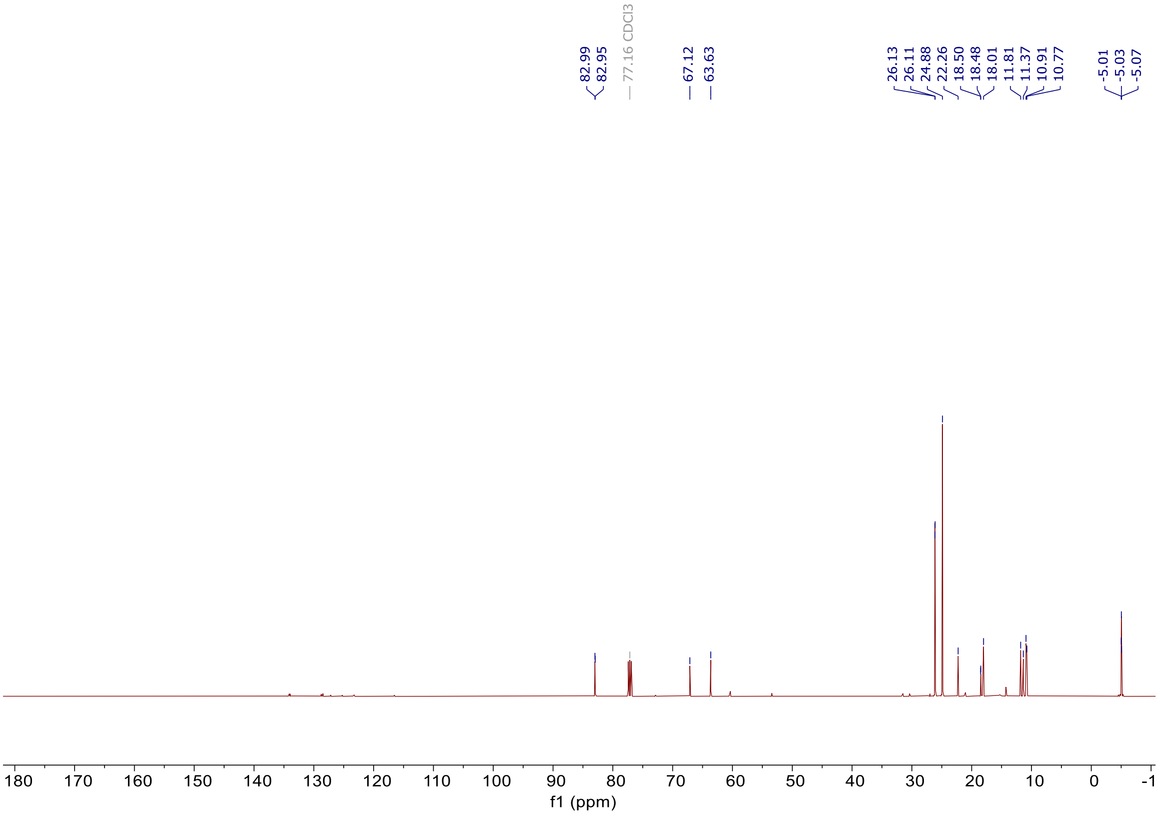

^{11}B NMR (160 MHz, $CDCl_3$) of compound **1e**

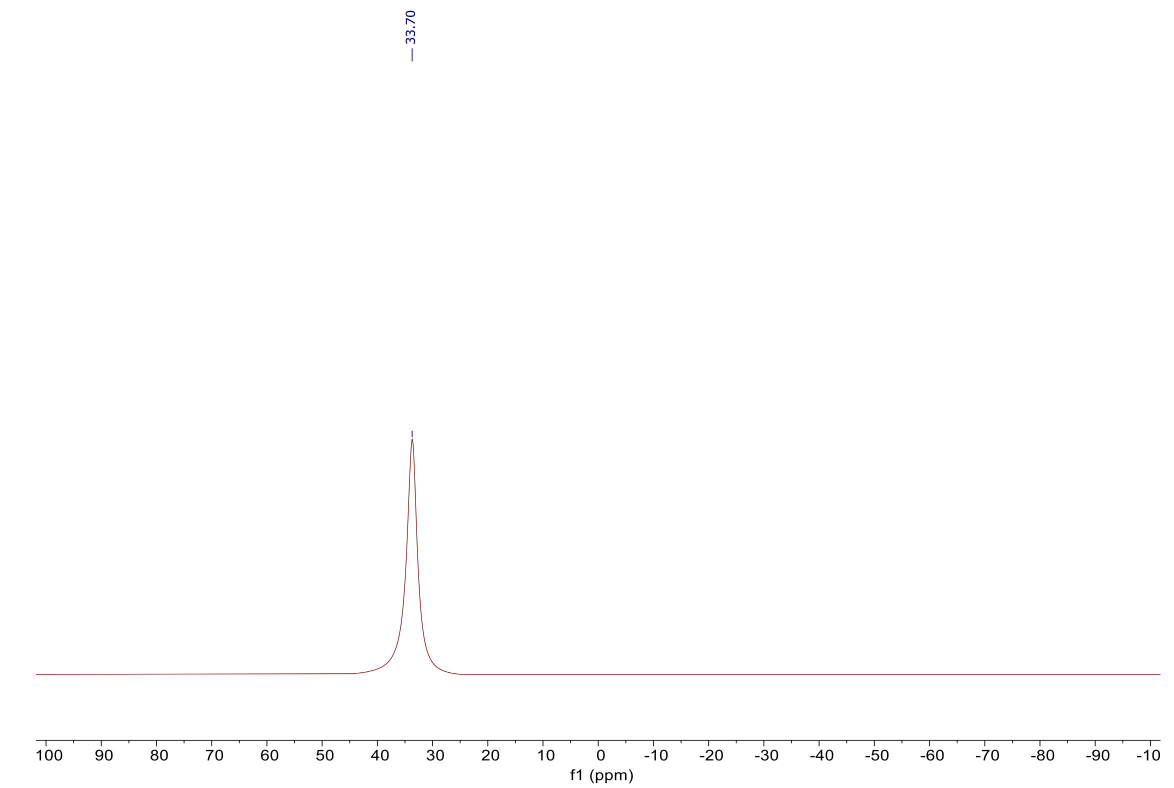

^{1}H NMR (500 MHz, $CDCl_3$) of compound **1f**

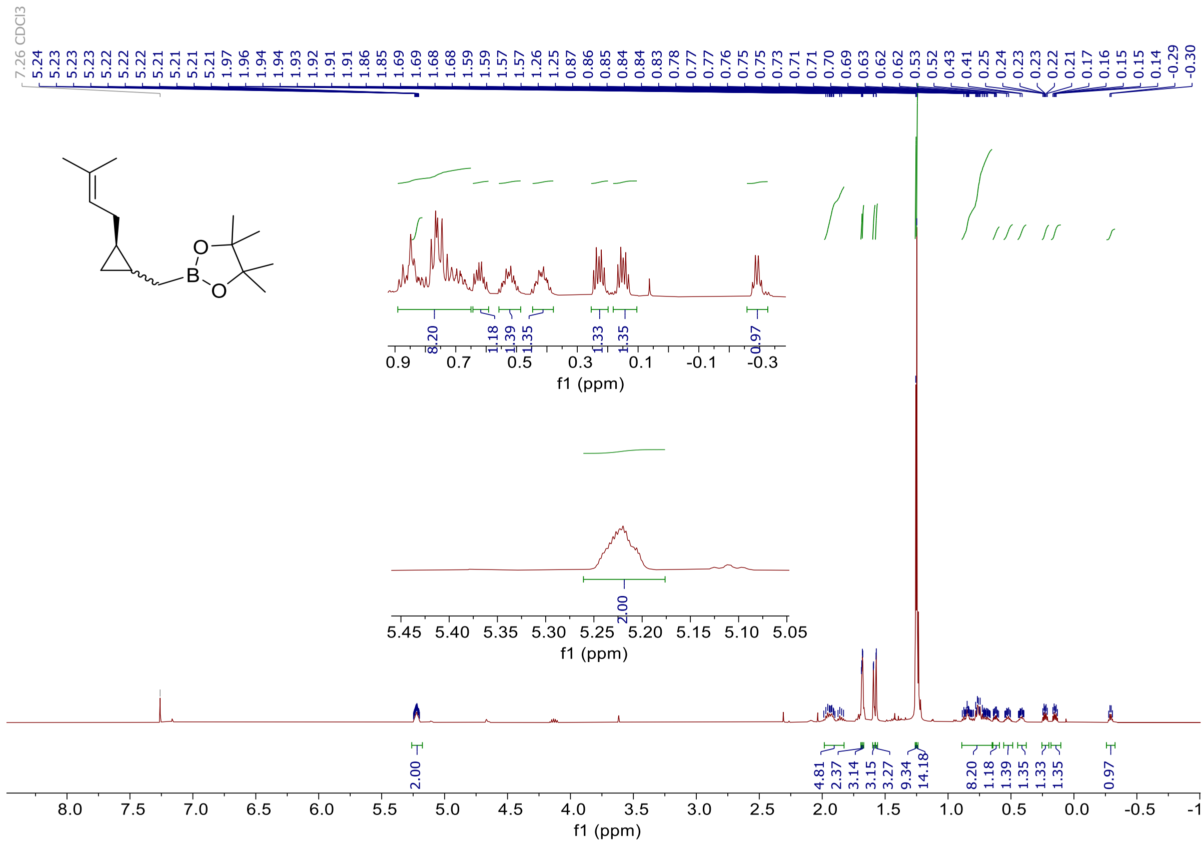

^{13}C NMR (126 MHz, $CDCl_3$) of compound **1f**

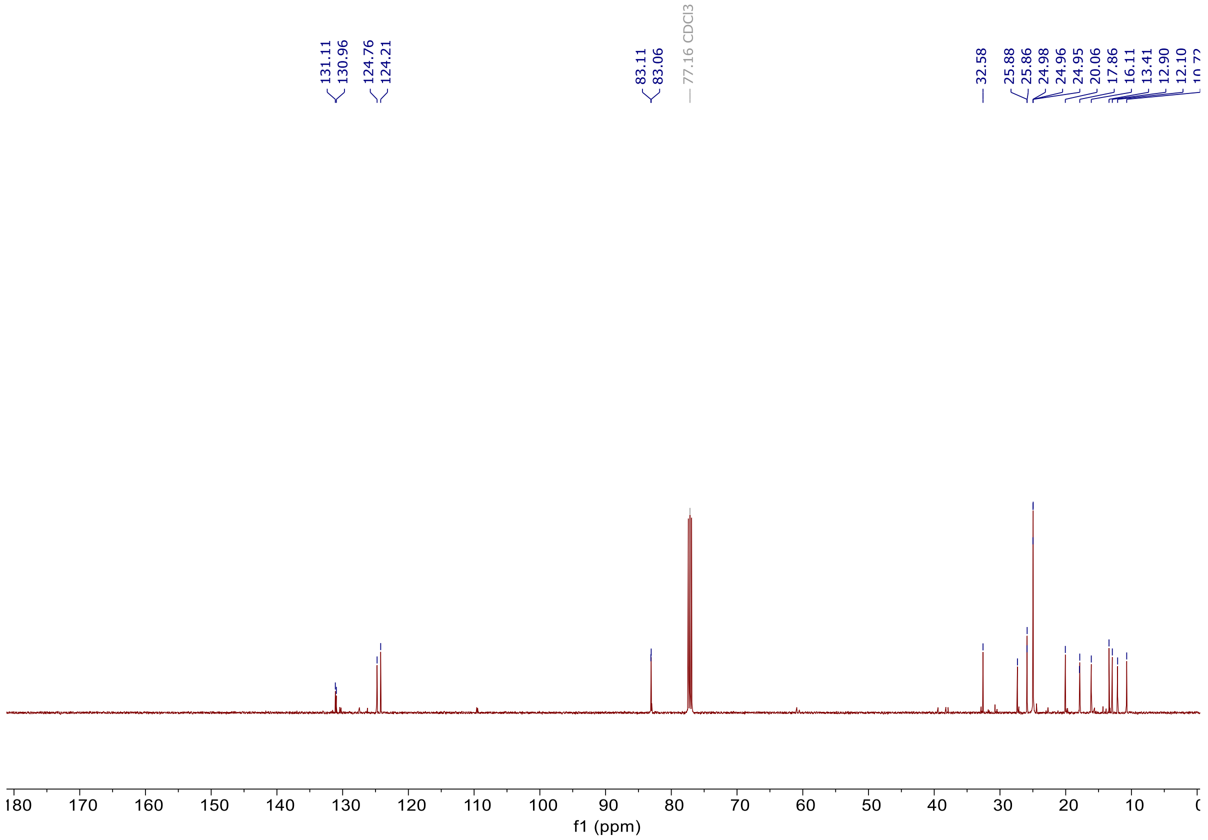

^{11}B NMR (160 MHz, $CDCl_3$) of compound **1f**

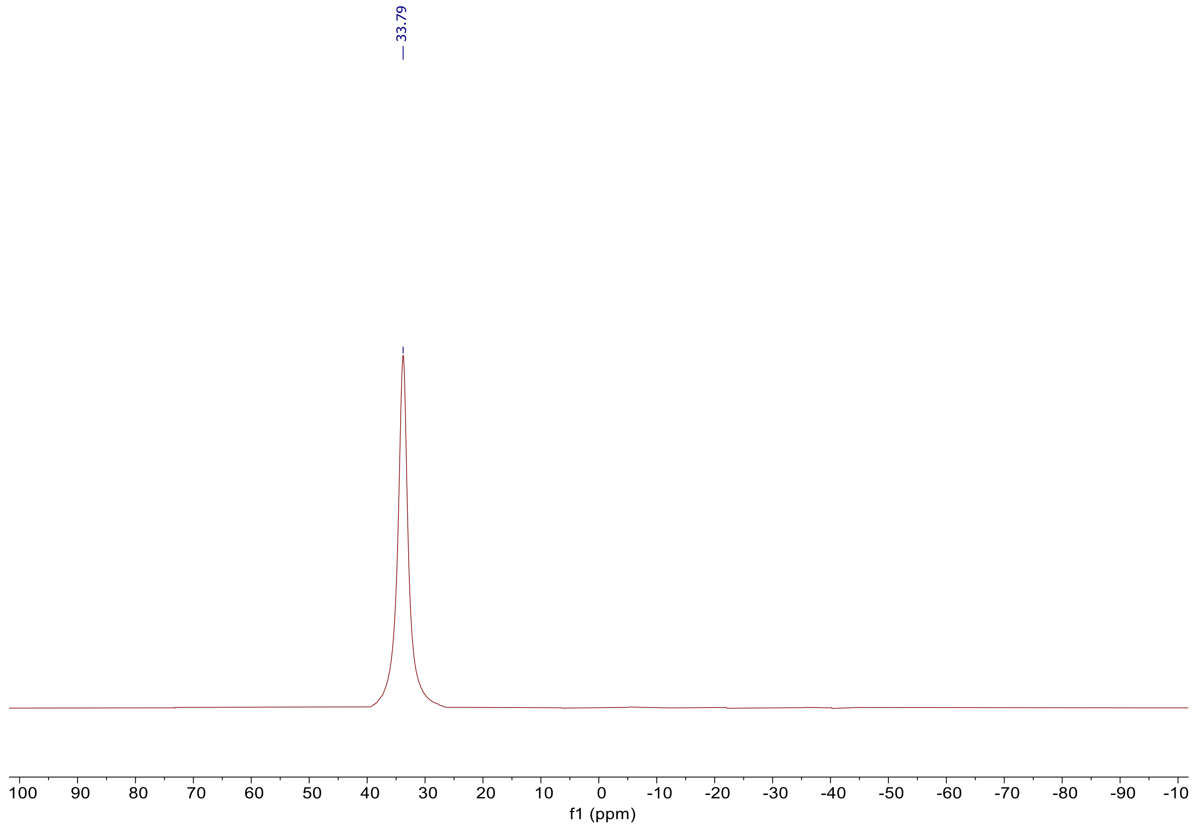

^{1}H NMR (500 MHz, $CDCl_3$) of compound **1g**

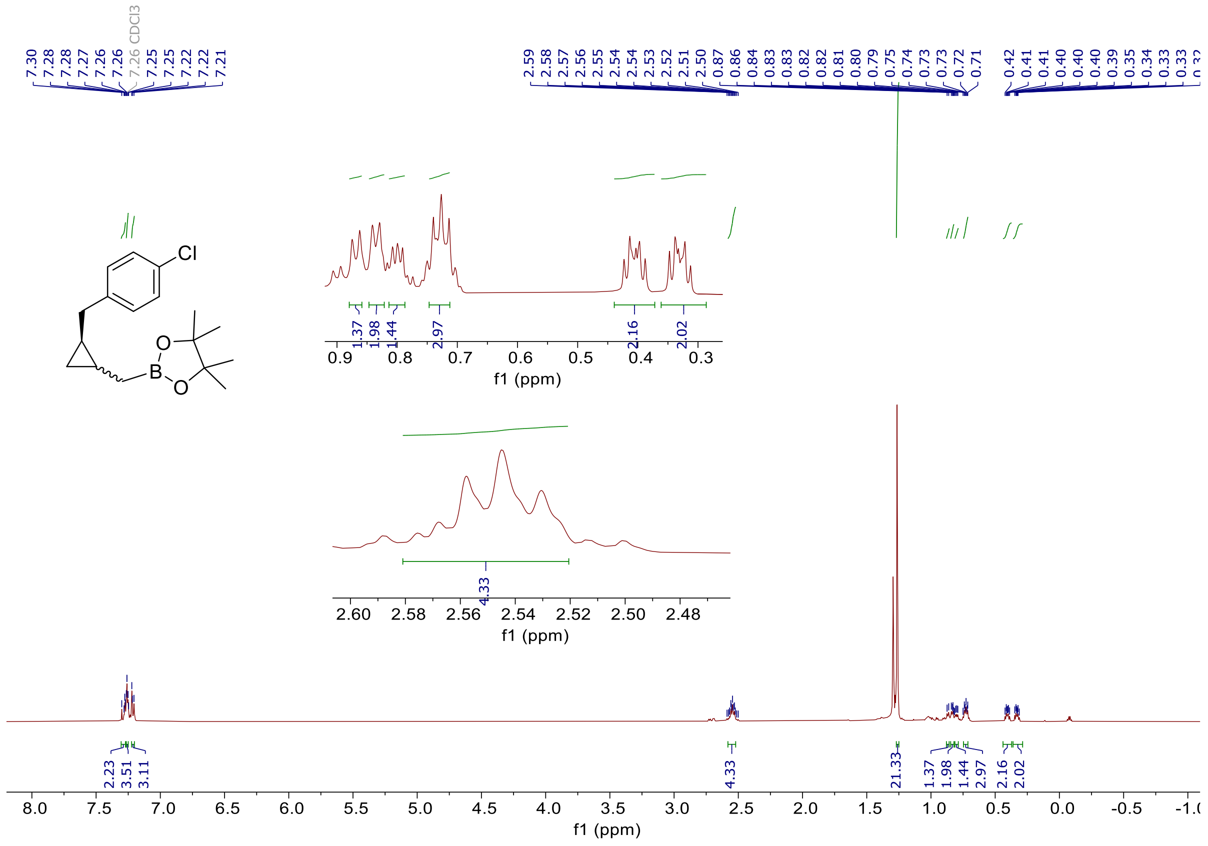

^{13}C NMR (126 MHz, $CDCl_3$) of compound **1g**

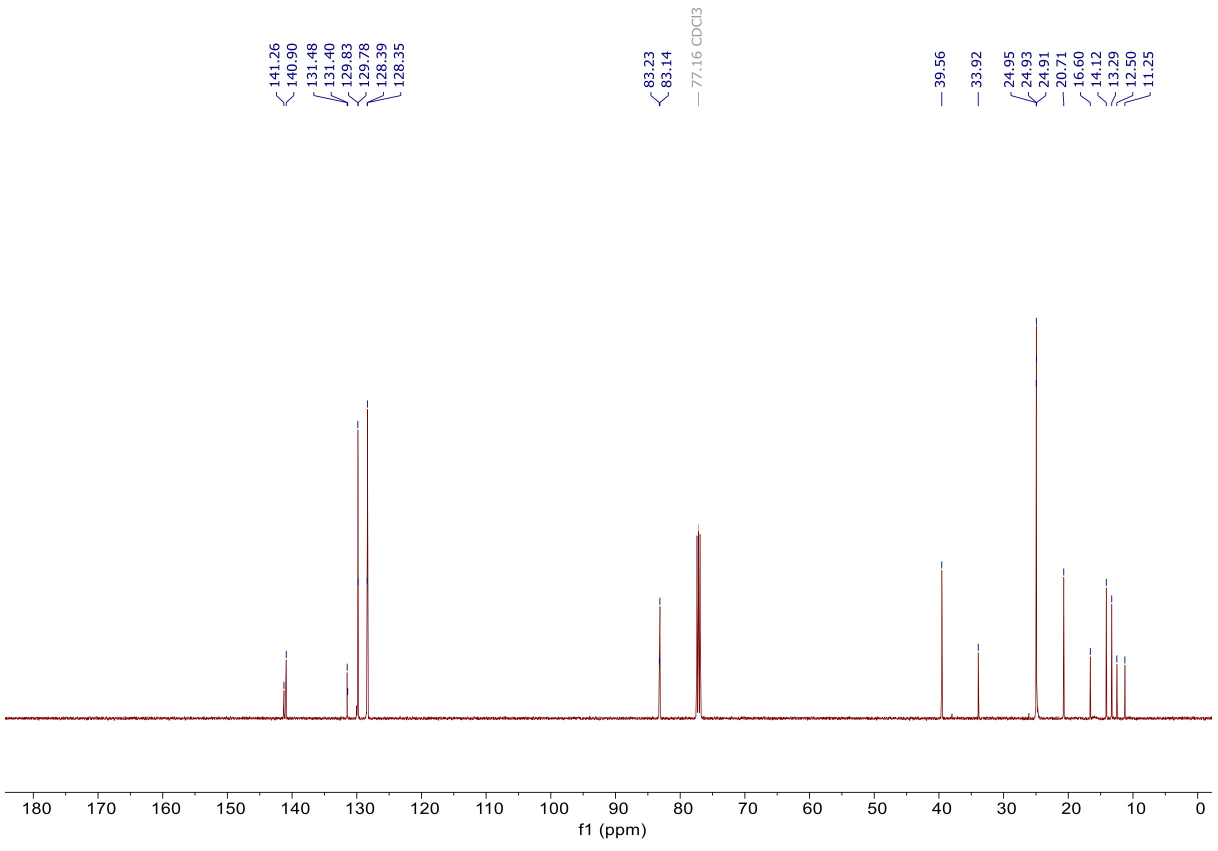

^{11}B NMR (160 MHz, $CDCl_3$) of compound **1g**

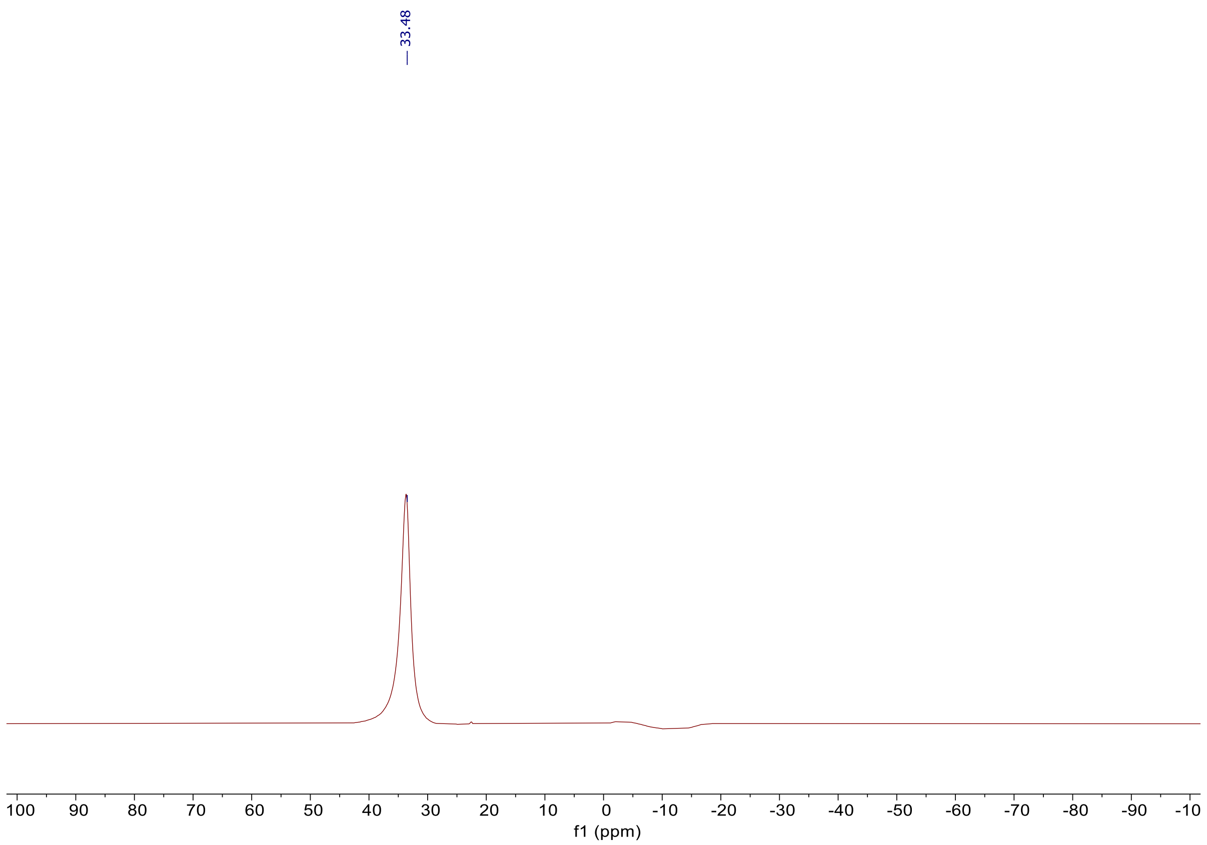

^{1}H NMR (500 MHz, $CDCl_3$) of compound **1h**

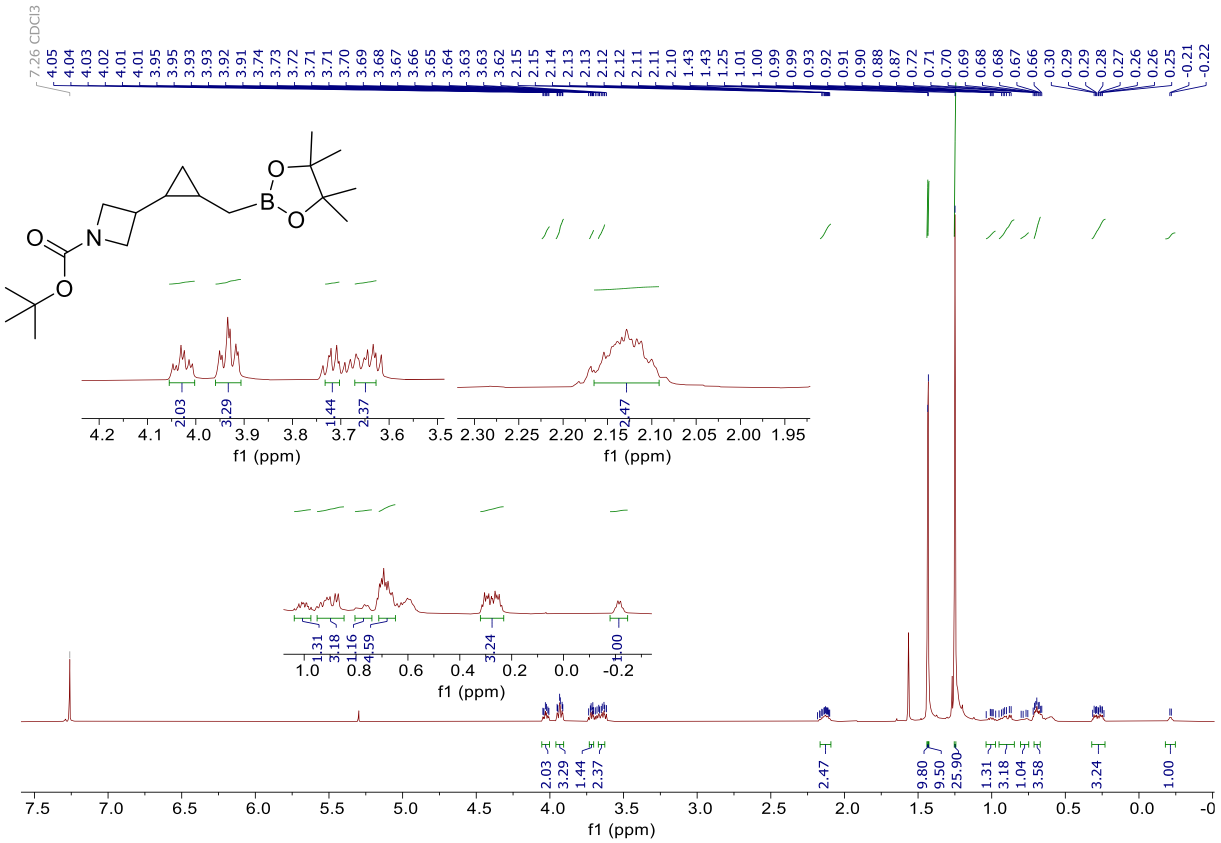

^{13}**C NMR** (126 MHz, $CDCl_3$) of compound **1h**

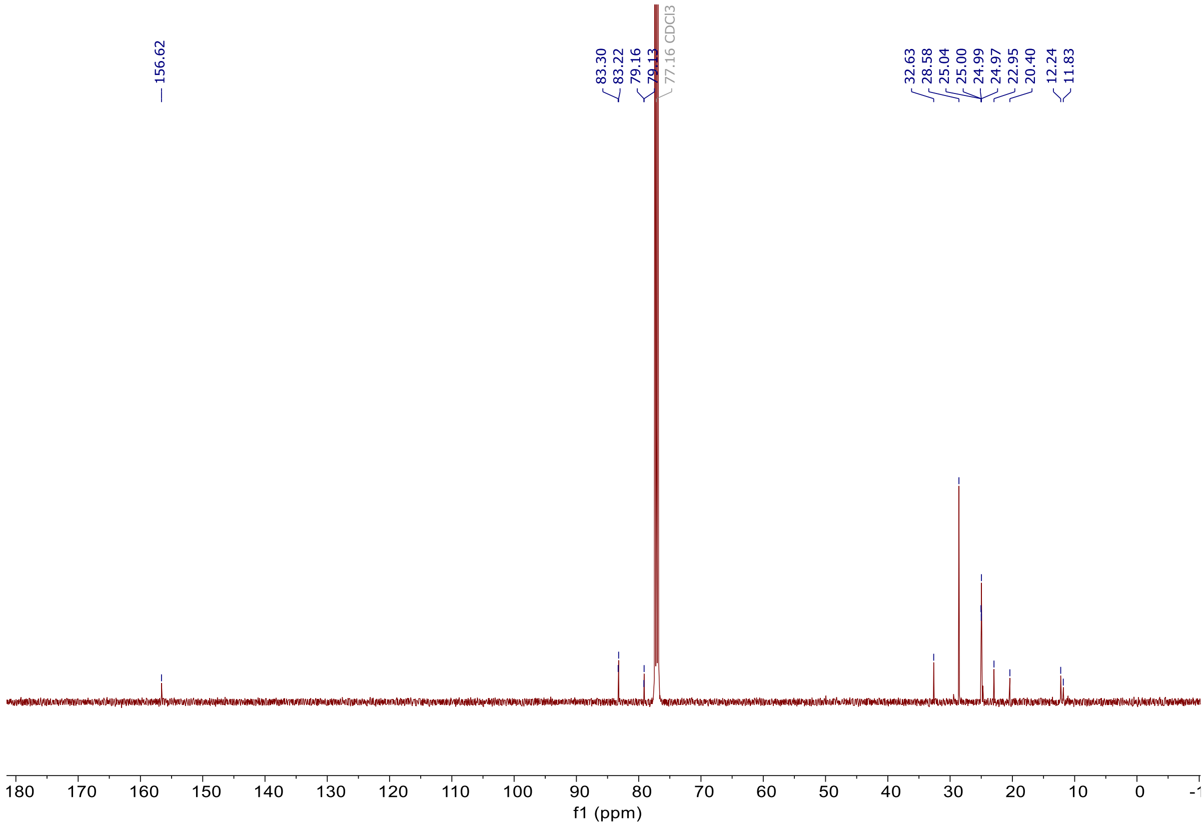

^{11}**B NMR** (160 MHz, $CDCl_3$) of compound **1h**

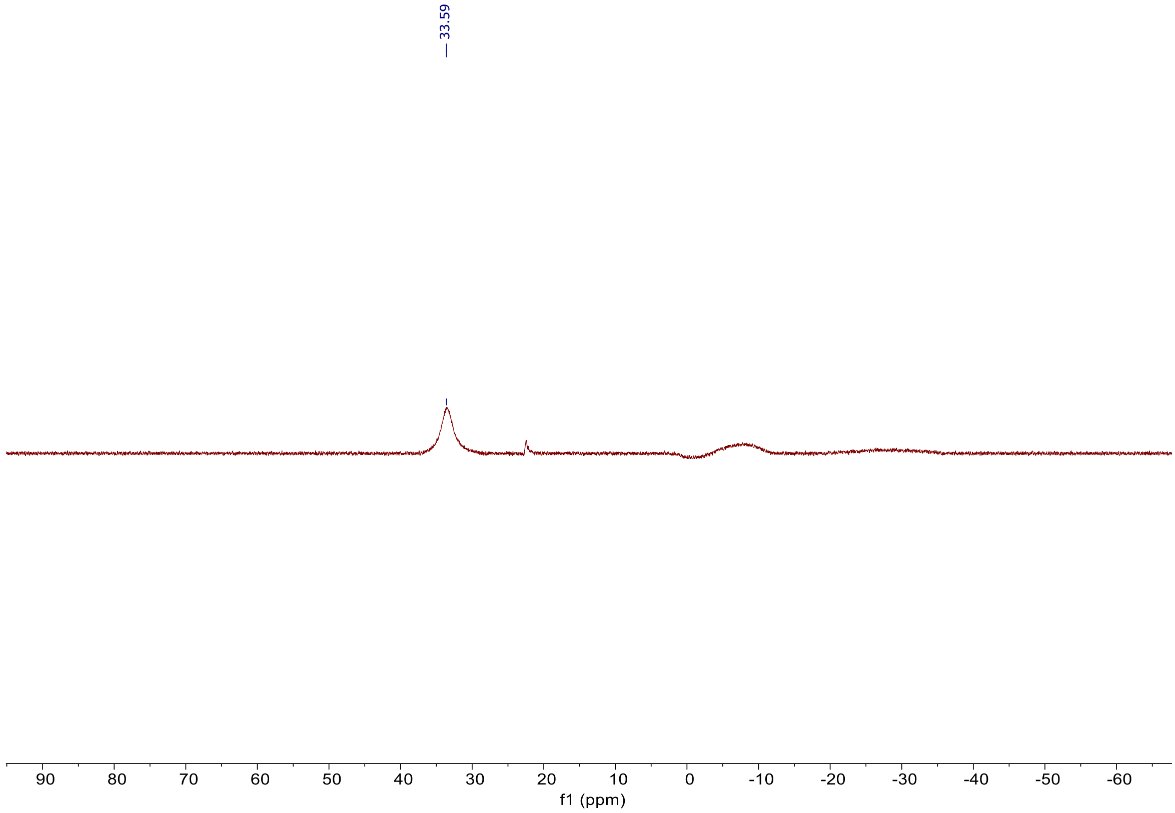

11.2. NMR Data: Negishi cross-coupling aryl halide's products

^{1}H NMR (500 MHz, $CDCl_3$) of compound **2a**

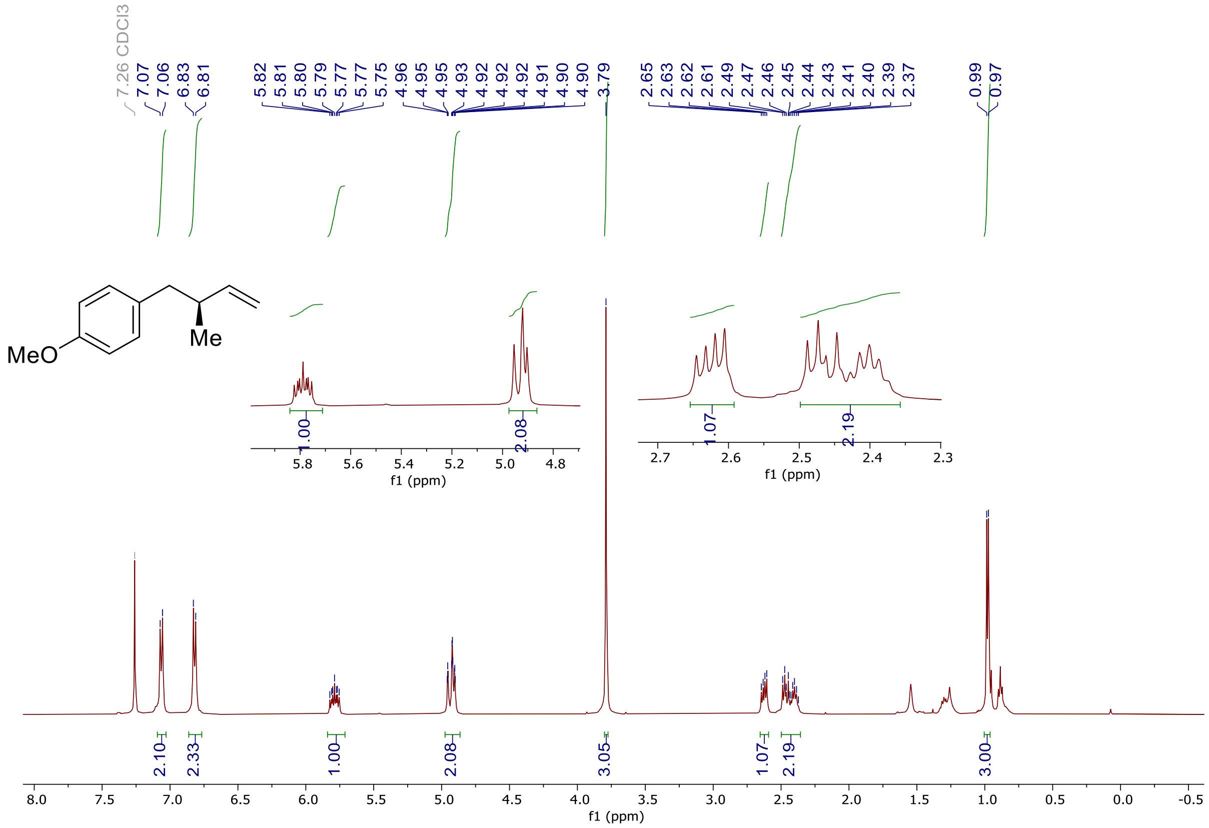

^{13}C NMR (126 MHz, $CDCl_3$) of compound **2a**

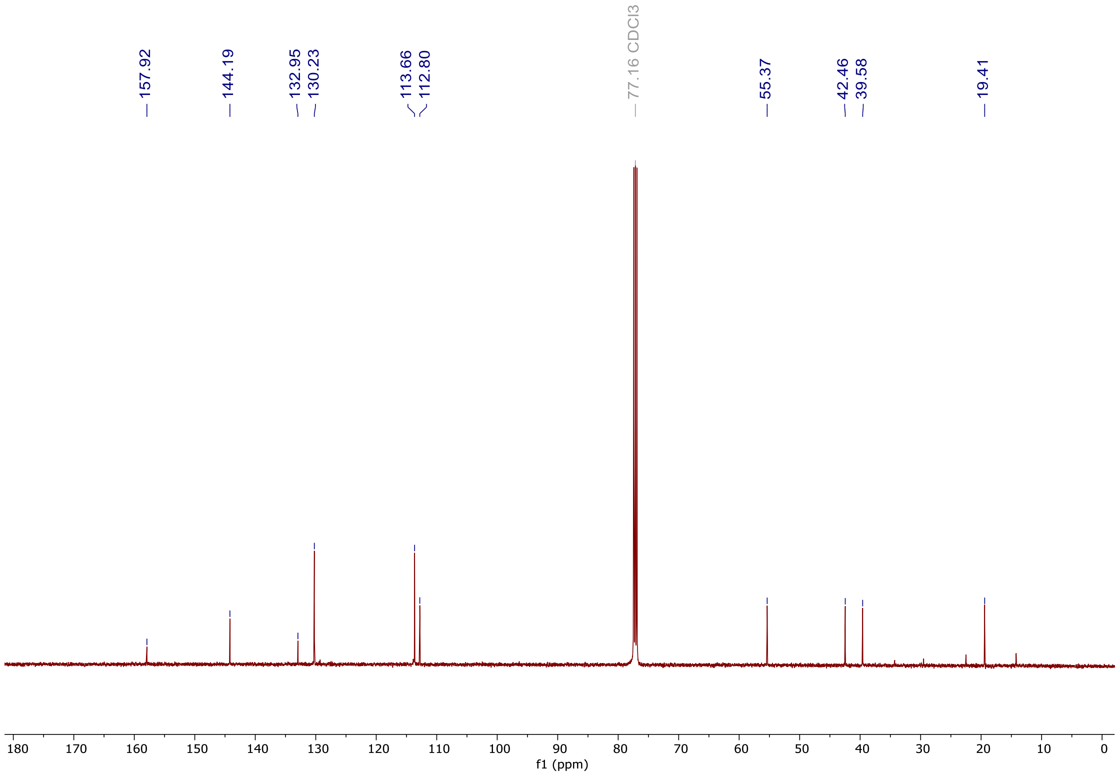

^{1}H NMR (300 MHz, $CDCl_3$) of compound **2b**

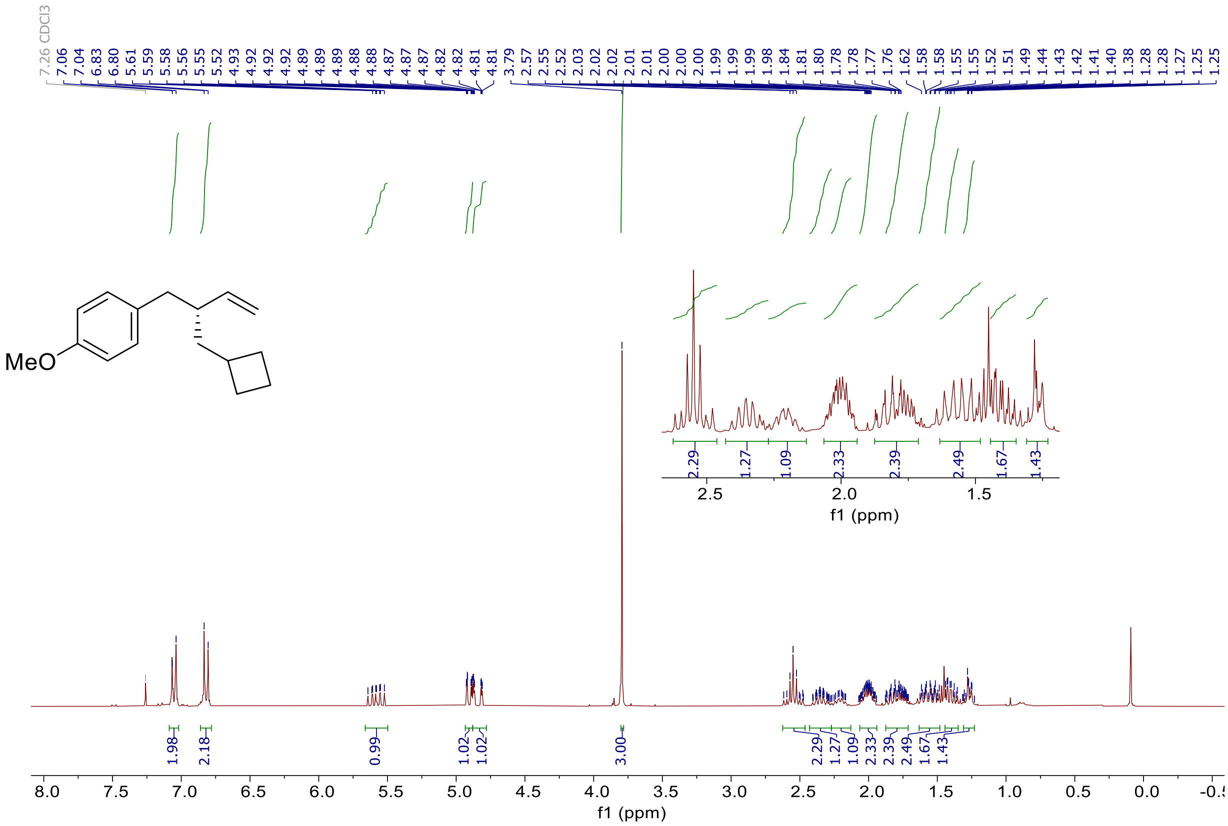

^{13}C NMR (76 MHz, $CDCl_3$) of compound **2b**

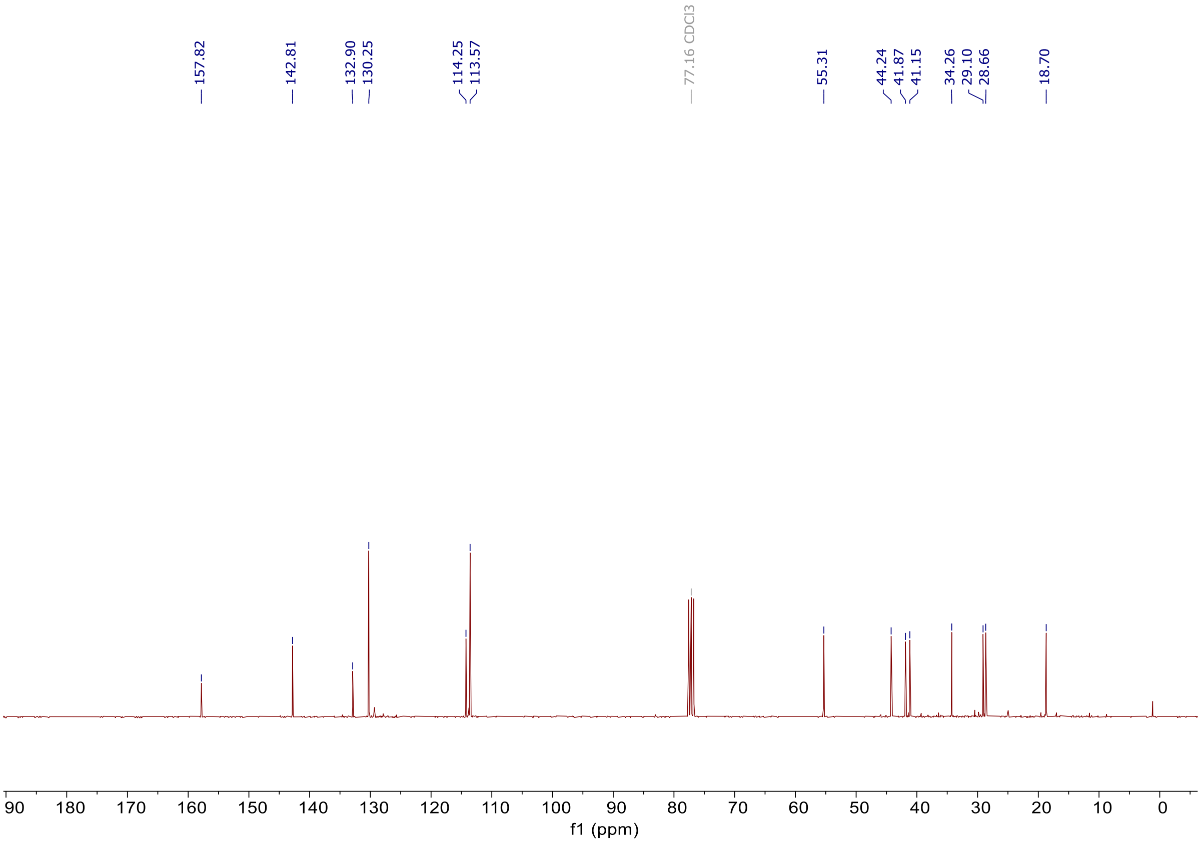

^{1}H NMR (500 MHz, $CDCl_3$) of compound **2c**

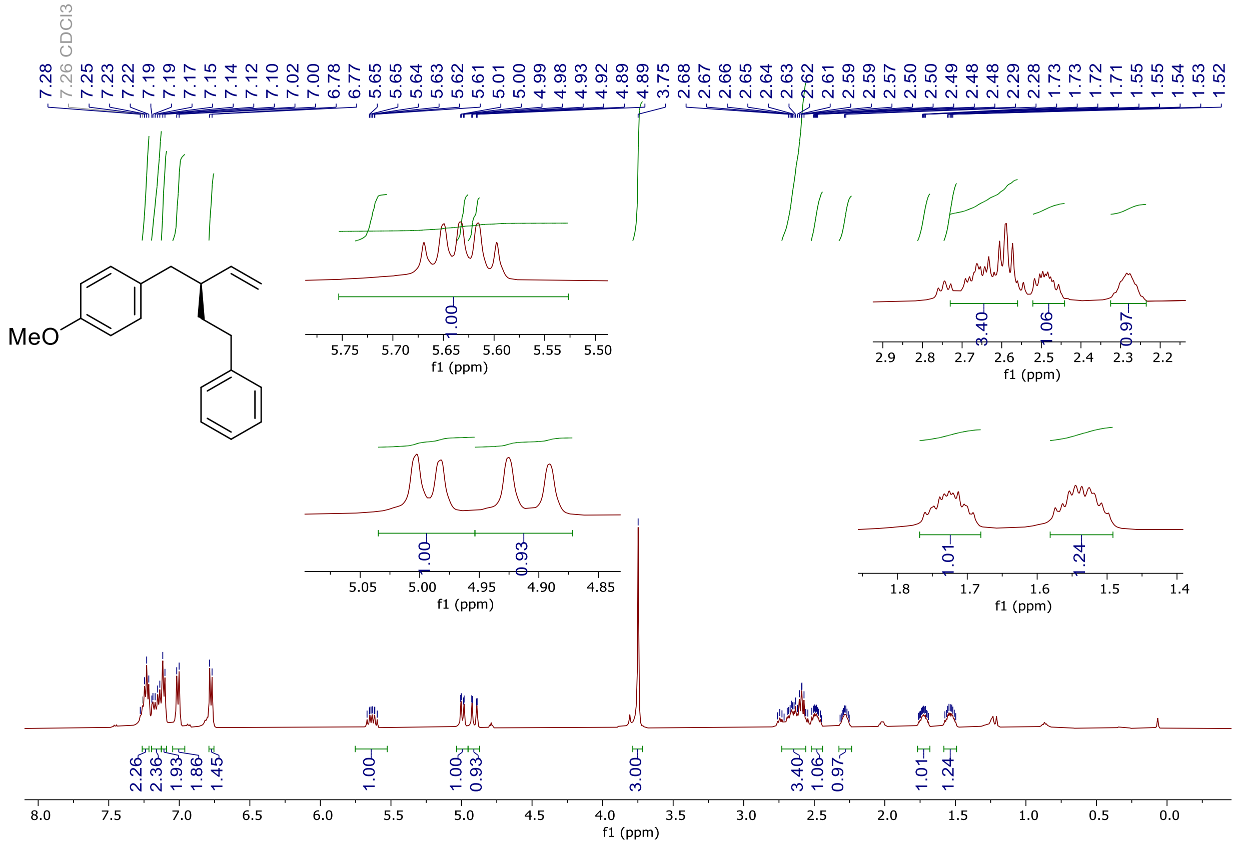

^{13}C NMR (126 MHz, $CDCl_3$) of compound **2c**

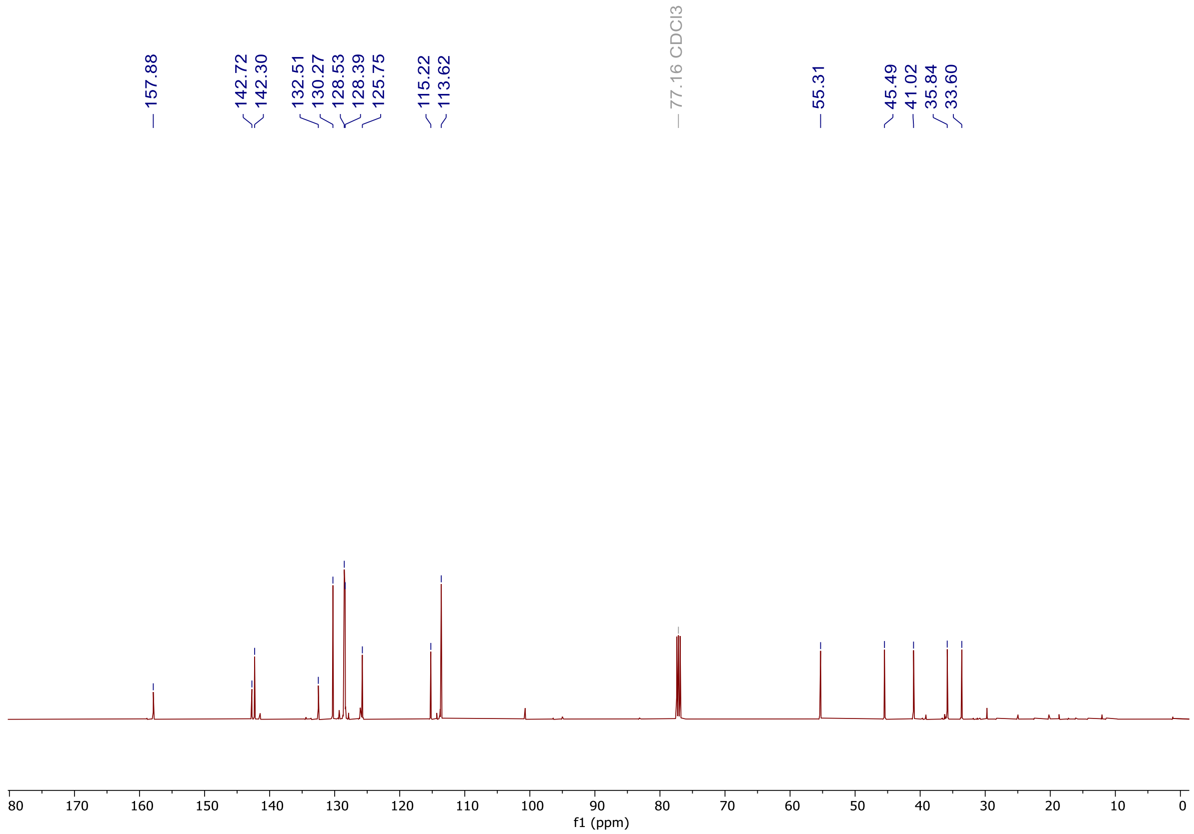

^{1}H NMR (300 MHz, $CDCl_3$) of compound **2d**

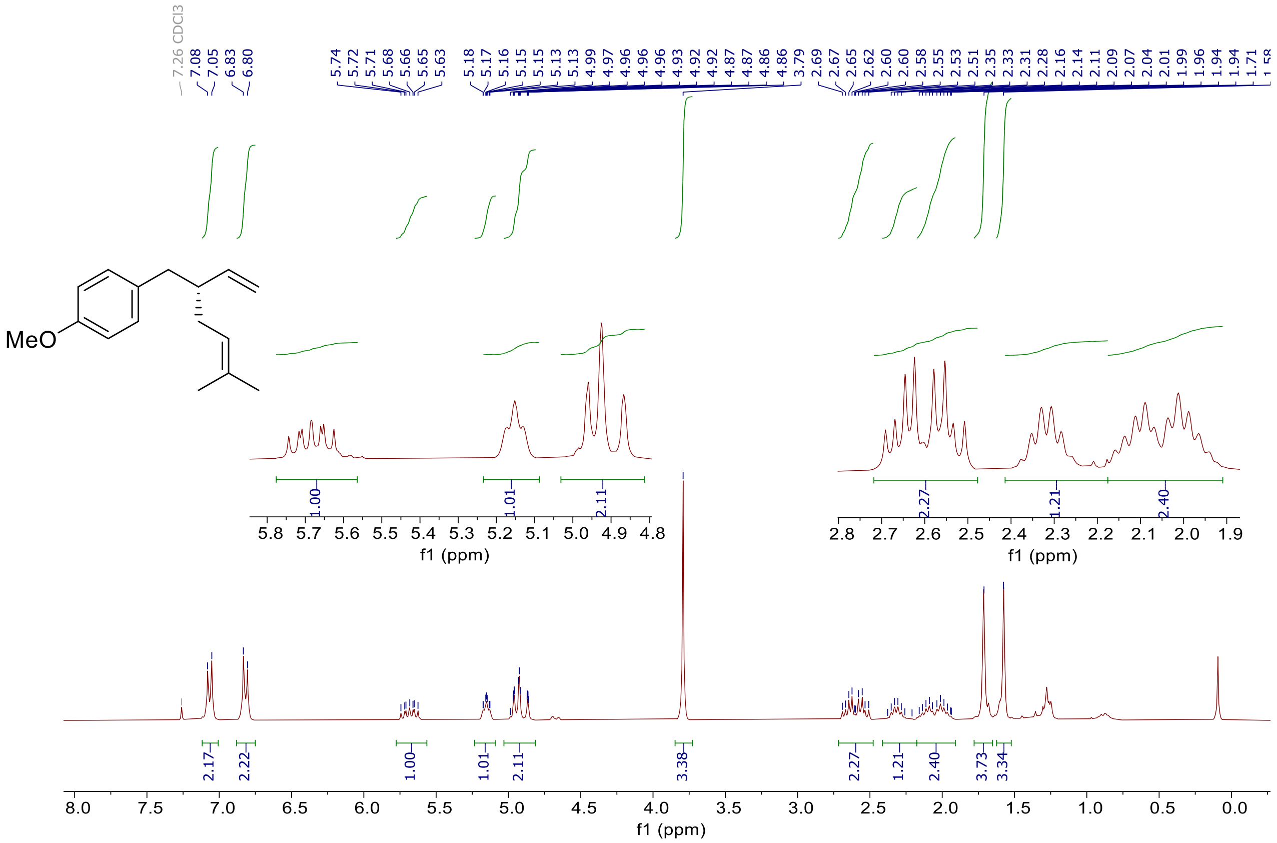

^{13}C NMR (76 MHz, $CDCl_3$) of compound **2d**

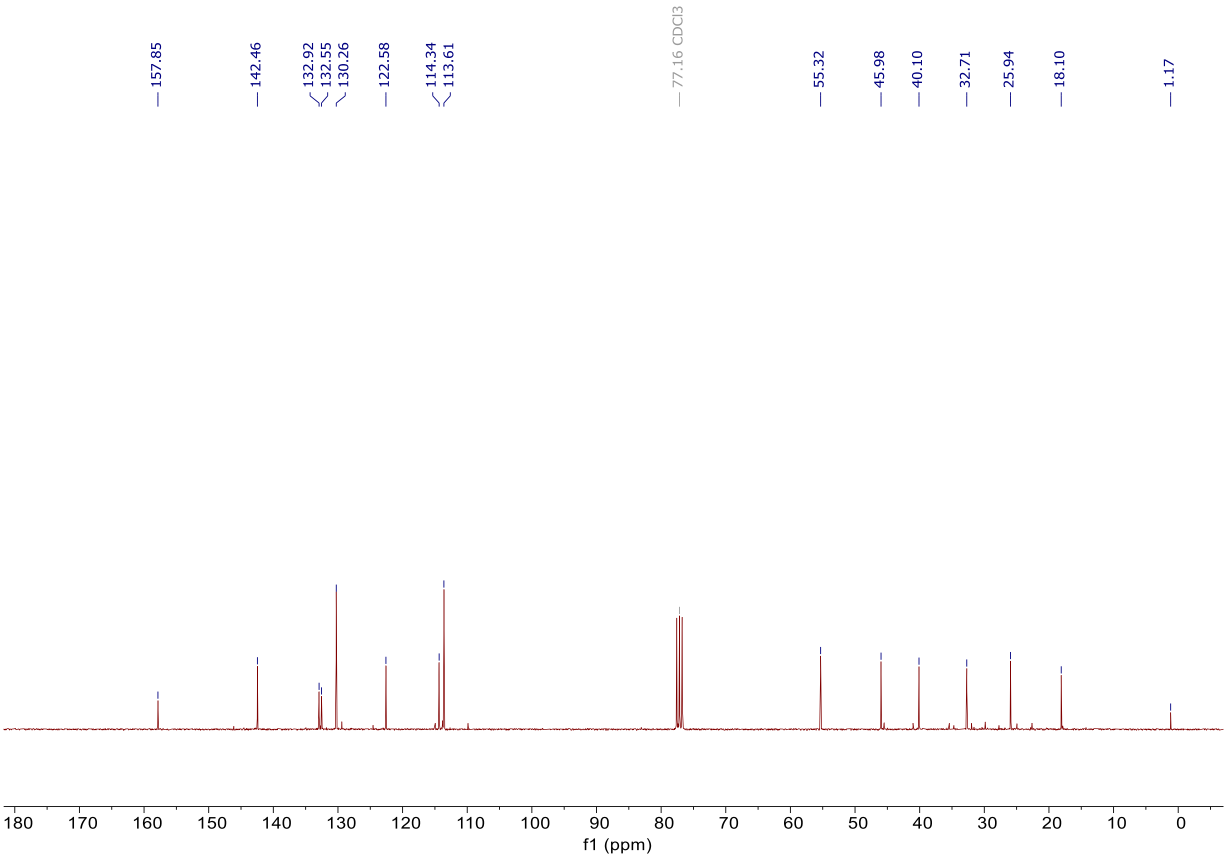

^{1}H NMR (300 MHz, $CDCl_3$) of compound **2e**

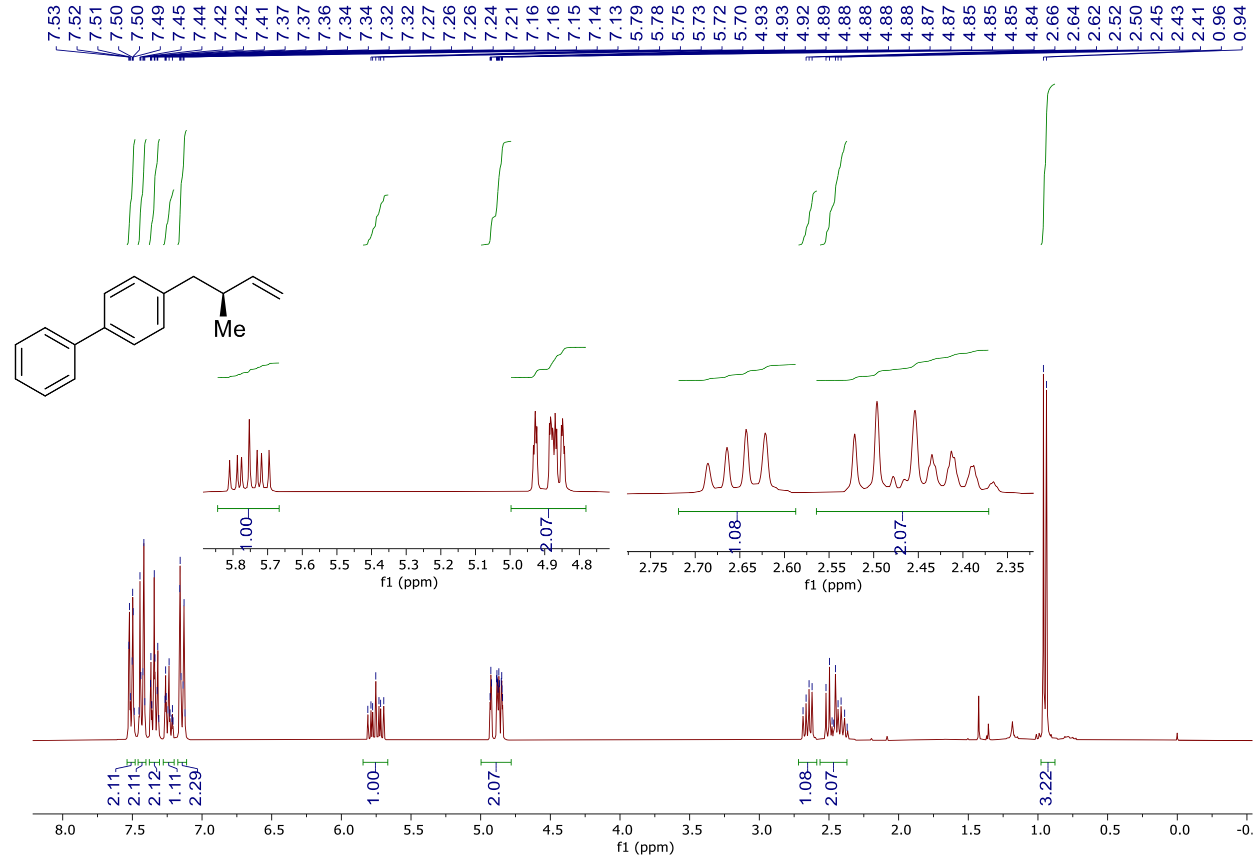

^{13}C NMR (76 MHz, $CDCl_3$) of compound **2e**

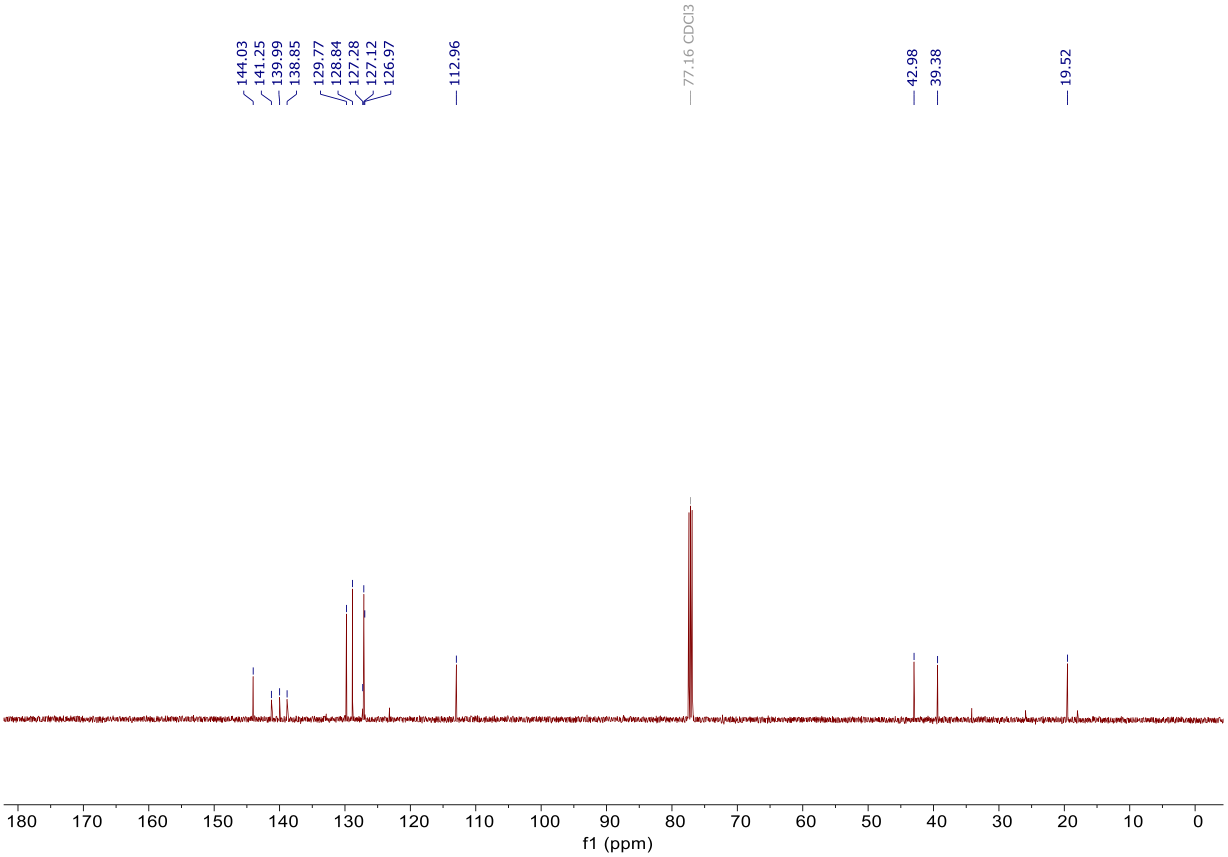

^{1}H NMR (500 MHz, $CDCl_3$) of compound **2f**

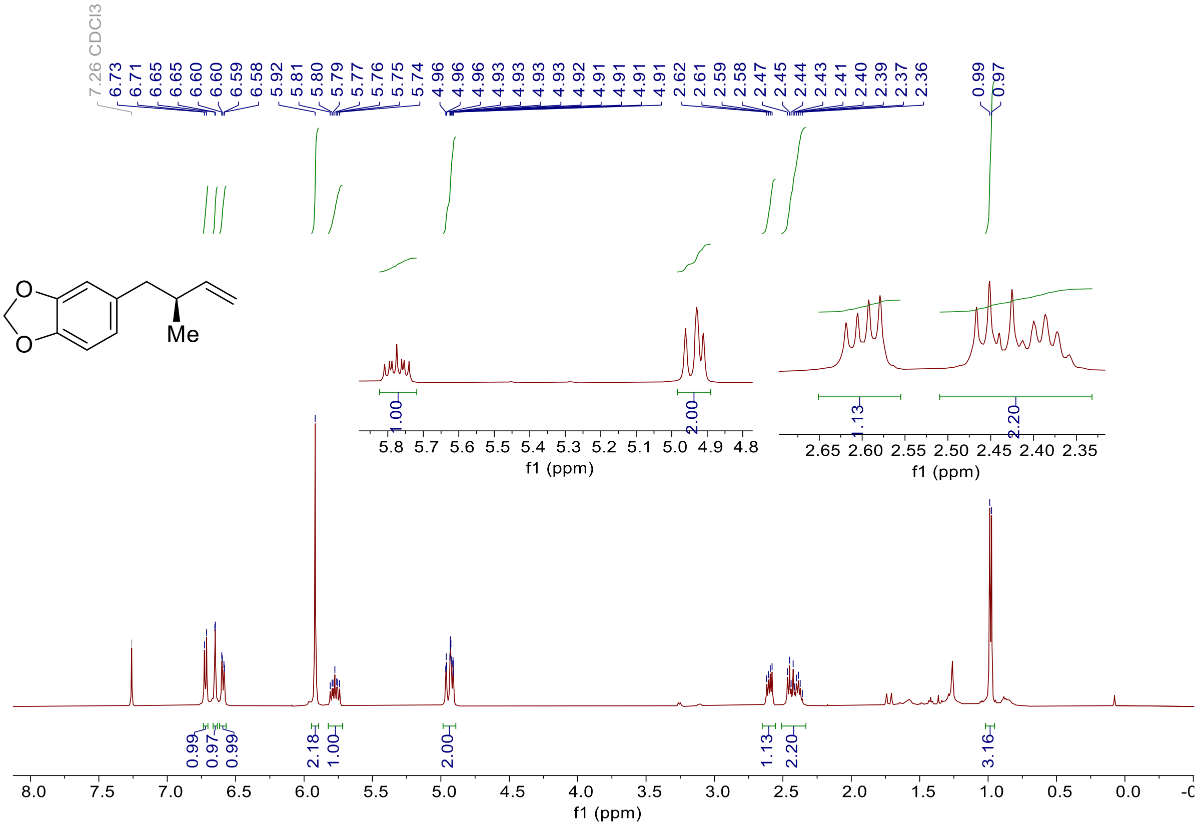

^{13}C NMR (126 MHz, $CDCl_3$) of compound **2f**

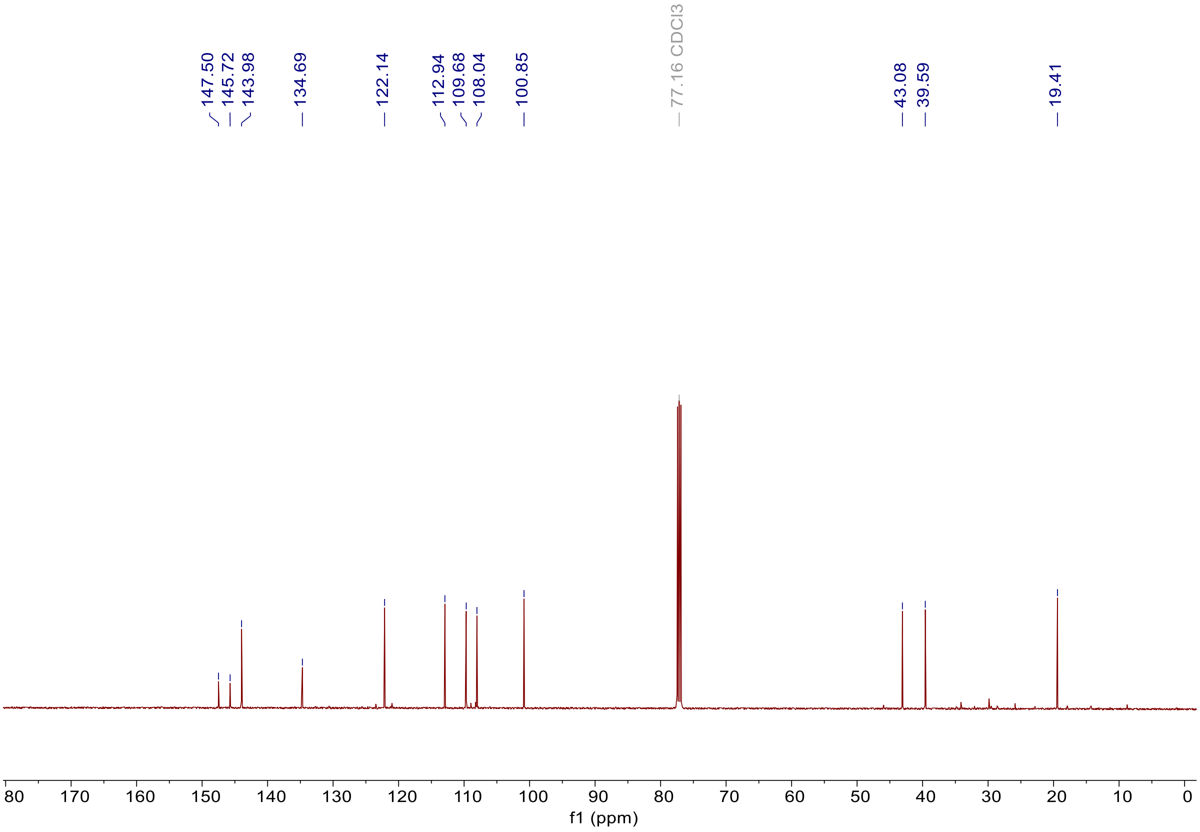

^{1}H NMR (500 MHz, $CDCl_3$) of compound **2g**

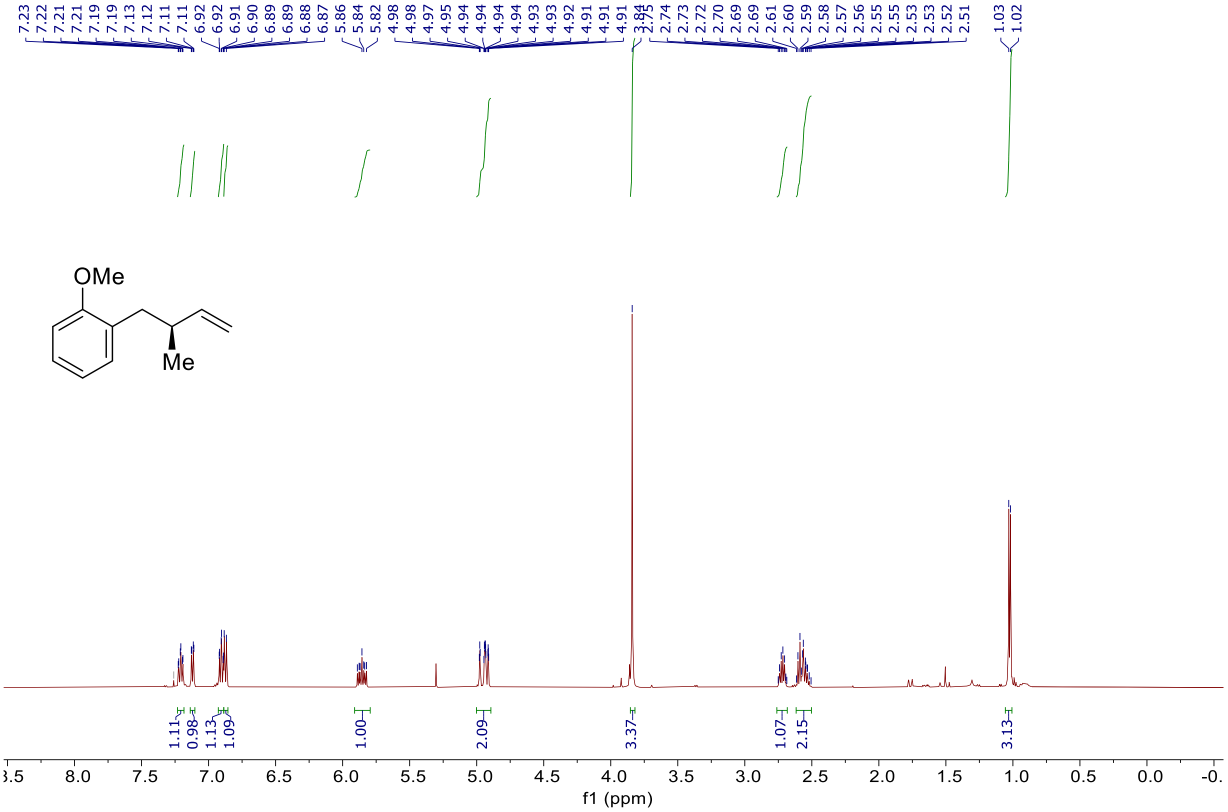

^{13}C NMR (126 MHz, $CDCl_3$) of compound **2g**

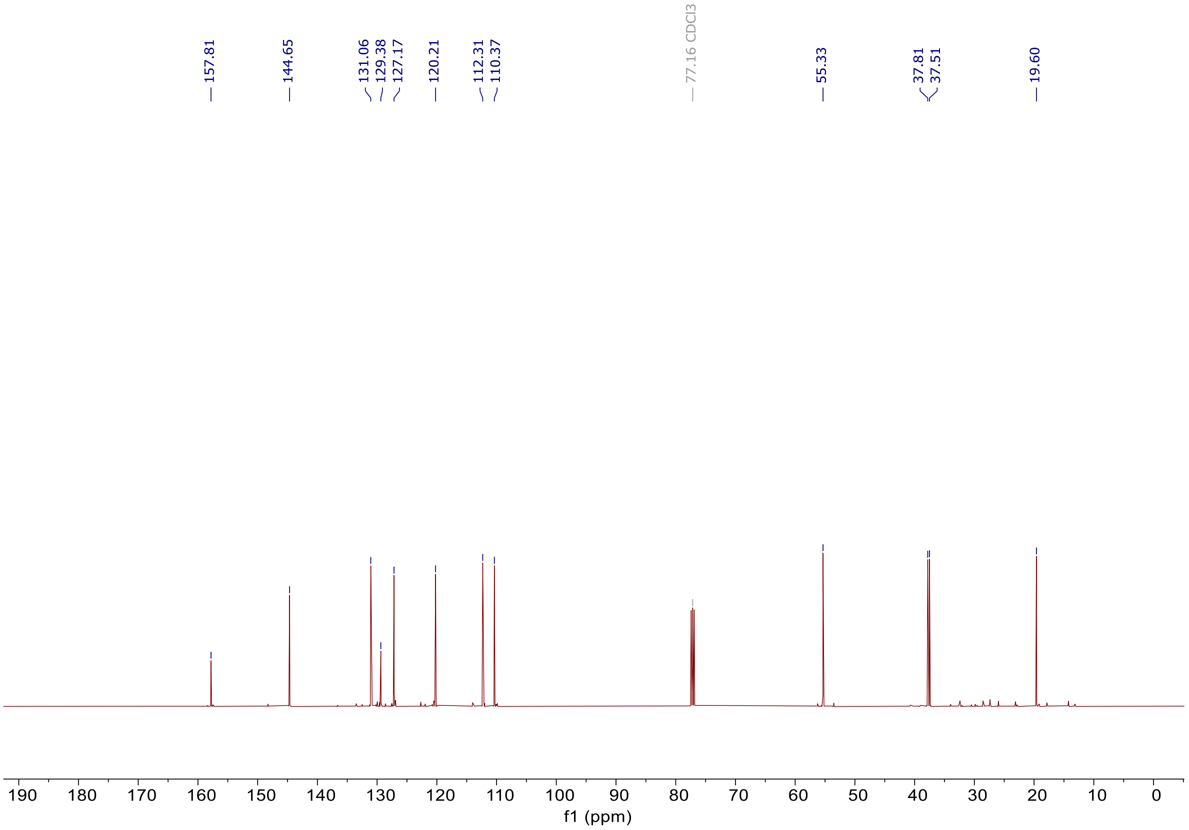

^{1}H NMR (500 MHz, $CDCl_3$) of compound **2h**

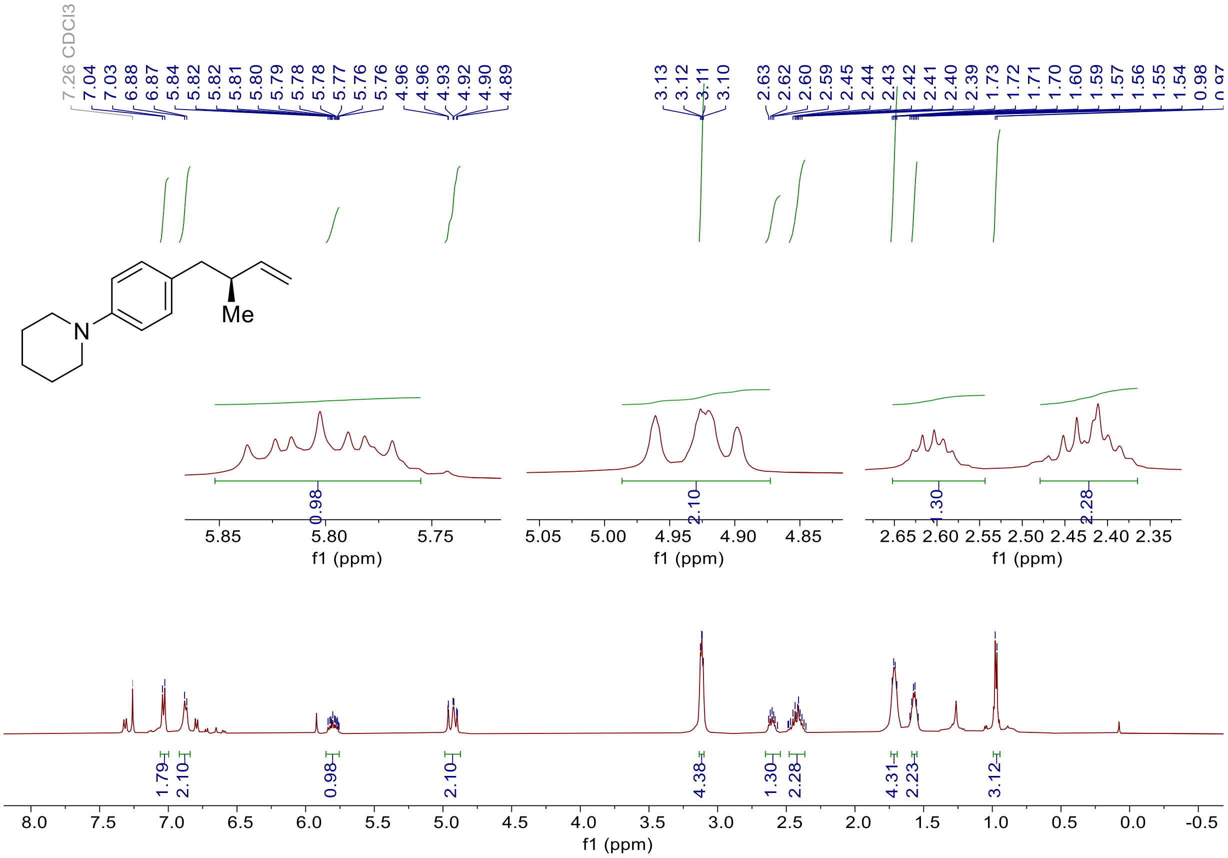

^{13}C NMR (126 MHz, $CDCl_3$) of compound **2h**

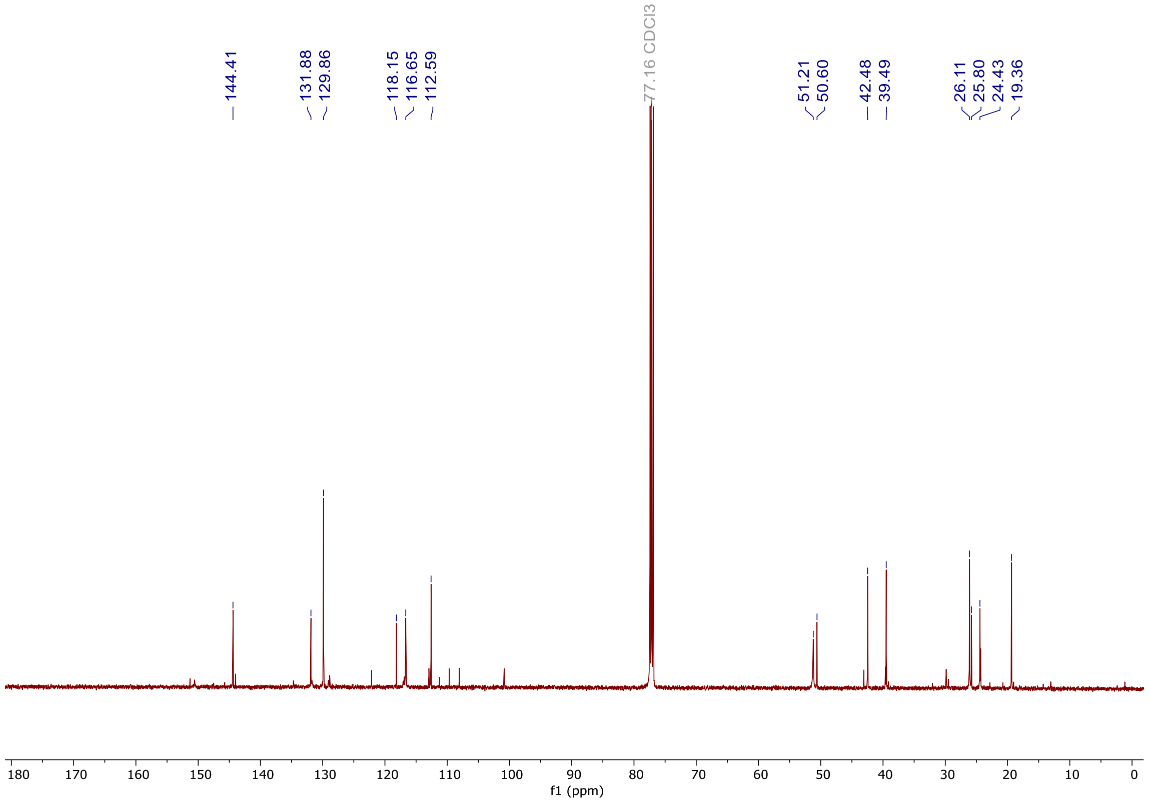

^{1}H NMR (300 MHz, $CDCl_3$) of compound **2i**

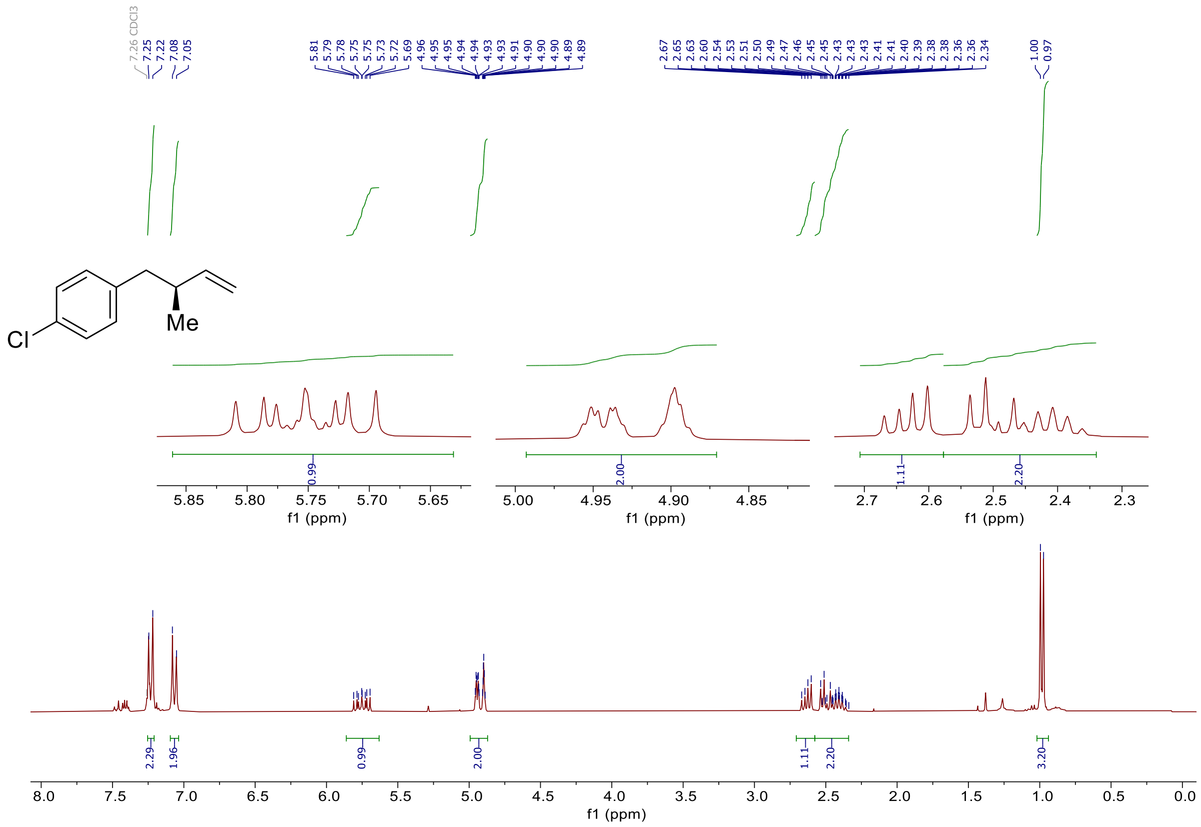

^{13}C NMR (126 MHz, $CDCl_3$) of compound **2i**

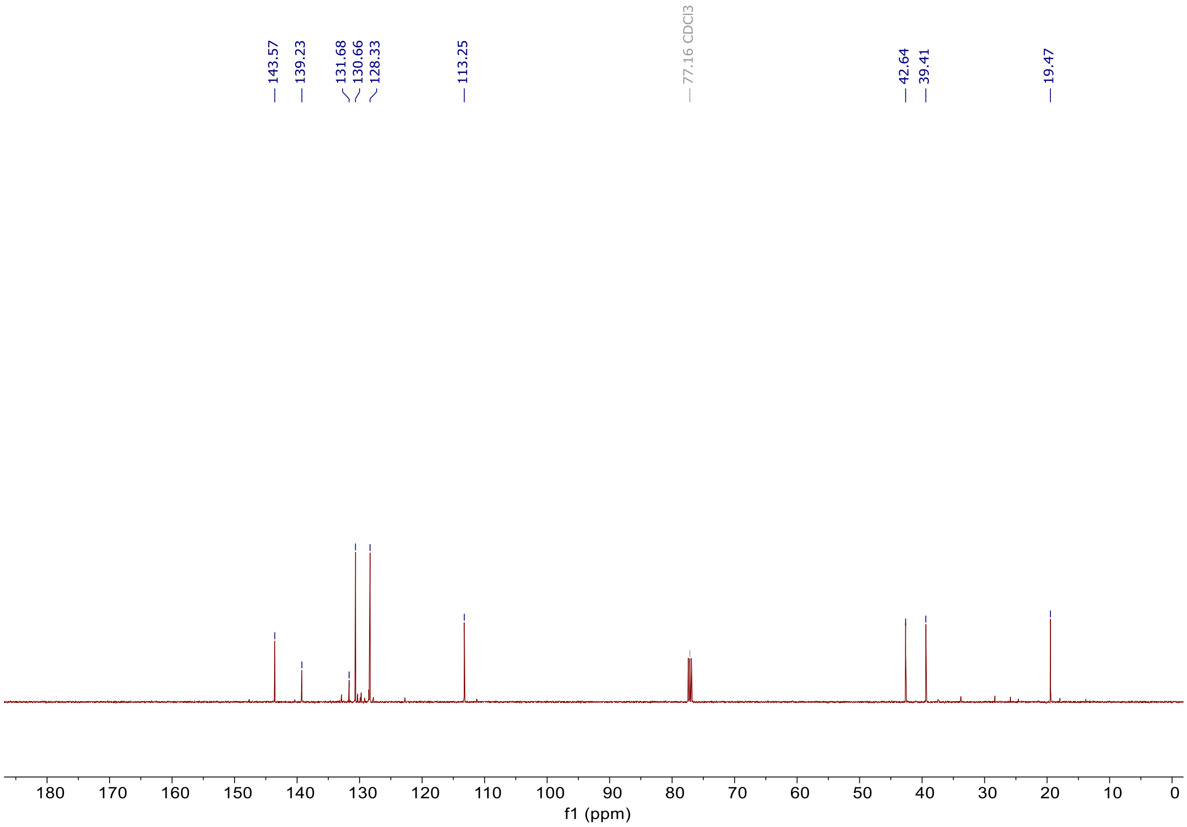

^{1}H NMR (300 MHz, $CDCl_3$) of compound **2j** (impurities in the aromatic region due to the close polarity of the SM and product)

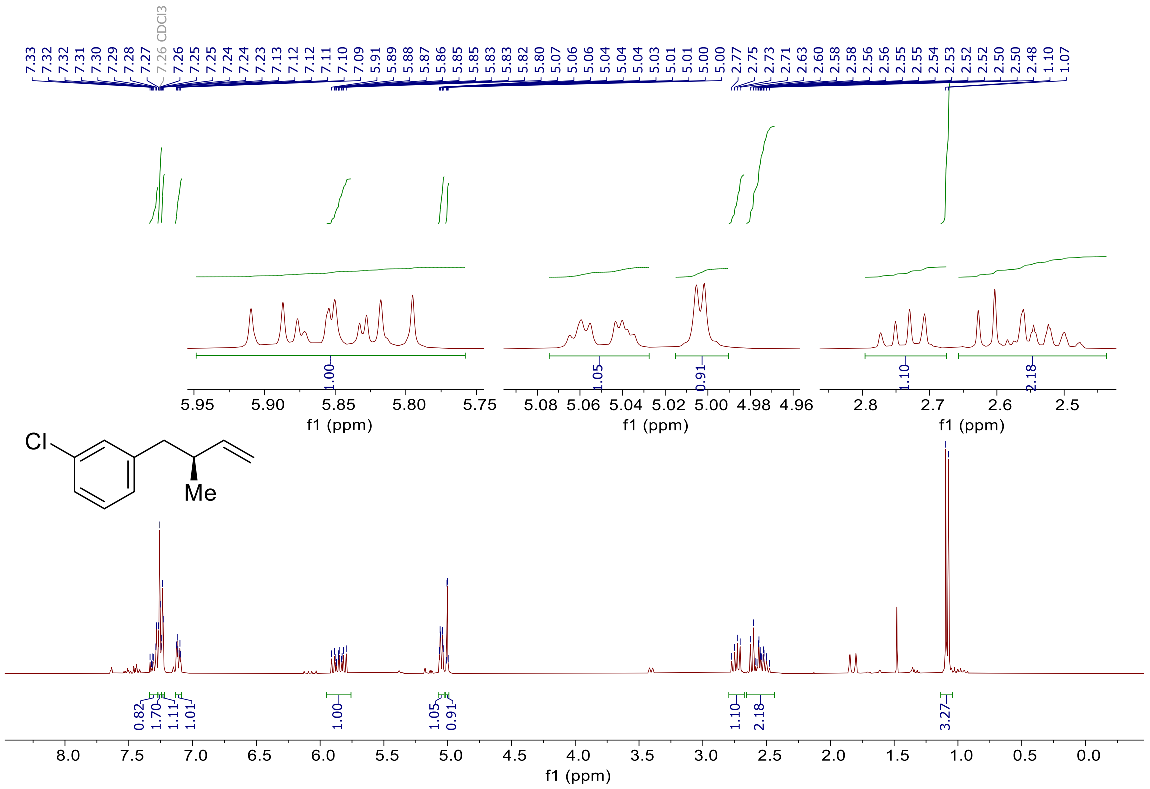

^{13}C NMR (75 MHz, $CDCl_3$) of compound **2j**

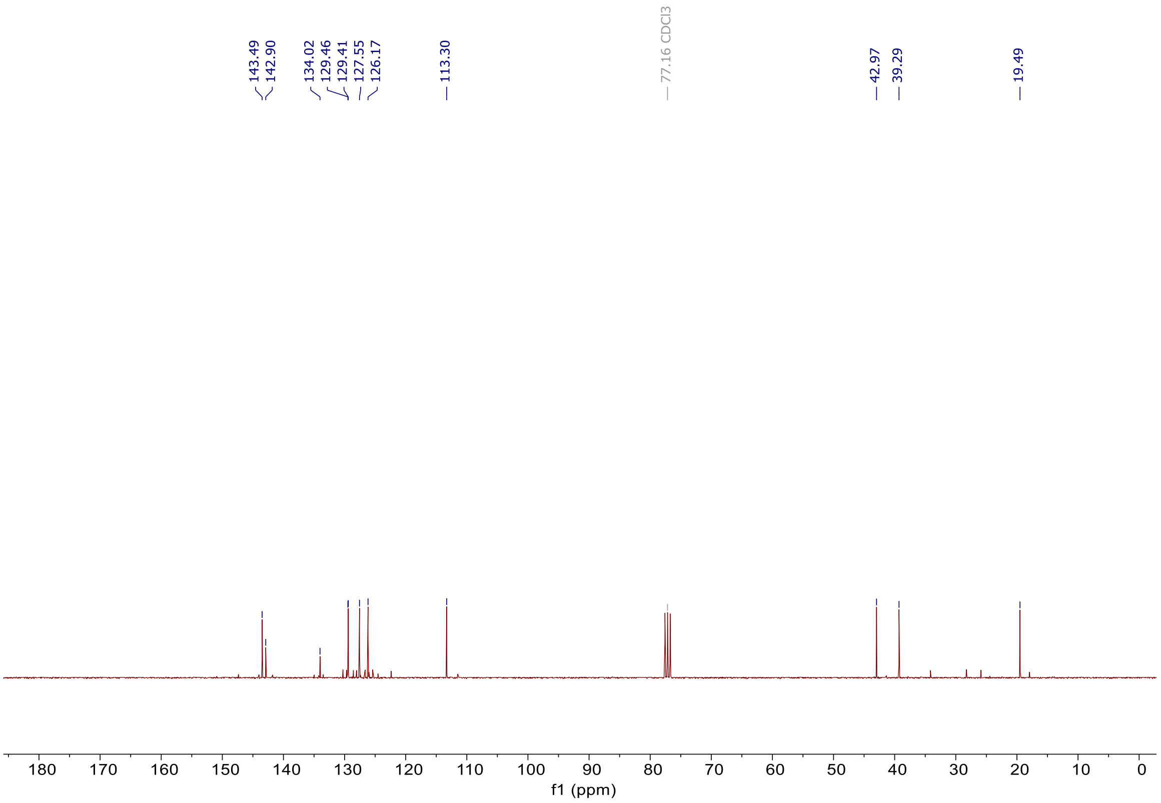

^{1}H NMR (300 MHz, $CDCl_3$) of compound **2k**

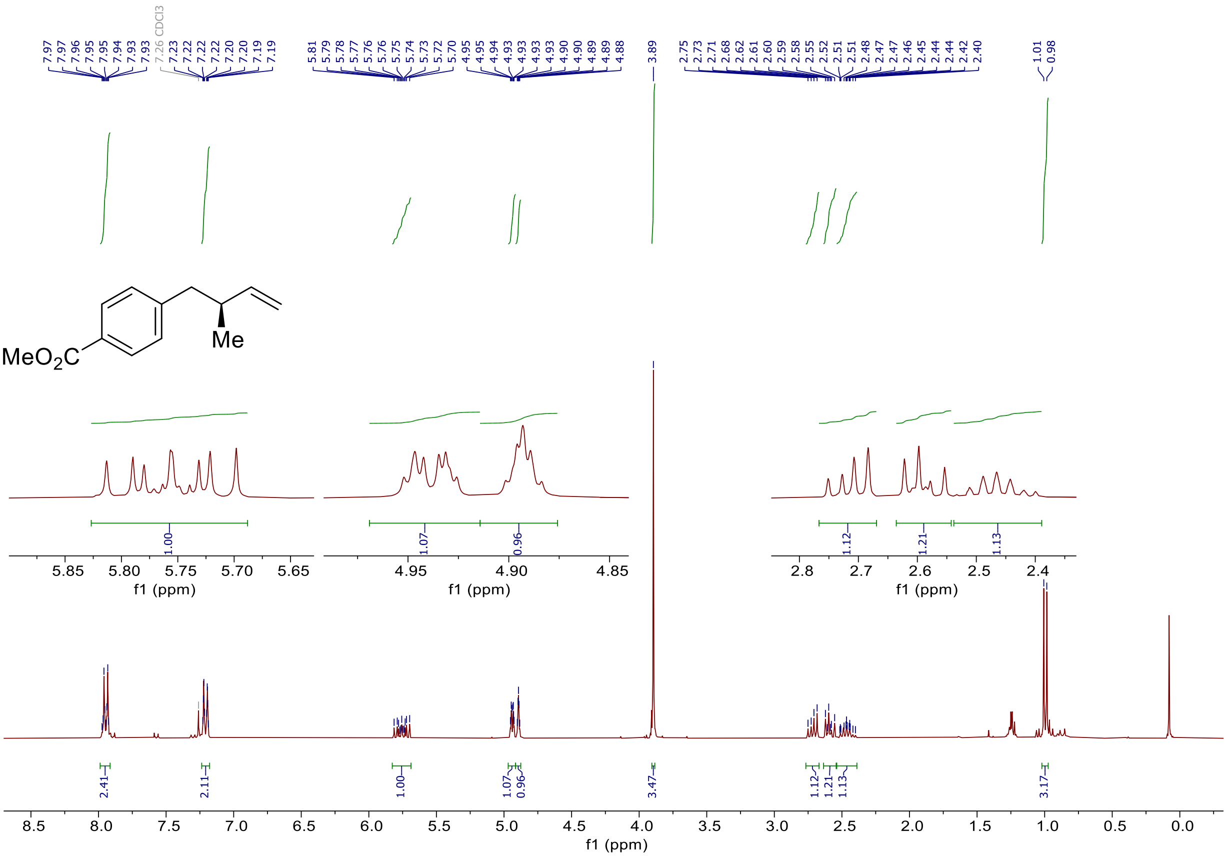

^{13}C NMR (126 MHz, $CDCl_3$) of compound **2k**

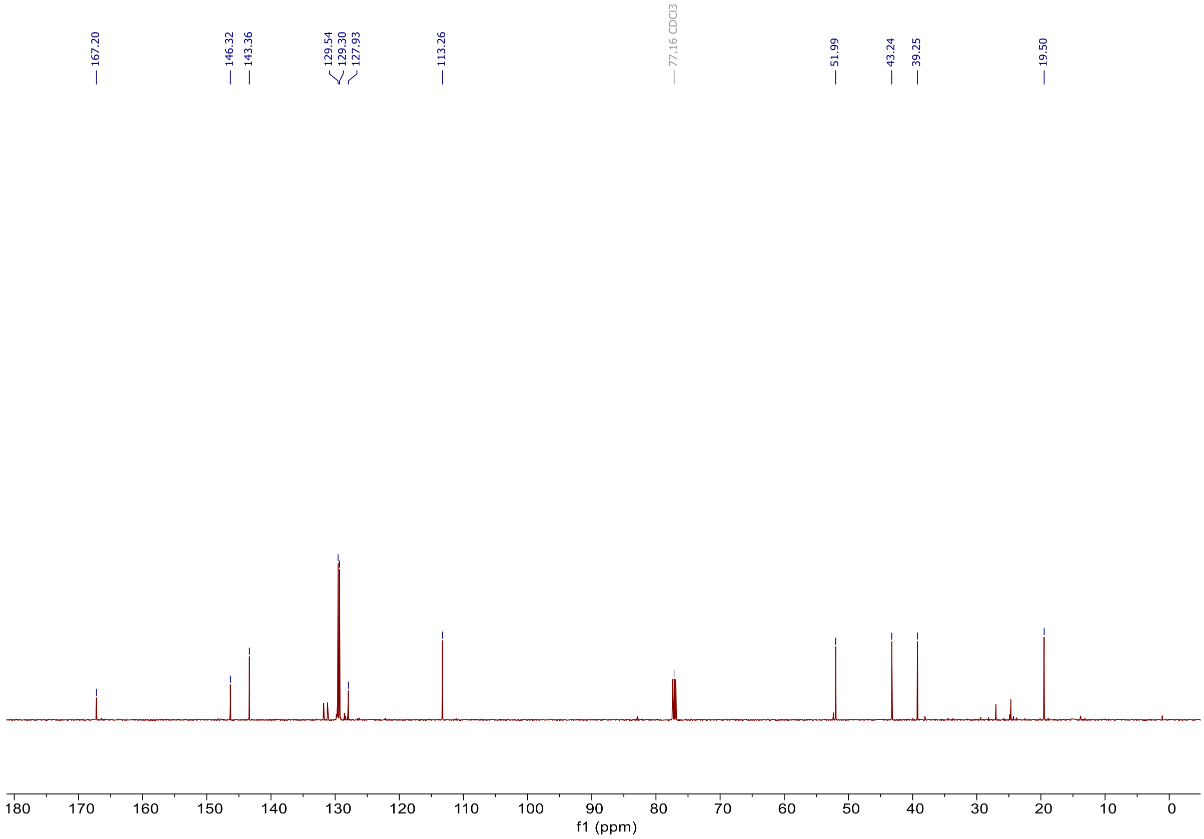

^{1}H NMR (300 MHz, $CDCl_3$) of compound **2l**

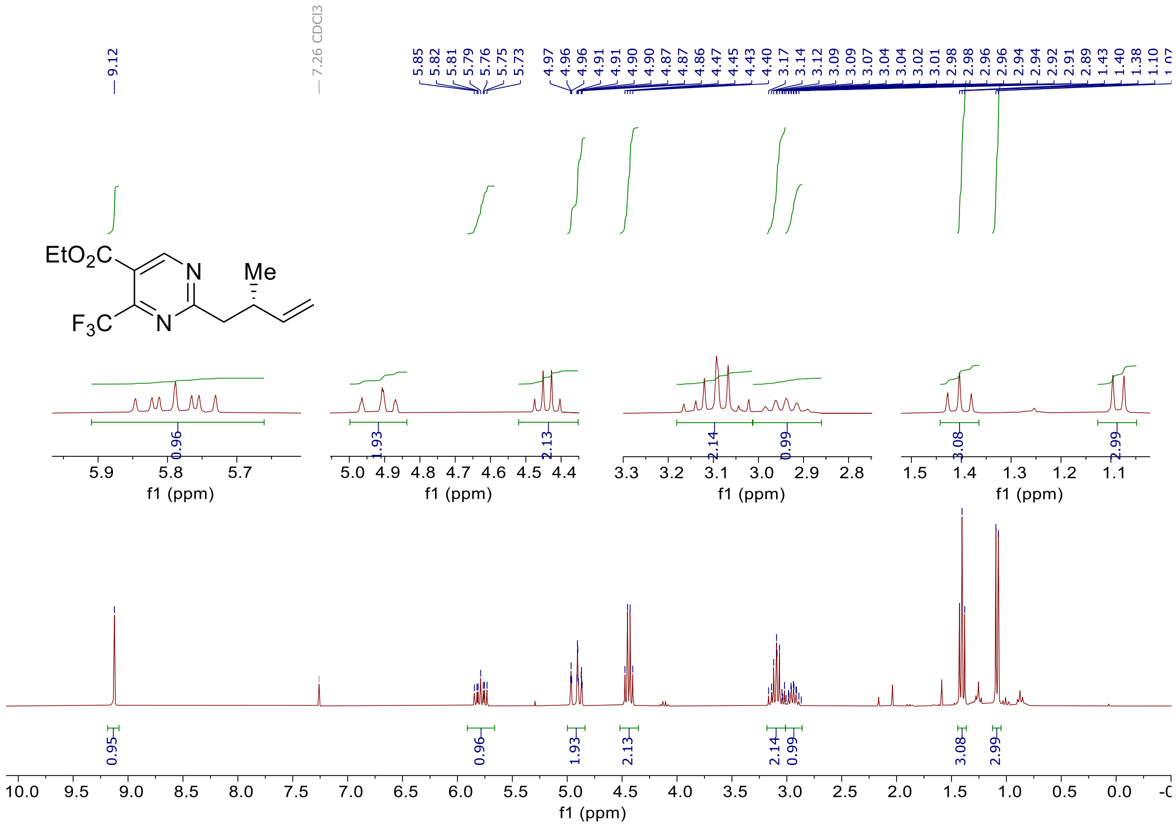

^{13}C NMR (76 MHz, $CDCl_3$) of compound **2l**

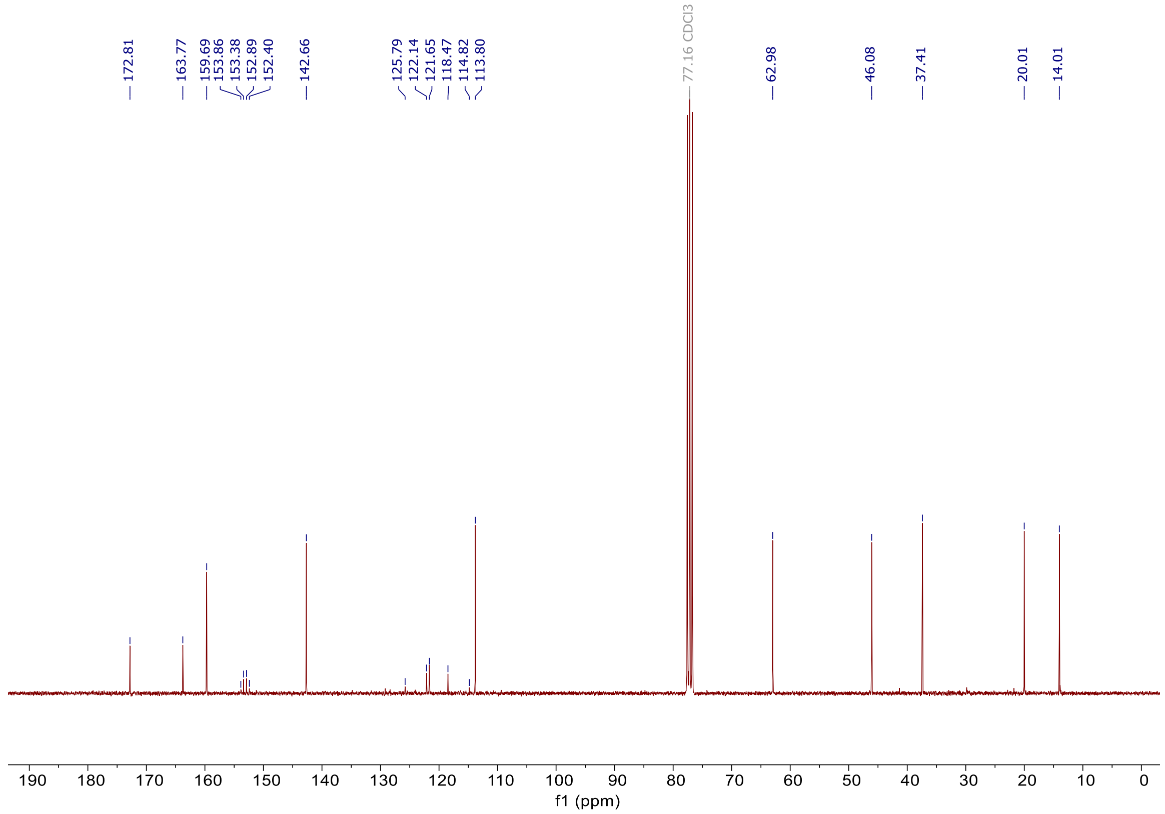

^{19}F NMR (282 MHz, $CDCl_3$) of compound **2l**

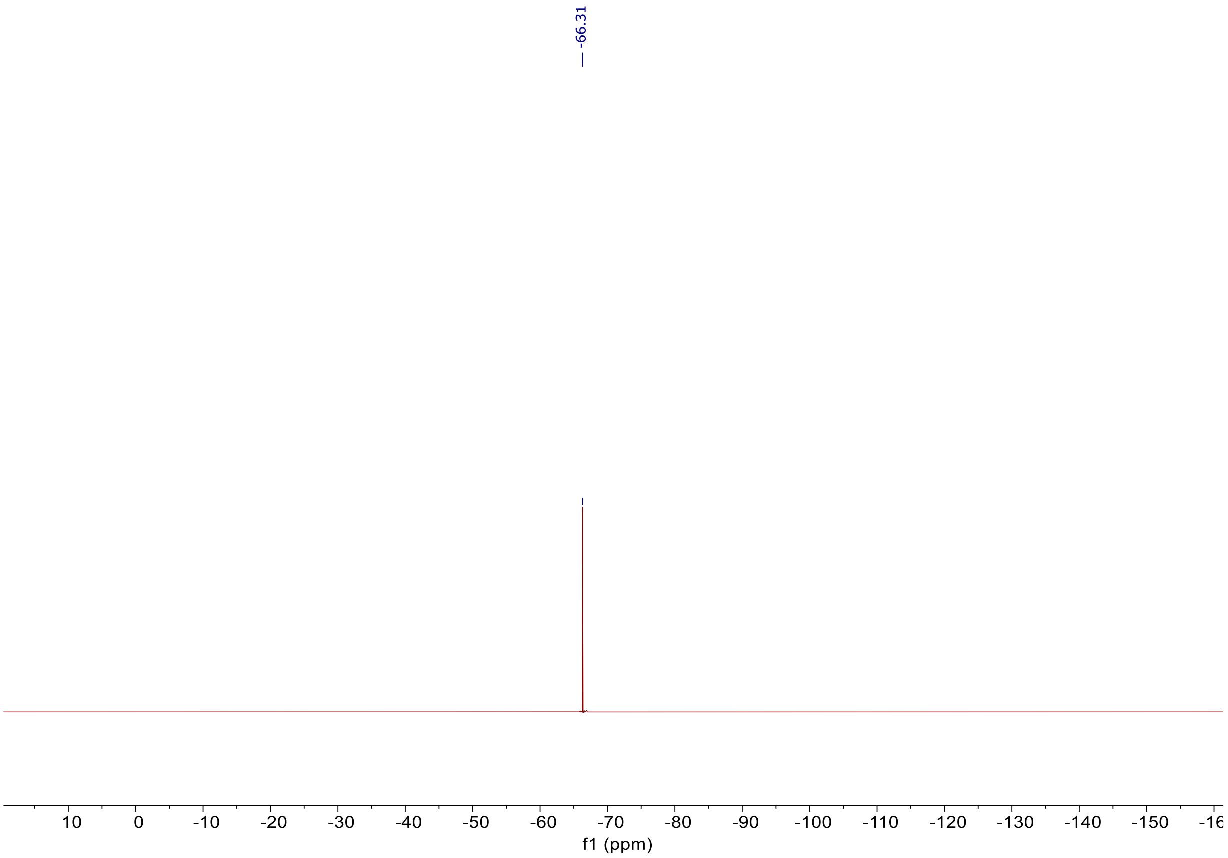

^{1}H NMR (300 MHz, $CDCl_3$) of compound **2m**

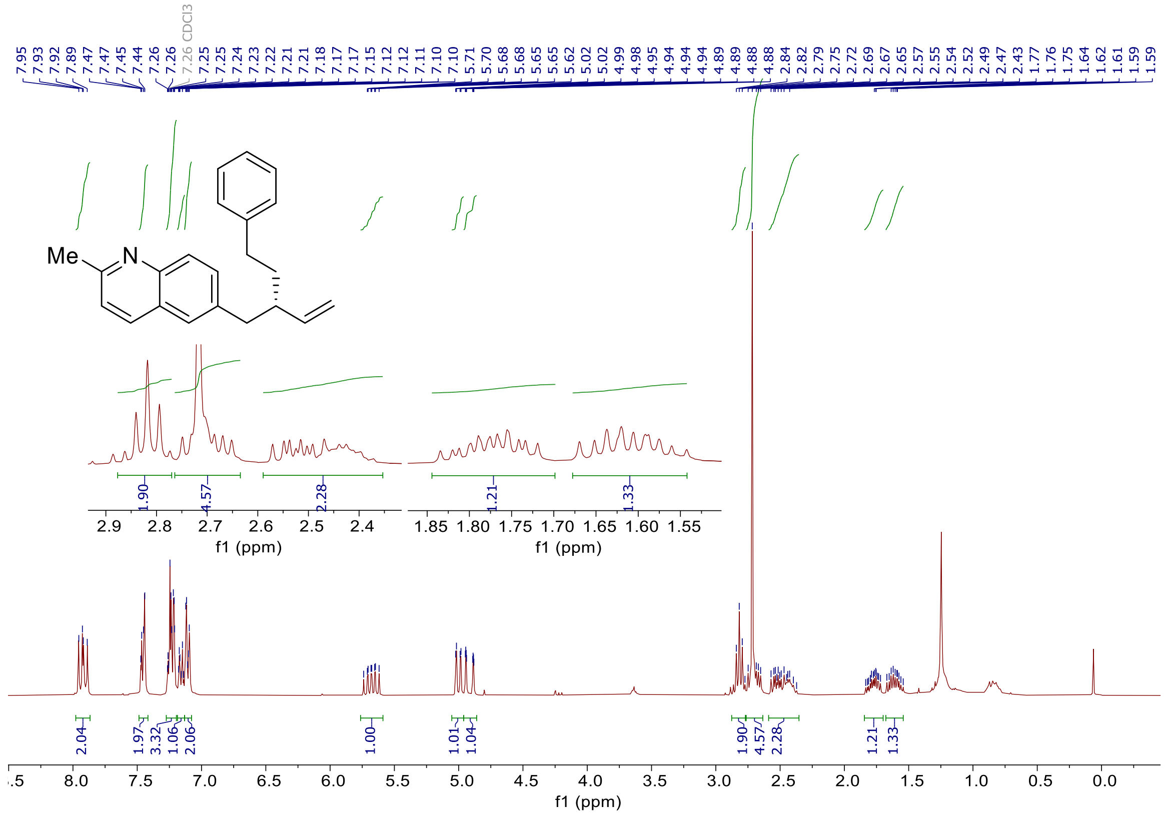

^{13}C NMR (76 MHz, $CDCl_3$) of compound **2m**

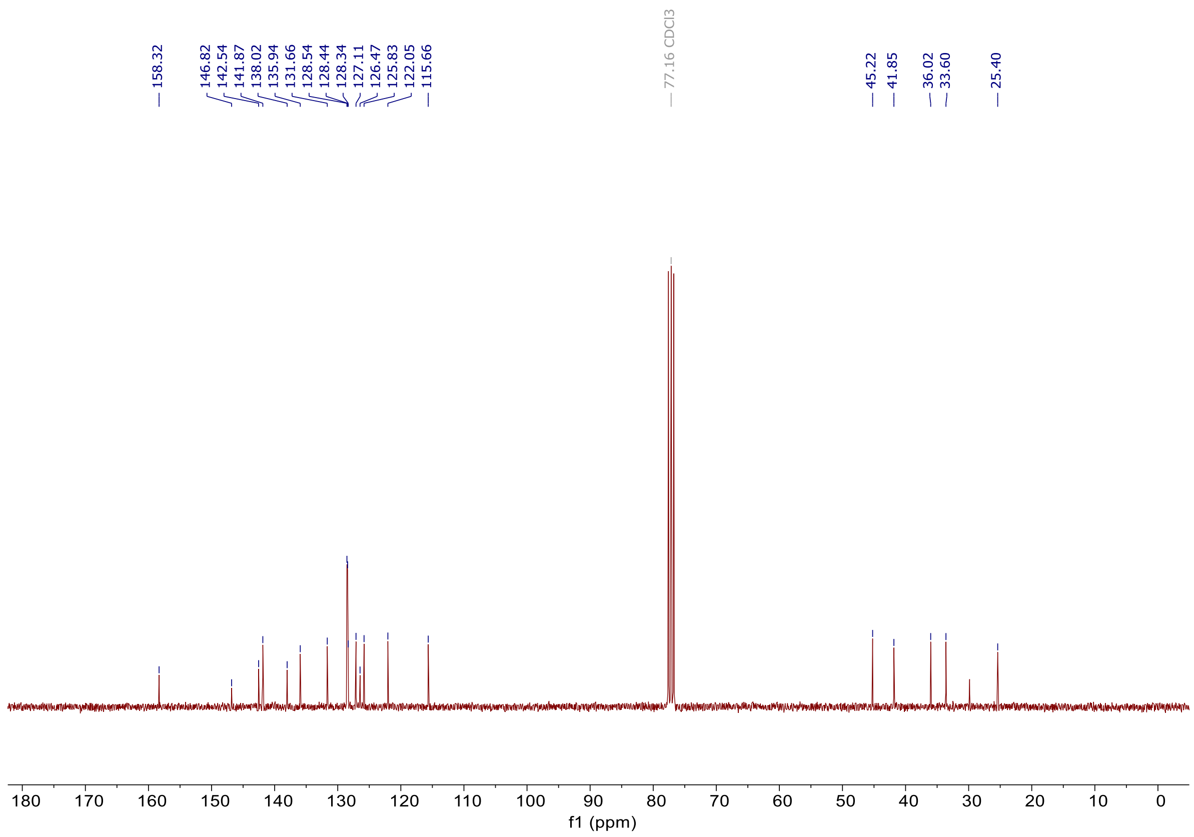

^{1}H NMR (300 MHz, $CDCl_3$) of compound **2n**

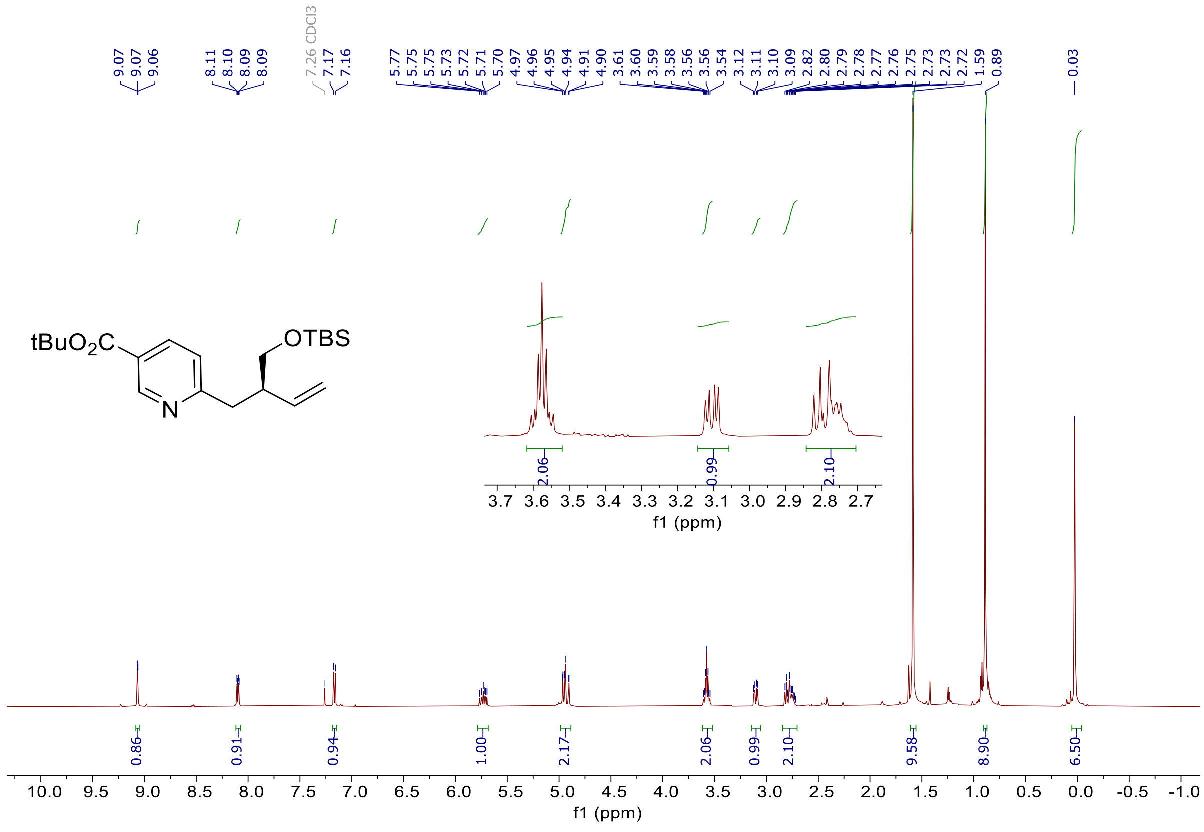

^{13}C NMR (76 MHz, $CDCl_3$) of compound **2n**

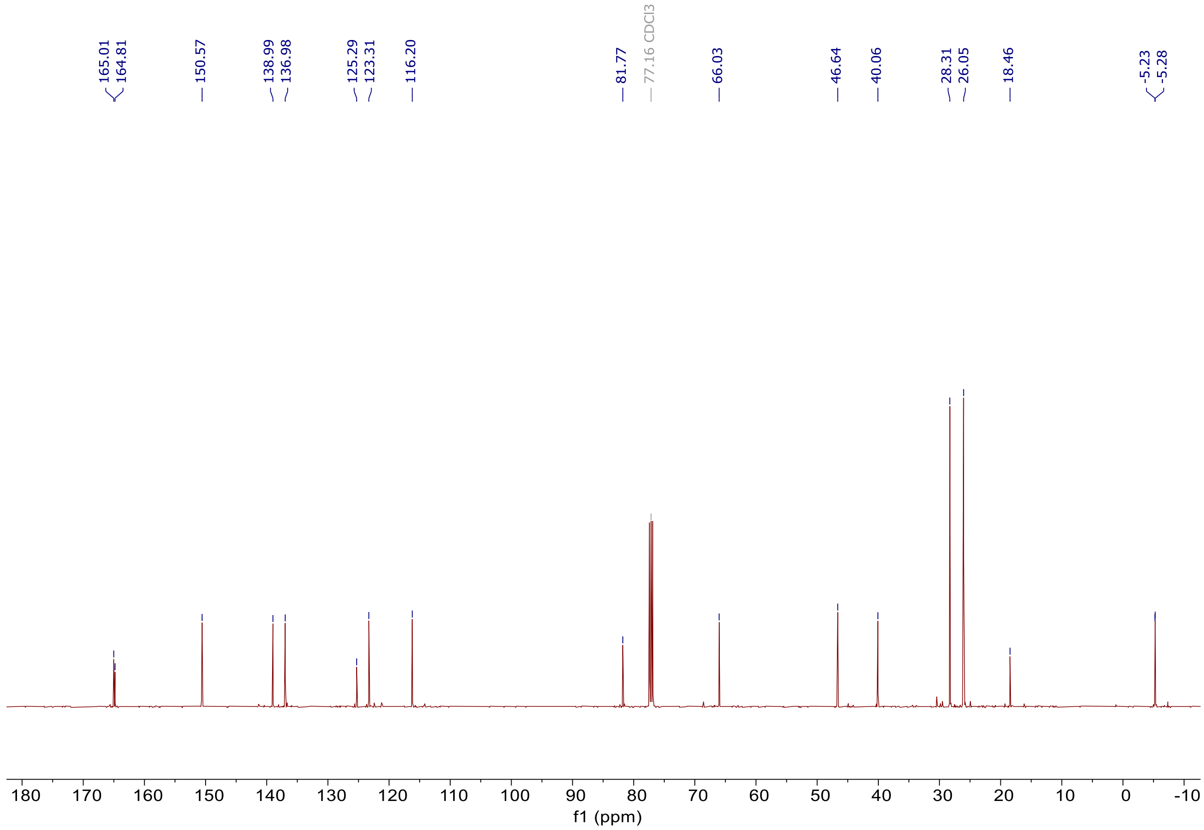

^{1}H NMR (500 MHz, $CDCl_3$) of compound **2o**

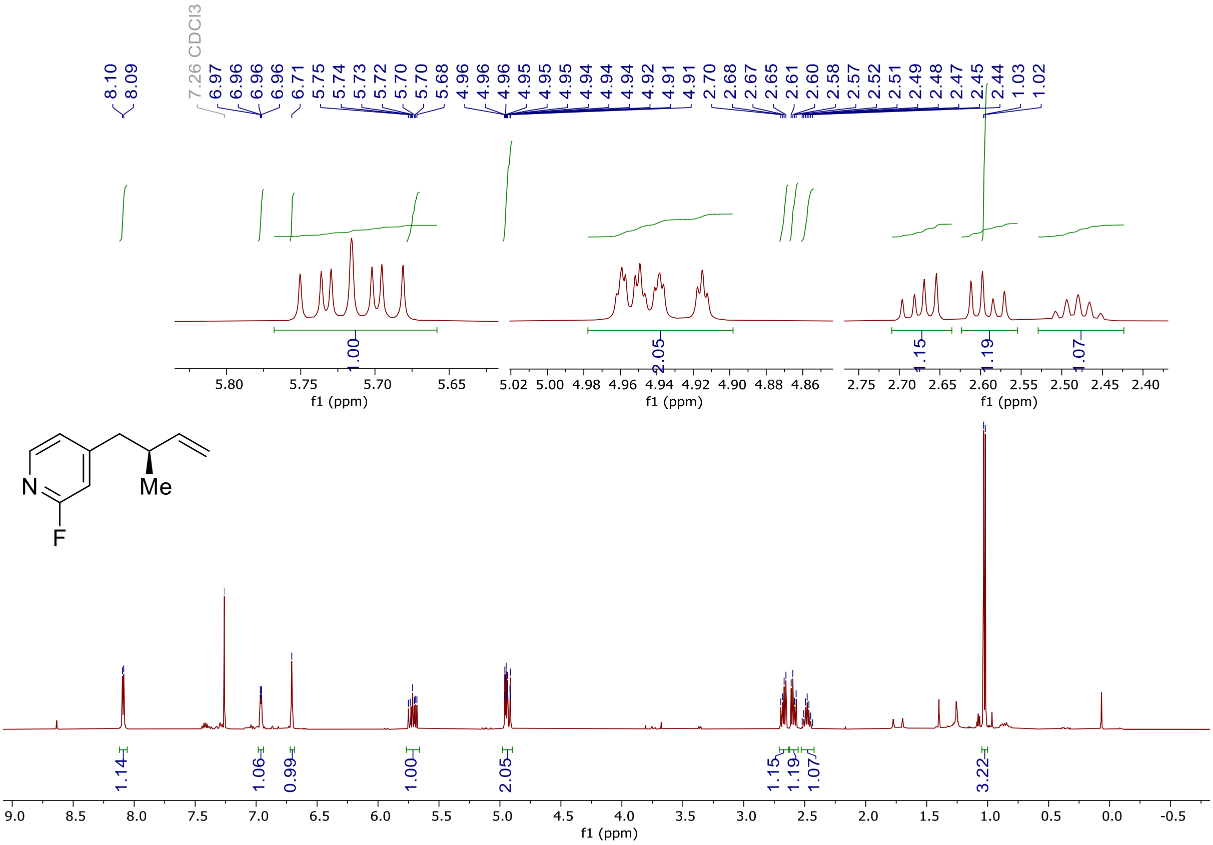

^{13}C NMR (126 MHz, $CDCl_3$) of compound **2o**

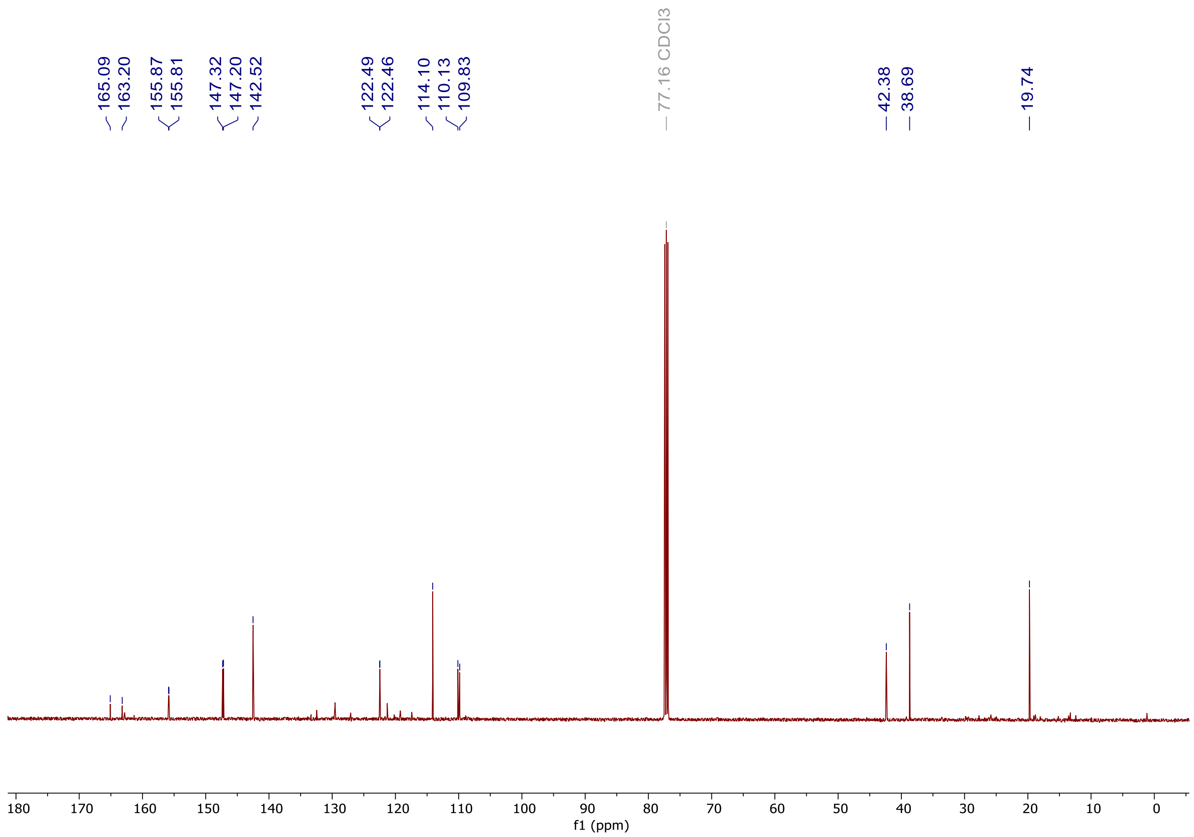

^{19}F NMR (471 MHz, $CDCl_3$) of compound **2o**

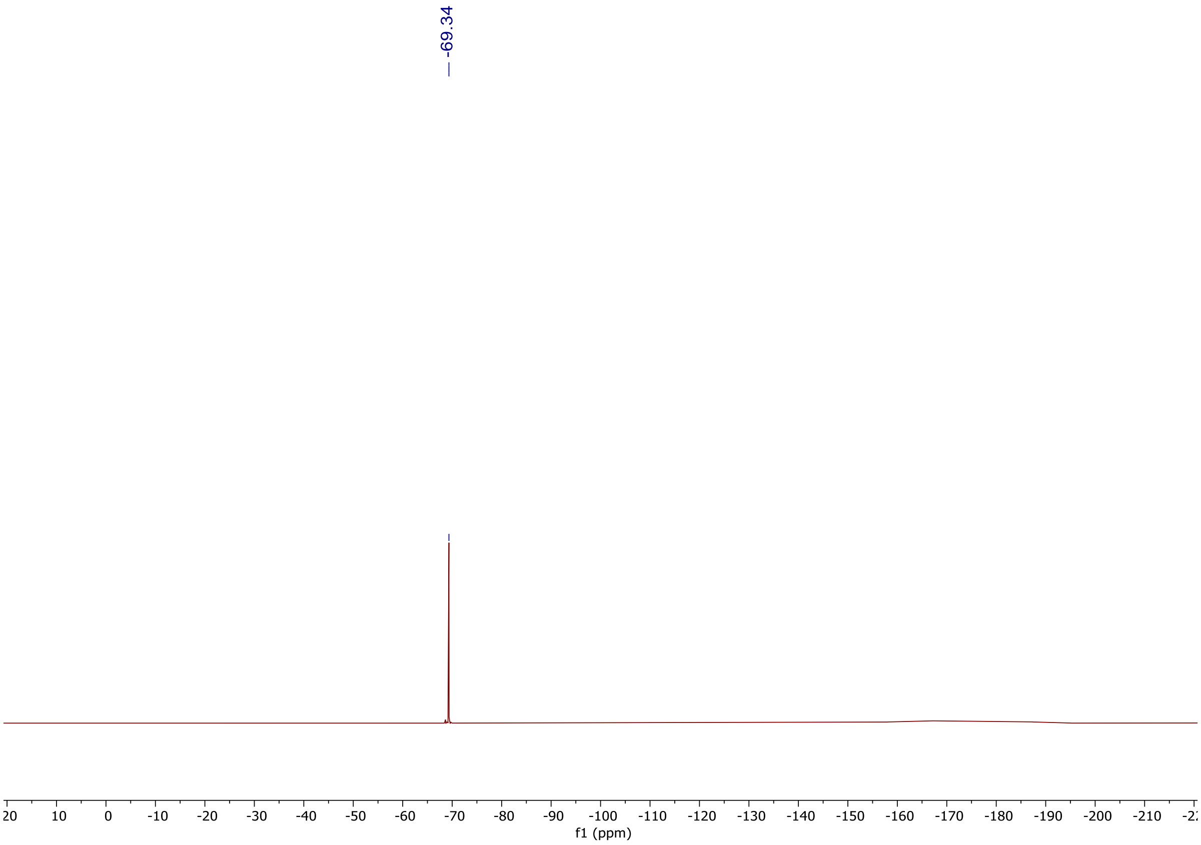

¹H NMR (300 MHz, $CDCl_3$) of compound **2p**

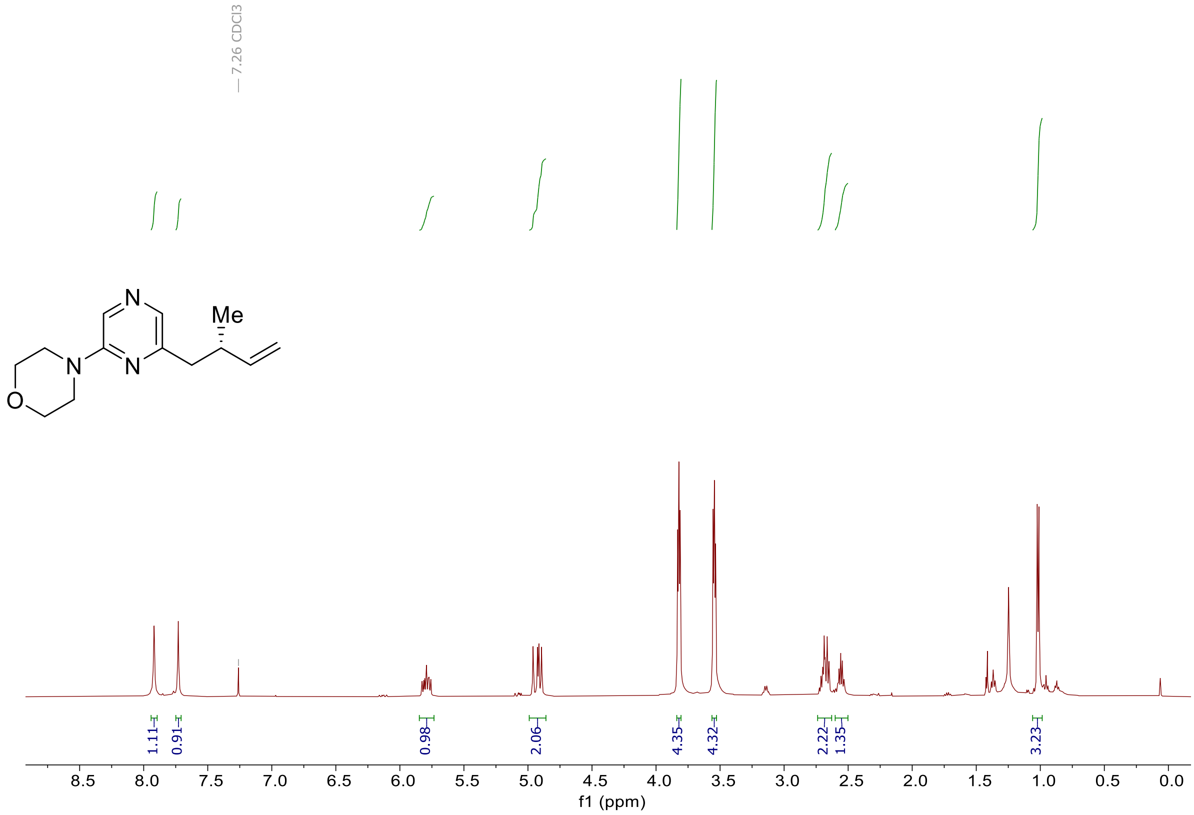

¹³C NMR (76 MHz, $CDCl_3$) of compound **2p**

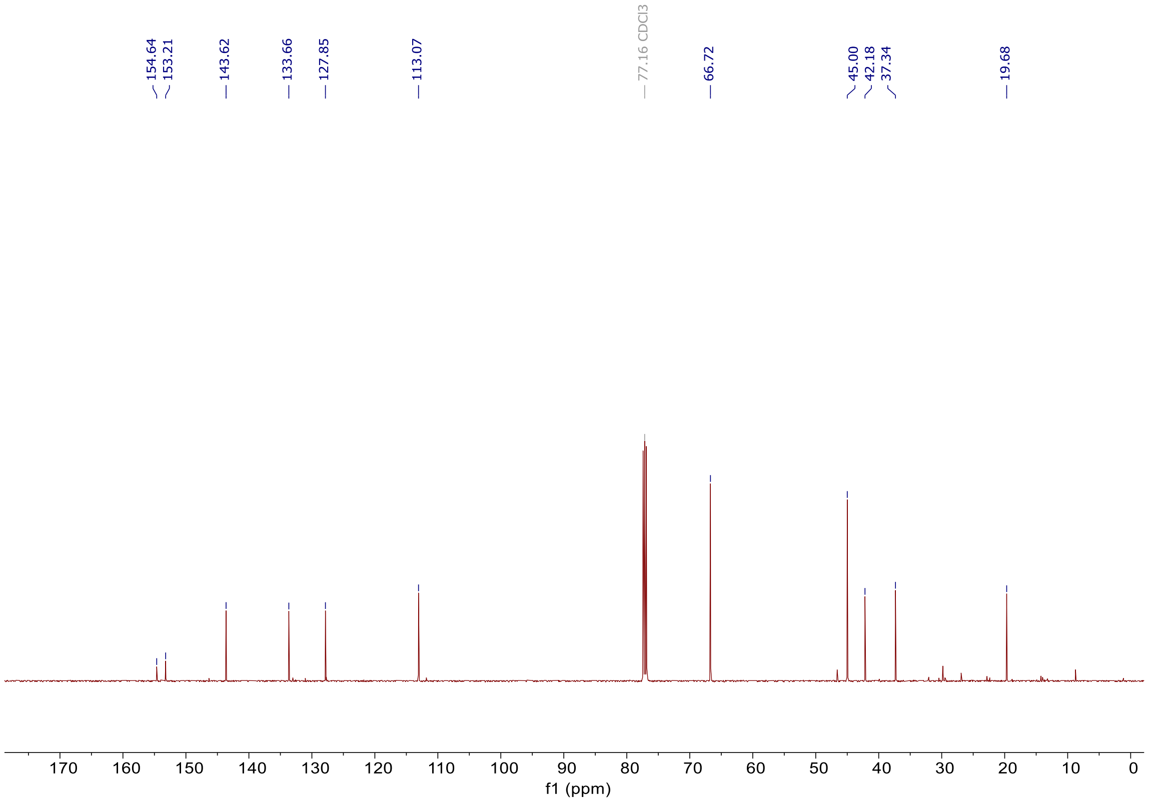

^{1}H NMR (300 MHz, $CDCl_3$) of compound **2q**

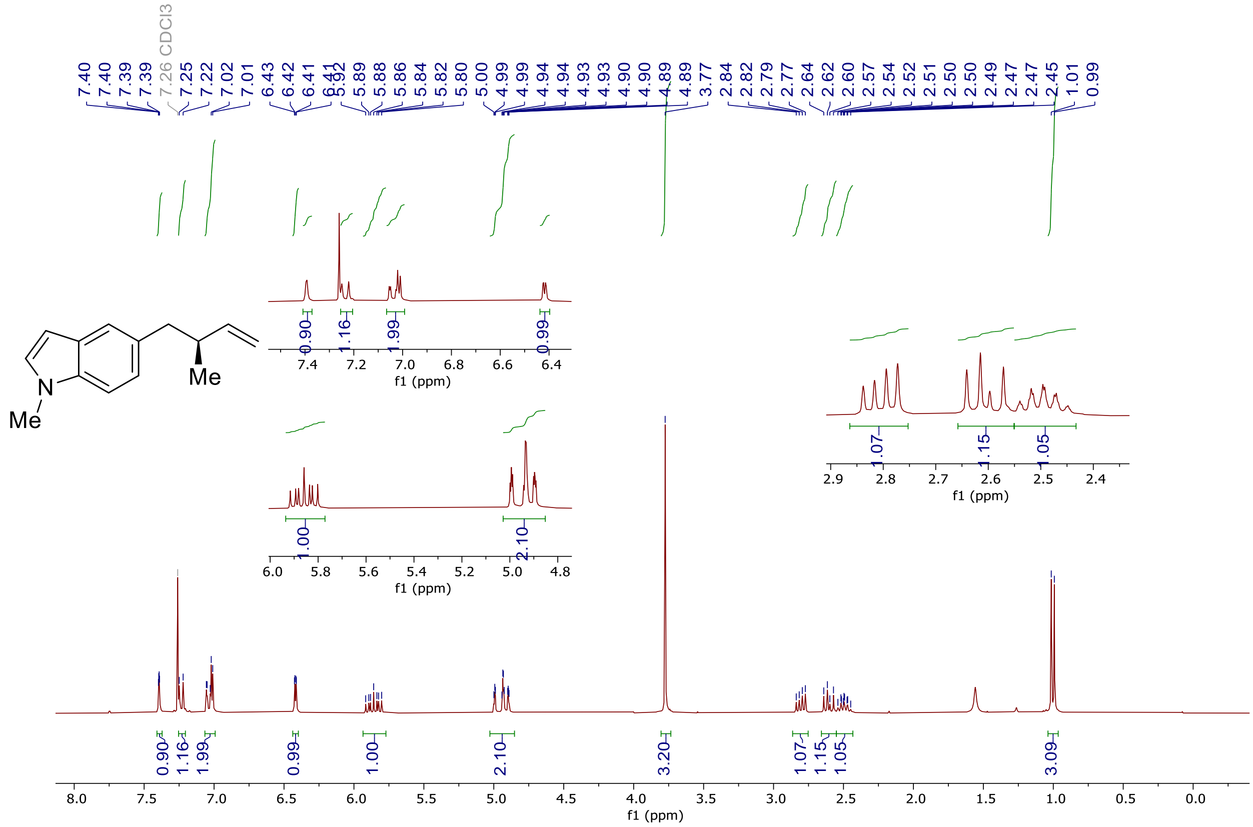

^{13}C NMR (75 MHz, $CDCl_3$) of compound **2q**

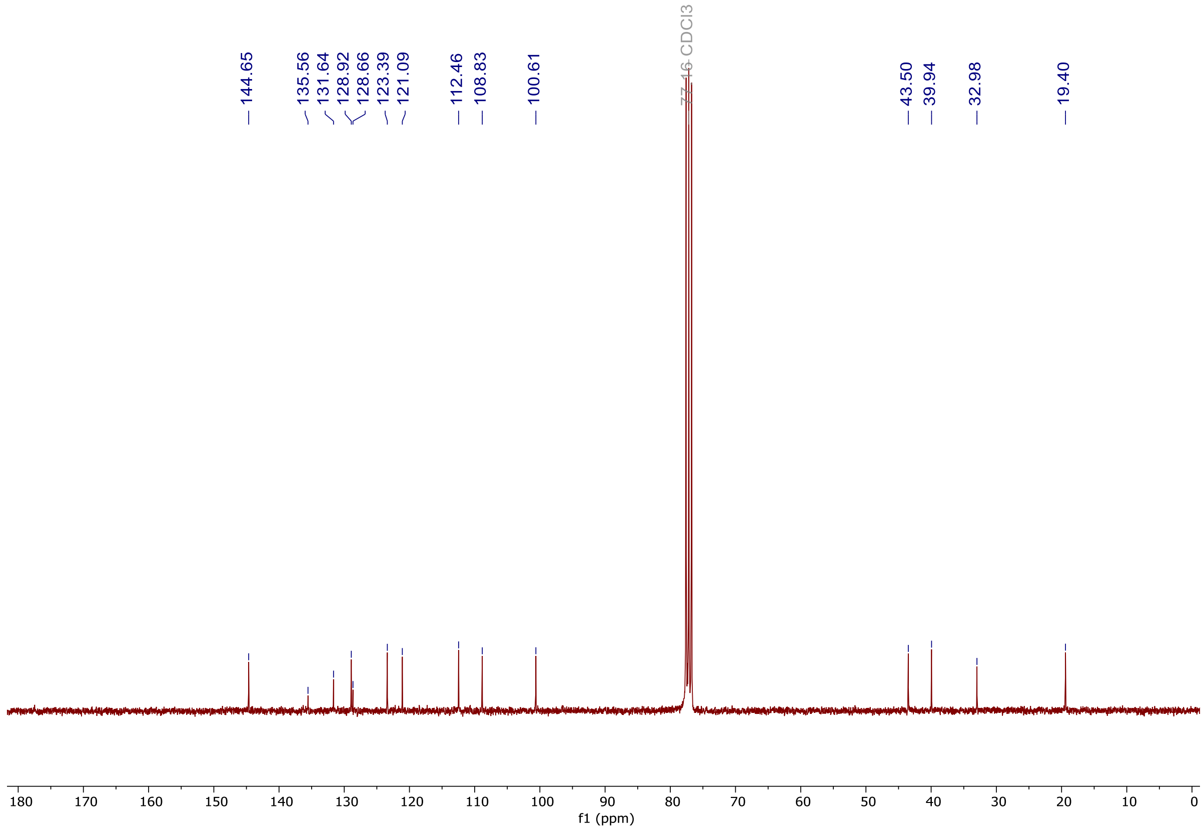

^{1}H NMR (300 MHz, $CDCl_3$) of compound **2r**

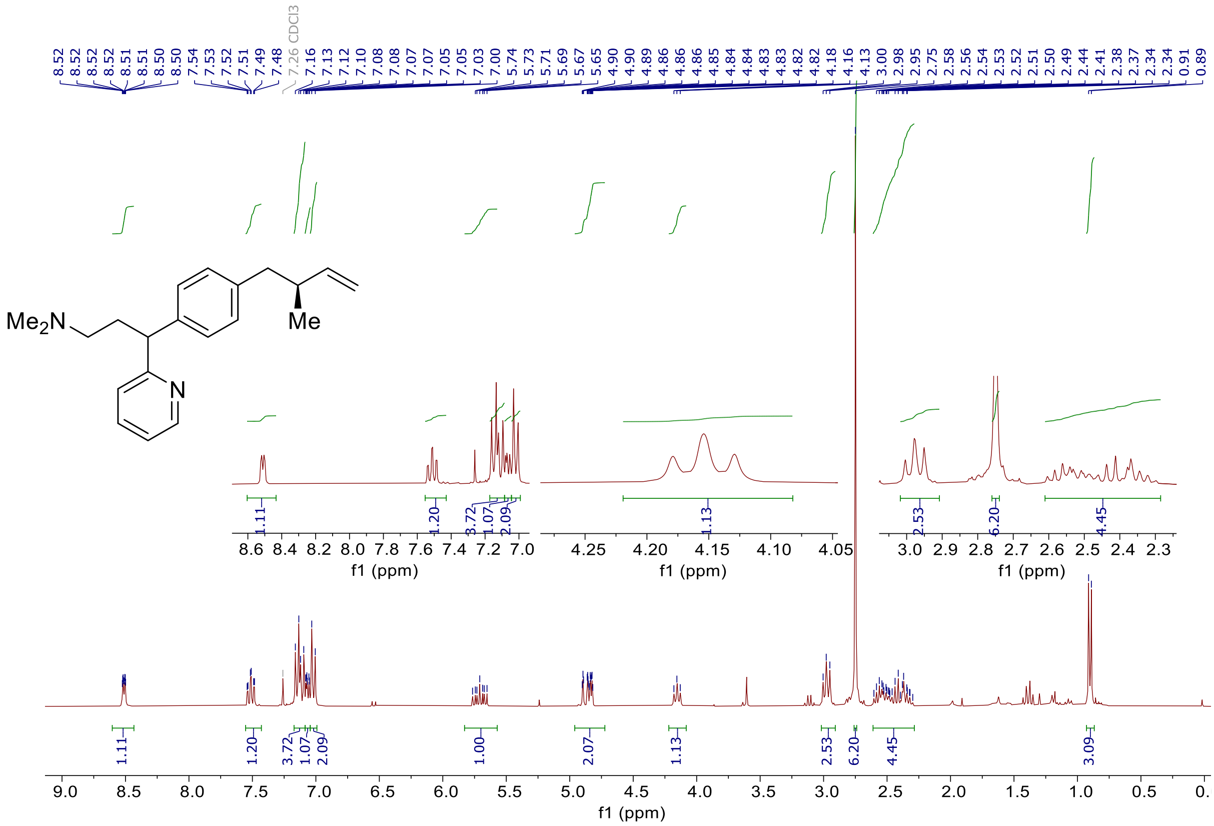

^{13}C NMR (76 MHz, $CDCl_3$) of compound **2r**

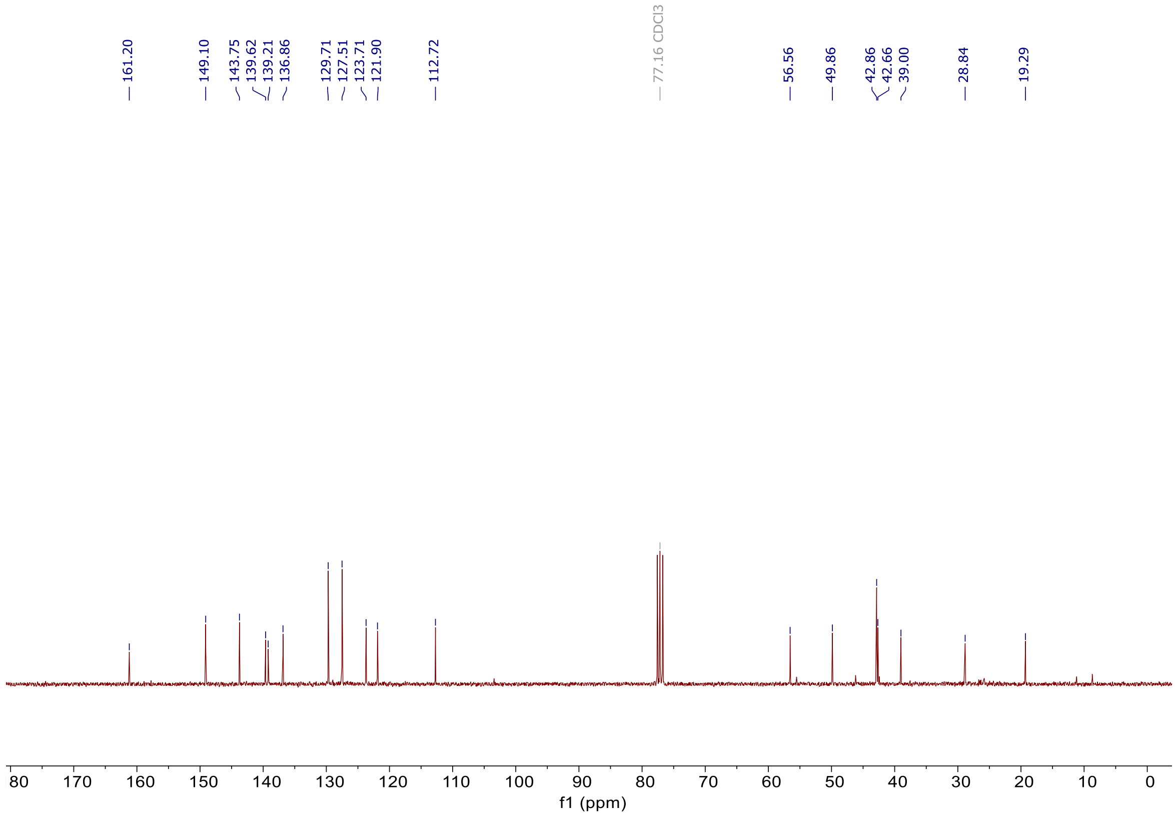

¹H NMR (300 MHz, DMSO^{d6}) of compound **2s**

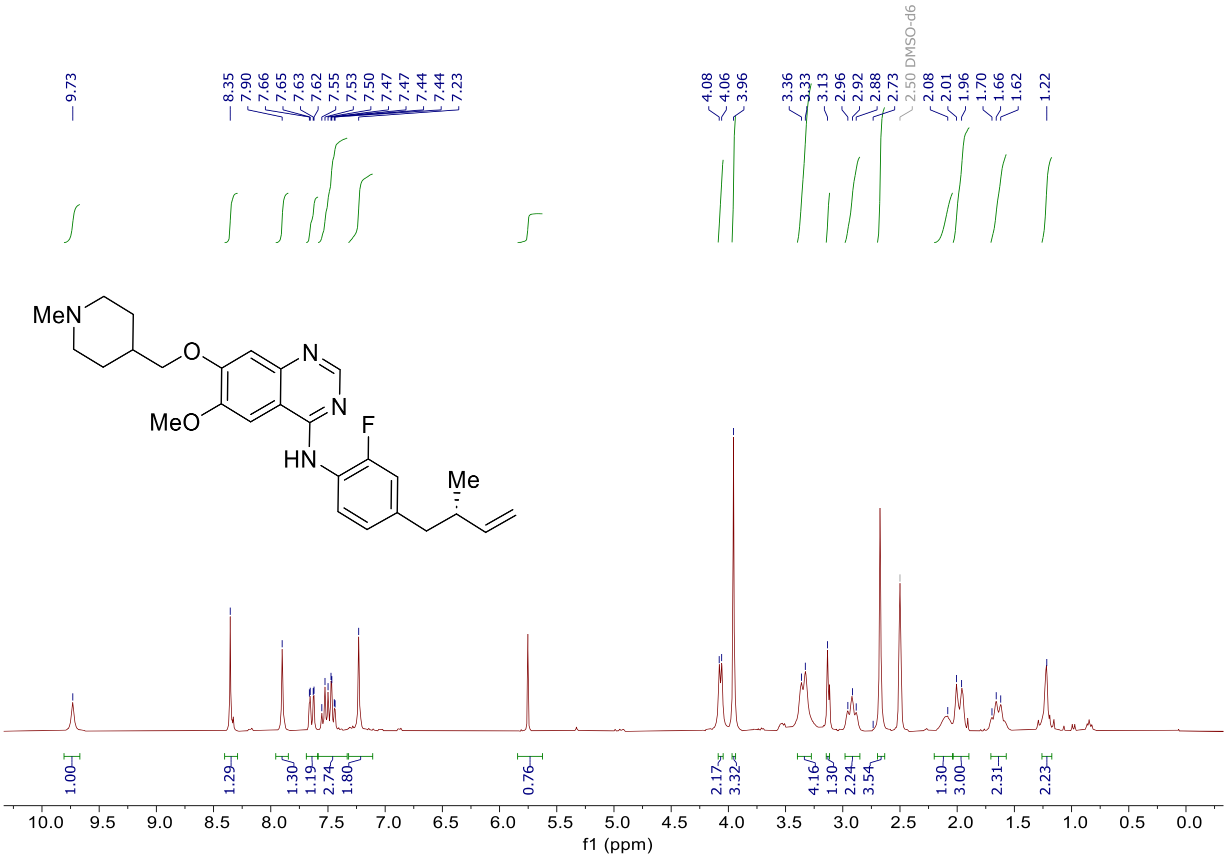

¹³C NMR (76 MHz, DMSO^{d6}) of compound **2s**

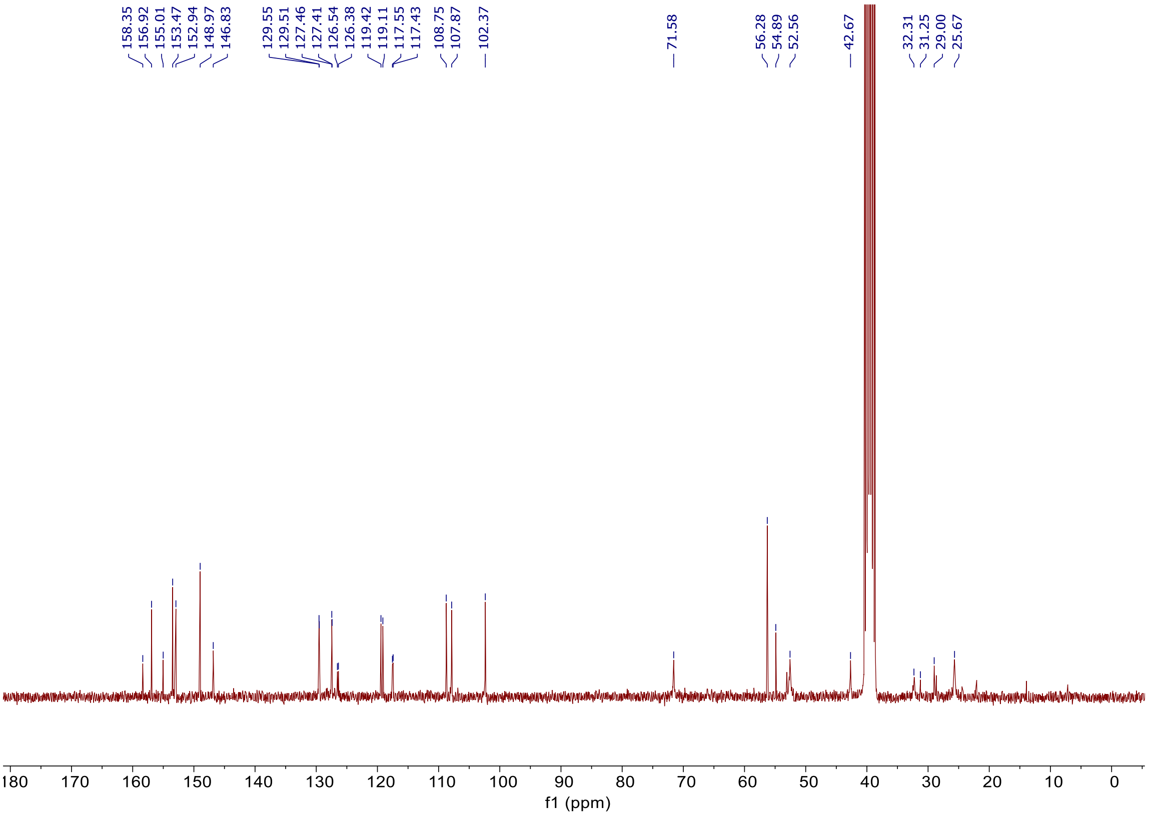

^{19}F NMR (282 MHz, MeOD) of compound **2s**

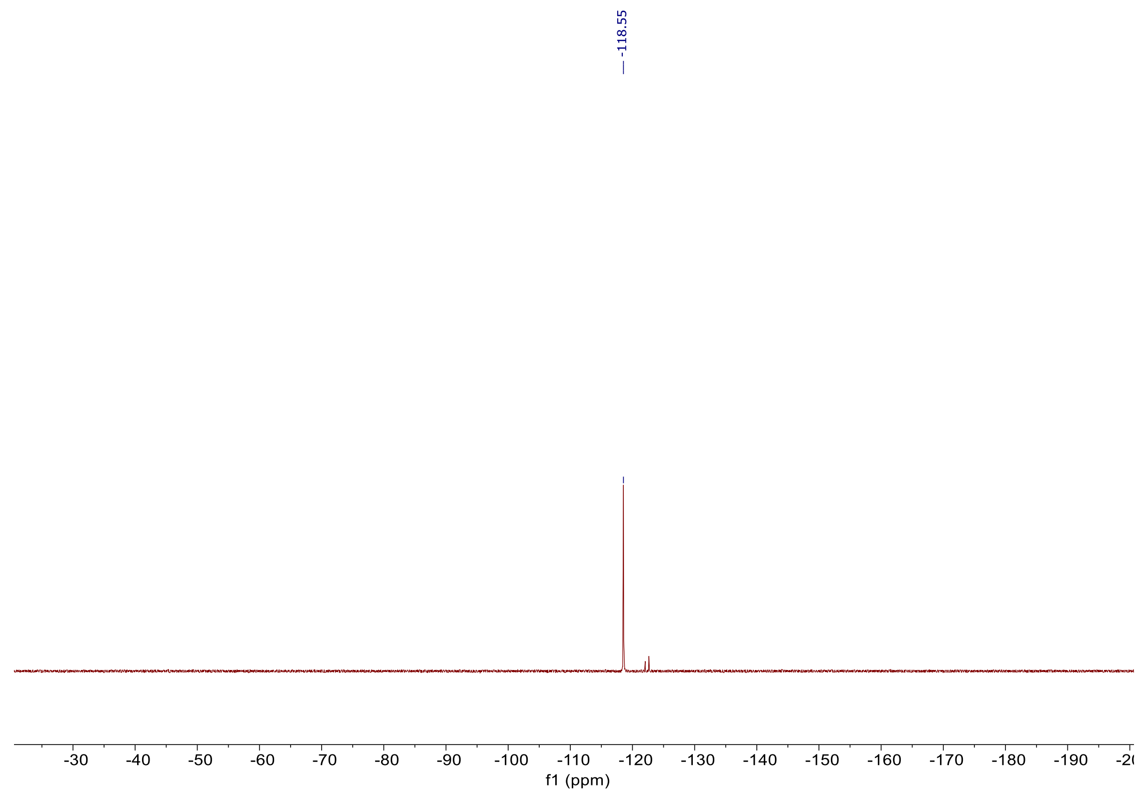

^{1}H NMR (300 MHz, $CDCl_3$) of compound **2t**

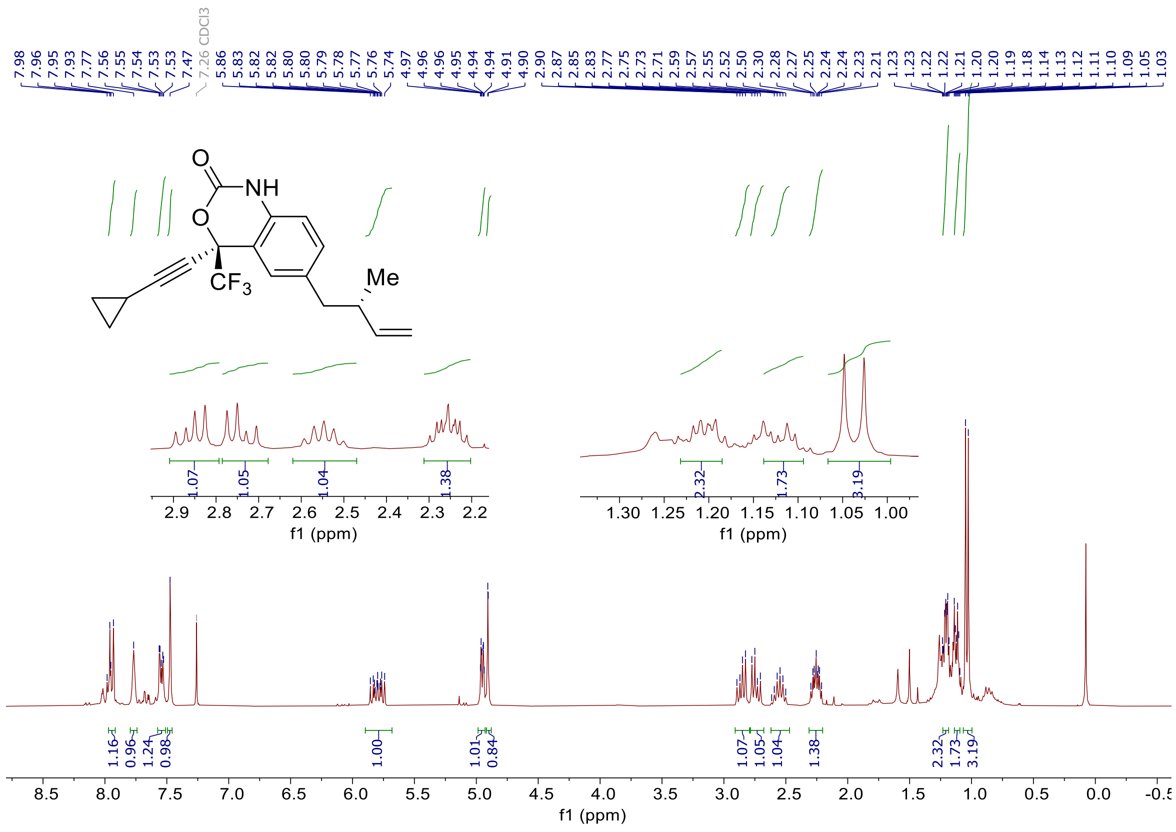

^{13}**C NMR** (75 MHz, $CDCl_3$) of compound **2t**

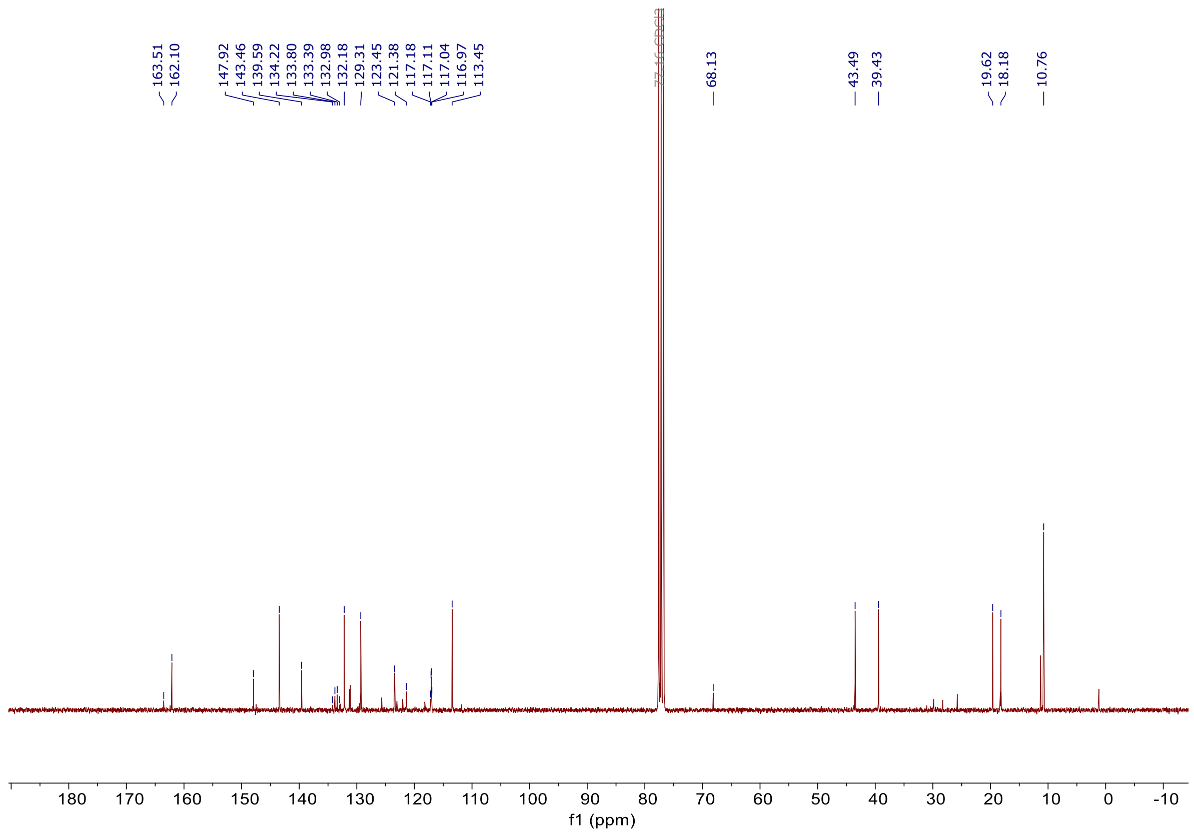

^{19}**F NMR** (282 MHz, $CDCl_3$) of compound **2t**

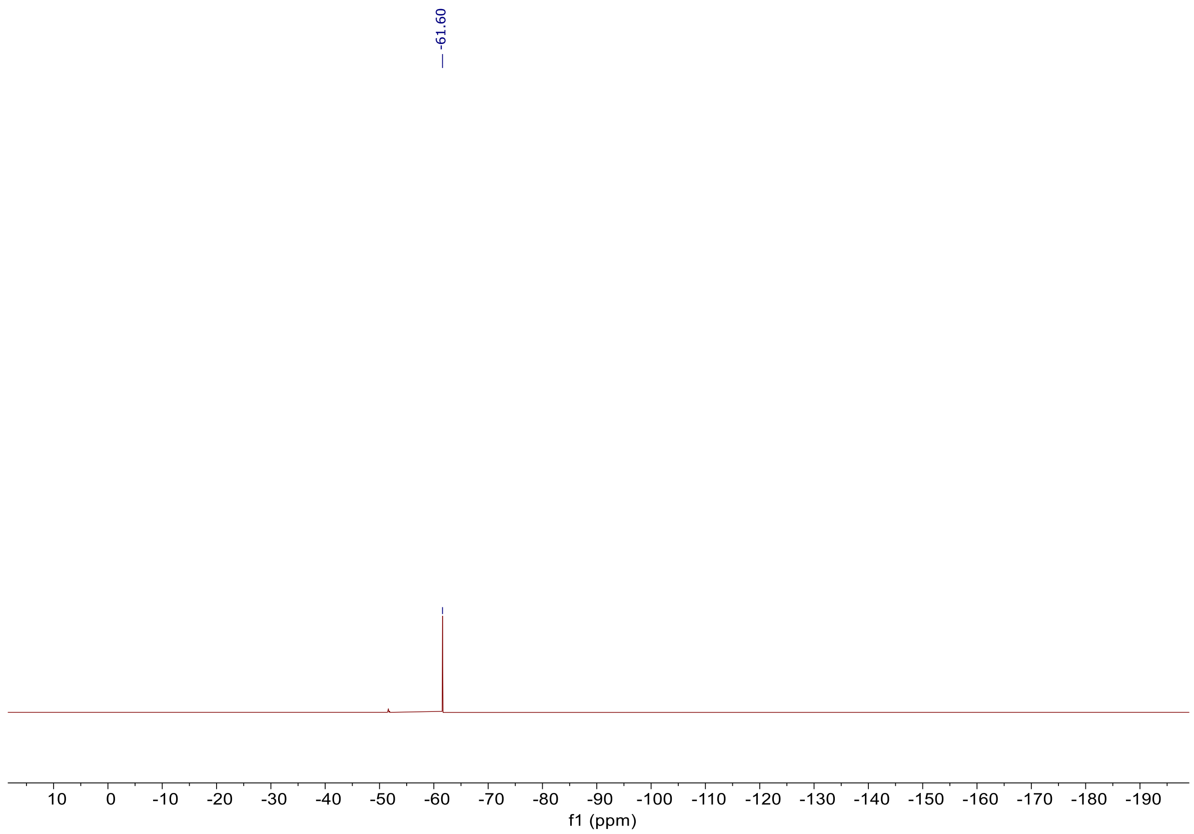

11.3. NMR Data: Negishi cross-coupling acyl halide's products

^{1}H NMR (300 MHz, $CDCl_3$) of compound **3e**

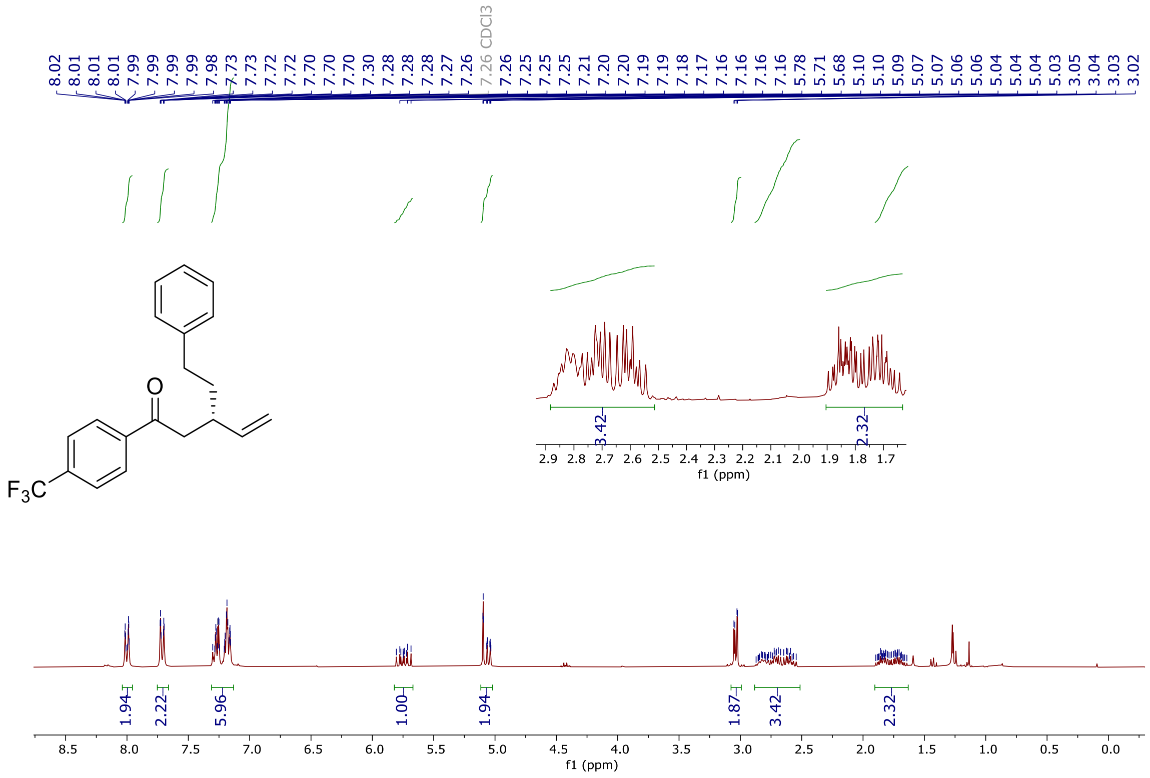

^{13}C NMR (76 MHz, $CDCl_3$) of compound **3e**

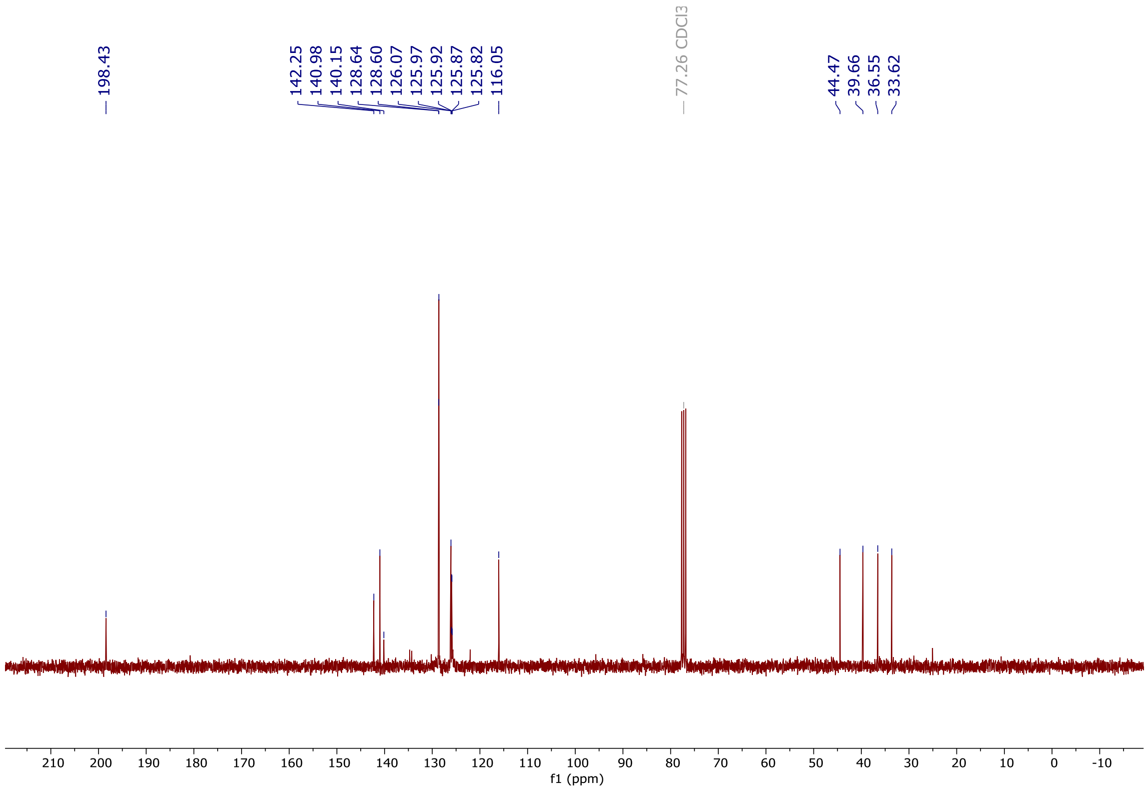

^{19}F NMR (470 MHz, $CDCl_3$) of compound **3e**

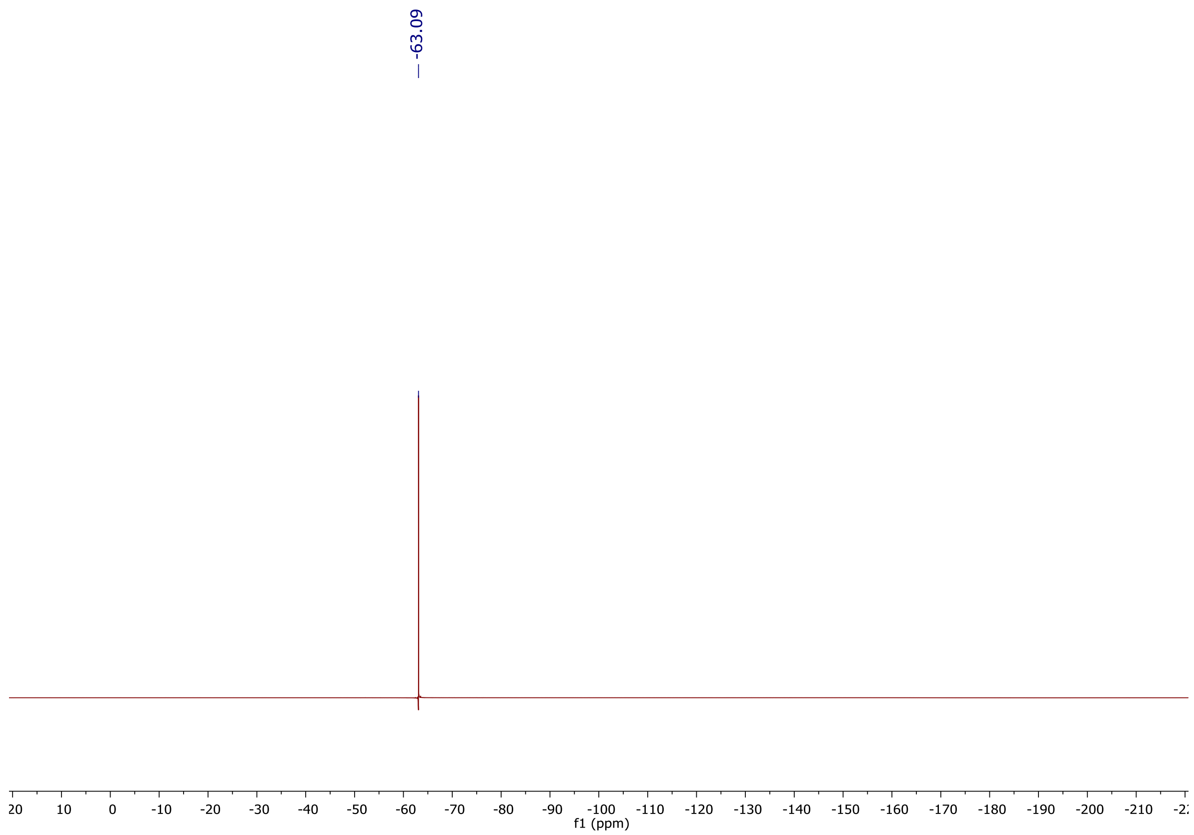

^{1}H NMR (300 MHz, $CDCl_3$) of compound **3f**

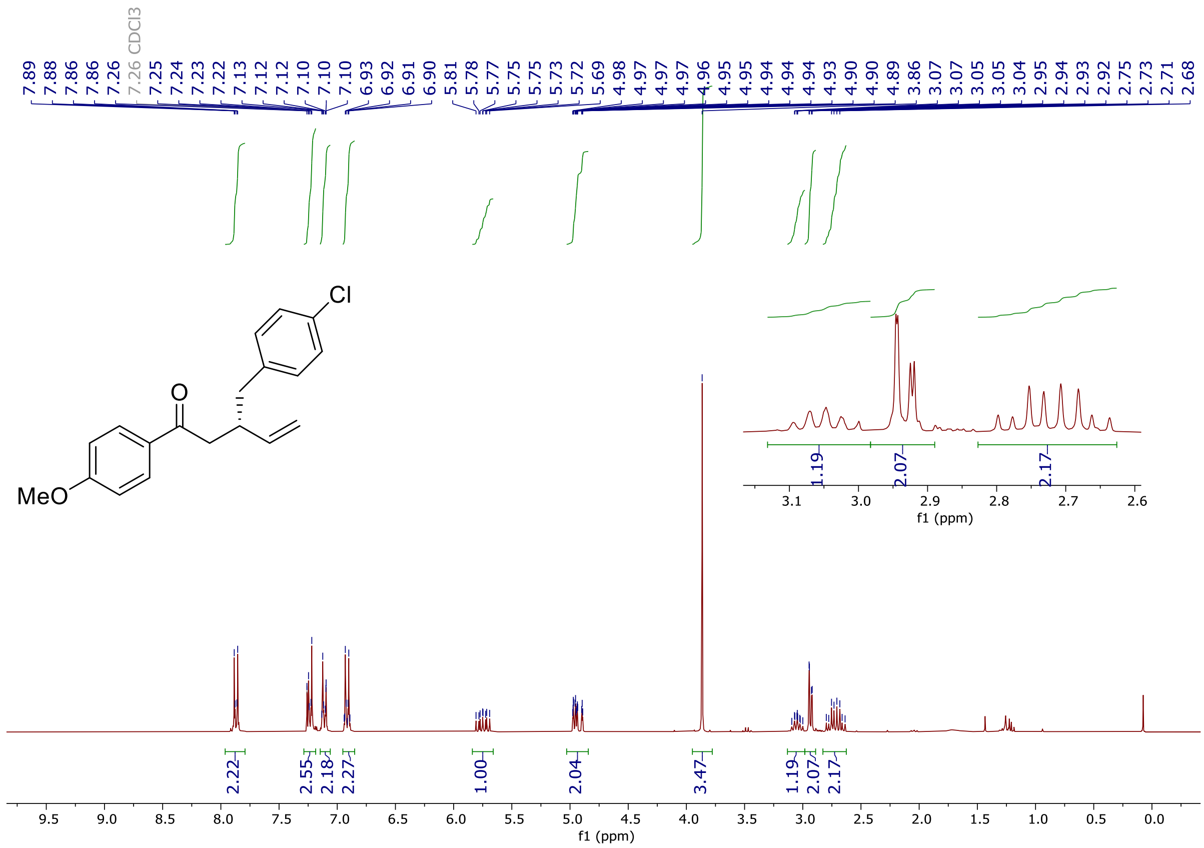

^{13}C NMR (126 MHz, $CDCl_3$) of compound **3f**

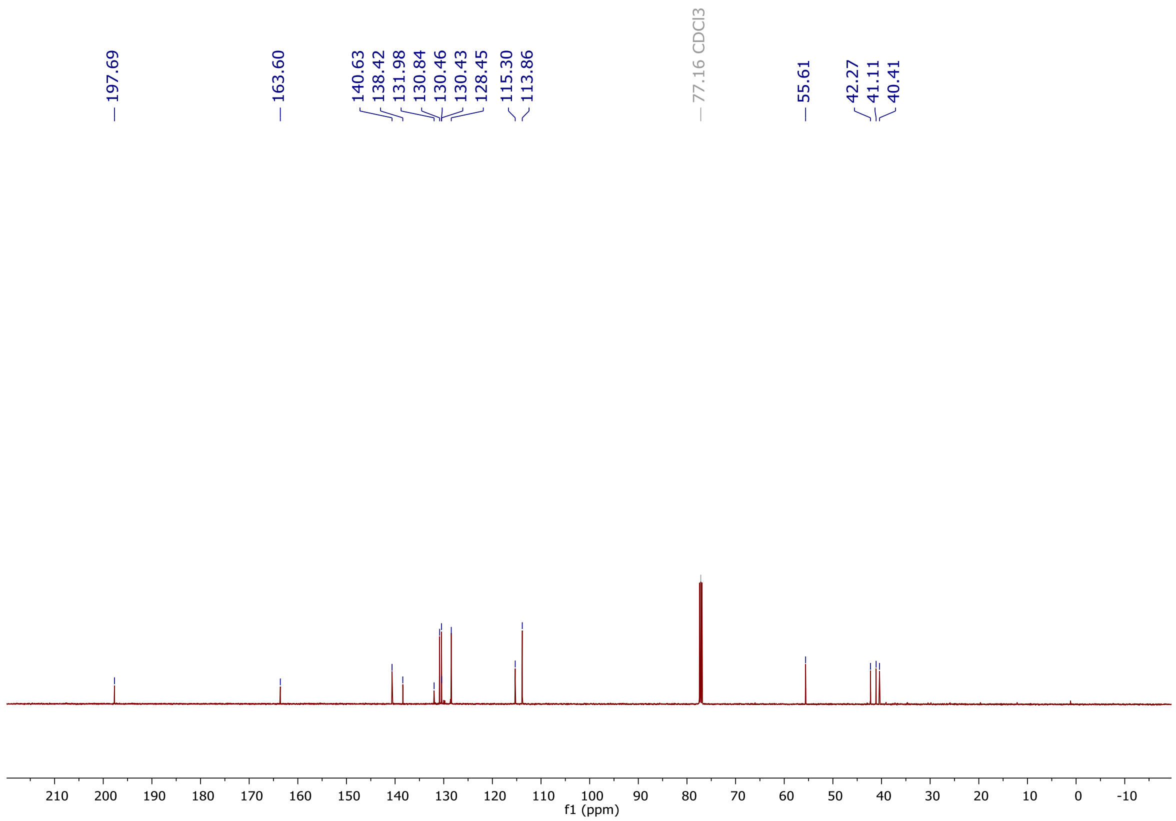

^{1}H NMR (300 MHz, $CDCl_3$) of compound **3g**

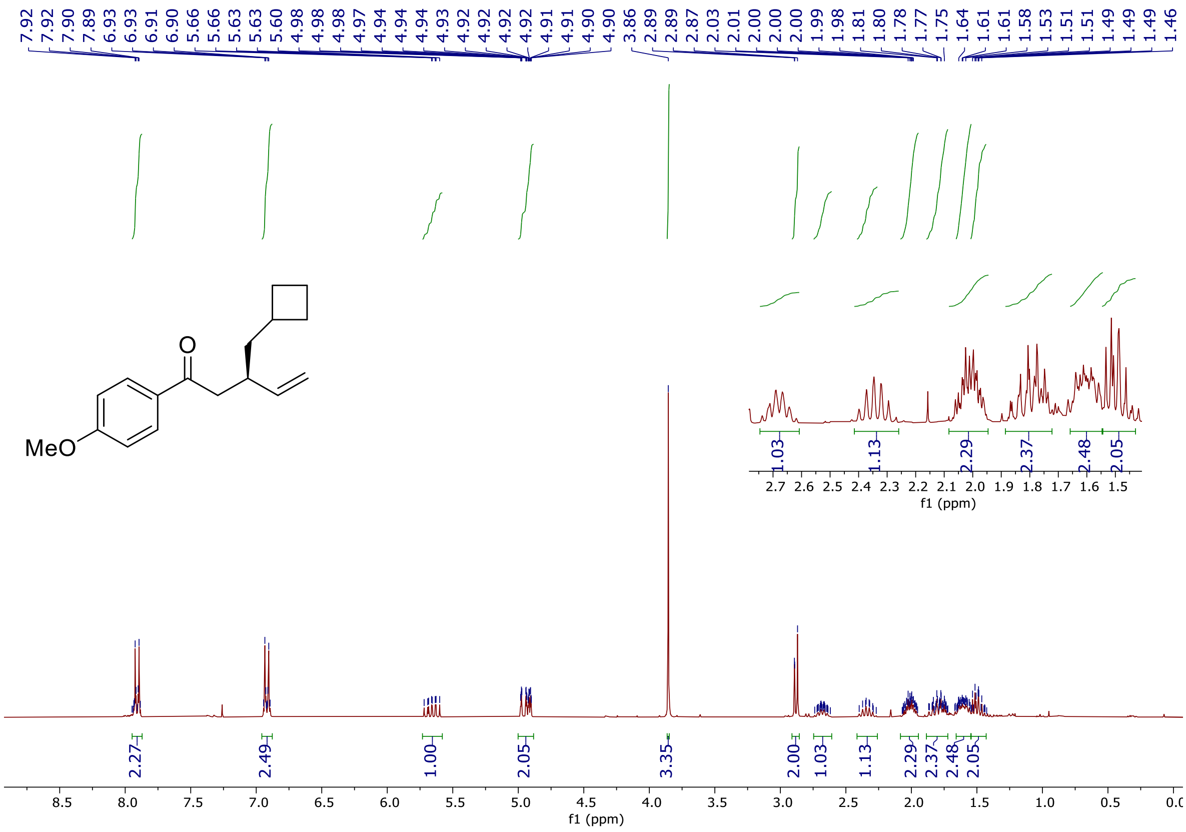

^{13}C NMR (126 MHz, $CDCl_3$) of compound **3g**

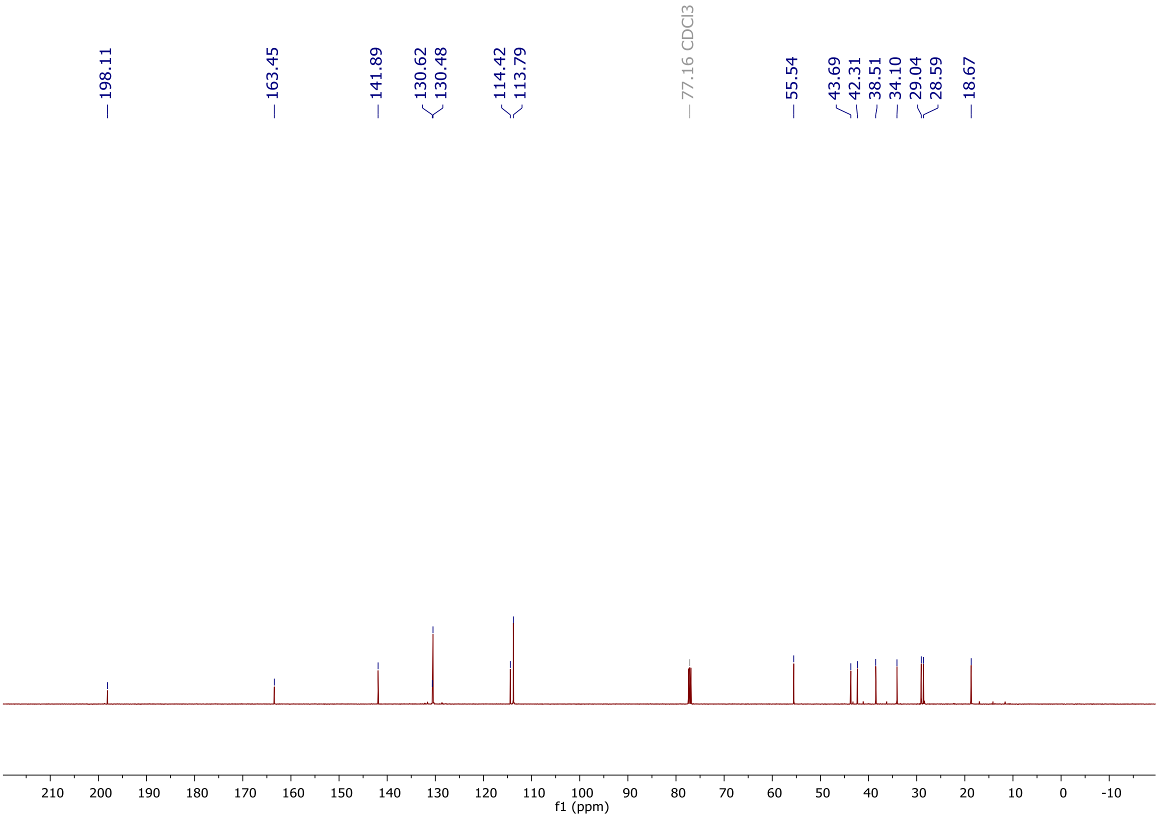

^{1}H NMR (300 MHz, $CDCl_3$) of compound **3h**

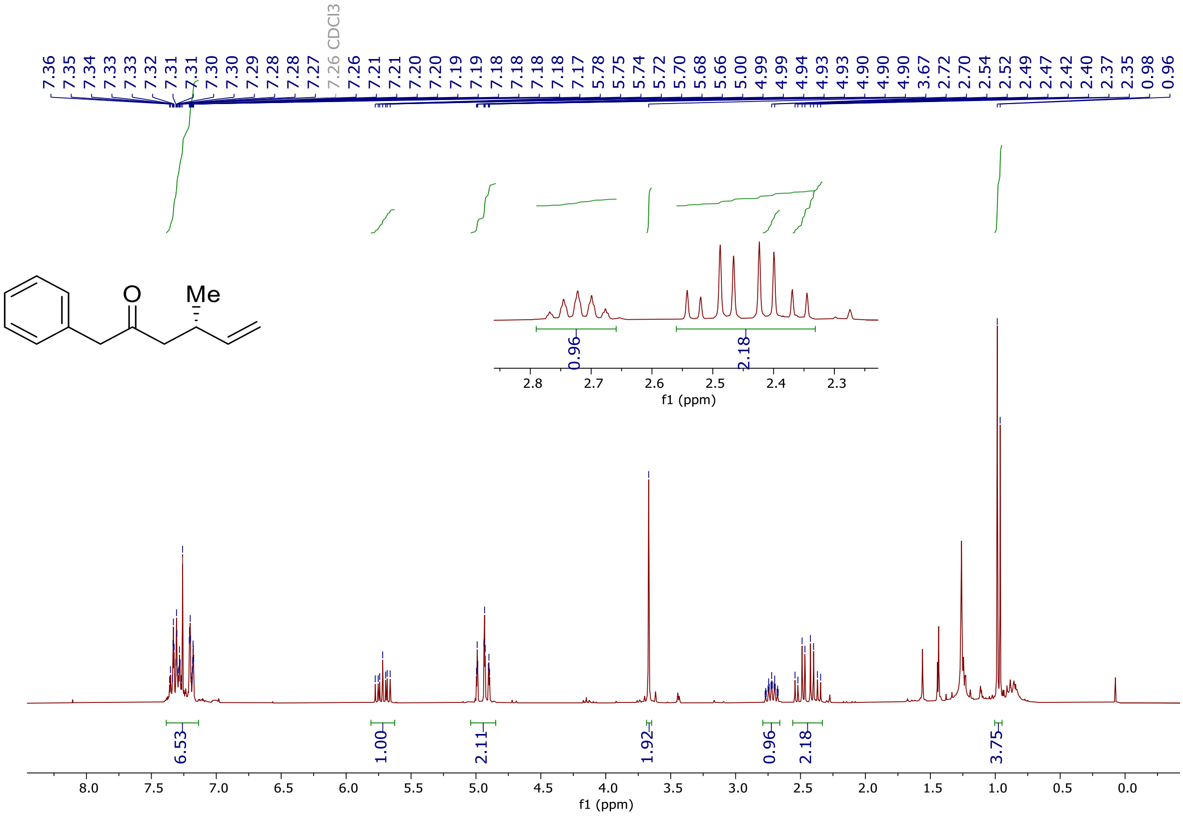

^{13}C NMR (76 MHz, $CDCl_3$) of compound **3h**

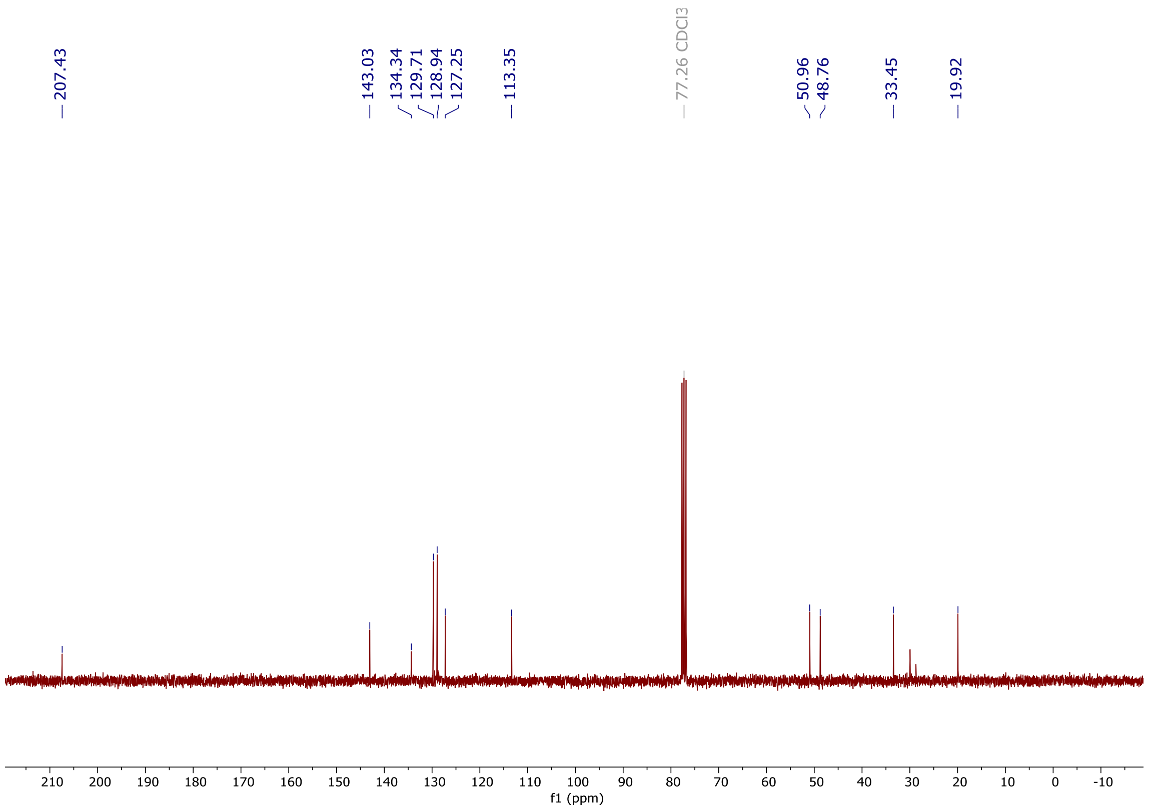

^{1}H NMR (300 MHz, $CDCl_3$) of compound **3i**

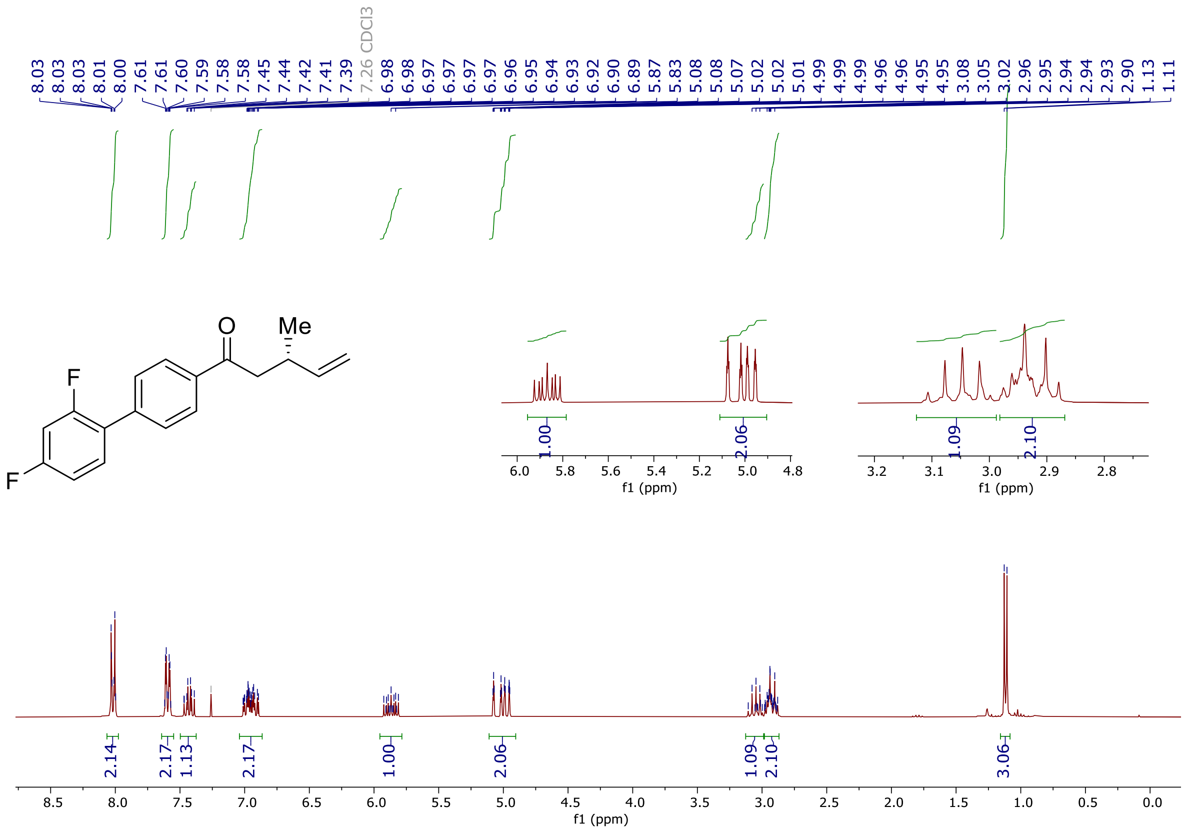

^{13}C NMR (126 MHz, $CDCl_3$) of compound **3i**

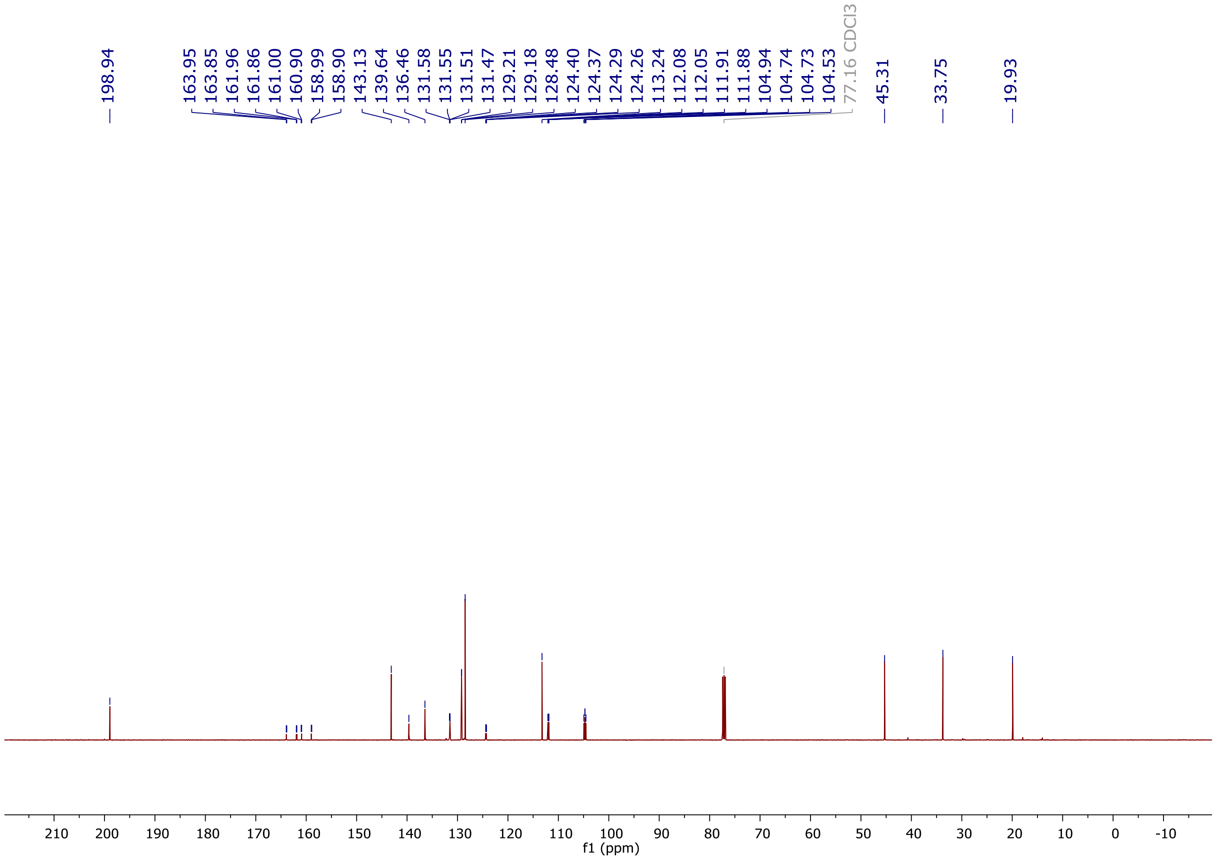

^{19}F NMR (282 MHz, $CDCl_3$) of compound **3i**

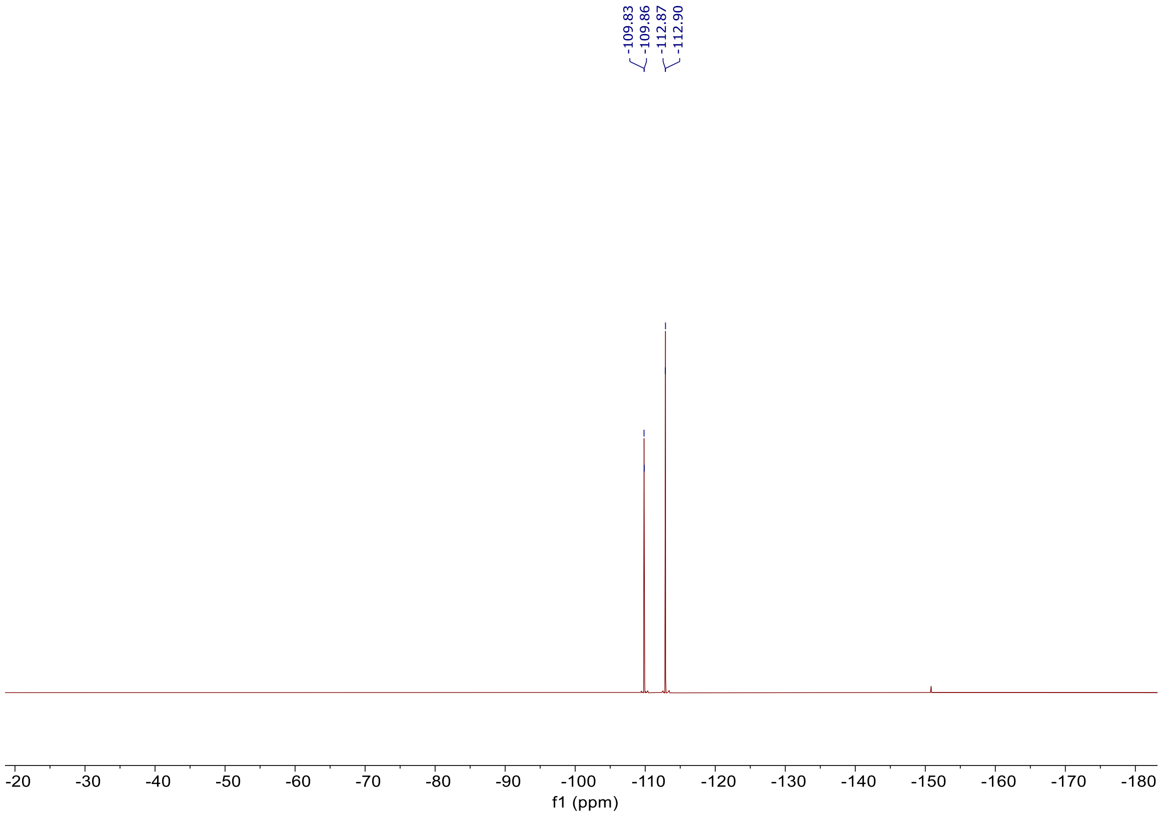

11.4. NMR Data: Negishi cross-coupling vinyl (pseudo)halide's products

^{1}H NMR (300 MHz, $CDCl_3$) of compound **4a**

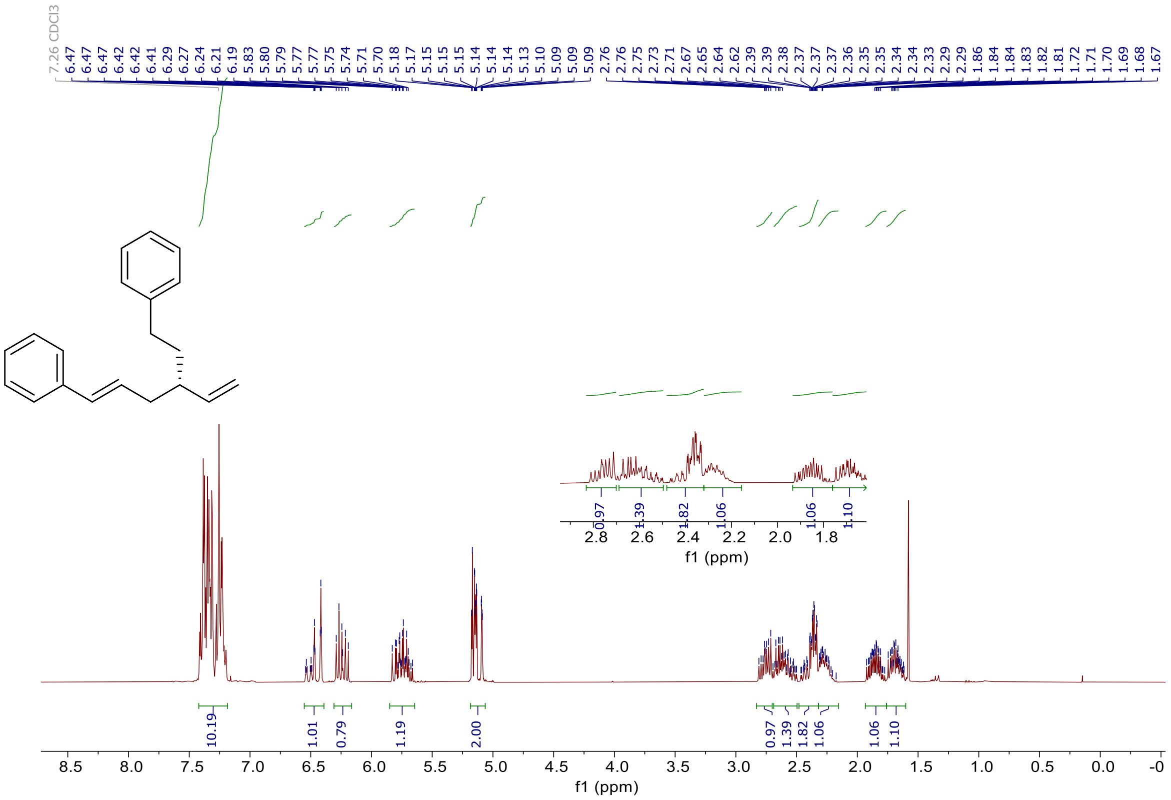

^{13}C NMR (76 MHz, $CDCl_3$) of compound **4a**

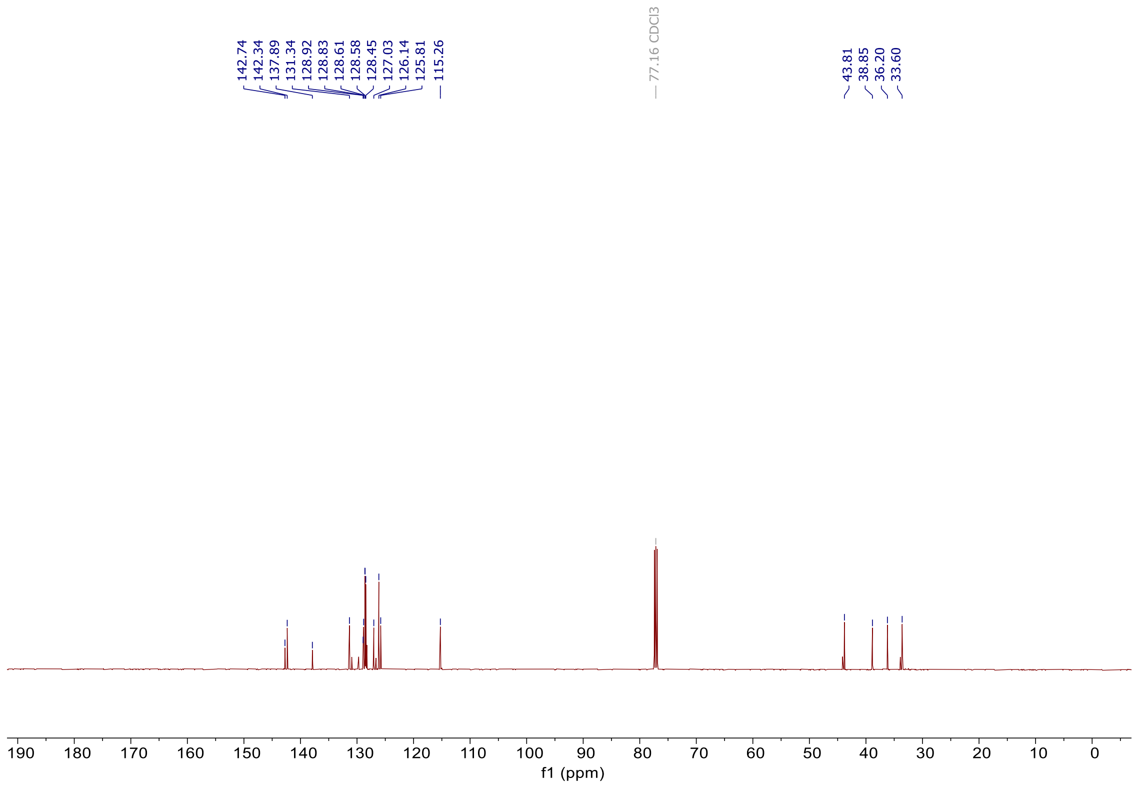

^{1}H NMR (300 MHz, $CDCl_3$) of compound **4b**

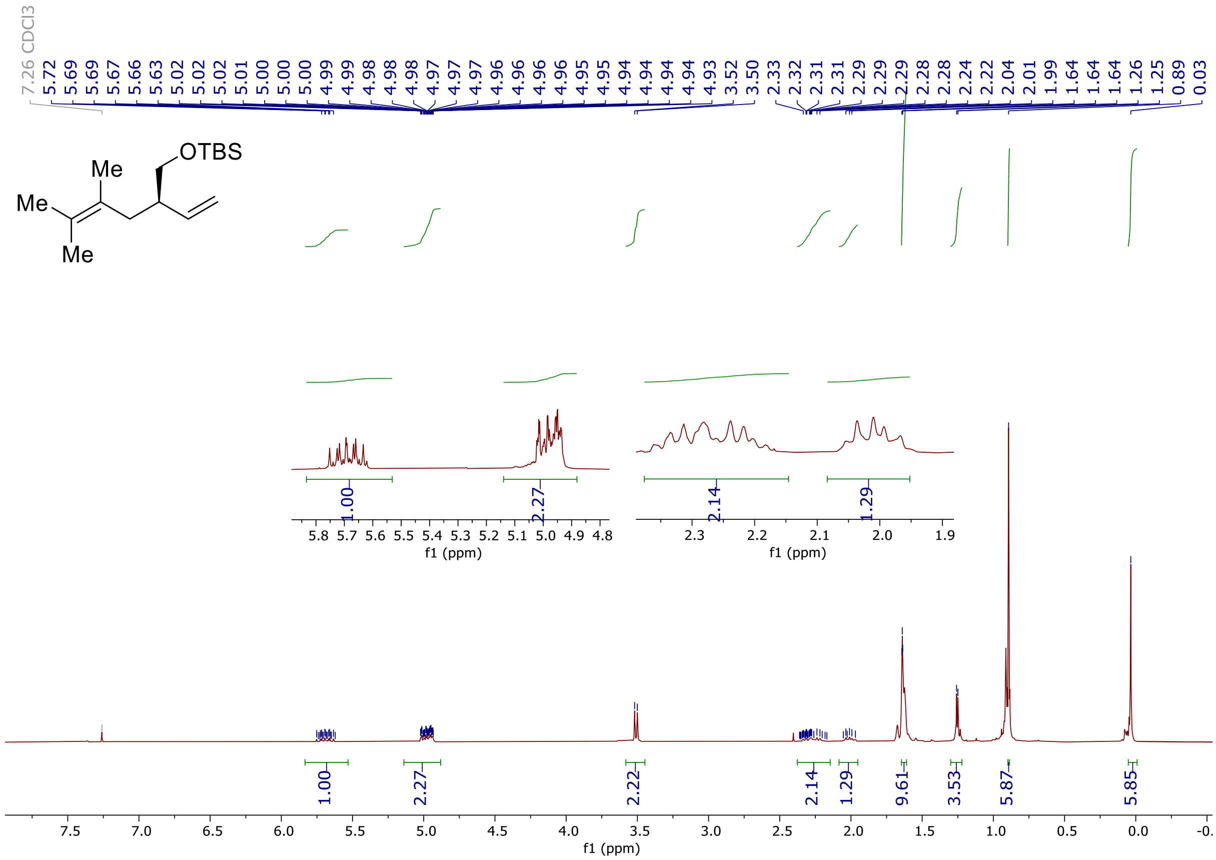

^{13}C NMR (126 MHz, $CDCl_3$) of compound **4b**

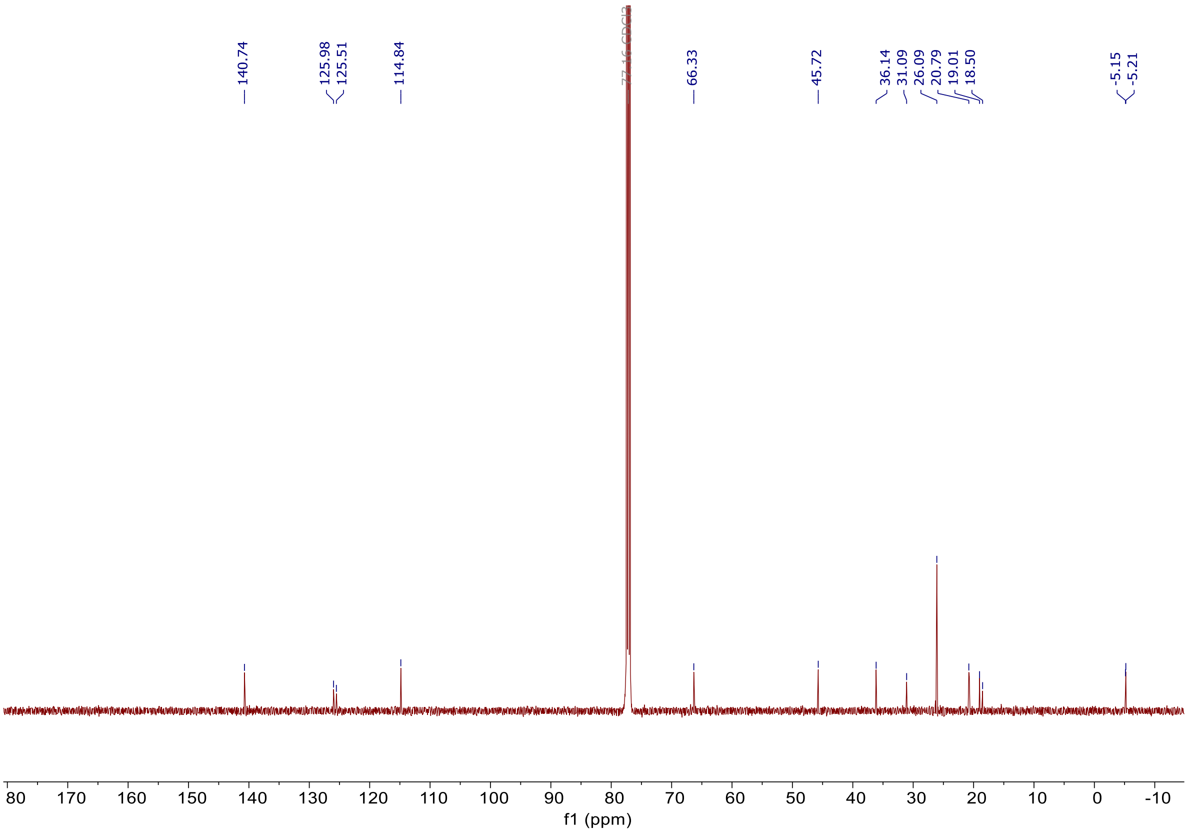

^{1}H NMR (300 MHz, $CDCl_3$) of compound **4c**

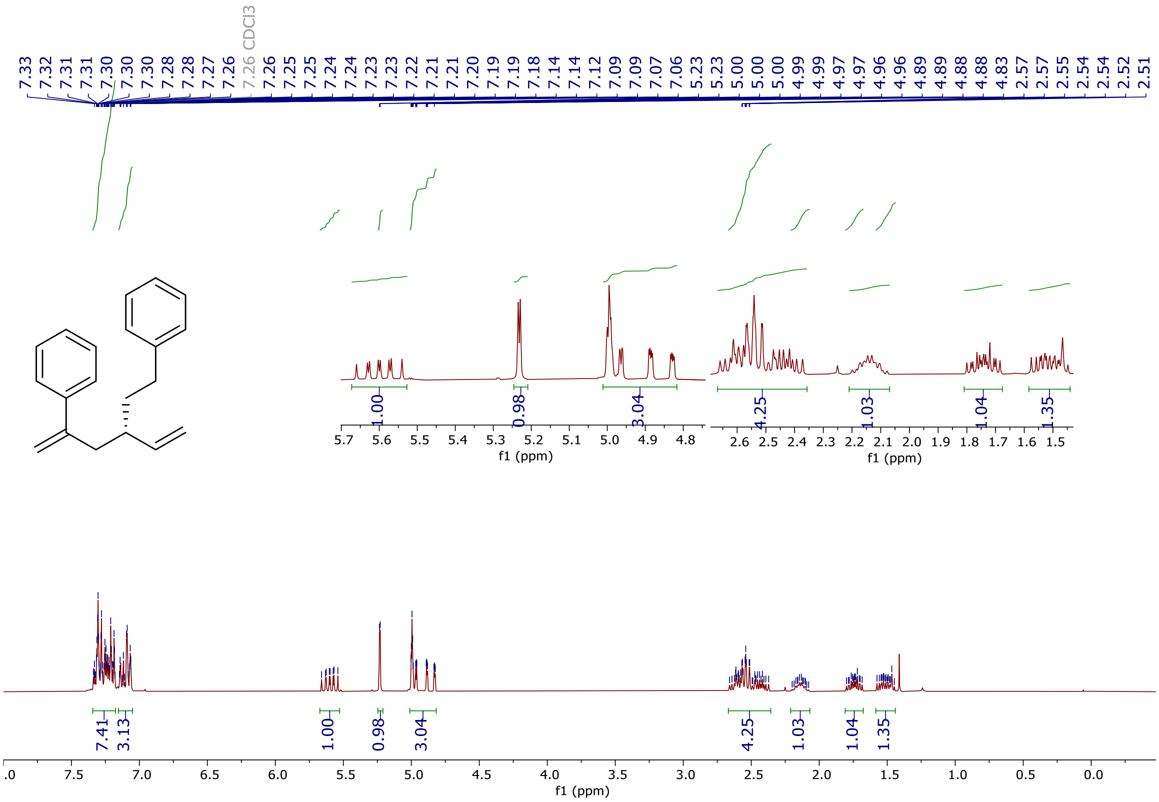

^{13}C NMR (76 MHz, $CDCl_3$) of compound **4c**

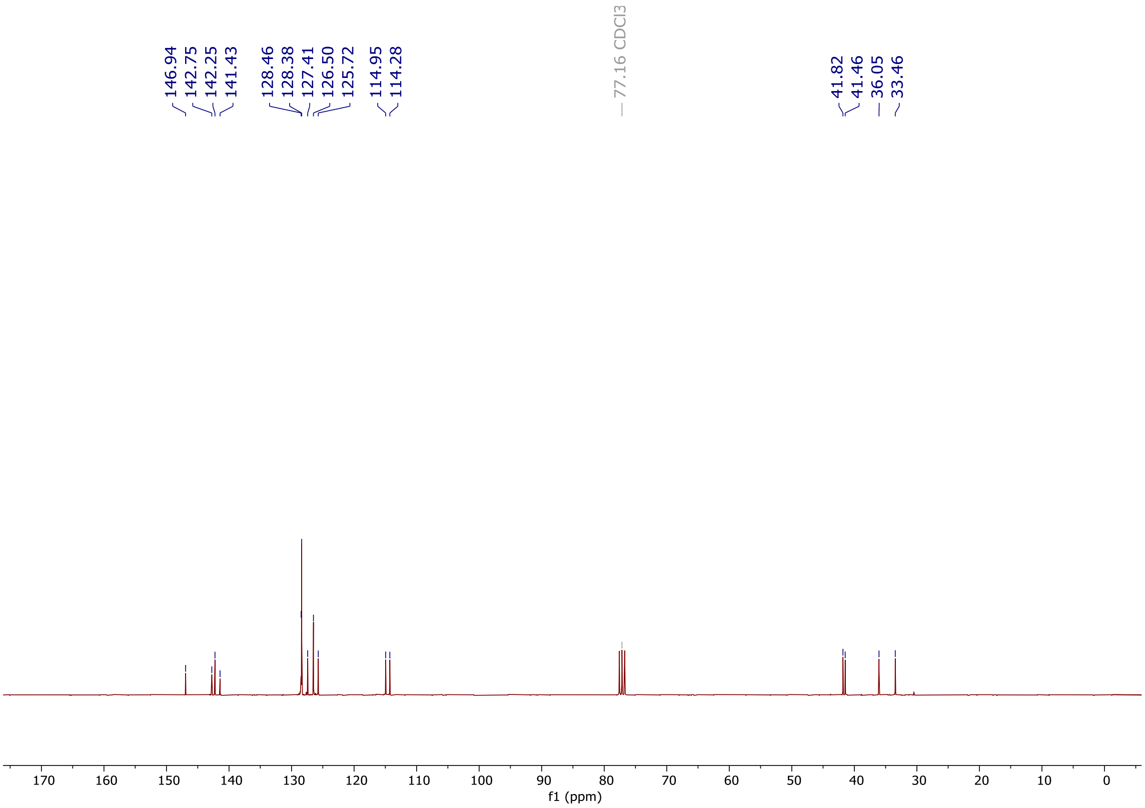

^{1}H NMR (300 MHz, $CDCl_3$) of compound **4d**

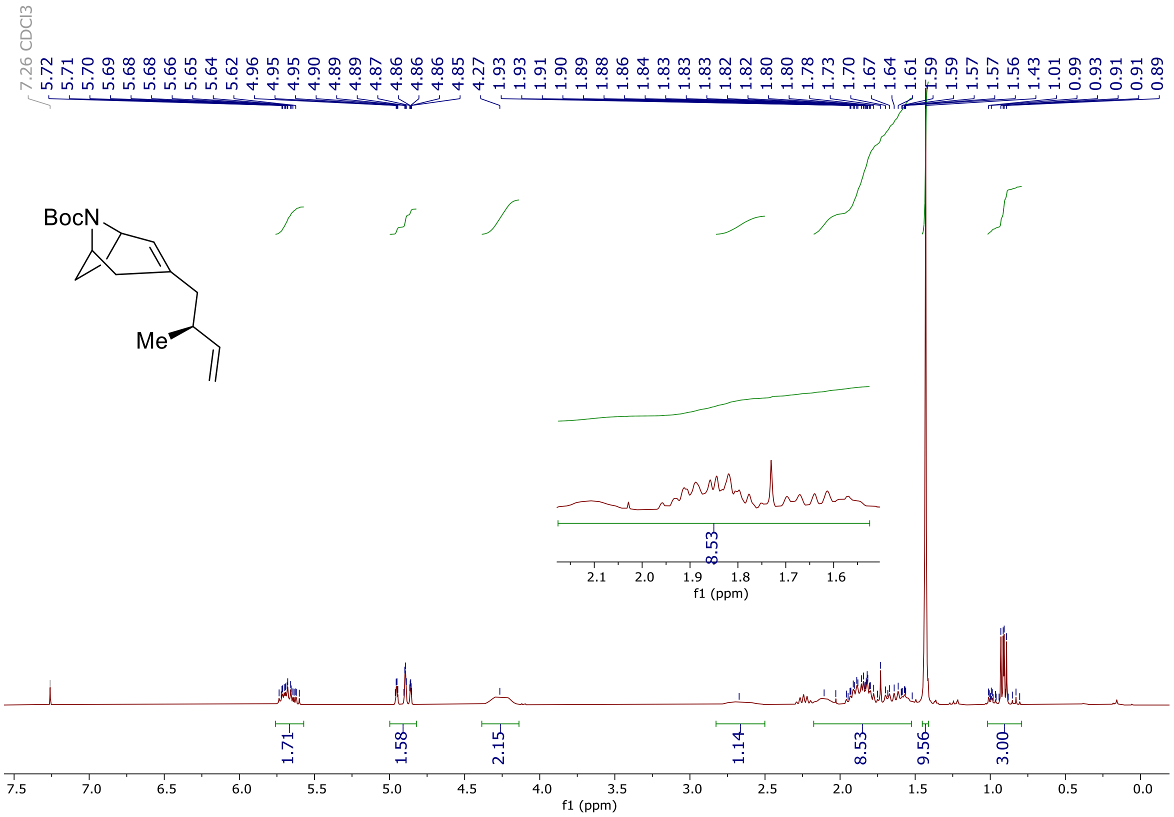

^{13}C NMR (76 MHz, $CDCl_3$) of compound **4d**

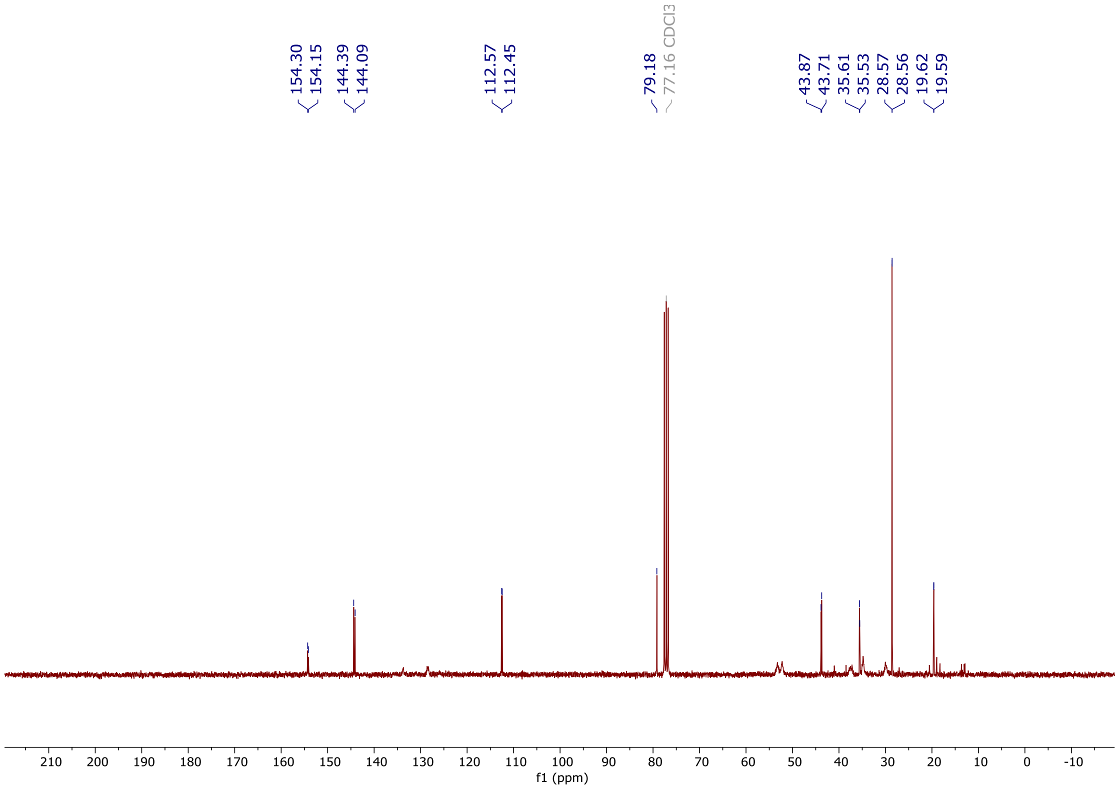

^{1}H NMR (300 MHz, $CDCl_3$) of compound **4e**

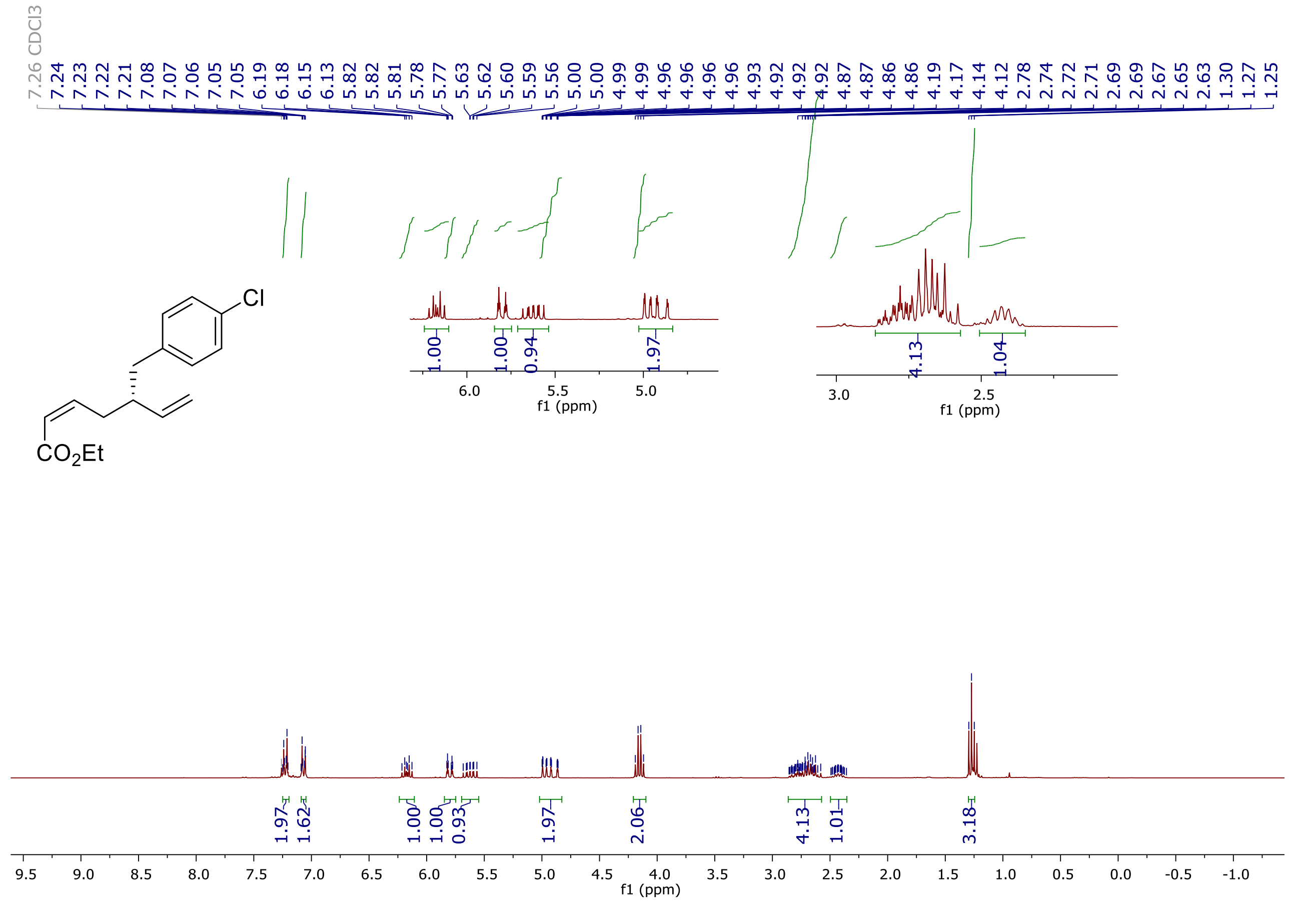

^{13}C NMR (126 MHz, $CDCl_3$) of compound **4e**

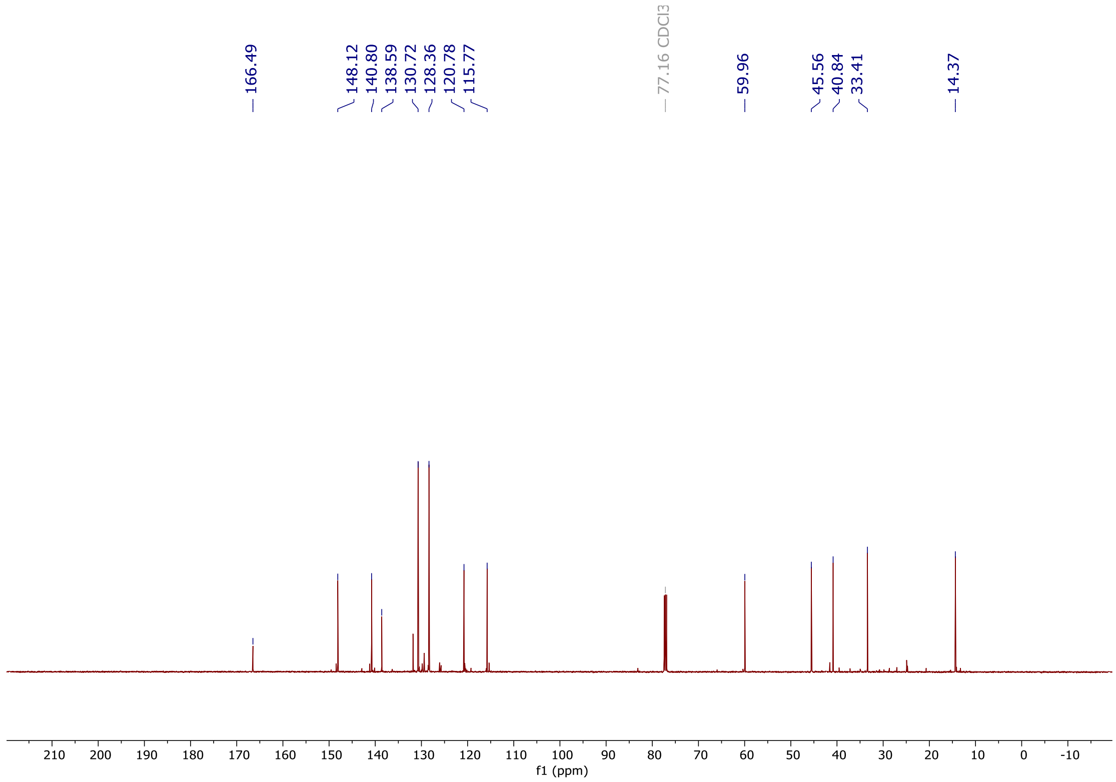

^{1}H NMR (500 MHz, $CDCl_3$) of compound **4f**

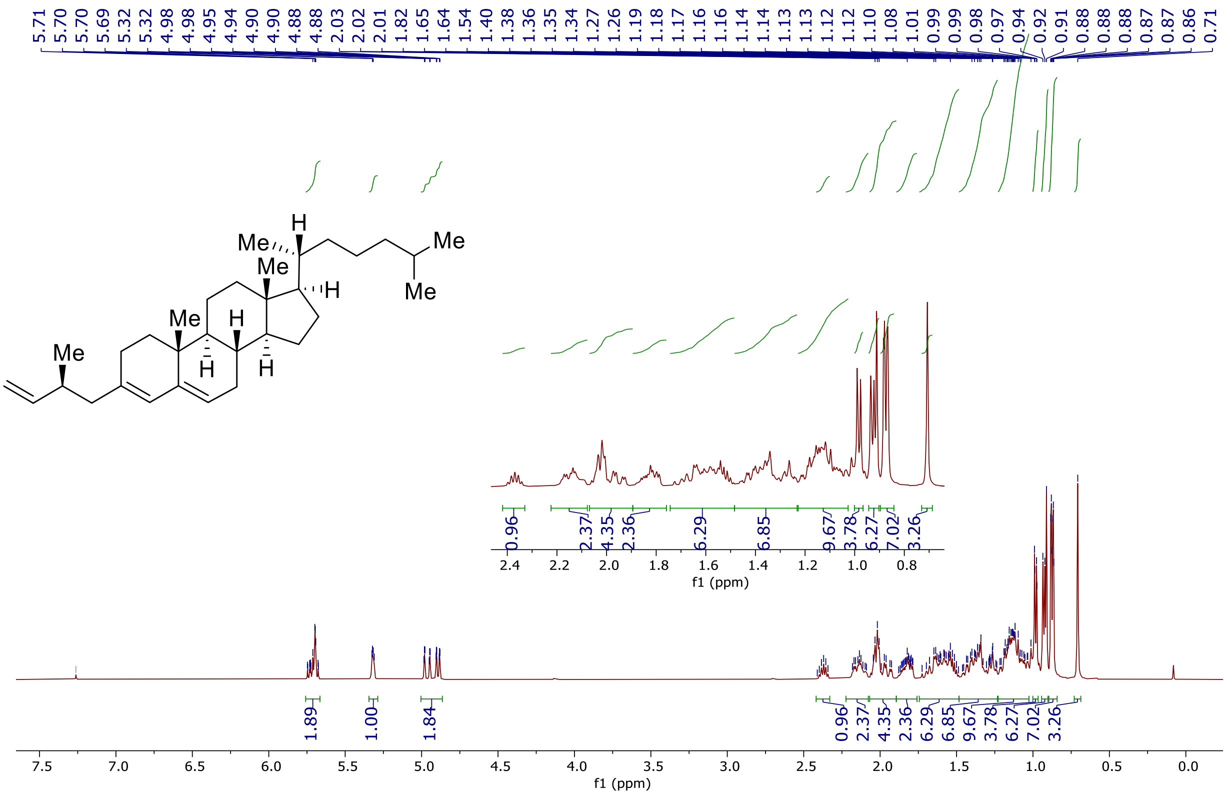

^{13}C NMR (126 MHz, $CDCl_3$) of compound **4f**

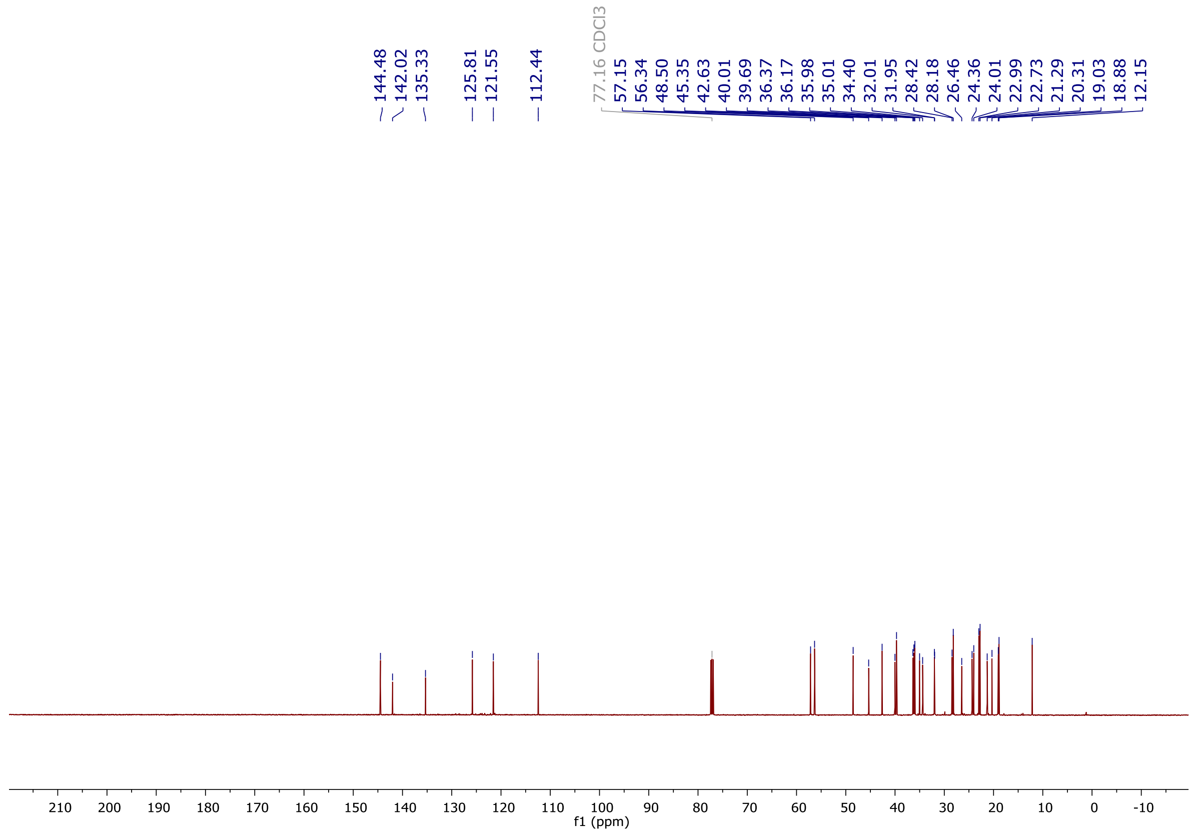

^{1}H NMR (300 MHz, $CDCl_3$) of compound **4g**

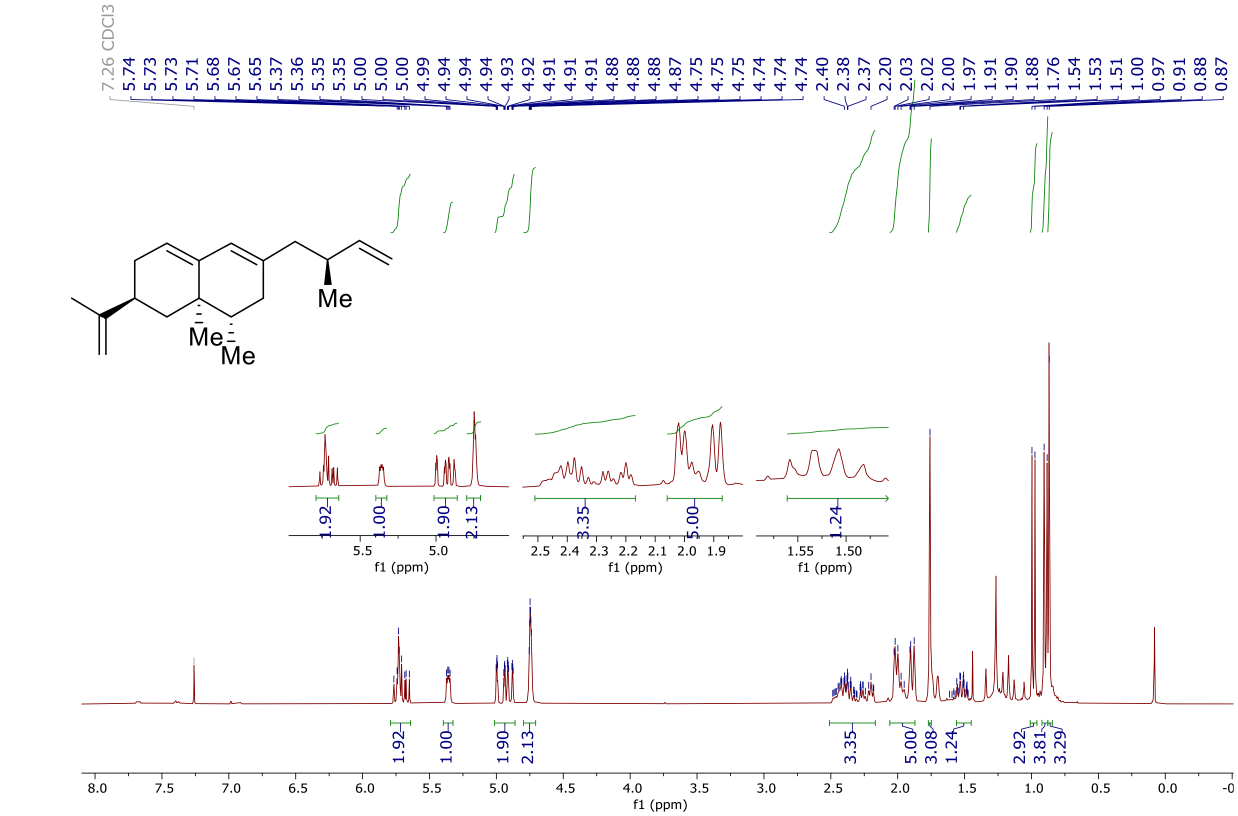

^{13}C NMR (76 MHz, $CDCl_3$) of compound **4g**

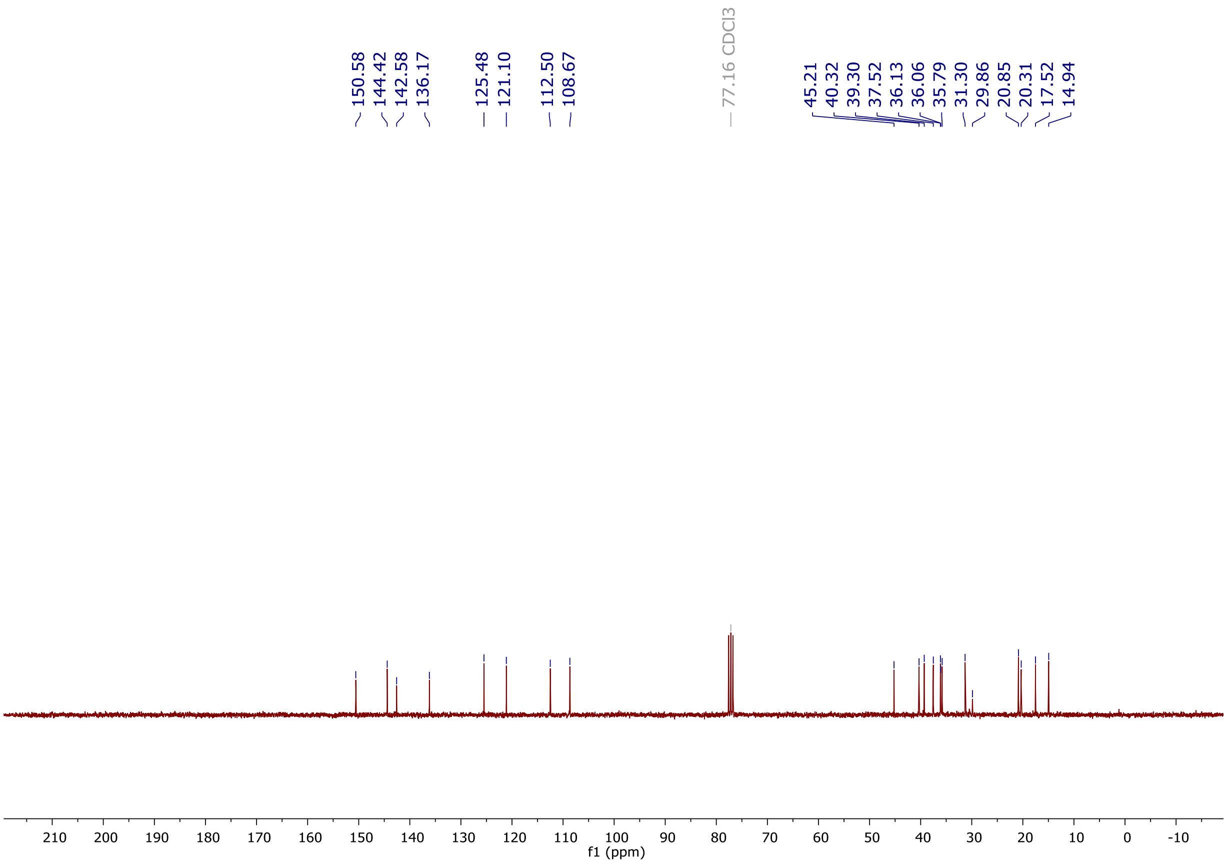

^{1}H NMR (300 MHz, $CDCl_3$) of compound **4h**

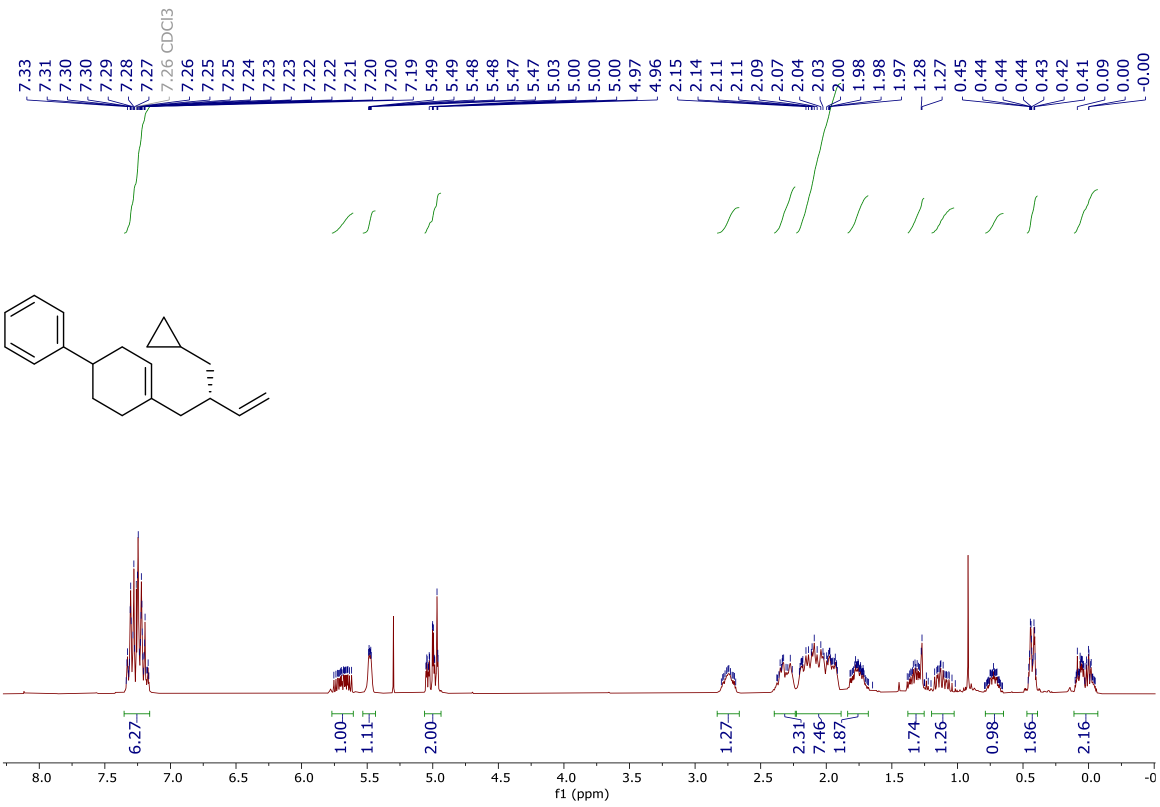

^{13}C NMR (126 MHz, $CDCl_3$) of compound **4h**

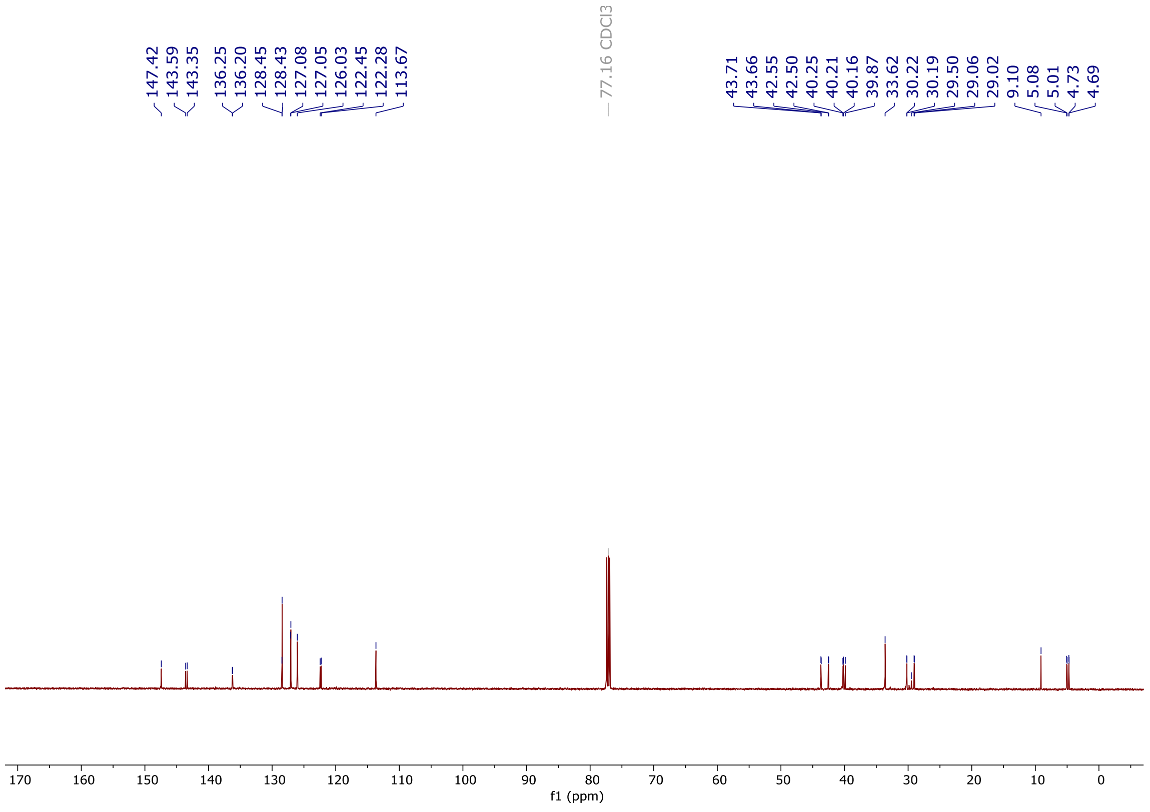

¹H NMR (300 MHz, $CDCl_3$) of compound **4i**

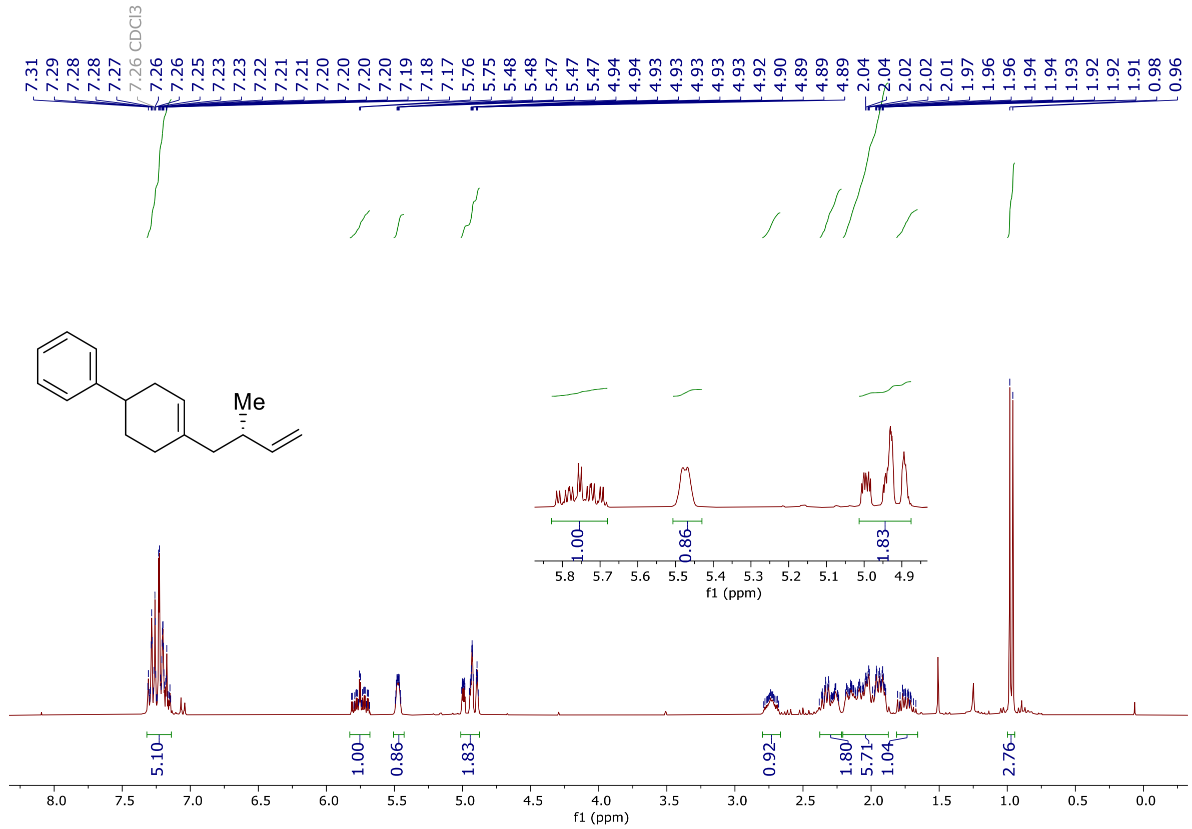

¹³C NMR (76 MHz, $CDCl_3$) of compound **4i**

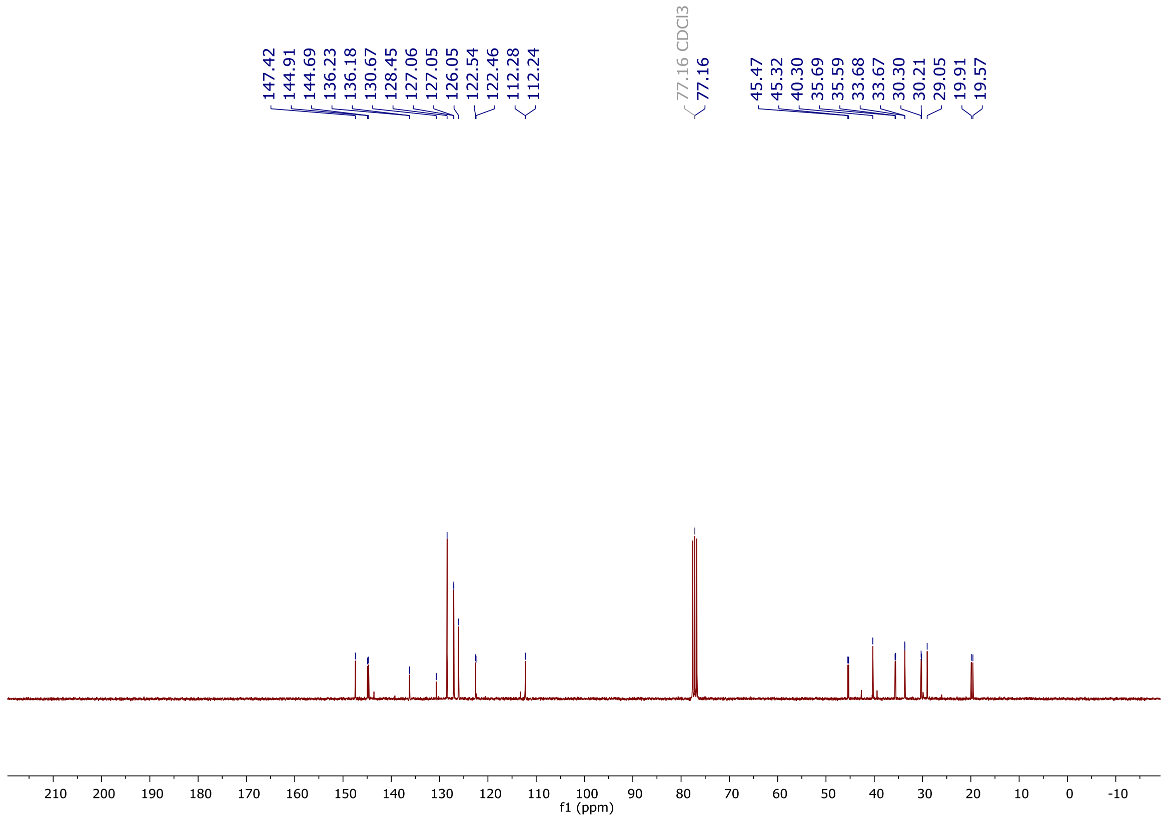

^{1}H NMR (300 MHz, $CDCl_3$) of compound **4j**

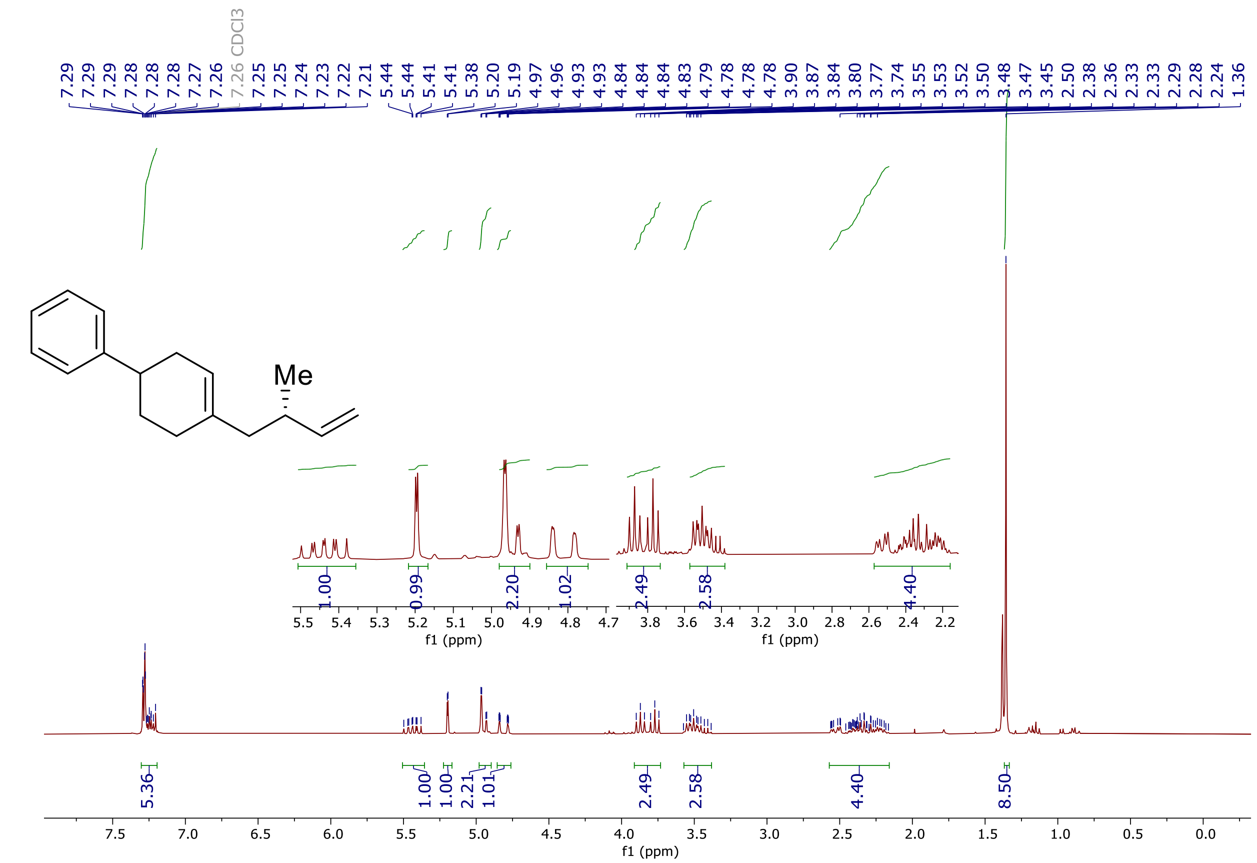

^{13}C NMR (126 MHz, $CDCl_3$) of compound **4j**

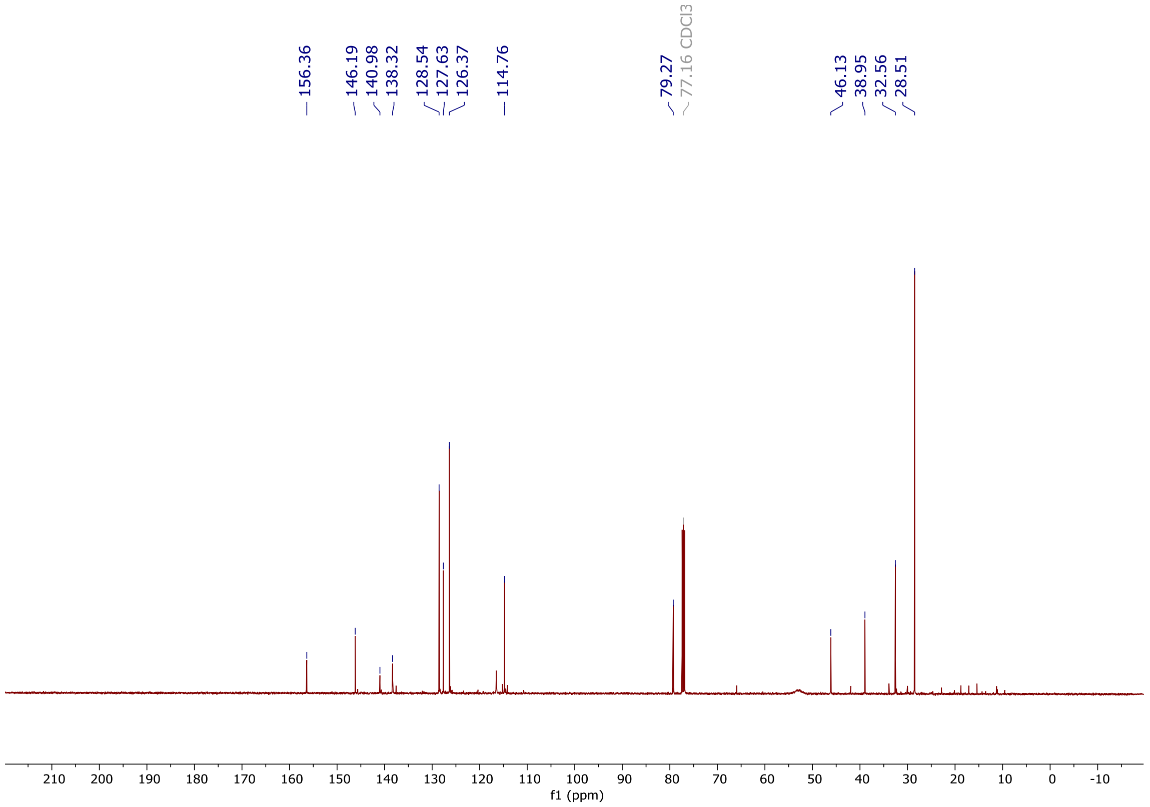

^{1}H NMR (500 MHz, CDCl$_3$) of compound **S4**

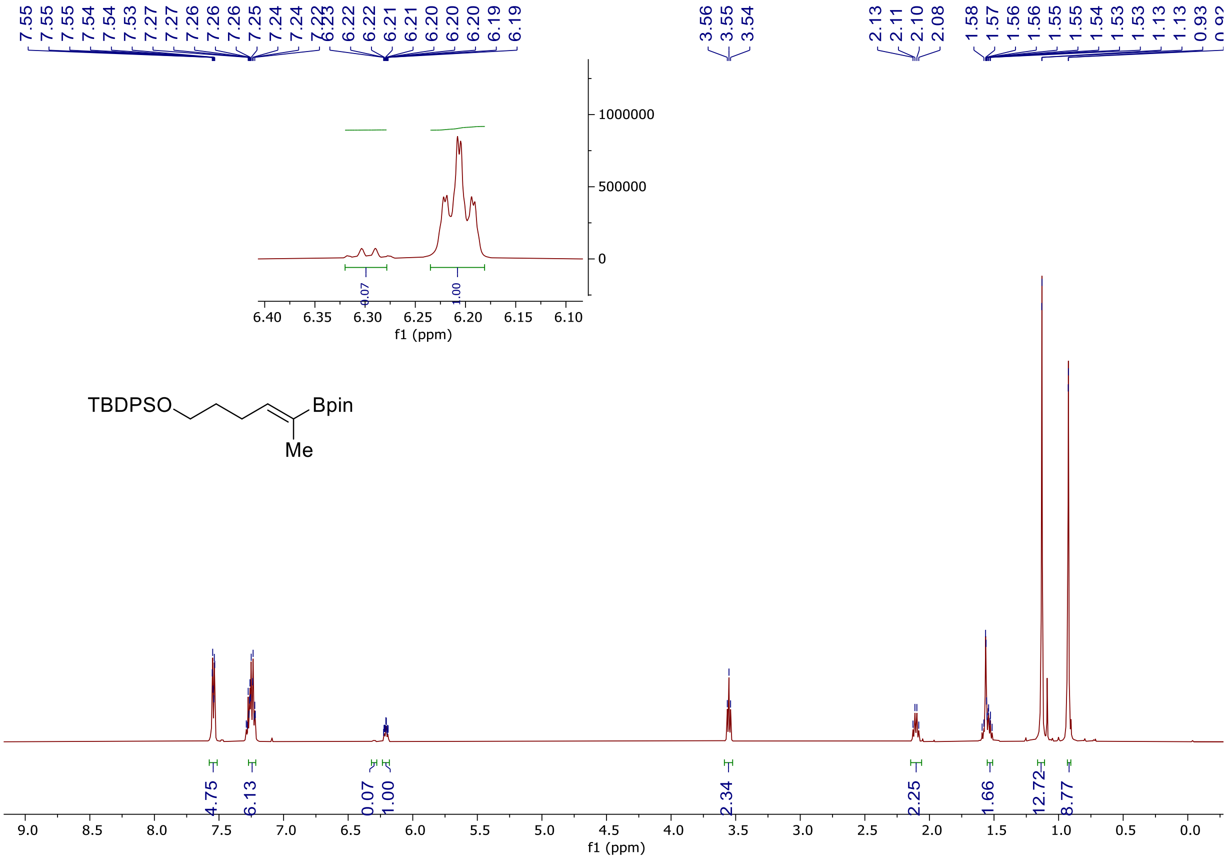

^{13}C NMR (76 MHz, CDCl$_3$) of compound **S4**

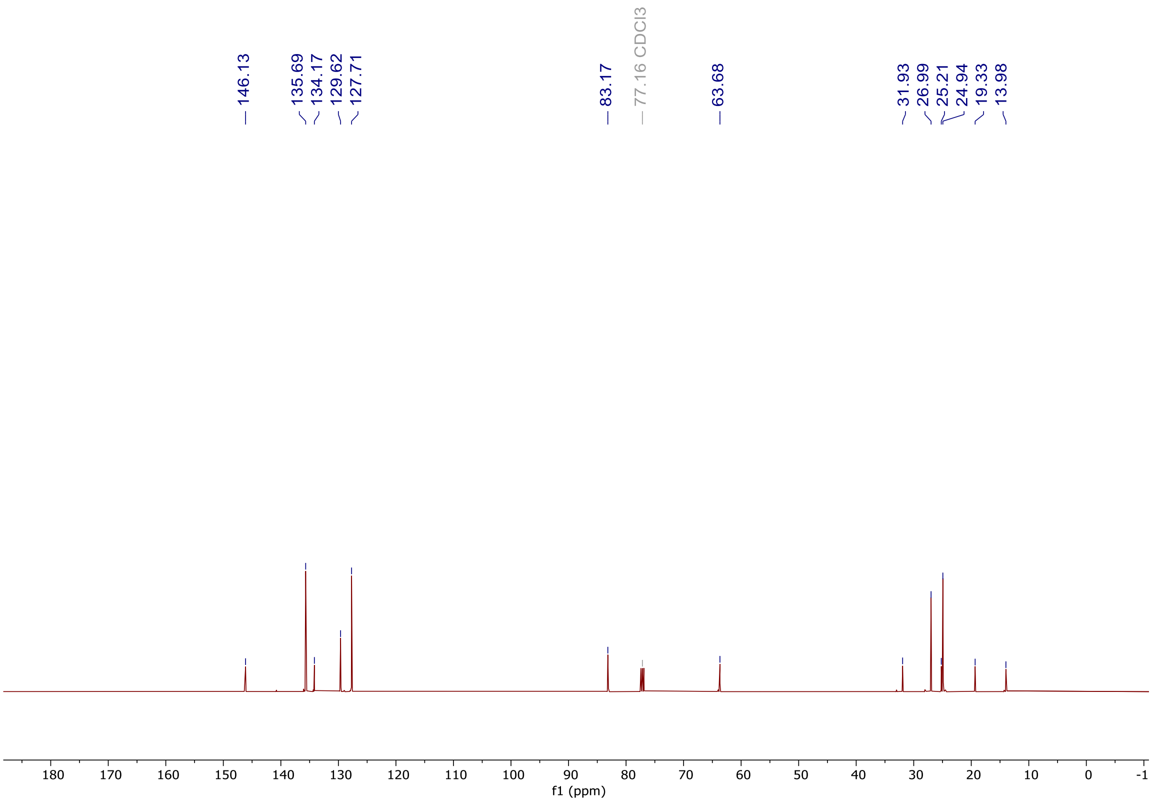

^{11}B NMR (160 MHz, $CDCl_3$) of compound **S4**

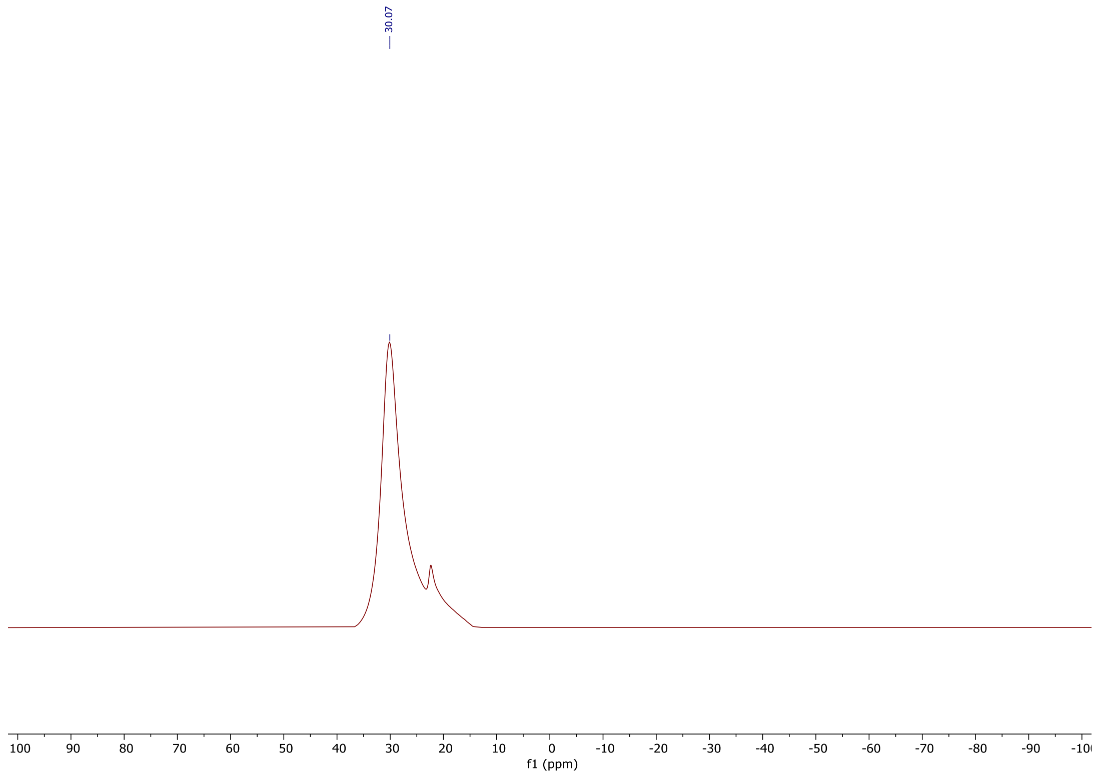

^{1}H NMR (300 MHz, $CDCl_3$) of compound **S5**

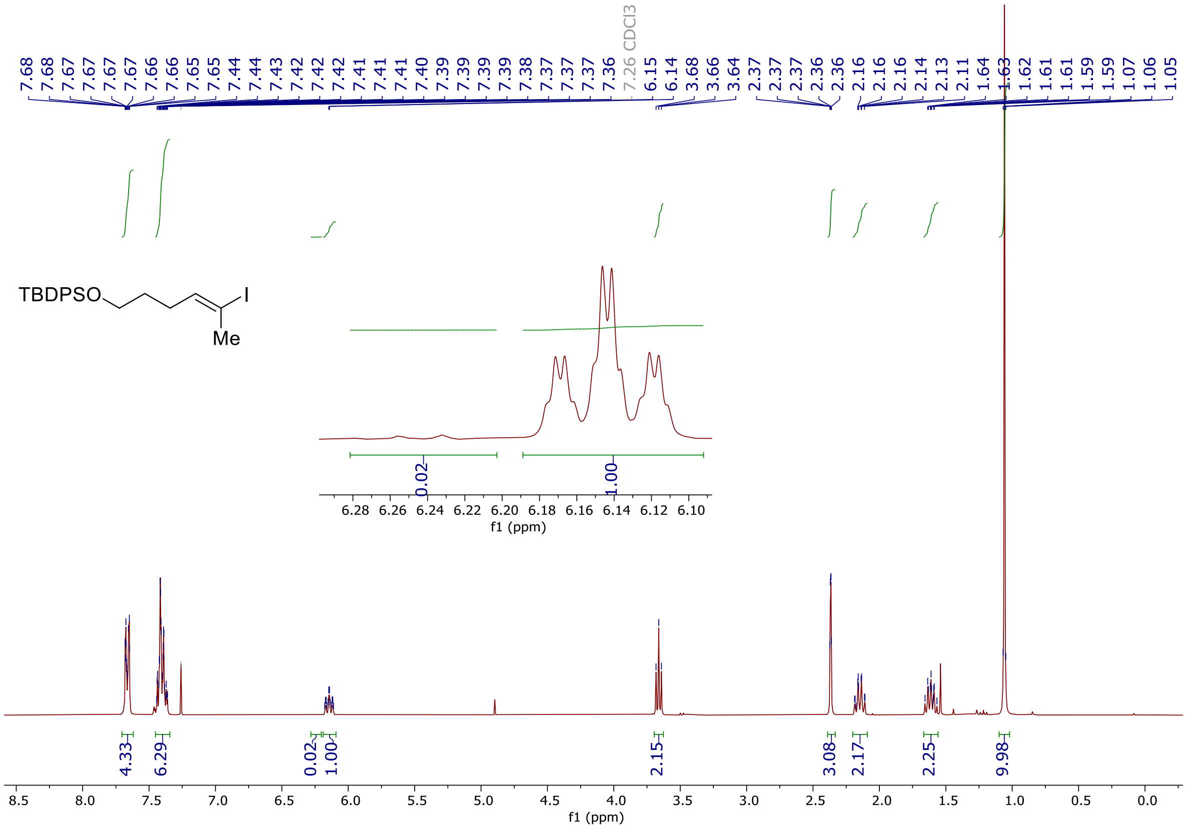

^{13}C NMR (76 MHz, $CDCl_3$) of compound **S5**

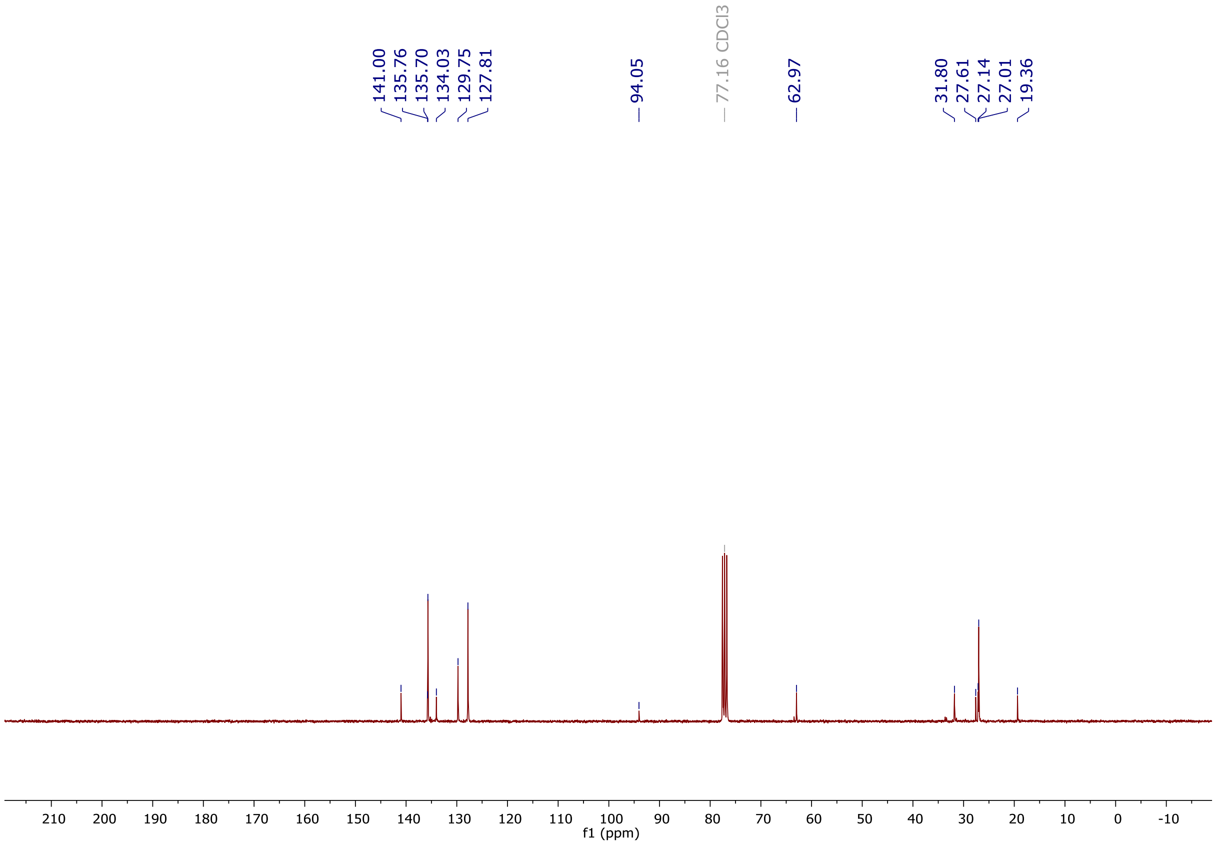

^{1}H NMR (300 MHz, $CDCl_3$) of compound **4k**

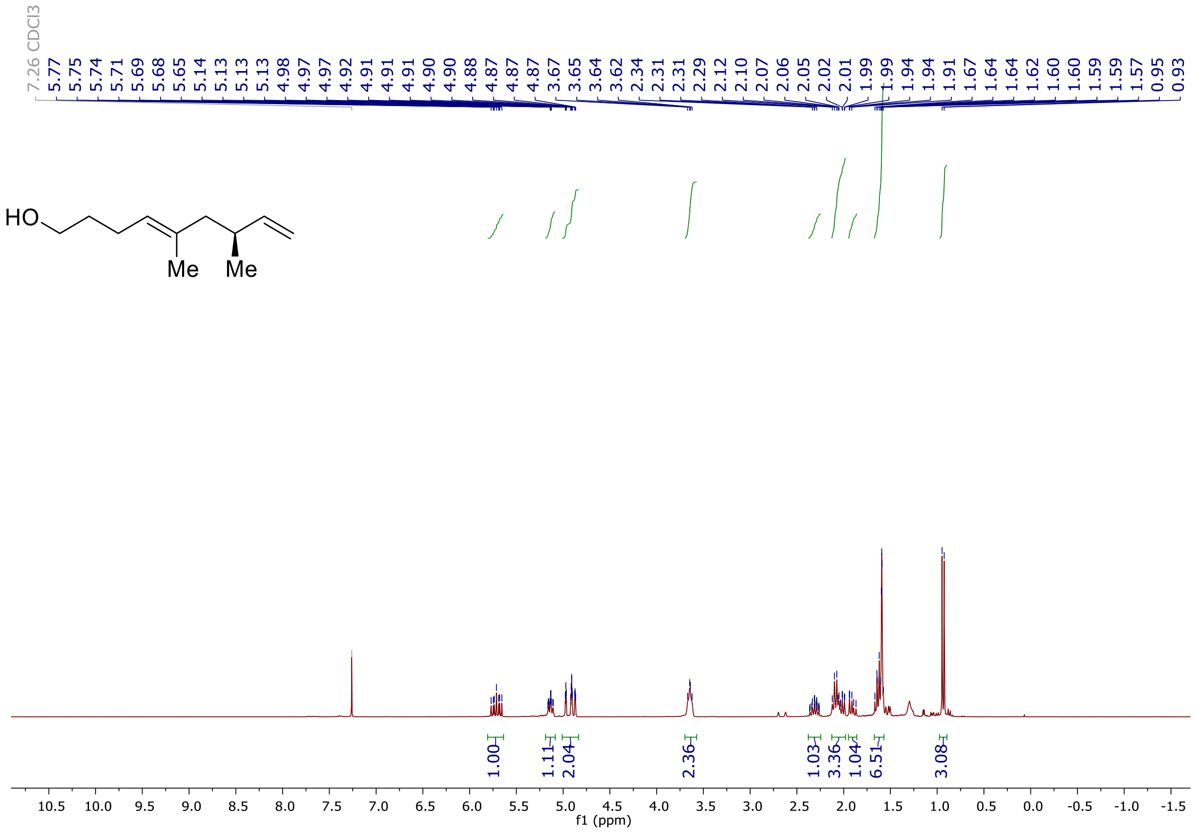

^{13}C NMR (76 MHz, $CDCl_3$) of compound **4k**

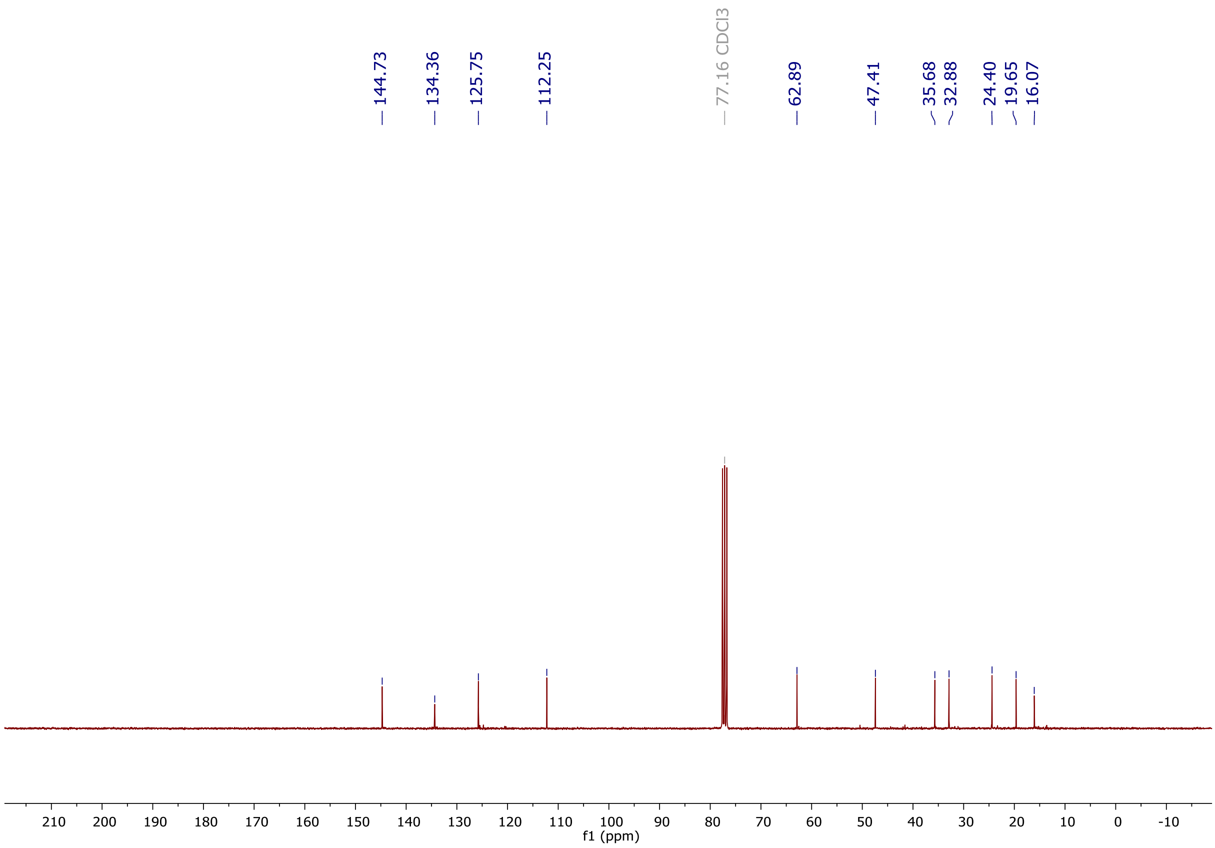

^{1}H NMR (300 MHz, $CDCl_3$) of compound **S6**

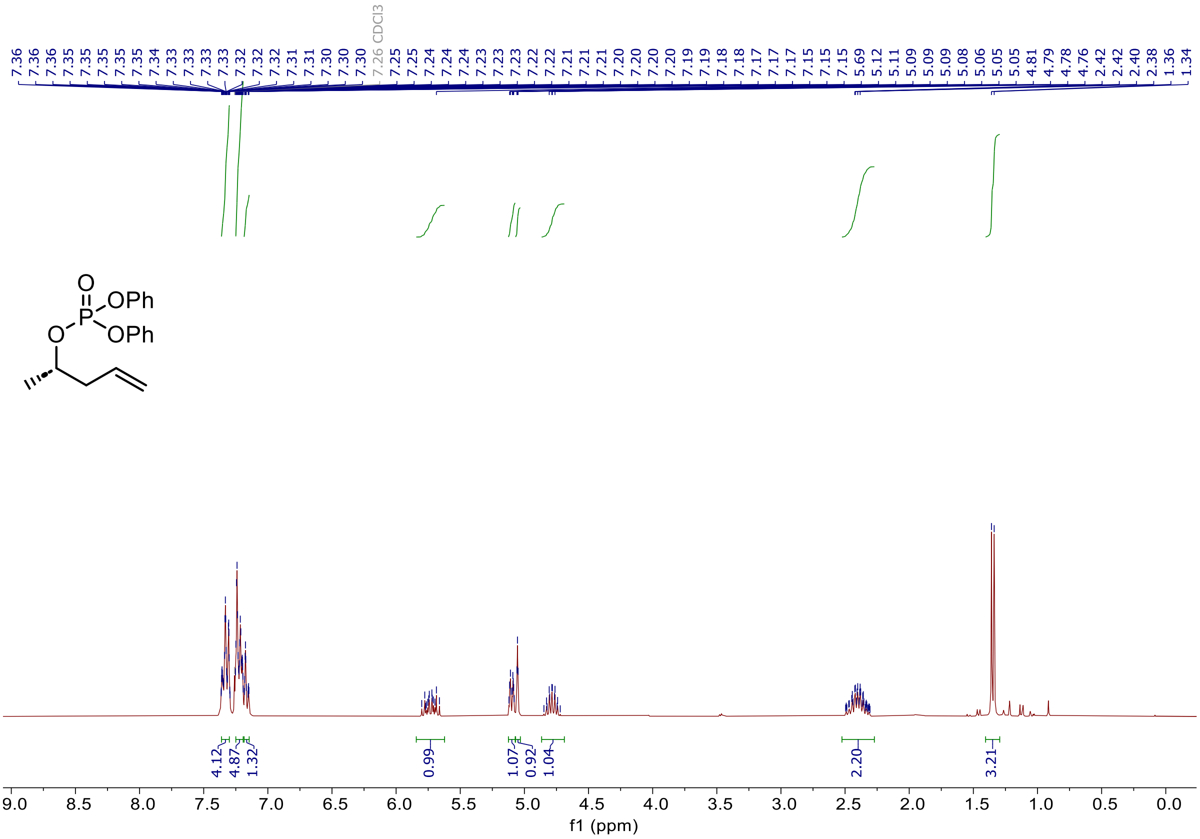

^{13}C NMR (76 MHz, $CDCl_3$) of compound **S6**

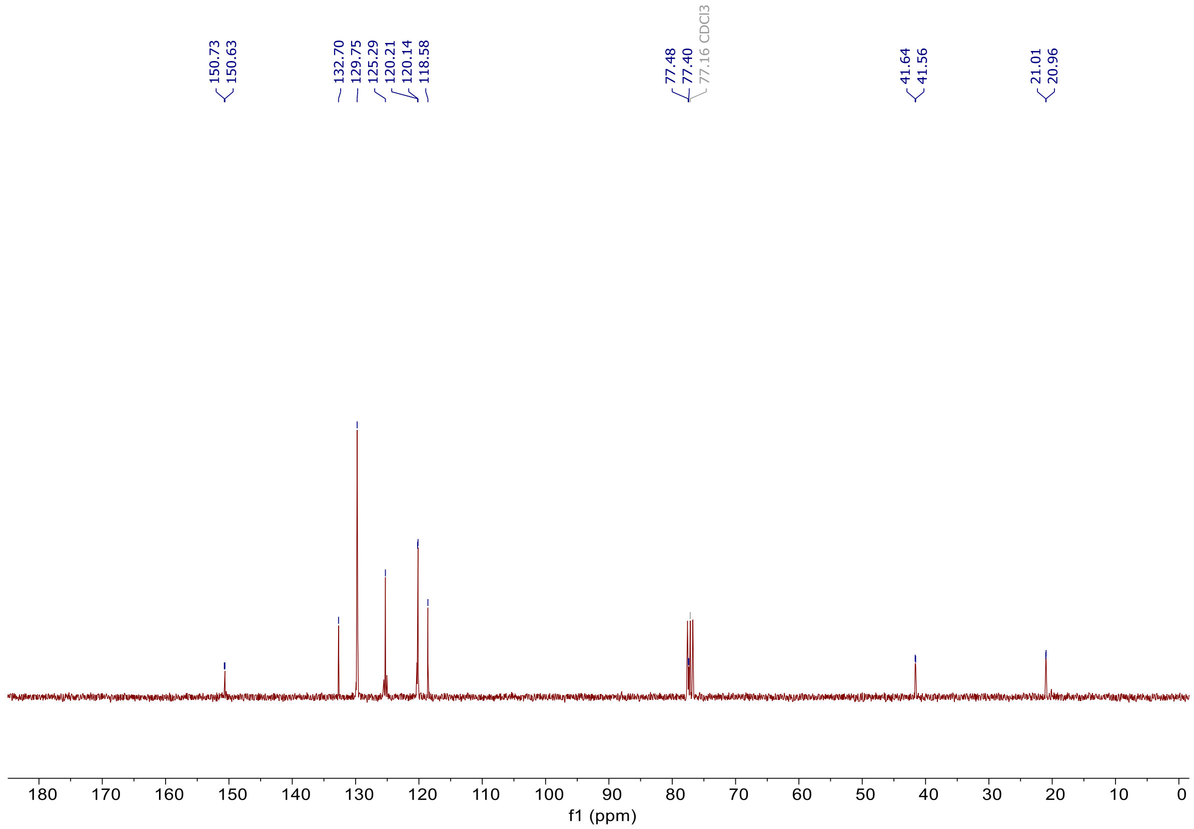

^{31}P NMR (121 MHz, $CDCl_3$) of compound **S6**

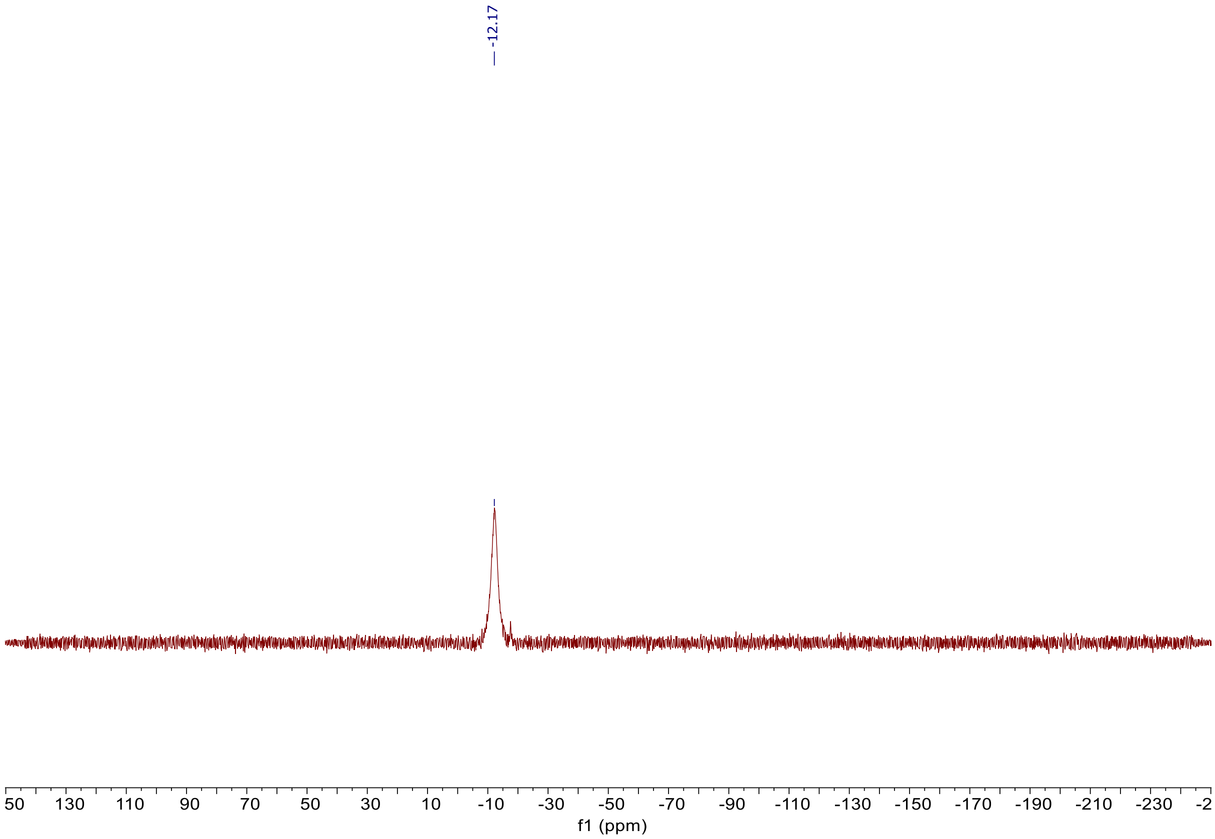

^{1}H NMR (300 MHz, $CDCl_3$) of compound **S7**

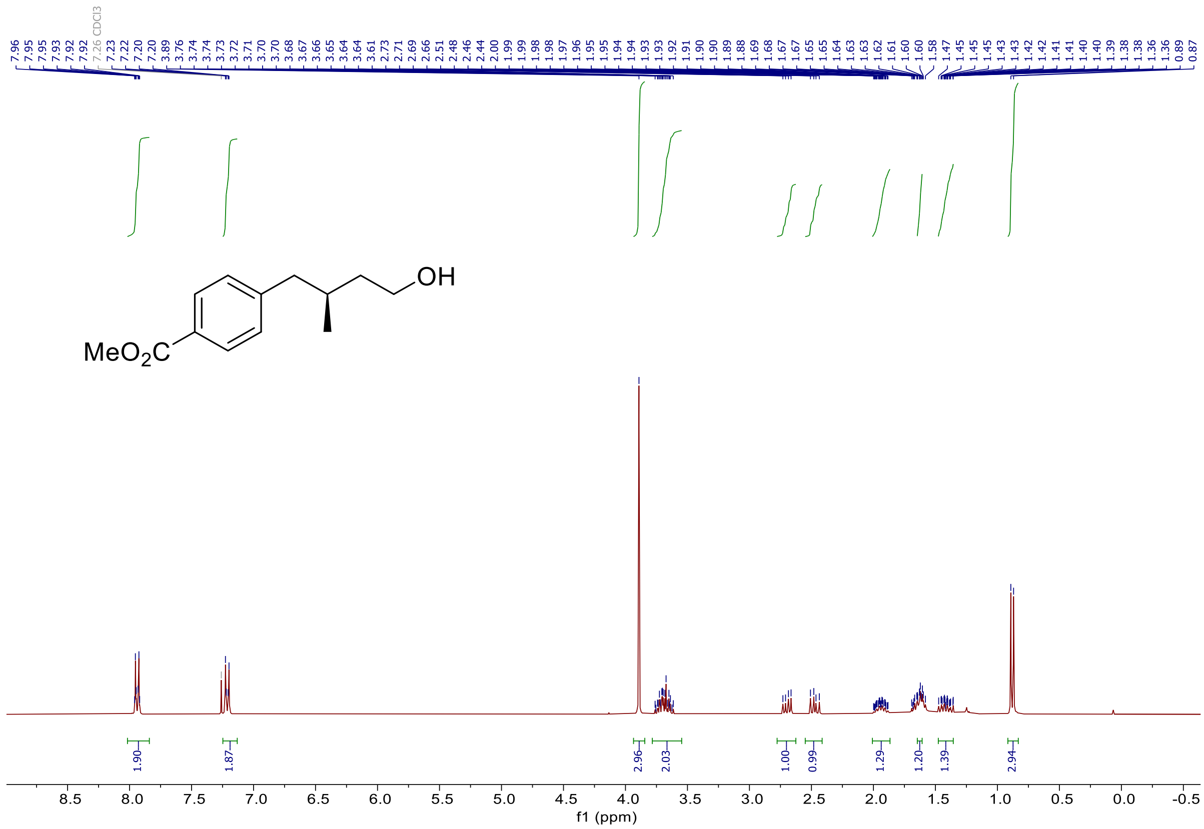

^{13}C NMR (75 MHz, $CDCl_3$) of compound **S7**

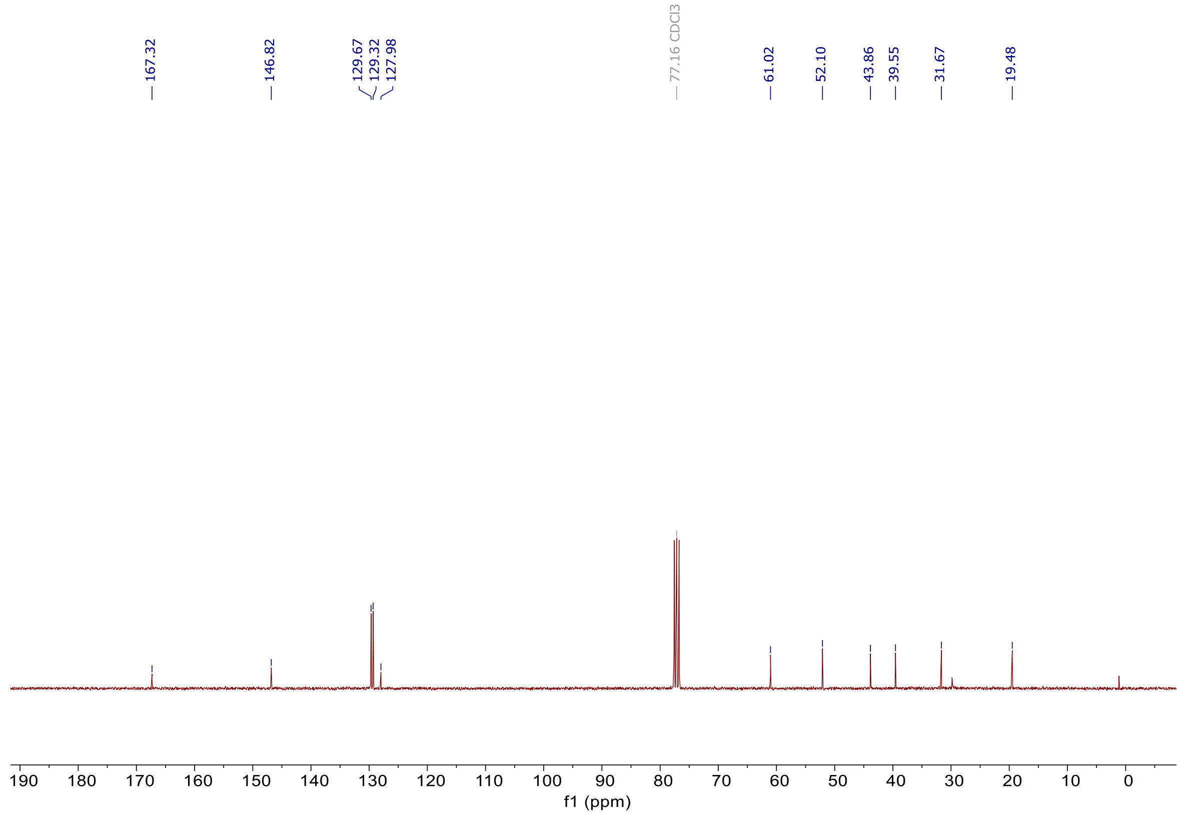

^{1}H NMR (300 MHz, $CDCl_3$) of compound **S8**

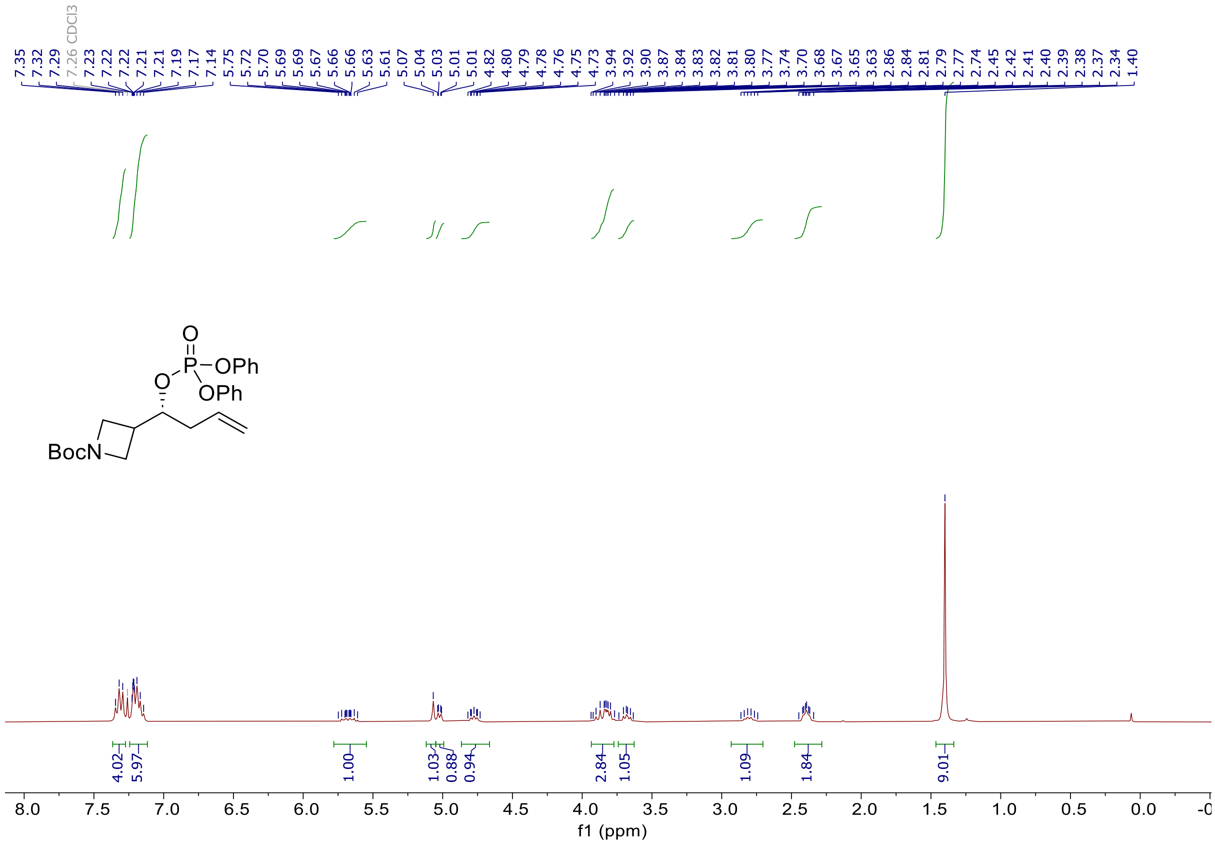

^{13}**C NMR** (75 MHz, $CDCl_3$) of compound **S8**

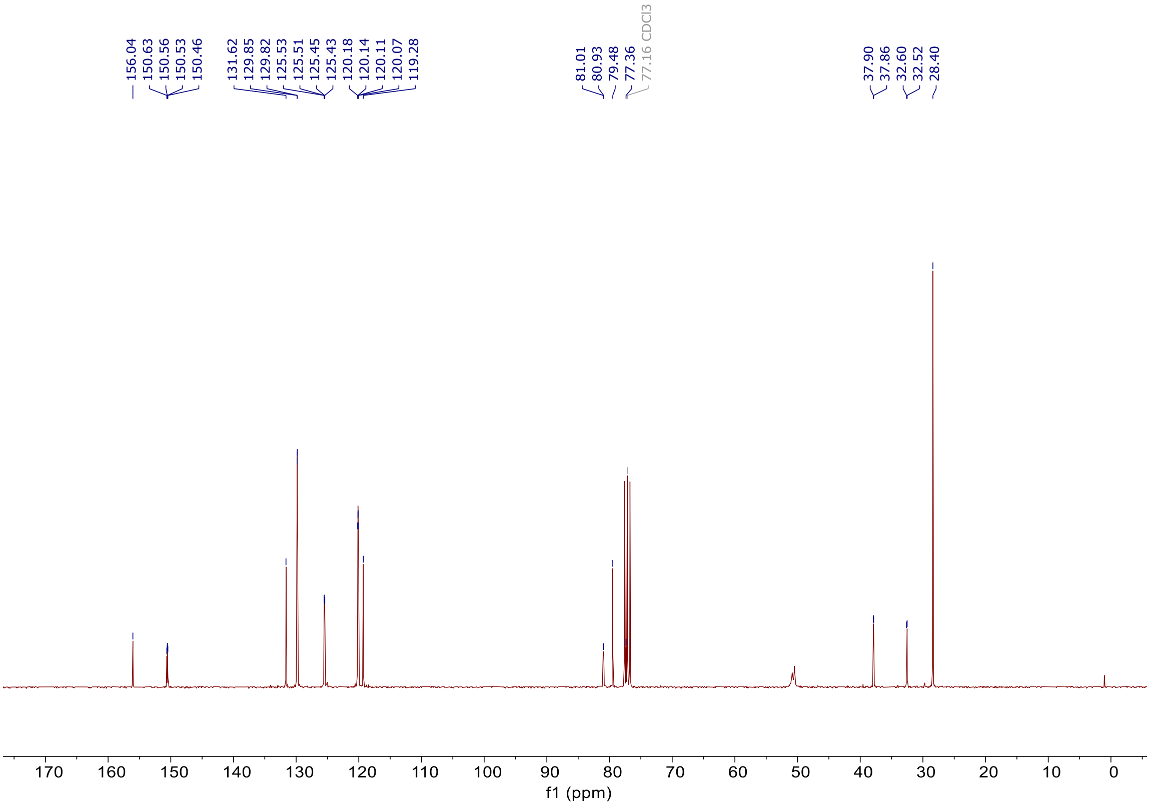

^{31}**P NMR** (122 MHz, $CDCl_3$) of compouns **S8**

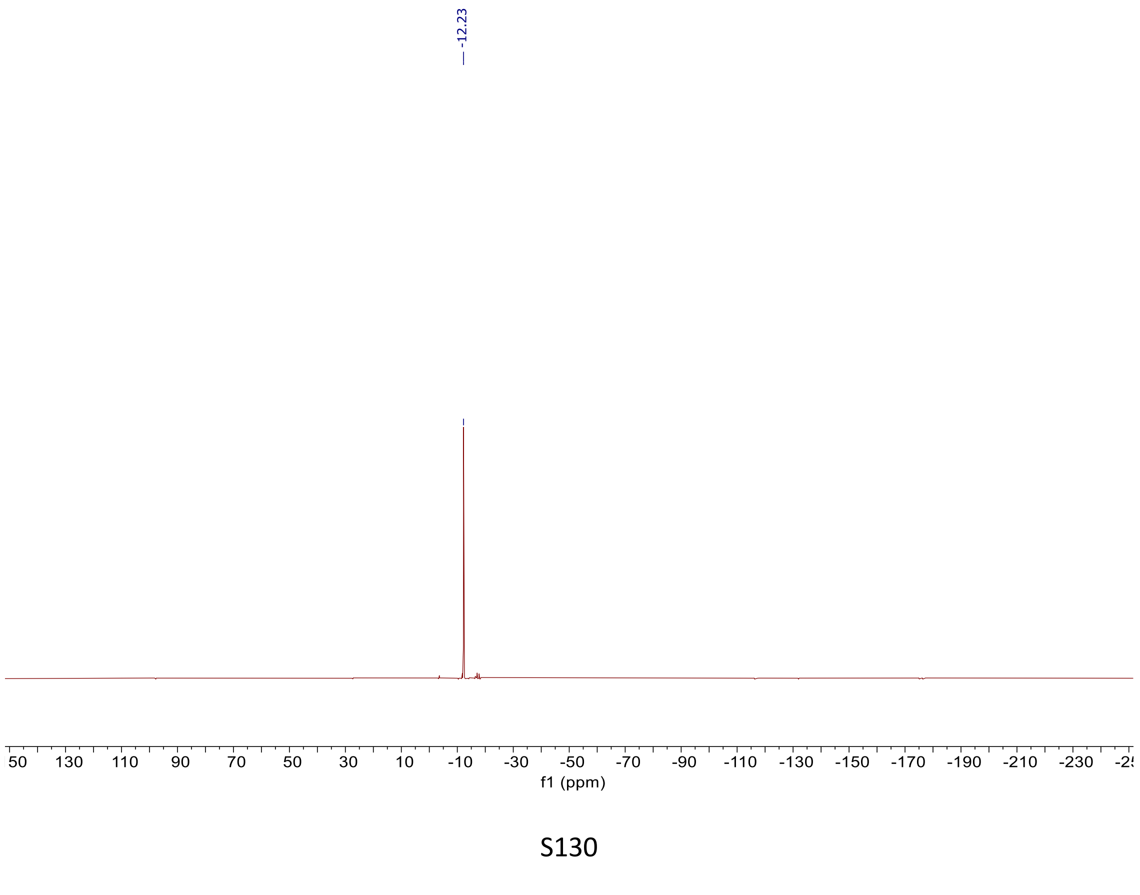

Supplement: Supplementary file 1 [file ja5c15781_si_001.pdf]
